# Antimalarials with Benzothiophene Moieties as Aminoquinoline Partners

**DOI:** 10.3390/molecules22030343

**Published:** 2017-02-24

**Authors:** Jelena Konstantinović, Milica Videnović, Jelena Srbljanović, Olgica Djurković-Djaković, Katarina Bogojević, Richard Sciotti, Bogdan Šolaja

**Affiliations:** 1 Faculty of Chemistry, University of Belgrade, Studentski trg 16, P.O. Box 51, 11158 Belgrade, Serbia; jelena_konstantinovic@chem.bg.ac.rs (J.K.); bogojevickatarina@gmail.com (K.B.); 2 Innovation Center of the Faculty of Chemistry, Studentski trg 12-16, 11158 Belgrade, Serbia; milica_videnovic@chem.bg.ac.rs; 3 Institute for Medical Research, University of Belgrade, Dr. Subotića 4, 11129 Belgrade, Serbia; jelena.srbljanovic@imi.bg.ac.rs (J.S.); olgicadj@imi.bg.ac.rs (O.D.-D.); 4 Experimental Therapeutics Branch, Walter Reed Army Institute of Research, Silver Spring, MD 20910, USA; richard.j.sciotti.civ@mail.mil

**Keywords:** antimalarials, thiophene, benzothiophene, aminoquinoline

## Abstract

Malaria is a severe and life-threatening disease caused by *Plasmodium* parasites that are spread to humans through bites of infected *Anopheles* mosquitoes. Here, we report on the efficacy of aminoquinolines coupled to benzothiophene and thiophene rings in inhibiting *Plasmodium falciparum* parasite growth. Synthesized compounds were evaluated for their antimalarial activity and toxicity, in vitro and in mice. Benzothiophenes presented in this paper showed improved activities against a chloroquine susceptible (CQS) strain, with potencies of IC_50_ = 6 nM, and cured 5/5 *Plasmodium berghei* infected mice when dosed orally at 160 mg/kg/day × 3 days. In the benzothiophene series, the examined antiplasmodials were more active against the CQS strain D6, than against strains chloroquine resistant (CQR) W2 and multidrug-resistant (MDR) TM91C235. For the thiophene series, a very interesting feature was revealed: hypersensitivity to the CQR strains, resistance index (RI) of <1. This is in sharp contrast to chloroquine, indicating that further development of the series would provide us with more potent antimalarials against CQR strains.

## 1. Introduction

Malaria is a severe and life-threatening disease caused by *Plasmodium* parasites that are spread to humans through bites of infected *Anopheles* mosquitoes. *Plasmodium* sporozoites injected into the bloodstream travel to the liver, where they are transformed into merozoites. They later re-enter the bloodstream, attacking the red blood cells where they begin the asexual replication. A few of these merozoites develop into sexual forms—gametocytes—which are being taken up by mosquitos during blood feeding, thus completing their life cycle [1]. This suggests that numerous stages of the life cycle of *Plasmodium* parasite could be potential drug targets for development of new antimalarials.

According to the 2015 World Health Organization (WHO) report it is estimated that 214 million cases of malaria occurred globally and the disease led to 438,000 deaths [2]. As artemisinin resistance has spread, posing a threat to malaria control [3], in order to prevent progression to life-threatening malaria artemisinin-based combination therapies (ACTs) have been recommended by the WHO [4]. A vaccine has yet to be discovered [5] and taking into account the constant loss of therapeutic efficacy due to the drug resistant strains, there is an urgent need for the discovery of new targets and treatments for malaria. Widespread chloroquine (CQ) resistance is largely attributable to mutations in the vacuole transmembrane protein (PfCRT) protein [6,7], found in the membrane of the digestive vacuole where hemoglobin digestion takes place.

A number of potential drug targets for novel antimalarials are known so far, most of them parasite proteins, such as *Plasmodium falciparum* enoyl-acyl carrier protein reductase [8,9,10], fatty acid synthase (PfFAS) [11], *N*-myristoyltransferase (NMT) [12,13], hexose transporter (PfHT1) [14,15,16], serine hydroxymethyltransferase (SHMT) [17], histone deacetylase [18], and many others. It is unclear if inhibition of these enzymes is the only mechanism of action (MOA) through which novel drugs inhibit parasite growth, but they are important in discovering enzyme function and metabolic pathways. Aminoquinolines, such as chloroquine, are well-known antimalarials. One of the hypotheses of their MOA is the accumulation in the parasite food vacuole, leading to inhibition of hemozoin formation. The toxic effect of free hematin containing ferriprotoporphyrin IX system is suppressed by “polymerization” into hemozoin; therefore, drugs that act as inhibitors of hematin sequestration are involved in the death of the parasite [19]. On the other hand, molecules that contain benzothiophene cores have an important role in medicinal chemistry due to their various biological properties, including antibacterial, antifungal and antitubercular activities [20]. They also act as inhibitors of mitogen activated protein kinase-activated protein kinase 2 (MK2) [21,22], C_17,20_-lyase inhibitors [23], liver receptor homolog-1 (LRH-1) antagonists [24], inhibitors of tyrosine phosphatase 1B and antihyperglycemic agents [25]. Regarding antimalarial activity, only one series of benzothiophene derivatives was evaluated and showed IC_50_ values up to 0.16 μM for *P. falciparum* 3D7 strain and inhibited parasite growth up to 65% at 50 mg/kg/day × 4 days in *P. berghei* infected mice [26].

Now, we report on the efficacy of aminoquinolines coupled to benzothiophene and thiophene rings in inhibiting *Plasmodium falciparum* parasite growth and compare the obtained results with CQ and previously reported related thiophene and benzothiophene-based 4-amino-7-chloroquinoline derivatives [27,28], as well as other antimalarial benzothiophene derivatives.

**Figure 1:**
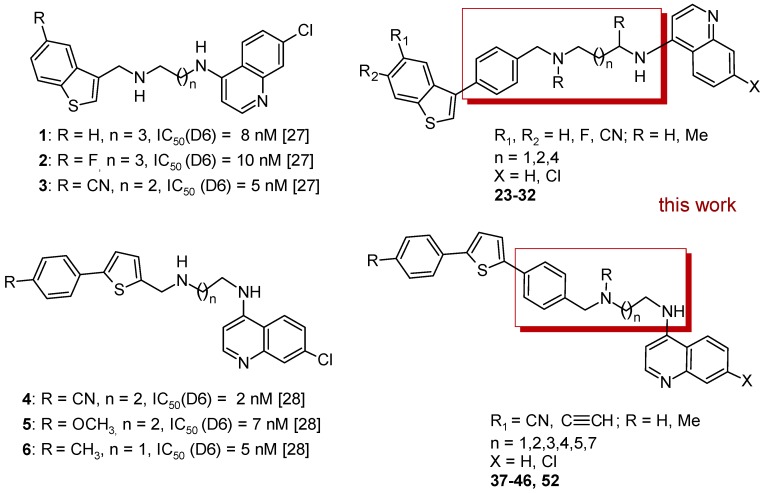
Benzothiophene and thiophene based antimalarials **23**–**32**, **37**–**46** and **52**.

The new series possesses an additional phenylene linker between aminoquinoline and benzothiophene/thiophene moieties (Figure 1), and the influence of the extra π-system on antiplasmodial activity was analyzed taking into account the influence of different methylene linkers.

## 2. Results

### 2.1. Chemistry

**Scheme 1:**
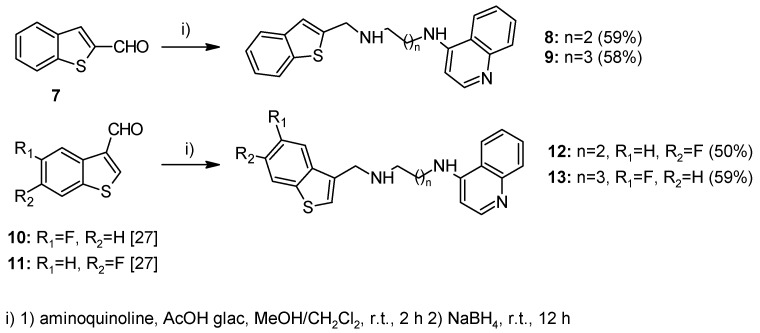
Synthesis of benzothiophene derivatives **8**, **9**, **12** and **13**.

The syntheses of the aminoquinoline antimalarials with a benzothiophene carrier were executed using procedures we developed earlier. Derivatives **8**, **9**, **12** and **13** were obtained by reductive amination, starting from the corresponding aldehydes (Scheme 1).

**Scheme 2:**
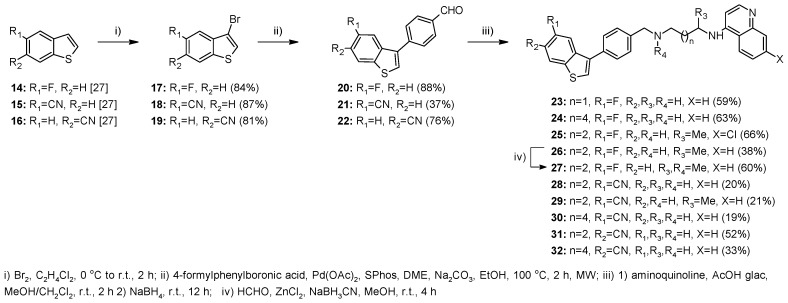
Synthesis of benzothiophene derivatives **23**–**32** with the phenylene linker. SPhos = 2-Dicyclohexylphosphino-2′,6′-dimethoxybiphenyl‎; DME = 1,2-dimetoxyethane

Substituted benzothiophene cores were synthesized starting from commercially available difluoro- or bromofluorobenzaldehyde. Bromination of benzothiophene core afforded C(3) substituted products **17**, **18** and **19** in good yields (>80%, Scheme 2), which were further coupled to 4-formylphenylboronic acid via Suzuki reactions to give the aldehydes **20**, **21** and **22**. These fragments are used in the key reductive amination reaction to prepare a diverse set of methylene-linked *P. falciparum* inhibitors. It is interesting to note that aldehydes **20** and **22** were obtained in good yields (88% and 76%, respectively), in contrast to **21** (37%). Finally, the reductive amination afforded target compounds **23**–**26** and **28**–**32** in 19%–77% isolated yield after column purification. In addition, compound **26** was methylated and gave **27** in moderate yield.

**Scheme 3:**
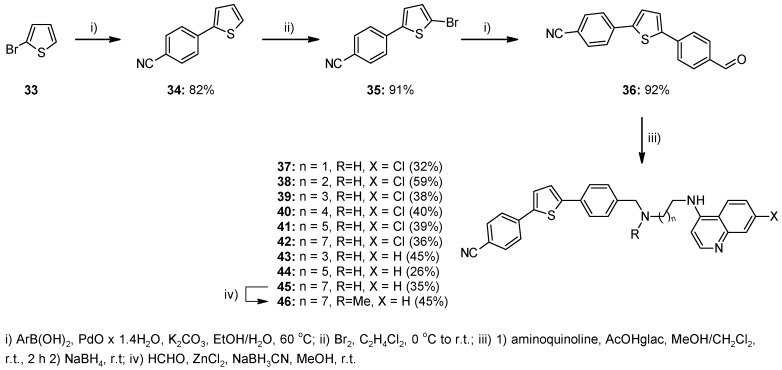
Synthesis of thiophene derivatives **37**–**46**.

In the thiophene series, the key intermediate, aldehyde **36**, was synthesized in few steps starting from commercially available 2-bromothiophene (**33**) as follows: 4-(thiophen-2-yl)benzonitrile (**34**) was obtained from 2-bromothiophene by Suzuki coupling with 4-cyanophenylboronic acid using PdO hydrate as a catalyst (Scheme 3) [29]. Subsequent bromination of thiophene **34** at C(5) position and additional Suzuki reaction with 4-formyphenylboronic acid afforded **36** in excellent yield (92%). After submitting **36** to reductive amination reactions with different aminoquinolines, the target aminoquinoline derivatives **37**–**44** were obtained in 26%–59% isolated yield. The compound **45** was further methylated providing derivative **46** with a tertiary amino group (Scheme 3).

**Scheme 4:**
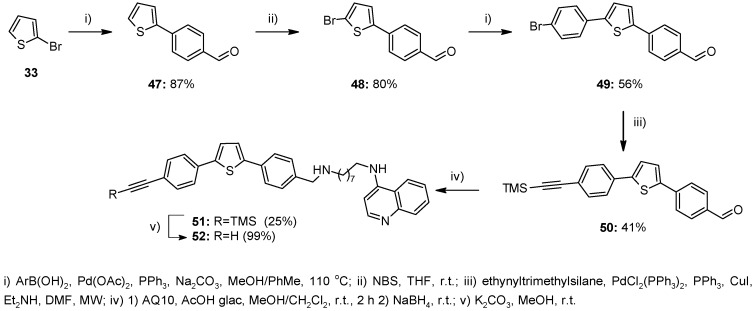
Synthesis of acetylene derivative **52**. NBS = *N*-bromosuccinimide; THF = tetrahydrofuran; DMF = *N*,*N*-dimethylformamide; MW = microwave irradiation; AQ10 = *N*-(quinolin-4-yl)octane-1,8-diamine.

To examine the significance of the cyano group for antiplasmodial potency, the terminal acetylenic derivative **52** was prepared (Scheme 4). 2-Bromothiophene (**33**) was coupled with 4-formylphenylboronic acid to afford **47**. Brominated product **48** obtained by reaction of **47** with NBS in THF was further submitted to Suzuki coupling with the 4-bromophenylboronic acid to afford the bromo aldehyde **49**, which was converted in the next step to **50** by Sonogashira coupling with ethynyltrimethylsilane applying microwave irradiation. The final acetylene derivative **52** was obtained after reductive amination of **50** that was followed by removal of trimethylsilyl group under basic conditions.

All tested compounds were fully characterized and their purity was >95% (as determined by High performance liquid chromatography (HPLC)). Full details are given in the Supplementary Materials.

### 2.2. In Vitro Antiplasmodial Activity

The antimalarial candidates were tested in vitro for their antiplasmodial activity against three *P. falciparum* strains: D6 (CQ susceptible (CQS) strain), W2 (CQ resistant (CQR) strain), and TM91C235 (Thailand, a multidrug-resistant (MDR) strain), using the Malaria SYBR Green Fluorescence Assay, a microtiter drug sensitivity assay that uses the intercalation of SYBR Green into malaria DNA as a measure of blood stage *P. falciparum* parasite proliferation in the presence of antimalarial compounds. This assay is performed as a dose response (12 two-fold serial dilutions) to obtain a calculated IC_50_ determination [30]. CQ and mefloquine (MFQ) were used as positive controls (Table 1 and Table 2).

As a general remark, to our content, all synthesized compounds were more active than CQ against the CQR W2 strain. Importantly, compounds **29**, **30** and **31** were as active as CQ against the CQS D6 strain (and had higher activity against W2 and C235 strains), while **28** was two times more active than CQ against the D6 strain. Comparison of in vitro antiplasmodial activities of fluoro and cyano analogues **26** with **29**, and **24** with **30**, clearly indicate that both cyano derivatives show significantly higher activities against CQS D6 strain. To analyze the effect of the cyano group in different positions of the benzothiophene core, we synthesized the pair of homologs **28**, **30**, and **31**, **32**, respectively. The compounds with cyano group at C(5) position were more potent than their C(6) isomers; it was also noticed that the shorter linker *n* = 2, instead of *n* = 4, was more favorable for antiplasmodial activity against D6 strain (**28** vs. **30**, **31** vs. **32**).

Unlike benzothiophenes, the compounds of the thiophene series were found to generally exhibit higher in vitro activity against CQR W2 than against CQS D6 strain (Table 2). We found it indicative that all derivatives in this series bearing cyano group were more active against MDR C235 strain in comparison to CQ, among which **41**, **42**, **44**, and **46** showed higher potency than MFQ as well. In addition, all tested C(7) chloro derivatives were more active than the respective des-chloro aminoquinolines against CQR W2 strain, e.g., **41** vs. **44** (13 nM vs. 74 nM). The effect of the cyano group of this series was additionally confirmed by comparison of its antimalarial activity to the activity of the respective acetylene analogue **52**, suggesting that cyano substituent may play an important role in the antiplasmodial activity of these derivatives.

## 3. Discussion

To investigate possible toxicity issues, the synthesized compounds were evaluated in vitro against a human hepatoma cell line (HepG2). The range of their activity falls within 1976–30605 nM, significantly higher in comparison to their respective antimalarial activities (Table 1 and Table 2).

Among all examined derivatives, the compounds **25**, **42**, and **46** have been chosen for in vivo evaluation since they showed significant activities against W2 clone in vitro (Table 1 and Table 2, RI values), which is in sharp contrast to CQ. In a separate host toxicity study, benzothiophene **25** was subjected to in vivo toxicity evaluation. At the 160 mg/kg/day × 3 days dose, **25** proved to be non-toxic, as all 5 mice survived 30 days after administration and showed normal appearance and behavior. Yet, using a modified Thompson test model [31], administering **25** at the same concentration of 160 mg/kg/day × 3 days to C57Bl6 female mice infected with 1 × 10^6^ *P. berghei* parasites, unfortunately led to death of 3 out of 4 mice, with only one surviving 31 days but with parasitemia. In this test model, *P. berghei* infected mice were treated with aminoquinolines suspended in 0.5% hydroxyethylcellulose-0.1% Tween 80 and administered per os (p.o.) once per day on days 3–5 postinfection (Table 3).

Regarding thiophene derivatives, they were all well tolerated by Hep G2 cells, possessing IC_50_ > 2000 nM. However, rather low selectivity indices were calculated for all derivatives (SI_HepG2/D6_ = 30–624) with an exception of derivative **43**, SI _HepG2/D6_ > 2000. In a host toxicity study at the concentration of 160 mg/kg × 3 days, compounds **42** and **46** with the longest methylene linker (eight methylene groups), proved to be non-toxic (all 5 mice survived). The compounds were further subjected to in vivo evaluation, using the modified Thompson test model, at 160 mg/kg/day × 3 days for **42**, and at 80 and 40 mg/kg/day × 4 days for **46**. Under applied treatment conditions, three sets of 5 infected mice died of malaria. Derivative **46** enabled one mouse survival until day 25 (D25) at both administered doses, while compound **42** provided survival with high level of parasitemia until day 24 (D24) at a higher dose (Table 3).

**Figure 2:**
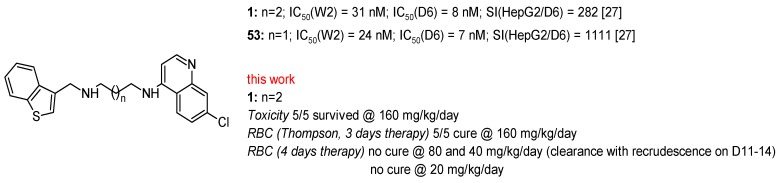
Antimalarial activity of compounds **1** and **53**. RBC—red blood cell.

**Figure 3:**
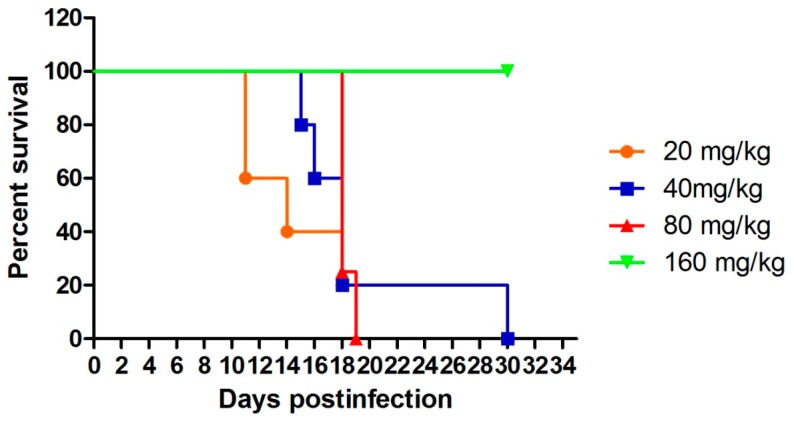
Effect of treatment with compound **1** at four dosage regimens (20, 40, 80, 160 mg/kg) on the survival of mice infected with *P. berghei* Antwerpen-Kasapa (ANKA) strain.

It is important to be aware that direct correlation of in vitro and in vivo results is not always justified. One of many examples is our previously reported compound **1** [27] (Figure 2), which was examined for its antimalarial activity in vivo, despite a relatively low SI (282). Surprisingly, compound **1** cured 5/5 mice using a modified Thompson test (Figure 3, Table 3) and cleared all parasites on day 7 (D7) (parasitemia before treatment was 0.3%–0.9%, and at the end of the study all 5 mice were parasite negative). The same compound was assessed in a dose response test in which *P. berghei* infected mice were treated per os (p.o.) for 4 days, starting with day 3 postinfection. Unfortunately, at all three applied doses (80, 40 and 20 mg/kg/day) benzothiophene **1** did not clear the parasites (Table 3). There was no detectable parasitemia up to day 11 at a dose as low as 40 mg/kg/day**;** however, recrudescence occurred between day 11 (D11) and day 14 (D14). In vivo activity results for **1** are summarized on Figure 3. On the other hand, compound **53** [27] with a propylene linker, which showed much more promising SI index (1111), proved to be toxic at concentration of 160 mg/kg/day, thus it was not evaluated in vivo (Figure 2).

**Figure 4:**
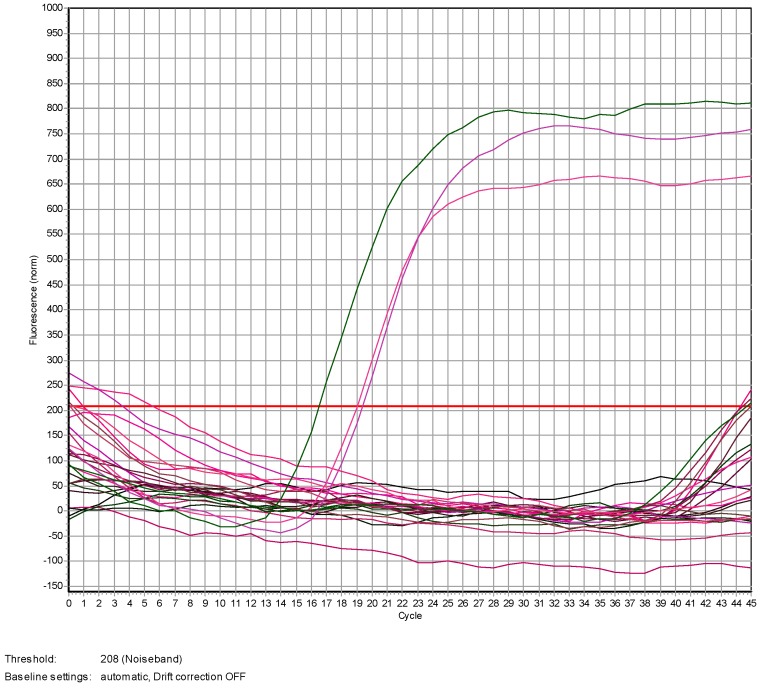
Real time (quantitative) PCR (qPCR) analysis for compound **1.** Amplification curves for blood and tissues of five mice. Horizontal line represents threshold for amplification. All values >40 Ct and amplification curves below threshold indicate absence of target DNA. The three amplification curves above threshold correspond to positive controls.

Parasite clearance provided by compound **1** with a 160 mg/kg/day × 3 days dose regimen, was further examined at the molecular level by polymerase chain reaction (PCR) [32]. Briefly, genomic DNA was extracted from the blood and liver of experimental animals using the DNeasy blood and tissue kit (Qiagen, Hilden, Germany) according to the manufacturer’s instructions. The primers and corresponding TaqMan (Applied BioSystems. Foster City, CA, USA) probe amplify and detect a highly conserved region of the 18S rRNA gene of the genus *Plasmodium*. Pure *P. berghei* gDNA samples were used as positive controls. Both blood and tissue samples of all 5 mice proved to be negative for *P. berghei* DNA (Figure 4).

The possible mechanism of action of compound **1** could include inhibition of β-hematin formation, since this compound showed a low IC_50_ value of 0.34 [33], which is 3.7 times lower than for CQ.

## 4. Materials and Methods

### 4.1. Chemistry

#### 4.1.1. General Experimental Procedures

Melting points were determined on a Boetius PHMK apparatus (Carl Zeiss, Germany) and were not corrected. IR spectra were recorded on a Nicolet 6700 Fourier-transformed infrared (FT-IR) diamond crystal spectrophotometer (Thermo-Scientific, Waltham, MA, USA). ^1^H- and ^13^C-NMR spectra were recorded on a Ultrashield Advance III spectrometer (at 500 and 125 MHz, respectively, Bruker, Billerica, MA, USA) and Gemini-200 spectrometer (at 50 MHz for ^13^C-NMR spectra, Varian, Palo Alto, CA, USA), in the indicated solvents (vide infra) using tetramethylsilane as the internal standard. Chemical shifts are expressed in ppm (δ) values and coupling constants (*J*) in Hz. High-resolution electrospray ionization mass spectrometry (ESI–MS, HRMS) spectra of the synthesized compounds were acquired on a 1200 Series instrument (Agilent Technologies, Santa Clara, CA, USA) equipped with a Zorbax Eclipse Plus C18 (100 × 2.1 mm i.d. 1.8 μm) column and diode array detector (DAD, 190–450 nm) in combination with a 6210 Time-of-Flight LC/MS instrument in positive ion mode. The samples were dissolved in MeOH (HPLC grade). The selected values were as follows: capillary voltage 4 kV; gas temperature 350 °C; drying gas 12 L·min^-1^; nebulizer pressure 45 psig; fragmentator voltage 70 V. Gas chromatography tandem mass spectrometry (GC-MS) analyses were performed on an Agilent 7890A GC (Agilent) system equipped with a 5975C inert XL EI/CI MSD and a flame ionization detector (FID) connected by capillary flow technology through a 2-way splitter with make-up gas. An HP-5 MS capillary column (Agilent Technologies, 25 mm i.d., 30 m length, 0.25 μm film thickness) was used. The flash chromatography was performed on a Biotage SP1 system (Biotage AB, Uppsala, Sweden) equipped with UV detector and FLASH 12+, FLASH 25+ or FLASH 40+ columns charged with KP-SIL (40–63 µm, pore diameter 60 Å), KP-C18-HS (40–63 µm, pore diameter 90 Å) or KP-NH (40–63 µm, pore diameter 100 Å) as an adsorbent.

#### 4.1.2. Method A—General Procedure for Reductive Amination for Compounds **8**, **9**, **12**, **13**, **23**–**26**, **28**–**32**, **37**–**45**, **51**

Amine (1.5 eq.) and appropriate aldehyde (1 eq.) were dissolved in MeOH/CH_2_Cl_2_ mixture (2:1 *v*:*v*), AcOH glac (1.5 eq.) was added, and the mixture was stirred under an Ar atmosphere at r.t. After 3 h, NaBH_4_ (6 eq.) was added, and stirring was continued for another 18 h. Solvent was removed under reduced pressure, and the residue was dissolved in CH_2_Cl_2_. The organic layer was washed with 2M NH_4_OH, water and then extracted with CH_2_Cl_2_. The combined organic layers were washed with brine and dried over anhydrous Na_2_SO_4_. Finally, the solvent was evaporated under reduced pressure.

#### 4.1.3. Method B—General Experimental Procedure for Bromination for Compounds **17**–**19**, **35**

Substituted benzothiophene (1 eq.) was dissolved in 1,2-dichloroethane. A solution of bromine (1.12 eq.) in 1,2-dichloroethane (CH_2_Cl_2_ for **17**) was slowly added to the reaction mixture at 0 °C, then warmed to r.t., and stirred for 2 h. Reaction progress was monitored by thin-layer chromatography (TLC, reverse phase silica gel, MeOH). Aqueous Na_2_S_2_O_3_ solution was added, and the desired product was extracted with CH_2_Cl_2_. Combined organic layers were washed with brine, and dried over anhydrous Na_2_SO_4_. After filtration, the solvent was removed under reduced pressure. The product was purified using column chromatography. [34]

#### 4.1.4. Method C—General Procedure for Suzuki Coupling for Compounds **20**–**22**

A suspension of Pd(OAc)_2_ (5.00 mol %) and SPhos (20.0 mol %) in DME was purged with Ar and stirred at r.t. for 10 mins. Brominated benzothiophene (1 eq.) in DME and 2 M Na_2_CO_3_ were then added. After 5 min, 4-formylphenylboronic acid (1.2 eq.) in EtOH was added. The mixture is once more purged with Ar and heated in a sealed vessel in microwave reactor at 100 °C for 2 h. Reaction mixture was filtered through Celite and transferred to a separation funnel. Water was added and extracted with CH_2_Cl_2_. Combined organic layers were washed with brine and dried over anhydrous Na_2_SO_4_. After filtration, the solvent was removed under reduced pressure. The product was purified using column chromatography.

#### 4.1.5. Method D—General Procedure for Synthesis of Aminoquinolines **AQ88**, **AQ9** and **AQ10**

A mixture of 4-chloroquinoline or 4,7-dichloroquinoline (1 eq.) and an appropriate diamine (5 eq.) were subjected to microwave irradiation using an Initiator 2.5 apparatus (Biotage) for 15 mins at 80 °C, followed by 30 mins at 95 °C and then 2 h at 140 °C. After cooling to room temperature 0.1 M aqueous NaOH was added and then extracted with CH_2_Cl_2_. Combined organic layers were dried over anhydrous Na_2_SO_4_. After filtration, the solvent was removed under reduced pressure. The crude product was subjected to silica-gel column chromatography using CH_2_Cl_2_/MeOH (NH_3_ satd.) as eluent to afford the final compound.

#### 4.1.6. Method E—General Procedure for *N*-Methylation of Aminoquinolines for Compounds **27** and **46**

To a stirred solution of aminoquinolines (1 eq.) in MeOH containing 37% aqueous formaldehyde (2 eq.) was added mixture of ZnCl_2_ (2 eq.) and NaHB_3_CN (4 eq.) in MeOH. After the reaction mixture was stirred at r.t. for 4 h, the solution was taken up in 0.1 M NaOH and most of the MeOH was evaporated under reduced pressure. Aqueous solution was extracted with CH_2_Cl_2_, the combined extracts were washed with water and brine and dried over anhydrous Na_2_SO_4_. The solvent was evaporated under reduced pressure. [35] 

#### 4.1.7. Method F—General Procedure for the Suzuki Coupling Reaction Using PdO × 1.4 H_2_O for Compounds **34**, **36**

An appropriate aryl-bromide (1 eq.) was added to the mixture of arylboronic acid (1.2 eq.), catalyst PdO × 1.4 H_2_O (0.01–0.05 eq.), K_2_CO_3_ (1.2 eq.) and ethanol/H_2_O (3:1 *v*/*v*). The mixture was stirred at 60 °C for 5 h, then diluted with water and extracted with CH_2_Cl_2_. The combined organic layers were washed with brine and dried over anhydrous Na_2_SO_4_. After filtration, the solvent was removed under reduced pressure. The product was purified using silica gel flash chromatography [29].

#### 4.1.8. Method G—General Procedure for the Suzuki Coupling in MeOH/Toluene for Compounds **47**, **49**

A solution of Pd(OAc)_2_ (0.05 eq.) and PPh_3_ (0.2 eq.) in toluene was stirred at r.t. under an argon atmosphere for 10 mins. After that, the solution of an appropriate aryl-bromide (1 eq.), an appropriate arylboronic acid (1.1 eq.) and 2M aq. Na_2_CO_3_ (2 eq.) in MeOH and toluene was added. The mixture was stirred at 110 °C under an argon atmosphere for 3 h. The reaction work up method is provided for each compound.

*N*-(7-Chloroquinolin-4-yl)ethane-1,2-diamine (**AQ2**), *N*-(7-chloroquinolin-4-yl)propane-1,3-diamine (**AQ3**), *N*-(7-chloroquinolin-4-yl)butane-1,4-diamine (**AQ4**), *N*-(7-chloroquinolin-4-yl)pentane-1,5-diamine (**AQ5**), *N*-(7-chloroquinolin-4-yl)hexane-1,6-diamine (**AQ6**), *N*-(quinolin-4-yl)propane-1,3-diamine (**AQ7**), *N*-(quinolin-4-yl)butane-1,4-diamine (**AQ8**), were prepared according to known procedures [36,37,38,39].

Full details are given in the Supplementary Materials.

### 4.2. In Vitro Antiplasmodial Activity

Synthesized aminoquinolines were screened in vitro against *P. falciparum* strains: CQ and MFQ susceptible strain D6 (clone of Sierra Leone/UNC isolate), CQ resistant but MFQ susceptible strain W2 (clone of Indochina isolate), and CQ and MFQ resistant strain TM91C235 (clone of South East Asian isolate) using the Malaria SYBR Green Fluorescence Assay. Full method details are given in Supplementary Materials I. Assessment of compound toxicity in a HepG2 (hepatocellular carcinoma) cell line followed the protocol described in Ref. [40].

### 4.3. In Vivo Antiplasmodial Activity and Toxicity

The *P. berghei* (ANKA) mouse efficacy tests were conducted using a modified version of the Thompson test. Groups of five mice were inoculated intraperitoneally with erythrocytes infected with *P. berghei* on day 0. Drugs were suspended in 0.5% hydroxyethylcellulose-0.1% Tween 80 and administered orally once a day beginning on day 3 post infection. Dosings are given in Table 3. All untreated infected (control) mice showed parasitemia on day 3, which reached levels between 11.2% and 30% on day 6 and succumbed to the infection on day 6–8. Therefore, the test was considered valid. Cure was defined as survival (with no parasitemia) until day 31 post-treatment. Parasitemia was determined by thin-blood Giemsa-stained smears prepared from mice tail blood of each animal on days 0, 3, 6, 10, 13, 17, 20, 24, 27, and 31 (postinfection). The slides were examined under a light microscope.

In a separate host toxicity study, groups of five healthy mice were administered 160 mg/kg/day × 3 days of the investigational compounds and individually monitored for behavior and appearance two times a day for 31 days. No toxicity was defined as survival past day 31 with no overt clinical manifestations of toxicity (changes in behavior and appearance).

The study followed the International Guiding Principles for biomedical research involving animals, and was reviewed by a local Ethics Committee and approved by the Veterinary Directorate at the Ministry of Agricultutre and Environmental Protection of Serbia (decision no. 323-07-02444/2014-05/1).

### 4.4. Genomic DNA Extraction and qPCR Analysis

#### 4.4.1. DNA Extraction

Genomic DNA was extracted from the blood and liver of experimental animals using the DNeasy blood and tissue kit (Qiagen, Hilden, Germany) according to the manufacturer’s instructions. Briefly, mice alive past day 31 with complete parasite clearance were sacrificed. Next, between 300 and 500 μL of blood was removed after heart puncture and the organs were removed, rinsed with dPBS and homogenized. The liver was homogenized using mechanical disruption and then subjected to proteinase K digestion. Approximately 100 μL of sample was used per each spin column for gDNA extraction.

#### 4.4.2. Real Time PCR 

Briefly, the primers and corresponding TaqMan probe amplify and detect a highly conserved region of the 18S rRNA gene of the genus *Plasmodium*. The primer and probe sequences were as follows: forward primer Plasmo 1: 5-GTTAAGGGAGTGAAGACGA TCAGA-3; reverse primer Plasmo 2: 5-AACCCAAAGACTTTGATTTC TCATAA-3; TaqMan probe Plasprobe: 5-*FAM*-ACCGTCGTAA TCTTAACCAT AAACTATGCC GACTAG-*TAMRA*-3. Each 20μL reaction contained 1× Maxima Probe qPCR Master Mix (Thermo Fisher Scientific, Waltham, MA, USA), 200 nM of each primer, 50 nM probe, 1U UNG (Thermo Fisher Scientific) and 3 μL template gDNA. The following PCR conditions were used: one holding step at 50 °C for 2 min, one holding step at 95 °C for 10 min, then 45 cycles of 95 °C for 15 s, 60 °C for 1 min. The PCR was performed in a StepOne Plus instrument (Applied BioSystems). Samples starting with Ct40 were considered negative. A positive (*P. berghei* gDNA) and negative control (H_2_O) were included in each run [32].

## 5. Conclusions

Benzothiophenes presented in this paper, in comparison to compounds discussed in [26] showed improved activities against the CQS strain, with potencies against D6 of IC_50_ = 6 nM, and cured 5/5 *P. berghei* infected mice dosed per os (p.o.) at 160 mg/kg/day × 3 days. However, comparing the two series of benzothiophenes (and thiophenes), with and without the phenyl linker, we found that introducing the phenyl linker did not improve the in vitro activity of the first series of aminoquinolines [27] In both benzothiophene series, all compounds were more active against the CQS strain D6, than against strains CQR W2 and MDR TM91C235, with the sole exception of **25** which was more active against the W2 strain. As for thiophene series—**37**–**42**, **44** and **46**—a very interesting feature was revealed: hypersensitivity to the resistant strains, with a resistance index of less than 1. This is in sharp contrast to chloroquine, thus indicating that further development of the series could provide us with antimalarials more powerful against CQR strains.

## Figures and Tables

**Table 1:** In vitro antiplasmodial activity of benzothiophene derivatives.

Structure		In Vitro Antimalarial Activity (*P. falciparum*, IC_50_, nM) ^a,b^			HepG2 ^f^ (IC_50_, nM)	SI ^g^ HepG2/D6	RI ^h^ W2/D6
		D6 ^c^	W2 ^d^	C235 ^e^			
**8**	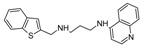	20	104	107	8867	440	5.2
**9**	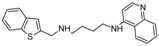	14	191	205	4867	339	13.6
**12**	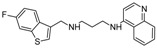	21	185	191	4477	211	8.8
**13**	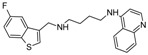	16	201	177	2726	172	12.6
**23**	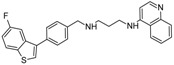	28	144	111	3825	136	5.1
**24**	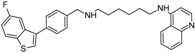	60	277	242	2233	37	4.6
**25**	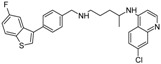	71	12	121	2087	29	0.2
**26**	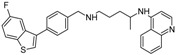	36	55	45	2345	65	1.5
**27**	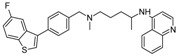	29	126	126	4998	173	4.3
**28**	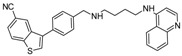	6	188	129	2575	401	31.3
**29**	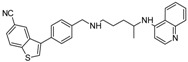	13	44	46	2413	192	3.4
**30**	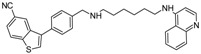	12	81	51	1976	162	6.8
**31**	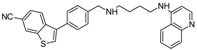	13	197	93	3577	276	15.2
**32**	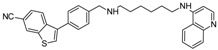	55	102	29	2049	37	1.8
**CQ ^i^**	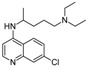	15 (6)	595 (5)	206 (5)			39.7
**MFQ ^i^**	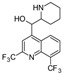	23 (6)	7 (5)	55 (5)			0.3

^a^ Antiplasmodial IC_50_ values (nM) (Malaria SYBR Green Fluorescence Assay) for isolates and clones of *P. falciparum*; ^b^ All in vitro experiments were done as technical quadruplicates, mean values are given; ^c^ Chloroquine (CQ) susceptible *P. falciparum* African D6 clone; ^d^ Chloroquine (CQ) resistant *P. falciparum* Indochina W2 clone; ^e^ *P. falciparum* CQ, MFQ and pyrimethamine resistant TM91C235 strain (Thailand); ^f^ Hepatocellular carcinoma; ^g^ Selectivity index; ^h^ Resistance index; ^i^ Control drugs: CQ as diphosphate; MFQ as HCl salt (number of replicates given in parentheses).

**Table 2:** In vitro antiplasmodial activity of thiophene derivatives.

Structure		In Vitro Antimalarial Activity (*P. falciparum*, IC_50_, nM) ^a,b^			HepG2 ^f^ (IC_50_, nM)	SI ^g^ HepG2/D6	RI ^h^ W2/D6
		D6 ^c^	W2 ^d^	C235 ^e^			
**37**	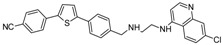	49	26	60	> 30605	624	0.5
**38**	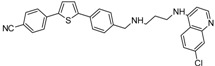	84	20	43	2564	30	0.2
**39**	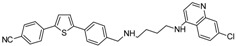	59	46	52	> 5792	98	0.8
**40**	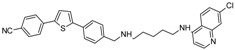	80	41	54	6702	84	0.5
**41**	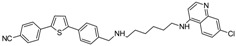	27	13	20	2415	89	0.5
**42**	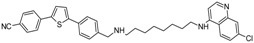	55	12	30	2092	38	0.2
**43**	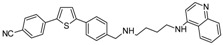	4	106	34	8153	2038	26
**44**	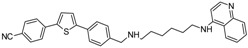	101	74	54	4085	40	0.7
**46**	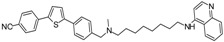	7	5	13	2570	367	0.7
**52**	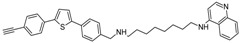	177	259	274	9230	52	1.5
**CQ ^i^**	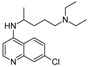	15 (6)	595 (5)	206 (5)			39.7
**MFQ ^i^**	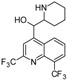	23 (6)	7 (5)	55 (5)			0.3

^a^ Antiplasmodial IC_50_ values (nM) (Malaria SYBR Green Fluorescence Assay) for isolates and clones of *P. falciparum*; ^b^ All in vitro experiments were done as technical quadruplicates, mean values are given; ^c^ CQ susceptible *P. falciparum* African D6 clone; ^d^ CQ resistant *P. falciparum* Indochina W2 clone; ^e^ *P. falciparum* multidrug resistant TM91C235 strain (Thailand). ^f^ Hepatocellular carcinoma; ^g^ Selectivity index; ^h^ Resistance index; ^i^ Control drugs: CQ as diphosphate; MFQ as HCl salt (number of replicates given in parentheses).

**Table 3:** In Vivo Antimalarial Activity ^a,b^.

Comp.	mg/kg/Day	Parasitemia (Day: # Mice, %Parasitemia)	Mice Dead/Day Died	Mice Alive on Day 31	Mean Survival Time (MST, Days)
**1**	20 ^c^	D3: 5 mice positive, 0.6%–2.4%; D7: 2 mice negative, 3 mice positive, 0.1%–0.2%; D11: 3 mice positive, 0.8%–1.9%; D14: 2 mice positive, 1.6%–3.7%; D18: 1 mouse positive, 6%; D21: 1 mouse positive, 27%; D25: 1 mouse positive, 63%; D28: 1 mouse positive, 66%	2/11	0/5	16.8
			1/14		
			1/18		
			1/30		
	40 ^c^	D3: 5 mice positive, 0.4%–0.9%; D7: 5 mice negative; D11: 5 mice negative; D14: 5 mice positive, 0.6%–2.4%; D18: 1 mouse positive, 2.2%; D21: 1 mouse positive, 7.3%; D25: 1 mouse positive, 37%; D28: 1 mouse positive, 53%	1/15	0/5	19.4
			1/16		
			2/18		
			1/30		
	80 ^c^	D3: 4 mice positive, 0.7%–1.1%; D7: 4 mice negative; D11: 4 mice negative; D14: 4 mice positive, 0.1%–0.5%; D18: 1 mouse positive, 2.3%	3/18	0/4	18.2
			1/19		
	160	D3: 5 mice positive, 0.3%–0.9%; D7–D31: 5 mice negative		5/5	>31
**25**	160	D3: 4 mice positive, 0.4%–0.6%; D7: 4 mice positive, 0.2%–0.4%; D10: 4 mice positive, 0.3%–0.6%; D14: 4 mice positive, 0.5%–0.8%; D17: 2 mouse positive, 1.5%–3.7%; D21: 1 mouse positive, 2.7%; D24: 1 mouse positive, 52%; D28: 1 mouse positive, 72%; D31: 1 mouse positive, 79%	2/17	1/4	20.8
			1/18		
**42**	160	D3: 5 mice positive, 0.2%–0.6%; D7: 5 mice positive, 0.2%–10%; D10: 4 mice positive, 9.2%–15%; D14: 3 mice positive, 12.8%–47%; D17: 3 mice positive, 50%–60%; D21: 2 mice positive, 64%–70%; D24: 1 mouse positive, 75%	1/9	0/5	18.6
			1/13		
			1/20		
			1/23		
			1/28		
**46**	40 ^c^	D3: 5 mice positive, 0.6%–2.2%; D7: 1 mouse negative, 4 mice positive, 0.2%–0.4%; D10: 4 mice positive, 1.6%–4.9%; D14: 2 mice positive, 8.1%–12%; D18: 2 mice positive, 16%–20%; D21: 2 mice positive, 22%–41%; D25: 1 mouse positive, 43%	1/10	0/5	15.8
			2/11		
			1/22		
			1/25		
	80 ^c^	D3: 5 mice positive, 0.8%–2.3%; D7: 2 mice negative, 2 mice positive, 0.1%–0.2%; D10: 4 mice positive, 0.2%–6.6%; D14: 1 mouse positive, 4.7%; D18: 1 mouse positive, 12%; D21: 1 mouse positive, 35%; D25: 1 mouse positive, 41%	1/7	0/5	14.4
			1/12		
			1/13		
			1/14		
			1/26		
**CQ**	160	D3: 5 mice positive, 0.3%–0.8%; D6-D31: 5 mice negative		5/5	>31
**Infected controls**	0	All mice died on day 7–8			

^a^ Groups of five *P*. *berghei* (ANKA strain) infected mice were treated per os (p.o.) once per day on days 3–5 postinfection with aminoquinolines suspended in 0.5% hydroxyethylcellulose-0.1% Tween 80. Mice alive on day 31 with no parasites in a blood film are considered cured; ^b^ All compounds were tested in separate experiments (mice groups) for toxicity. None exerted any toxic effect when dosed orally at 160 mg/kg/day × 3 days; ^c^ Groups of five *P*. *berghei* (ANKA strain) infected mice were treated per os (p.o.) once per day on days on 3–6 postinfection with aminoquinolines suspended in 0.5% hydroxyethylcellulose-0.1% Tween 80. Mice alive on day 31 with no parasites in a blood film are considered cured.

## Supplementary Materials

Supplementary Material—I (Chemistry); Supplementary Material—II (NMR spectra of synthesized compounds, HPLC analyses for purity) are available online.

## References

[1] Soulard V., Bosson-Vanga H., Lorthiois A., Roucher C., Franetich J., Zanghi G., Bordessoulles M., Tefit M., Thellier M., Morosan S. (2015). *Plasmodium falciparum* full life cycle and *Plasmodium ovale* liver stages in humanized mice. Nat. Commun..

[2] World Health Organization World Malaria Report, 2015. http://www.who.int/malaria/publications/world-malaria-report-2015/report/en/.

[3] Ashley E.A., Dhorda M., Fairhurst R.M., Amaratunga C., Lim P., Suon S., Sreng S., Anderson J.M., Mao S., Sam B. (2014). Spread of Artemisinin Resistance in *Plasmodium falciparum* Malaria. N. Engl. J. Med..

[4] World Health Organization Overview of Malaria Treatment. http://www.who.int/malaria/areas/treatment/overview/en/.

[5] Dholakia N., Dhandhukia P., Roy N. (2015). Screening of potential targets in *Plasmodium falciparum* using stage-specific metabolic network analysis. Mol. Divers..

[6] Hastings I.M., Bray P.G., Ward S.A. (2002). Parasitology. A requiem for chloroquine. Science.

[7] Fidock D.A., Nomura T., Talley A.K., Cooper R.A., Dzekunov S.M., Ferdig M.T., Ursos L.M. B., Sidhu A.B.S., Naude B., Deitsch K.W. (2000). Mutations in the *P. falciparum* Digestive Vacuole Transmembrane Protein PfCRT and Evidence for Their Role in Chloroquine Resistance. Mol. Cell.

[8] Banerjee T., Sharma S.K., Kapoor N., Dwivedi V., Surolia N., Surolia A. (2011). Benzothiophene carboxamide derivatives as inhibitors of *Plasmodium falciparum* enoyl-ACP reductase. IUBMB Life.

[9] Chhibber M., Kumar G., Parasuraman P., Ramya T.N.C., Surolia N., Surolia A. (2006). Novel diphenyl ethers: Design, docking studies, synthesis and inhibition of enoyl ACP reductase of *Plasmodium falciparum* and *Escherichia coli*. Bioorg. Med. Chem..

[10] Sharma S.K., Parasuraman P., Kumar G., Surolia N., Surolia A. (2007). Green Tea Catechins Potentiate Triclosan Binding to Enoyl-ACP Reductase from *Plasmodium falciparum* (PfENR). J. Med. Chem..

[11] Carballeira N.M., Bwalya A.G., Itoe M.A., Andricopulo A.D., Cordero-Maldonado M.L., Kaiser M., Mota M.M., Crawford A.D., Guido R.V.C., Tasdemir D. (2014). 2-Octadecynoic acid as a dual life stage inhibitor of *Plasmodium* infections and plasmodial FAS-II enzymes. Bioorg. Med. Chem. Lett..

[12] Rackham M.D., Brannigan J.A., Moss D.K., Yu Z., Wilkinson A.J., Holder A.A., Tate E.W., Leatherbarrow R.J. (2013). Discovery of Novel and Ligand-Efficient Inhibitors of Plasmodium falciparum and Plasmodium vivax *N*-Myristoyltransferase. J. Med. Chem..

[13] Rackham M.D., Brannigan J.A., Rangachari K., Meister S., Wilkinson A.J., Holder A.A., Leatherbarrow R.J., Tate E.W. (2014). Design and Synthesis of High Affinity Inhibitors of *Plasmodium falciparum* and *Plasmodium vivax*
*N*-Myristoyltransferases Directed by Ligand Efficiency Dependent Lipophilicity (LELP). J. Med. Chem..

[14] Slavic K., Krishna S., Derbyshire E.T., Staines H.M. (2011). Plasmodial sugar transporters as anti-malarial drug targets and comparisons with other protozoa. Malar. J..

[15] Slavic K., Derbyshire E.T., Naftalin R.J., Krishna S., Staines H.M. (2009). Comparison of effects of green tea catechins on apicomplexan hexose transporters and mammalian orthologues. Mol. Biochem. Parasitol..

[16] Saliba K.J., Krishna S., Kirk K. (2004). Inhibition of hexose transport and abrogation of pH homeostasis in the intraerythrocytic malaria parasite by an *O*-3-hexose derivative. FEBS Lett..

[17] Witschel M.C., Rottmann M., Schwab A., Leartsakulpanich U., Chitnumsub P., Seet M., Tonazzi S., Schwertz G., Stelzer F., Mietzner T. (2015). Inhibitors of Plasmodial Serine Hydroxymethyltransferase (SHMT): Cocrystal Structures of Pyrazolopyrans with Potent Blood- and Liver-Stage Activities. J. Med. Chem..

[18] Agbor-Enoh S., Seudieu C., Davidson E., Dritschilo A., Jung M. (2009). Novel Inhibitor of *Plasmodium* Histone Deacetylase That Cures *P. berghei*-Infected Mice. Antimicrob. Agents Chemother..

[19] O’Neill P.M., Bray P.G., Hawley S.R., Ward S.A., Park B.K. (1998). 4-Aminoquinolines—Past, present, and future: a chemical perspective. Pharmacol. Ther..

[20] Kharb R., Bansal K. (2013). Perspectives on Antimicrobial Potential of Benzothiophene Derivatives. Res. J. Pharm. Biol. Chem. Sci..

[21] Anderson D.R., Meyers M.J., Kurumbail R.G., Caspers N., Poda G.I., Long S.A., Pierce B.S., Mahoney M.W., Mourey R.J. (2009). Benzothiophene inhibitors of MK2. Part 1: Structure–activity relationships, assessments of selectivity and cellular potency. Bioorg. Med. Chem. Lett..

[22] Anderson D.R., Meyers M.J., Kurumbail R.G., Caspers N., Poda G.I., Long S.A., Pierce B.S., Mahoney M.W., Mourey R.J., Parikh M.D. (2009). Benzothiophene inhibitors of MK2. Part 2: Improvements in kinase selectivity and cell potency. Bioorg. Med. Chem. Lett..

[23] Matsunaga N., Kaku T., Itoh F., Tanaka T., Hara T., Miki H., Iwasaki M., Aono T., Yamaoka M., Kusaka M. (2004). C_17,20_-lyase inhibitors I. Structure-based de novo design and SAR study of C_17,20_-lyase inhibitors. Bioorg. Med. Chem..

[24] Rey J., Hu H., Kyle F., Lai C., Buluwela L., Coombes R.C., Ortlund E.A., Ali S., Snyder J.P., Barrett A.G.M. (2012). Discovery of a New Class of Liver Receptor Homolog-1 (LRH-1) Antagonists: Virtual Screening, Synthesis and Biological Evaluation. ChemMedChem..

[25] Malamas M.S., Sredy J., Moxham C., Katz A., Xu W., McDevitt R., Adebayo F.O., Sawicki D.R., Seestaller L., Sullivan D. (2000). Novel Benzofuran and Benzothiophene Biphenyls as Inhibitors of Protein Tyrosine Phosphatase 1B with Antihyperglycemic Properties. J. Med. Chem..

[26] Pérez-Silanes S., Berrade L., García–Sánchez R.N., Mendoza A., Galiano S., Pérez-Solórzano B.M., Nogal-Ruiz J.J., Martínez-Fernández A.R., Aldana I., Monge A. (2009). New 1-Aryl-3-Substituted Propanol Derivatives as Antimalarial Agents. Molecules.

[27] Terzić N., Konstantinović J., Tot M., Burojević J., Djurković-Djaković O., Srbljanović J., Štajner T., Verbić T., Zlatović M., Machado M. (2016). Reinvestigating Old Pharmacophores: Are 4-Aminoquinolines and Tetraoxanes Potential Two-Stage Antimalarials?. J. Med. Chem..

[28] Opsenica I.M., Verbić T.Ž., Tot M., Sciotti R.J., Pybus B.S., Djurković-Djaković O., Slavić K., Šolaja B.A. (2015). Investigation into novel thiophene- and furan-based 4-amino-7-chloroquinolines afforded antimalarials that cure mice. Bioorg. Med. Chem..

[29] Amoroso F., Colussi S., Del Zotto A., Llorca J., Trovarelli A. (2011). PdO hydrate as an efficient and recyclable catalyst for the Suzuki–Miyaura reaction in water/ethanol at room temperature. Catal. Commun..

[30] Johnson J.D., Dennull R.A., Gerena L., Lopez-Sanchez M., Roncal N.E., Waters N.C. (2007). Assessment and Continued Validation of the Malaria SYBR Green I-Based Fluorescence Assay for Use in Malaria Drug Screening. Antimicrob. Agents Chemother..

[31] Opsenica I., Burnett J.C., Gussio R., Opsenica D., Todorović N., Lanteri C.A., Sciotti R.J., Gettayacamin M., Basilico N., Taramelli D. (2011). A Chemotype That Inhibits Three Unrelated Pathogenic Targets: The Botulinum Neurotoxin Serotype A Light Chain, *P. falciparum* Malaria, and the Ebola Filovirus. J. Med. Chem..

[32] Rougemont M., van Saanen M., Sahli R., Hinrikson H.P., Bille J., Jaton K. (2004). Detection of Four *Plasmodium* Species in Blood from Humans by 18S rRNA Gene Subunit-Based and Species-Specific Real-Time PCR Assays. J. Clin. Microbiol..

[33] Parapini S., Basilico N., Pasini E., Egan T.J., Olliaro P., Taramelli D., Monti D. (2000). Standardization of the physicochemical parameters to assess in vitro the beta-hematin inhibitory activity of antimalarial drugs. Exp. Parasitol..

[34] Brendle J.J., Outlaw A., Kumar A., Boykin D.W., Patrick D.A., Tidwell R.R., Werbovetz K.A. (2002). Antileishmanial Activities of Several Classes of Aromatic Dications. Antimicrob. Agents Chemother..

[35] Kim S., Oh C.H., Ko J.S., Ahn K.H., Kim Y.J. (1985). Zinc-modified cyanoborohydride as a selective reducing agent. J. Org. Chem..

[36] Musonda C.C., Gut J., Rosenthal P.J., Yardley V., Carvalho de Souza R.C., Chibale K. (2006). Application of multicomponent reactions to antimalarial drug discovery. Part 2: New antiplasmodial and antitrypanosomal 4-aminoquinoline γ- and δ-lactams via a ‘catch and release’ protocol. Bioorg. Med. Chem..

[37] Peck R.M., Preston R.K., Creech H.J. (1959). Nitrogen mustard analogs of antimalarial drugs. J. Am. Chem. Soc..

[38] Price C.C., Leonard N.J., Peel E.W., Reitsema R.H. (1946). Some 4-amino-7-chloroquinoline derivatives. J. Am. Chem. Soc..

[39] Singh C., Malik H., Puri S.K. (2004). Synthesis and antimalarial activity of a new series of trioxaquines. Bioorg. Med. Chem..

[40] Opsenica I.M., Tot M., Gomba L., Nuss J.E., Sciotti R.J., Bavari S., Burnett J.C., Šolaja B.A. (2013). 4‑Amino-7-chloroquinolines: Probing Ligand Efficiency Provides Botulinum Neurotoxin Serotype A Light Chain Inhibitors with Significant Antiprotozoal Activity. J. Med. Chem..

