# Supplementary material for: Antimalarials with Benzothiophene Moieties as Aminoquinoline Partners
Source: Molecules. 2017 Feb 24;22(3):343. doi: 10.3390/molecules22030343 (PMC6155332; doi:10.3390/molecules22030343)
Supplement: Supplementary file 1 [file molecules-22-00343-s001.zip › Supplementary_material_I_3Feb2017.pdf]

Supplementary Material – I

Antimalarials with benzothiophene moieties as aminoquinoline partners

Jelena Konstantinović[1], Milica Videnović[2], Jelena Srbljanović[3], Olgica Djurković-Djaković[3], Katarina Bogojević[1], Richard Sciotti[4] and Bogdan Šolaja[1,*]

[1] Faculty of Chemistry, University of Belgrade, Studentski trg 16, P.O. Box 51, 11158, Belgrade, Serbia; jelena_konstantinovic@chem.bg.ac.rs (J.K.), bogojevickatarina@gmail.com (K.B.), bsolaja@chem.bg.ac.rs (B.Š.)

[2] Innovation center of the Faculty of Chemistry, Studentski trg 12-16, 11158 Belgrade, Serbia; milica_videnovic @chem.bg.ac.rs (M.V.)

[3] Institute for Medical Research, University of Belgrade, Dr. Subotića 4, 11129 Belgrade, Serbia; jelena.srbljanovic@imi.bg.ac.rs (J.S.); olgicadj@imi.bg.ac.rs (O.DjDj.)

[4] Experimental Therapeutics Branch, Walter Reed Army Institute of Research, Silver Spring, Maryland 20910, United States; richard.j.sciotti.civ@mail.mil (R.S.)

[*] Correspondence: bsolaja@chem.bg.ac.rs; Tel.: +381-11-263-86-06

Table of contents

Chemistry S3

In vitro antiplasmodial activity S33

Chemistry

Compounds were analyzed for purity (HPLC) using a Agilent 1200 HPLC system equipped with Quat Pump (G1311B), Injector (G1329B) 1260 ALS, TCC 1260 (G1316A) and Detector 1260 DAD VL+ (G1315C). HPLC analysis for each compound was performed in two diverse systems. All compounds were ≥95% pure.

Method A: Zorbax Eclipse Plus C18 4.6 × 150 mm, 1.8μ, S.N. USWKY01594 was used as the stationary phase. Eluent was made of the following solvents: 0.2% formic acid in water (A) and methanol (B). The analysis were performed at the UV max of the compounds (at 330 nm for compounds **8**, **9**, **12**, **13**, **23**, **24**, **25**, **26**, **27**, **28**, **29**, **30**, **31**, **32**, **41**, **44** and **52**) to maximize selectivity. Compounds were dissolved in methanol, final concentrations were ~1 mg/mL. Flow rate was 0.5 mL/min.

Compounds **8**, **9**, **13**, **25**, and **26** were eluted using gradient protocol: 0-1 min 95%A, 1-6 min 95%A→ 5%A, 6-11 min 5%A, 11-14 min 5%A→ 95%A.

Compounds **12**, **27**, **28**, **29**, **30**, **31** and **32** were eluted using gradient protocol: 0-1 min 95%A, 1-6 min 95%A→ 5%A, 6-11 min 5%A, 11-14 min 5%A→ 95 %A, 14-15 min 95%A.

Compound **23** was eluted using gradient protocol: 0-1 min 95%A, 1-6 min 95%A→ 5%A, 6-11 min 5%A, 11-14 min 5%A→ 95 %A, 14-18 min 95%A.

Compound **24** was eluted using gradient protocol: 0-1 min 95%A, 1-6 min 95%A→ 5%A, 6-11 min 5%A, 11-14 min 5%A→ 95%A, 14-16 min 95%A.

Compound **41** was eluted using gradient protocol: 0-1 min 95%A, 1-2 min 95%A→ 5%A, 2-10 min 5%A, 10-11 min 5%A→ 95%A, 11-13 min 95%A.

Compound **44** was eluted using gradient protocol: 0-1.5 min 95%A, 1.5-5 min 95%A→ 5%A, 5-16 min 5%A, 16-18 min 5%A→ 95%A, 18-19 min 95% A.

Compound **52** was eluted using gradient protocol: 0-2 min 95%A, 2-6 min 95%A→ 5%A, 6-17 min 5%A, 17-19 min 5%A→ 95%A, 19-21 min 95% A.

Method B: Zorbax Eclipse Plus C18 4.6 x 150 mm, 1.8μ, S.N. USWKY01594 was used as the stationary phase. Eluent was made of the following solvents: 0.2% formic acid in water (A) and acetonitrile (B). The analysis were performed at the UV max of the compounds (at 330 nm for compounds **8, 9, 12, 13, 24**, **26**, **27**, **31, 32, 41, 44, 52** and at 254 nm for compounds **23**, **25, 28, 29** and **30**) to maximize selectivity. Compounds were dissolved in methanol, final concentrations were ~1 mg/mL. Flow rate was 0.5 mL/min.

Compounds **8**, **9**, **13, 23**, **24, 25** and **26** were eluted using gradient protocol: 0-1 min 95%A, 1-6 min 95%A→ 5%A, 6-11 min 5%A, 11-14 min 5%A→ 95 %A.

Compounds **12, 27**, **28**, **29**, **30**, **31** and **32** were eluted using gradient protocol: 0-1 min 95%A, 1-6 min 95%A→ 5%A, 6-11 min 5%A, 11-14 min 5%A→ 95 %A, 14-15 min 95%A.

Compound **41** was eluted using gradient protocol: 0-1 min 95%A, 1-1.5 min 95%A→ 5%A, 1.5-9 min 5%A, 9-10 min 5%A→ 95%A, 10-12 min 95%A.

Compound **44** was eluted using gradient protocol: 0-1.5 min 95%A, 1.5-5 min 95%A→ 5%A, 5-16 min 5%A, 16-18 min 5%A→ 95%A, 18-19 min 95% A.

Compound **52** was eluted using gradient protocol: 0-1.5 min 95%A, 1.5-5 min 95%A→ 5%A, 5-14 min 5%A, 14-15 min 5%A→ 95%A, 15-16 min 95% A.

Method C: Zorbax Eclipse Plus C18 2.1 x 100 mm, 1.8μ, S.N. USUXU04444 was used as the stationary phase. Eluent was made of the following solvents: 0.2% formic acid in water (A) and methanol (B). The analysis were performed at the UV max of the compounds (at 330 nm for compounds **38, 39, 40** and **43** to maximize selectivity. Compounds were dissolved in methanol, final concentrations were ~1 mg/mL. Flow rate was 0.2 mL/min.

Compounds **38, 39** and **40** were eluted using gradient protocol: 0-1 min 95%A, 1-1.5 min 95%A→ 5%A, 1.5-8 min 5%A, 8-10 min 5%A→ 95%A, 10-12 min 95%A.

Compound **43** was eluted using gradient protocol: 0-1 min 95%A, 1-1.5 min 95%A→ 5%A, 1.5-9 min 5%A, 9-10 min 5%A→ 95%A, 10-12 min 95%A.

Method D: Zorbax Eclipse Plus C18 2.1 x 100 mm, 1.8μ, S.N. USUXU04444 was used as the stationary phase. Eluent was made of the following solvents: 0.2% formic acid in water (A) and acetonitrile (B). The analysis were performed at the UV max of the compounds (at 330 nm for compounds **27, 38, 39, 40** and **42**) to maximize selectivity. Compounds were dissolved in methanol, final concentrations were ~1 mg/mL. Flow rate was 0.2 mL/min.

Compounds **38, 39** and **40** were eluted using gradient protocol: 0-1 min 95%A, 1-1.5 min 95%A→ 5%A, 1.5-8 min 5%A, 8-10 min 5%A→ 95%A, 10-12 min 95%A.

Compound **42** was eluted using gradient protocol: 0-1 min 95%A, 1-8 min 95%A→ 5%A, 8-10 min 5%A, 10-12 min 5%A→ 95%A, 12-14 95%A.

Compound **43** was eluted using gradient protocol: 0-1 min 95%A, 1-1.5 min 95%A→ 5%A, 1.5-8 min 5%A, 8-10 min 5%A→ 95%A.

Method E: Zorbax Eclipse Plus C18 2.1 x 100 mm, 1.8μ, S.N. USUXU04444 was used as the stationary phase. Eluent was made of the following solvents: water (A) and acetonitrile (B). The analysis were performed at the UV max of the compound (at 330 nm for compound **37**)to maximize selectivity. The compound was dissolved in methanol, final concentration was ~1 mg/mL. Flow rate was 0.2 mL/min.

Compound **37** was eluted using gradient protocol: 0-1 min 95%A, 1-1.5 min 95%A→ 5%A, 1.5-9 min 5%A, 9-10 min 5%A→ 95%A, 10-12 min 95%A.

Method F: Zorbax Eclipse Plus C18 2.1 x 100 mm, 1.8μ, S.N. USUXU04444 was used as the stationary phase. Eluent was made of the following solvents: water (A) and methanol (B). The analysis were performed at the UV max of the compounds (at 330 nm for compound **37**) to maximize selectivity. The compound was dissolved in methanol, final concentration was ~1 mg/mL. Flow rate was 0.2 mL/min. Compound **37** was eluted using gradient protocol: 0-1 min 95%A, 1-1.5 min 95%A→ 5%A, 1.5-9 min 5%A, 9-10 min 5%A→ 95%A, 10-12 min 95%A.

Method G: Zorbax Eclipse Plus C18 4.6 x 150 mm, 1.8μ, S.N. USWKY01594 was used as the stationary phase. Eluent was made of the following solvents: water (A) and acetonitrile (B). The analysis were performed at the UV max of the compounds (at 330 nm for compound **42**) to maximize selectivity. The compound was dissolved in methanol, final concentration was ~1 mg/mL. Flow rate was 0.5 mL/min. Compound **42** was eluted using gradient protocol: 0-1.5 min 95%A, 1.5-5 min 95%A→ 5%A, 5-13 min 5%A, 13-14 min 5%A→ 95%A, 14-15 min 95% A.

Method H: Poroshell 120 EC-C18, 4.6 x 50mm, 2.7μ, S.N. USCFU07797 was used as the stationary phase. Eluent was made of the following solvents: 0.2% formic acid in water (A) and acetonitrile (B). Analyses were performed at the UV max of the compounds (330 nm for compound **46**) to maximize selectivity. The compound was dissolved in methanol, final concentration was ~1 mg/mL. Flow rate was 0.5 mL/min. Compound **46** was eluted using gradient protocol: 0-1 min 95%A, 1-3 min 95%A→ 5%A, 3-8 min 5%A, 8-10 min 5%A→ 95%A, 10-11 min 95%A.

Method I: Poroshell 120 EC-C18, 4.6 x 50mm, 2.7μ, S.N. USCFU07797 was used as the stationary phase. Eluent was made of the following solvents: 0.2% formic acid in water (A) and methanol (B). The analysis were performed at the UV max of the compounds (330 nm for compound **46**) to maximize selectivity. The compound was dissolved in methanol, final concentration was ~1 mg/mL. Flow rate was 0.5 ml/min. Compound **46** was eluted using gradient protocol: 0-1 min 95%A, 1-3 min 95%A→ 5%A, 3-8 min 5%A, 8-10 min 5%A→ 95%A, 10-11 min 95%A.

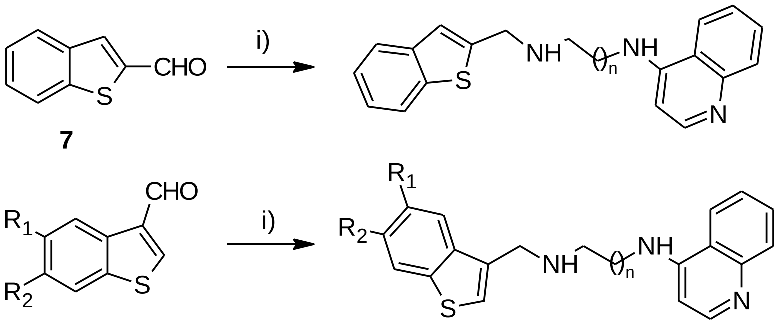

8: n=2 (59%)
9: n=3 (58%)

12: n=2, R_1=H, R_2=F (50%)
13: n=3, R_1=F, R_2=H (59%)

10: R_1=F, R_2=H[1]
11: R_1=H, R_2=F[1]

i) 1) aminoquinoline, AcOH glac, MeOH/CH_2Cl_2, r.t., 2 h 2) $NaBH_4$, r.t., 12 h

_N_-(1-benzothiophen-2-ylmethyl)-_N_'-(quinolin-4-yl)propane-1,3-diamine (8).

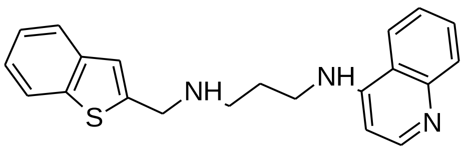

Compound **8** was prepared by method A, using 1-benzothiophene-2-carbaldehyde (94.0 mg, 0.580 mmol), **AQ7** [1] (175 mg, 0.870 mmol), glac. AcOH (50 µL, 0.8 mmol) and MeOH/CH_2Cl_2 (24 mL, 2:1, v/v). The product was purified using column chromatography (dry-flash, SiO_2, eluent EtOAc/hexane gradient 1/9 → EtOAc, EtOAc/MeOH gradient 95/5 → 1/1, flash, Biotage SP1, NH column, eluent EtOAc/hexane gradient 8/2 → EtOAc, EtOAc/MeOH gradient 95/5 → MeOH and flash, Biotage SP1, SiO_2 column, eluent EtOAc/MeOH+NH_3 (9/1) gradient 95/5 → 1/1). Final product **8** was obtained as a pale yellow oil (119 mg, 59%). IR (ATR): 3262w, 3059w, 2928w, 2838w, 1617w, 1585s, 1542m, 1458w, 1438w, 1396w, 1372w, 1339w, 1284w, 1243w, 1131w, 862w, 810w, 765m, 728w cm^{-1}. ^{1}H NMR (500MHz, $CDCl_3$, δ): 8.55-8.48 (m, H-C(2')), 7.98-7.91 (m, H-C(8')), 7.79 (d, *J* = 7.8, H-C(5')), 7.73-7.68 (m, H-C(7) and H-C(4)), 7.57-7.53 (m, H-C(7')), 7.36-7.28 (m, H-C(5) and H-C(6)), 7.20-7.15 (m, H-C(6') and H-C(3)), 6.96 (bs, H-N exchangeable with D_2O), 6.35-6.32 (m, H-C(3')), 4.12 (s, 2H, ArC*H*2-), 3.45-3.39 (m, 2H, ArNHC*H*2-), 2.96-2.92 (m, 2H, $ArCH_2NHCH_2$-), 2.15 (bs, H-N exchangeable with D_2O), 1.95-1.88 (m, 2H, $ArNHCH_2CH_2$-). ^{13}C NMR (50 MHz, $CDCl_3$, δ): 150.77, 150.36, 144.26, 139.65, 139.56, 129.25, 128.94, 124.32, 124.10, 123.19, 122.37, 121.84, 120.40, 118.91, 98.06, 49.27, 48.27, 43.12, 27.62. HRMS: *m*/*z* 348.15289 corresponds to molecular formula $C_{21}H_{21}N_3SH^+$ (error in ppm 0.07). HPLC purity (λ = 330 nm): method A: RT 10.408, area 96.80%; method B: RT 8.140, area 96.44%.

_N_-(1-benzothiophen-2-ylmethyl)-_N_'-(quinolin-4-yl)butane-1,4-diamine (9).

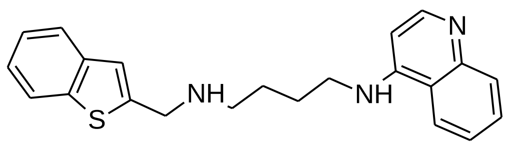

Compound **9** was prepared by method A, using 1-benzothiophene-2-carbaldehyde (120 mg, 0.74 mmol), **AQ8** [1] (240 mg, 1.1 mmol), glac. AcOH (63 μL, 1.1 mmol) and MeOH/CH_2Cl_2 (24 mL, 2:1, v/v). The product was purified using column chromatography (dry-flash, SiO_2, eluent EtOAc/hexane gradient 1/9 → EtOAc, EtOAc/MeOH gradient 95/5 → 1/9). Final product **9** was obtained as a pale yellow oil (160 mg, 58%). IR (ATR): 3649w, 3626w, 3436w, 3250m, 3117w, 3060m, 2929m, 2855m, 1617w, 1582s, 1541m, 1457m, 1438m, 1396w, 1374w, 1342m, 1280w, 1260w, 1225w, 1129w, 1065w, 1016w, 972w, 861w, 811w, 764m, 744m, 703w, 589w, 558w, 476w cm^{-1}. ^{1}H NMR (500MHz, $CDCl_3$, δ): 8.52 (d, *J* = 5.2, H-C(2')), 7.96 (d, *J* = 8.2, H-C(8′)), 7.79-7.73 (m, H-C(4) and H-C(7)), 7.68 (d, *J* = 7.6, H-C(5′)), 7.60-7.56 (m, H-C(7′)), 7.35-7.25 (m, H-C(5) and H-C(6) and H-C(6′)), 7.13 (s, H-C(3)), 6.37 (d, *J* = 5.2, H-C(3')), 5.62-5.55 (m, H-N exchangeable with D_2O), 4.08 (s, 2H, ArC*H*$_2$-), 3.34-3.27 (m, 2H, ArNHC*H*$_2$-), 2.75 (t, *J* = 6.8, 2H, ArCH$_2$NHC*H*$_2$-), 2.05-1.80 (m, 3H, ArNHCH$_2$C*H*$_2$- and H-N exchangeable with D_2O), 1.68 (quin, *J* = 6.8, 2H, ArNHCH$_2$CH$_2$C*H*$_2$-). ^{13}C NMR (125 MHz, $CDCl_3$, δ): 150.98, 149.78, 148.35, 145.07, 139.70, 139.53, 129.77, 128.86, 124.40, 124.17, 123.88, 123.08, 122.32, 121.27, 119.51, 118.76, 98.60, 49.07, 48.36, 43.09, 27.60, 26.30. HRMS: *m*/*z* 362.16771 corresponds to molecular formula $C_{22}H_{23}N_3SH^+$ (error in ppm -2.30). HPLC purity (λ = 330 nm): method A: RT 8.370, area 96.73%; method B: RT 8.293, area 97.83%.

***N*-[(6-fluoro-1-benzothiophen-3-yl)methyl]-*N*'-(quinolin-4-yl)propane-1,3-diamine (12).**

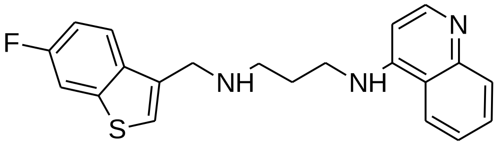

Compound **12** was prepared by method A, using aldehyde **11** (164.3 mg, 0.9117 mmol), **AQ7** [1] (275.3 mg, 1.368 mmol), glac. AcOH (78 μL, 1.4 mmol) and MeOH/CH_2Cl_2 (24 mL, 2:1, v/v). The product was purified using column chromatography (dry-flash, SiO_2, eluent EtOAc/hexane gradient 1/9 → EtOAc, EtOAc/MeOH gradient 95/5 → 4/6, flash, Biotage SP1, NH column, eluent EtOAc/hexane gradient 8/2 → EtOAc, EtOAc/MeOH gradient 95/5 → MeOH and flash, Biotage SP1, SiO_2 column, eluent EtOAc/MeOH+NH_3 (9/1) gradient 95/5 → 3/7). Final product **12** was obtained as a colorless oil (165 mg, 50%). IR (ATR): 3252m, 3072m, 2925m, 2850m, 2359w, 1583s, 1539m, 1466m, 1398w, 1372w, 1338w, 1283w, 1252w, 1210w, 1132w, 1051w, 895w, 851w, 811w, 765w, 736w cm^{-1}. ^{1}H NMR (500MHz, $CDCl_3$, δ): 8.52-8.49 (m, H-C(2')), 7.93 (d, *J* = 8.6, H-C(8')), 7.76-7.71 (m, H-C(4)), 7.58-7.48 (m, H-C(7)) and H-C(7') and H-C(5′)), 7.29 (s, H-C(2)), 7.15-7.10 (m, H-C(6′)), 7.08-6.99 (m, H-C(5) and H-N exchangeable with D_2O), 6.34-6.31 (m, H-C(3′)), 4.07 (s, 2H, ArC*H*$_2$-), 3.45-3.39 (m, 2H, ArNHC*H*$_2$-), 3.04-2.98 (m, 2H, ArCH$_2$NHC*H*$_2$-), 2.10-1.85 (m, 3H, ArCH$_2$NHCH$_2$C*H*$_2$- and H-N exchangeable with D_2O). ^{13}C NMR (125 MHz, $CDCl_3$, δ): 160.64 (d, *J* = 243.2),

150.82, 150.30, 148.09, 141.56 (d, *J* = 9.6), 134.75, 134.20, 129.41, 128.88, 124.24, 122.89 (d, *J* = 3.5), 122.54 (d, *J* = 8.8), 120.06, 118.87, 113.20 (d, *J* = 24.5), 108.97 (d, *J* = 25.4), 98.10, 49.17, 47.75, 43.49, 27.74. HRMS: *m/z* 366.14294 corresponds to molecular formula $C_{21}H_{20}FN_3SH^+$ (error in ppm -1.45). HPLC purity (λ = 330 nm): method A: RT 8.353, area 98.30%; method B: RT 7.413, area 95.19%.

_N_-[(5-fluoro-1-benzothiophen-3-yl)methyl]-_N'_-(quinolin-4-yl)butane-1,4-diamine (13).

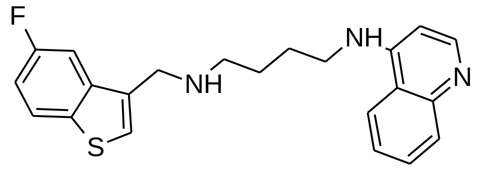

Compound **13** was prepared by method A, using aldehyde **10** (90.0 mg, 0.499 mmol), **AQ8** [1] (161.3 mg, 0.7492 mmol), glac. AcOH (43 μL, 0.75 mmol) and MeOH/CH_2Cl_2 (24 mL, 2:1, v/v). The product was purified using column chromatography (dry-flash, SiO_2, eluent EtOAc/hexane gradient 1/9 → EtOAc, EtOAc/MeOH gradient 95/5 → 8/2 and flash, Biotage SP1, NH column, eluent EtOAc/hexane gradient 8/2 → EtOAc, EtOAc/MeOH gradient 95/5 → MeOH). Final product **13** was obtained as pale yellow oil (112 mg, 59%). IR (ATR): 3649w, 3566w, 3255m, 3074m, 2933m, 2858m, 1582s, 1541m, 1438m, 1396w, 1374w, 1342w, 1249w, 1198w, 1130w, 1082w, 1035w, 914w, 855w, 808w, 765w, 736w, 647w cm^{-1}. ^{1}H NMR (500MHz, $CDCl_3$, δ): 8.53 (d, *J* = 5.2, H-C(2')), 7.98-7.93 (m, H-C(8’)), 7.76 (dd, J_1 = 4.8, J_2 = 8.7, H-C(7)), 7.70 (d, *J* = 8.2, H-C(5’)), 7.60-7.55 (m, H-C(7’)), 7.52-7.47 (m, H-C(4)), 7.37 (s, H-C(3)), 7.30-7.25 (m, H-C(6’)), 7.13-7.06 (m, H-C(6)), 6.37 (d, *J* = 5.2, H-C(3')), 5.55 (bs, H-N exchangeable with D_2O), 4.01-3.97 (m, 2H, ArCH_2-), 3.33-3.27 (m, 2H, ArNHCH_2-), 2.78 (t, *J* = 6.9, 2H, $ArCH_2NHCH_2$-), 1.89-1.81 (quin, *J* = 6.9, 2H, $ArNHCH_2CH_2$-), 1.80-1.66 (m, 3H, H-N exchangeable with D_2O and $ArNHCH_2CH_2CH_2$-). ^{13}C NMR (125 MHz, $CDCl_3$, δ): 160.70 (d, *J* = 240.1), 150.97, 149.75, 148.33, 139.49 (d, *J* = 9.0), 135.94, 134.89 (d, *J* = 4.5), 129.78, 128.87, 125.33, 124.38, 123.92 (d, *J* = 9.0), 119.40, 118.74, 113.20 (d, *J* = 25.3), 107.50 (d, *J* = 23.5), 98.60, 49.02, 47.67, 43.07, 27.68, 26.42. HRMS: *m/z* 190.58320 corresponds to molecular formula $C_{22}H_{22}N_3SFH_2^{2+}$ (error in ppm 0.00) *m/z* 380.15904 corresponds to molecular formula $C_{22}H_{22}N_3SFH^+$ (error in ppm -0.21). HPLC purity (λ = 330 nm): method A: RT 10.817, area 98.73%; method B: RT 8.234, area 97.72%.

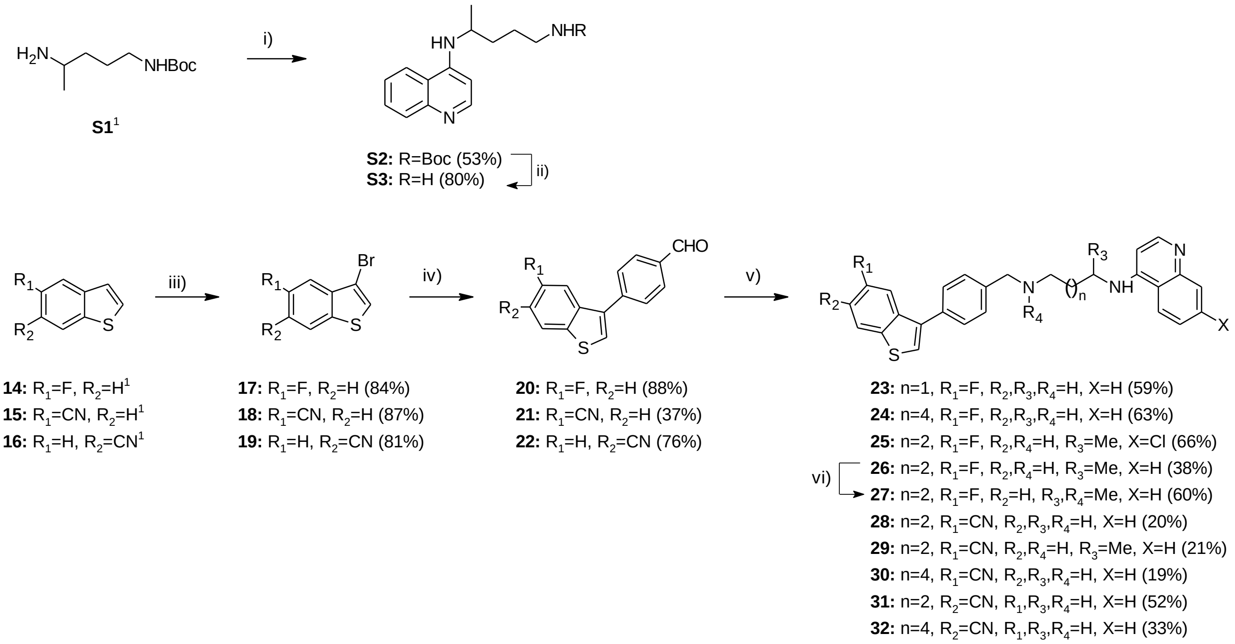

i) 4-chloroquinoline, 80°C, 1 h; 120-130 °C, 6-8 h; ii) 1) CF_3COOH/CH_2Cl_2, r.t., 24 h; 2) 2.5 M NaOH; iii) Br_2, $C_2H_4Cl_2$, 0 °C to r.t., 2 h; iv) 4-formylphenylboronic acid, $Pd(OAc)_2$, SPhos, DME, Na_2CO_3, EtOH, 100 °C, 2 h, MW; v) 1) aminoquinoline, AcOH glac, $MeOH/CH_2Cl_2$, r.t., 2 h 2) $NaBH_4$, r.t., 12 h; vi) HCHO, $ZnCl_2$, $NaBH_3CN$, MeOH, r.t., 4 h

***tert*-butyl [4-(quinolin-4-ylamino)pentyl]carbamate (S2).**

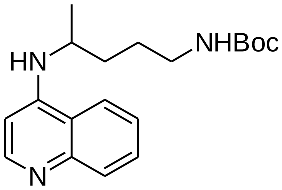

4-chloroquinoline (216 mg, 1.32 mmol) and amine *tert*-butyl (4-aminopentyl)carbamate **S1** [1] (401 mg, 1.98 mmol) were heated at 120-130 °C for 8 h with continued stirring as previously described in literature [2]. The reaction mixture was cooled to r.t. and taken up in CH_2Cl_2. The organic layer was washed with $NaHCO_3$, wather and brine. The organic layer was dried over anhydrous Na_2SO_4 and solvent was evaporated under reduced pressure. The product **S2** was purified using column chromatography (dry-flash, SiO_2, eluent hexane/EtOAc gradient 1/1 → EtOAc, EtOAc/MeOH gradient 9/1 → 7/3 and flash, Biotage SP, NH column, 25+M, eluent hexane, hexane/EtOAc gradient 7/3 → EtOAc, EtOAc/MeOH → MeOH). Final product **S2** was obtained as a yellow oil (230.5 mg, 53%). IR (ATR): 3430w, 3343m, 3237m, 3116w, 3059w, 2974m, 2933m, 2870w, 1691s, 1618w, 1580s, 1537s, 1453m, 1394m, 1367m, 1343m, 1258m, 1171m, 1045w, 1024w, 976w, 870w, 810w, 765m, 738m, 703w cm^{-1}. ^1H NMR (500 MHz, $CDCl_3$, δ): 8.53 (d, *J* = 5.4, H-C(2)), 7.99-7.95 (m, H-C(8)), 7.80-7.74 (m, H-C(5)), 7.64-7.60 (m, H-C(7)), 7.44-7.39 (m, H-C(6)), 6.42 (d, *J* = 5.4, H-C(3)), 5.04-4.94 (m, NH), 4.61 (bs, NH), 3.80-3.70 (m, 1H, ArNHC*H*-), 3.25-3.10 (m, 2H, $ArNHCH(CH_3)CH_2CH_2CH_2$-), 1.75-1.55 (m, 4H, $ArNHCH(CH_3)CH_2$- and $ArNHCH(CH_3)CH_2CH_2$-), 1.44 (s, 9H, $-NHCOOC(CH_3)_3$), 1.32 (d, *J* = 6.2, 3H, $ArNHCH(CH_3)$-). ^{13}C NMR

(125 MHz, $CDCl_3$, δ): 156.11, 151.00, 148.76, 148.64, 129.99, 128.91, 124.46, 119.29, 118.76, 98.88, 79.30, 48.12, 40.33, 33.52, 28.38, 27.03, 20.46. HRMS: *m/z* 330.21811 corresponds to molecular formula $C_{19}H_{27}N_3O_2H^+$ (error in ppm 1.54).

***N*⁴-(quinolin-4-yl)pentane-1,4-diamine (S3).**

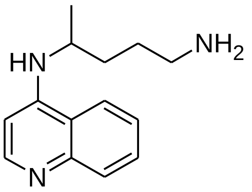

A solution of compound **S2** (210 mg, 0.64 mmol) in TFA/CH_2Cl_2 (5.5 mL, 1:10, v/v), was stirred at r.t. for 6 h. Solvents were evaporated under reduced pressure and the residue was treated with CH_2Cl_2/2.5M NaOH. The organic layer was dried over $MgSO_4$, and the solvent was evaporated under reduced pressure. Final product **S3** was obtained as a yellow oil (130 mg, 92%). IR (ATR): 3293m, 2972m, 2933m, 2872w, 1583s, 1547m, 1503w, 1447w, 1397w, 1373w, 1342w, 1282w, 1257w, 1226w, 1148w, 917w, 890w, 809w, 769m cm^{-1}. ^1H NMR (500 MHz, CD_3OD, δ): 8.32 (d, *J* = 5.5, H-C(2)), 8.17-8.13 (m, H-C(8)), 7.80-7.76 (m, H-C(5)), 7.63-7.57 (m, H-C(7)), 7.43-7.38 (m, H-C(6)), 6.50 (d, *J* = 6.0, H-C(3)), 3.82-3.74 (m, 1H, ArNHC*H*-), 2.65 (t, 2H, *J* = 7.0, ArCH(CH_3)CH_2CH_2C*H*₂-), 1.80-1.70 (m, 1H, ArNHCH(CH_3)C*H*₂-), 1.70-1.50 (m, 3H, ArNHCH(CH_3)C*H*₂- and ArNHCH(CH_3)CH_2C*H*₂-), 1.31 (d, 3H, *J* = 6.4, C*H*₃). ^{13}C NMR (125 MHz, CD_3OD, δ): 152.02, 151.25, 149.12, 130.39, 128.82, 125.43, 122.28, 120.33, 99.35, 49.42, 42.51, 34.67, 30.66, 20.43. HRMS: *m/z* 115.58609 corresponds to molecular formula $C_{14}H_{19}N_3H_2^{2+}$ (error in ppm -1.16).

3-Bromo-5-fluoro-1-benzothiophene (17).

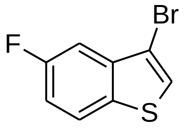

Compound **17** was prepared by method B, using 5-fluoro-1-benzothiophene [1] (250 mg, 1.6 mmol), Br_2 (91 μL, 1.8 mmol) and CH_2Cl_2 (6.3 mL + 6.3 mL). The product was purified using column chromatography (dry-flash, SiO_2, eluent hexane). Final product **17** was obtained as colorless crystals (320 mg, 84%). M.p. = 70 – 73 °C. IR (ATR): 3964w, 3856w, 3818w, 3795w, 3739w, 3641w, 3145w, 3099s, 3072m, 3021w, 2925w, 2889w, 2732w, 2684w, 2623w, 2561w, 2423w, 2378w, 2264w, 2214w, 2162w, 2103w, 2055w, 1945w, 1880w, 1834w, 1712w, 1665w, 1602s, 1564w, 1538w, 1494m, 1425s, 1326w, 1293m, 1244m, 1188s, 1126m, 1064w, 971m, 857s, 807w, 761w, 716w, 642w, 614w cm^{-1}. ^1H NMR (500MHz, $CDCl_3$, δ): 7.78 (dd, J_1 = 4.7, J_2 = 8.8, H-C(7)), 7.53-7.48 (m, H-C(4) and H-C (2)), 7.19-7.14 (m, H-C(6)). ^{13}C NMR (125 MHz, $CDCl_3$, δ): 161.36 (d, *J* = 241.2), 138.92 (d, *J* = 9.9), 133.92, 125.89, 124.01 (d, *J* = 9.5), 114.36 (d, *J* = 25.3), 108.87 (d, *J* = 24.4), 107.02 (d, *J* = 4.5). GC/MS (*m/z*, %): 231.9 ([M^+], 100), 229.9 (99), 151.0 (22), 107.0 (58).

3-Bromo-1-benzothiophene-5-carbonitrile (18).

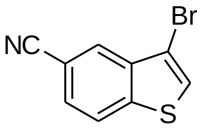

Compound **18** was prepared by method B, using 1-benzothiophene-5-carbonitrile [1] (100.0 mg, 0.6281 mmol), Br_2 (35 μL, 0.69 mmol) and DCE (2.5 mL + 2.5 mL). The product was purified using column chromatography (dry-flash, SiO_2, eluent hexane,

hexane/EtOAc gradient 97/3 → 9/1). Final product **18** was obtained as colorless crystals (130 mg, 87%). M.p. = 144 – 147 °C. IR (ATR): 3116s, 3074m, 2224s, 1768m, 1597m, 1572m, 1497m, 1431s, 1322m, 1293m, 1253m, 1154w, 1128w, 1058m, 966w, 887m, 808m cm^{-1}. ^{1}H NMR (500 MHz, $CDCl_3$, δ): 8.16-8.14 (m, H-C(7)), 7.93 (d, *J* = 8.2, H-C(4)), 7.64-7.61 (m, H-C(6)), 7.60 (s, H-C(2)). ^{13}C NMR (125 MHz, $CDCl_3$, δ): 142.68, 137.68, 127.76, 127.20, 126.11, 123.78, 118.82, 109.03, 107.72. GC/MS (*m*/*z*, %): 239.8 ([M$^+$], 100).

3-Bromo-1-benzothiophene-6-carbonitrile (19).

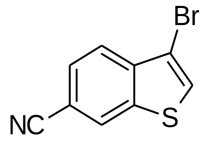

Compound **19** was prepared by method B, using compound 1-benzothiophene-6-carbonitrile [1] (1.05 g, 6.60 mmol), Br_2 (0.37 mL, 7.2 mmol) and DCE (26.3 mL + 26.3 mL). The product was purified using column chromatography (dry-flash, SiO_2, eluent hexane, hexane/EtOAc 97/3 → EtOAc). Final product **19** was obtained as colorless crystals (320 mg, 84%). M.p. = 140 – 144 °C (EtOAc). IR (ATR): 3950w, 3858w, 3821w, 3422m, 3145m, 3096s, 3070s, 3034m, 2979m, 2935m, 2873w, 2852m, 2805w, 2718w, 2659m, 2629w, 2582w, 2528w, 2492w, 2225s, 2176m, 2048w, 1903w, 1788m, 1770m, 1640m, 1598w, 1548m, 1513m, 1484w, 1455m, 1413s, 1392m, 1350w, 1322s, 1258s, 1195s, 1164m, 1135m, 1058w, 937s, 894m, 816s, 769s, 709m, 605m cm^{-1}. ^{1}H NMR (500 MHz, $CDCl_3$, δ): 8.19-8.18 (m, H-C(7)), 7.93-7.90 (m, H-C(4)), 7.71-7.68 (m, H-C(2) and H-C(5)). ^{13}C NMR (125 MHz, $CDCl_3$, δ): 140.35; 138.54; 128.16; 127.63; 127.38; 123.92; 118.68; 108.91; 108.04. GC/MS (*m*/*z*, %): 238.9 ([M$^+$], 100); 158.0 (16); 114.0 (36).

4-(5-Fluoro-1-benzothiophen-3-yl)benzaldehyde (20).

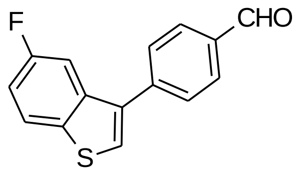

Compound **20** was prepared by method C, using compound **17** (241 mg, 1.04 mmol), 4-formylphenylboronic acid (195 mg, 1.30 mmol), $Pd(OAc)_2$ (11.6 mg, 0.0519 mmol), SPhos (85.3 mg, 0.208 mmol), 2M Na_2CO_3 (1.6 mL), DME (2 mL + 2 mL) and EtOH (2 mL). The product was purified using column chromatography (dry-flash, SiO_2, eluent hexane → hexane/EtOAc = 9/1). Final product **20** was obtained as white crystals (235 mg, 88%). M.p. = 125 – 127 °C. IR (ATR): 3094w, 2831w, 2736w, 1979w, 1702s, 1606s, 1568w, 1493w, 1439m, 1388w, 1306w, 1282w, 1255w, 1208m, 1171w, 1117w, 885w, 860w, 836w, 816w, 789w cm^{-1}. ^{1}H NMR (500MHz, $CDCl_3$, δ): 10.08 (s, CHO), 8.03-7.99 (m, 2H-Ar), 7.85 (dd, J_1 = 4.9, J_2 = 8.8, H-C(7)), 7.75-7.71 (m, 2H-Ar), 7.59 (s, H-C(2)), 7.58-7.54 (m, H-C(4)), 7.21-7.15 (m, H-C(6)). ^{13}C NMR (125 MHz, $CDCl_3$, δ): 191.69, 161.26 (d, *J* = 241.0), 141.59, 138.50, 136.56 (d, *J* = 3.8), 136.09, 135.56, 130.33, 128.97, 127.41, 124.20 (d, *J* = 9.9), 113.8 (d, *J* = 25.3), 108.33 (d, *J* = 23.5). GC/MS (*m*/*z*, %): 256.0 ([M$^+$], 100), 226.0 (35), 183.0 (31).

3-(4-Formylphenyl)-1-benzothiophene-5-carbonitrile (21).

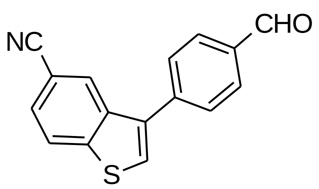

Compound **21** was prepared by method C, using compound **18** (300.0 mg, 1.260 mmol), 4-formylphenylboronic acid (236.2 mg, 1.575 mmol), $Pd(OAc)_2$ (14.1 mg, 0.0628 mmol), SPhos (103.4 mg, 0.2519 mmol), 2 M Na_2CO_3 (2.4 mL), DME (3.6 mL + 3.6 mL) and EtOH (3.6 mL). The product was purified using column chromatography (dry-flash, SiO_2, eluent hexane/CH_2Cl_2 gradient 9/1→1/9, CH_2Cl_2, CH_2Cl_2/MeOH = 9/1). Final product **21** was obtained as pale yellow powder (120 mg, 37%). M.p. = 210 – 215 °C. IR (ATR): 3084m, 2854m, 2756w, 2229s, 2011w, 1686s, 1604s, 1568w, 1438w, 1389m, 1344w, 1303w, 1211s, 1167m, 1106m, 1059w, 1014w, 893w, 856w, 833s, 811s, 798m, 737w, 654w, 560w, 485w cm^{-1}. ^1H NMR (500 MHz, C_5D_5N, δ): 10.23 (s, H-CHO), 8.36-8.33 (m, H-C(4)), 8.12 (d, *J* = 8.2, H-C(7)), 8.10-8.07 (m, 2H-Ar), 7.97 (s, H-C(2)), 7.77-7.73 (m, 2H-Ar), 7.71-7.68 (m, H-C(6)). ^{13}C NMR (125 MHz, C_5D_5N, δ): 192.58, 145.72, 141.19, 138.04, 136.85, 136.31, 131.08, 130.03, 129.34, 128.19, 127.54, 125.21, 120.27, 109.35.

3-(4-Formylphenyl)-1-benzothiophene-6-carbonitrile (22).

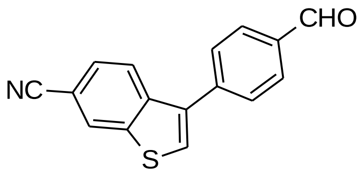

Compound **22** was prepared by method C, using compound **19** (89.3 mg, 0.375 mmol), 4-formylphenylboronic acid (70.3 mg, 0.469 mmol), $Pd(OAc)_2$ (4.2 mg, 0.019 mmol), SPhos (30.8 mg, 0.0750 mmol), 2 M Na_2CO_3 (0.7 mL), DME (1 mL + 1 mL) and EtOH (1 mL) . The product was purified using column chromatography (dry-flash, SiO_2, eluent hexane/ CH_2Cl_2 gradient 9/1→2/8). Final product **22** was obtained as white powder (75 mg, 76%). M.p. = 186 – 188 °C. IR (ATR): 3083m, 2982w, 2837m, 2747m, 2225s, 2176w, 1998w, 1686s, 1603s, 1572m, 1489m, 1454w, 1396m, 1348m, 1307m, 1264w, 1215s, 1171m, 1105w, 886w, 858w, 823s, 791m, 717w cm^{-1}. ^1H NMR (500 MHz, $CDCl_3$, δ): 10.11 (s, H-CHO), 8.29-8.27 (m, H-C(7)), 8.06-8.02 (m, 2H-Ar), 7.99-7.96 (m, H-C(4)), 7.78 (s, H-C(2)), 7.75-7.72 (m, 2H-Ar), 7.68-7.64 (m, H-C(5)). ^{13}C NMR (125 MHz, $CDCl_3$, δ): 191.56; 140.74; 140.64; 140.14; 137.06; 135.87; 130.38; 129.46; 129.23; 127.78; 127.36; 123.41; 118.94; 108.29. HRMS: *m/z* 264.04675 corresponds to molecular formula $C_{16}H_9NOSH^+$ (error in ppm -3.84).

***N*-[4-(5-fluoro-1-benzothien-3-yl)benzyl]-*N'*-quinolin-4-ylpropane-1,3-diamine (23).**

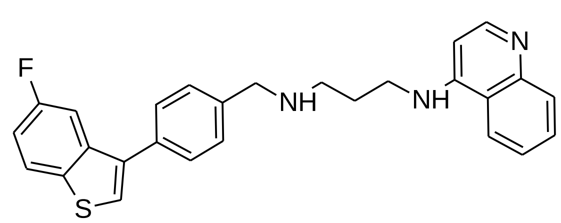

Compound **23** was prepared by method A, using aldehyde **20** (65.3 mg, 0.255 mmol), **AQ7** [1] (76.9 mg, 0.382 mmol), glac. AcOH (22 μL, 0.38 mmol) and MeOH/CH_2Cl_2 (18 mL, 2:1, v/v). The product was purified using column chromatography (dry-flash, SiO_2, eluent hexane/EtOAc gradient 1/1 → EtOAc, EtOAc/MeOH gradient 95/5 → 4/6). Final product **23** was obtained as a pale yellow foam (66.2 mg, 59%). M.p. = 37 – 40 °C. IR (ATR): 3648w, 3254m, 3063m,

2927m, 2849m, 1720w, 1583s, 1542m, 1495w, 1438m, 1401w, 1371w, 1338w, 1255w, 1196w, 1132w, 884w, 863w, 808w, 766m, 737m, 651w, 542w, 432w cm^{-1}. ^1H NMR (500MHz, $CDCl_3$, δ): 8.51 (d, *J* = 5.2, H-C(2')), 7.96 (d, *J* = 7.8, H-C(8′)), 7.84 (dd, J_1 = 4.9, J_2 = 8.8, H-C(7)), 7.71-7.67 (m, H-C(4)), 7.59-7.53 (m, 4H-Ar), 7.49-7.46 (m, 3H-Ar), 7.26-7.22 (m, H-C(6′)), 7.18-7.12 (m, H-C(6)), 6.35 (d, *J* = 5.5, H-C(3')), 3.92 (s, 2H, ArCH_2-), 3.45 (t, *J* = 6.0, 2H, ArNHCH_2-), 3.02 (t, *J* = 5.6, 2H, ArCH_2NHCH_2-), 2.64 (bs, H-N exchangeable with D_2O), 1.99 (quin, *J* = 5.8, 2H, ArNHCH_2CH_2-). ^{13}C NMR (125 MHz, $CDCl_3$, δ): 161.13 (d, *J* = 240.1), 150.72, 150.50, 147.71, 139.18, 139.06 (d, *J* = 8.8), 137.48 (d, *J* = 4.5), 136.01, 134.62, 129.09, 128.91, 128.70, 125.77, 124.34, 124.05 (d, *J* = 9.0), 120.47, 118.92, 113.39 (d, *J* = 25.3), 108.48 (d, *J* = 23.5), 98.00, 54.07, 49.24, 43.87, 27.60. HRMS: *m/z* 221.59133 corresponds to molecular formula $C_{27}H_{24}N_3SFH_2^{2+}$ (error in ppm 1.38); *m/z* 442.17549 corresponds to molecular formula $C_{27}H_{24}N_3SFH^+$ (error in ppm 1.61). HPLC purity: method A (*λ* = 330 nm): RT 11.323, area 96.48%; method B (*λ* = 254 nm): RT 7.986, area 95.32%.

***N*¹-(quinolin-4-yl)hexane-1,6-diamine AQ9.**

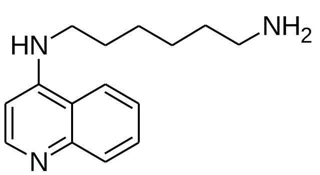

The general procedure D was followed using 4-chloroquinoline (300 mg, 1.83 mmol) and 1,6-diaminohexane (1.49 g, 12.8 mmol). The final product was obtained as brown oil (249.8 mg, 55%). IR (ATR): 3318m, 3079m, 2928m, 2854m, 1618w, 1582s, 1545m, 1502w, 1468w, 1439m, 1376m, 1342m, 1262w, 1126w, 935w, 884w, 809w, 771m cm^{-1}. ^1H NMR (500 MHz, CD_3OD, δ): 8.34 (d, *J* = 5.5, H-C(2′)), 8.09 (d, *J* = 8.5, H-C(8′)), 7.80 (d, *J* = 8.3, H-C(5′)), 7.63 – 7.60 (m, H-C(7′)), 7.44 – 7.41 (m, H-C(6′)), 6.48 (d, *J* = 5.7, H-C(3′)), 3.35 (t, 2H, *J* = 3.6, H-C(1)), 2.64 – 2.61 (m, 2H, H-C(6)), 1.79 – 1.73 (m, 2H, H-C(2)), 1.53 – 1.38 (m, 6H, 2H-C(3), 2H-C(4) and H-C(5)). ^{13}C NMR (125 MHz, CD_3OD, δ): 152.82, 151.38, 149.10, 130.54, 128.96, 125.64, 122.32, 120.45, 99.24, 44.05, 42.60, 33.84, 29.58, 28.26, 27.94. HRMS *m/z* 244.18071 corresponds to molecular formula $C_{15}H_{21}N_3$ (error in ppm -0.49).

***N*-[4-(5-fluoro-1-benzothien-3-yl)benzyl]-*N*'-quinolin-4-ylhexane-1,6-diamine (24).**

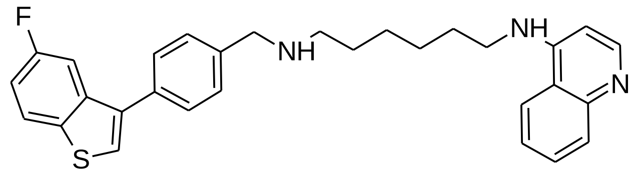

Compound **24** was prepared by method A, using aldehyde **20** (62.8 mg, 0.245 mmol), **AQ9** (89.4 mg, 0.368 mmol), glac. AcOH (21 μL, 0.37 mmol) and MeOH/CH_2Cl_2 (18 mL, 2:1, v/v). The product was purified using column chromatography (dry-flash, SiO_2, eluent EtOAc/hexane gradient 1/9 → EtOAc, EtOAc/MeOH gradient 95/5 → 2/8 and flash, Biotage SP1, NH column, eluent EtOAc/hexane gradient 8/2 → EtOAc, EtOAc/MeOH gradient 95/5 → MeOH). Final product **24** was obtained as a pale yellow oil (74.2 mg, 63%). IR (ATR): 3258w, 3068w, 2930m, 2855w, 1584s, 1540m, 1495w, 1439m, 1397w, 1374w, 1342w, 1252w, 1196w, 1117w, 884w, 864w, 809w, 768w, 738w, 652w cm^{-1}. ^1H NMR (500MHz, $CDCl_3$, δ):

8.56-8.53 (m, H-C(2')), 8.00-7.96 (m, H-C(8′)), 7.81 (dd, J_1 = 4.9, J_2 = 8.8, H-C(7)), 7.73 (d, J = 8, H-C(5′)), 7.63-7.58 (m, H-C(7′)), 7.58-7.53 (m, H-C(4)), 7.52-7.48 (m, H-C(10) and H-C(11)), 7.45-7.42 (m, H-C(8) and H-C(9) and H-C(2)), 7.41-7.37 (m, H-C(6′)), 7.16-7.10 (m, H-C(6)), 6.40 (d, J = 5.4, H-C(3')), 5.09 (bs, H-N exchangeable with D_2O), 3.85 (s, 2H, ArC*H*2-), 3.32-3.26 (m, 2H, ArNHC*H*2-), 2.69 (t, J = 7.1, 2H, $ArCH_2NHCH_2$-), 1.94 (bs, H-N exchangeable with D_2O), 1.79-1.72 (m, 2H, $ArNHCH_2CH_2$-), 1.61-1.54 (m, 2H, $ArCH_2NHCH_2CH_2$-), 1.52-1.41 (m, 4H, $ArNHCH_2CH_2CH_2$- and $ArCH_2NHCH_2CH_2CH_2$-). ^{13}C NMR (125 MHz, $CDCl_3$, δ): 161.03 (d, J = 240.1), 150.93, 149.67, 148.27, 140.09, 139.07 (d, J = 9.0), 137.62 (d, J = 4.5), 135.93, 134.09, 129.80, 128.94, 128.54, 128.45, 125.51, 124.51, 123.93 (d, J = 9.0), 119.20, 118.63, 113.24 (d, J = 24.4), 108.47 (d, J = 23.5), 98.69, 53.74, 49.32, 43.12, 29.99, 28.82, 27.04. HRMS: *m/z* 484.22010 corresponds to molecular formula $C_{30}H_{30}N_3SFH^+$ (error in ppm -3.35). HPLC purity (λ = 330 nm): method A: RT 9.792, area 95.26%; method B: RT 8.382, area 95.22%.

***N*⁴-(7-chloroquinolin-4-yl)-*N*¹-[4-(5-fluoro-1-benzothien-3-yl)benzyl]pentane-1,4-diamine (25).**

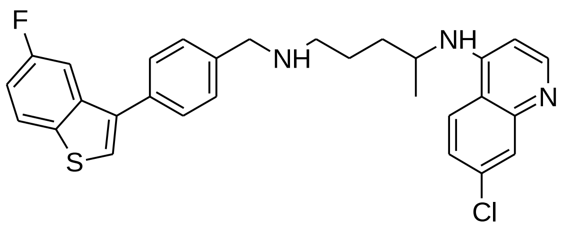

Compound **25** was prepared by method A, using aldehyde **20** (61.5 mg, 0.240 mmol), amine N^4-(7-chloroquinolin-4-yl)pentane-1,4-diamine [1] (94.9 mg, 0.360 mmol), glac. AcOH (21 μL, 0.36 mmol) and $MeOH/CH_2Cl_2$ (15 mL, 2:1, v/v). The product was purified using column chromatography (dry-flash, SiO_2, eluent hexane/EtOAc gradient 1/1 → EtOAc, EtOAc/MeOH gradient 95/5 → 6/4). Final product **25** was obtained as a white powder (79.4 mg, 66%). M.p. = 44 – 47 °C. IR (ATR): 3247w, 3074w, 2929w, 2858w, 1607m, 1576s, 1536w, 1491w, 1441m, 1375w, 1334w, 1252w, 1197w, 1142w, 882w, 810w, 649w cm^{-1}. ^1H NMR (500MHz, $CDCl_3$, δ): 8.51 (d, J = 5.2, H-C(2')), 7.94-7.93 (m, H-C(8′)), 7.83 (dd, J_1 = 4.8, J_2 = 8.8, H-C(7)), 7.64 (d, J = 9.2, H-C(5′)), 7.58-7.54 (m, H-C(4)), 7.53, 7.44 (ABq, J_{AB} = 8.2, 4H-Ar), 7.46 (s, H-C(2)), 7.28-7.24 (m, H-C(6′)), 7.15 (td, J_1 = 2.8, J_2 = 8.8, H-C(6)), 6.41 (d, J = 5.5, H-C(3')), 5.35-5.30 (m, H-N exchangeable with D_2O), 3.86 (s, 2H, ArC*H*2-), 3.77-3.68 (m, 1H, ArNHC*H*(CH_3)-), 2.77-2.71 (m, 2H, $ArCH_2NHCH_2$-), 1.86-1.47 (m, 5H, $ArNHCH(CH_3)CH_2$- and $ArNHCH(CH_3)CH_2CH_2$- and H-N exchangeable with D_2O), 1.33 (d, J = 6.4, 3H, $ArNHCH(CH_3)$-). ^{13}C NMR (125 MHz, $CDCl_3$, δ): 161.08 (d, J = 240.1), 152.00, 149.35, 148.98, 139.79, 139.08 (d, J = 9.0), 137.56 (d, J = 3.8), 135.98, 134.73, 134.30, 128.82, 128.56, 128.54, 125.63, 125.00, 123.98 (d, J = 9.0), 121.05, 117.27, 113.31 (d, J = 25.3), 108.49 (d, J = 23.5), 99.17, 53.75, 49.00, 48.30, 34.14, 26.47, 20.24. HRMS: *m/z* 252.58726 corresponds to molecular formula $C_{29}H_{27}ClN_3SFH_2^{2+}$ (error in ppm 0.26); *m/z* 504.16695 corresponds to molecular formula $C_{29}H_{27}ClN_3SFH^+$ (error in ppm -0.30). HPLC purity: method A (λ = 330 nm): RT 9.825, area 97.97%; method B (λ = 254 nm): RT 8.027, area 96.12%.

N^1-[4-(5-fluoro-1-benzothien-3-yl)benzyl]-N^4-quinolin-4-ylpentane-1,4-diamine (26).

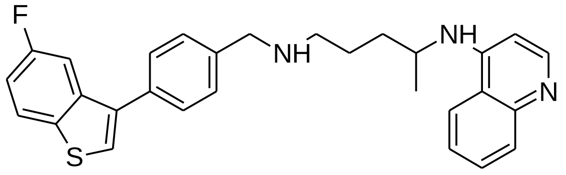

Compound **26** was prepared by method A, using aldehyde **20** (94.2 mg, 0.368 mmol), amine **S3** (126.4 mg, 0.5513 mmol), glac. AcOH (30 μL, 0.5 mmol) and MeOH/CH_2Cl_2 (24 mL, 2:1, v/v). The product was purified using column chromatography (dry-flash, SiO_2, eluent hexane/EtOAc gradient 1/1 → EtOAc, EtOAc/MeOH gradient 95/5 → 4/6 and flash, Biotage SP1, NH column, eluent EtOAc/hexane gradient 8/2 → EtOAc, EtOAc/MeOH gradient 95/5 → MeOH). Final product **26** was obtained as a colorless oil (66.1 mg, 38%). IR (ATR): 3265w, 3068w, 2928m, 2855w, 1579s, 1536m, 1496w, 1439m, 1396w, 1341w, 1254w, 1224w, 1196w, 1135w, 1116w, 885w, 865w, 808w, 766w, 737w cm^{-1}. ^1H NMR (500MHz, $CDCl_3$, δ): 8.53 (d, *J* = 5.2, H-C(2')), 7.96 (d, *J* = 8.4, H-C(8′)), 7.82 (dd, J_1 = 5.0, J_2 = 8.8, H-C(7)), 7.72 (d, *J* = 8.2, H-C(5′)), 7.62-7.57 (m, H-C(7′)), 7.57-7.54 (m, H-C(4)), 7.50, 7.42 (ABq, J_{AB} = 8.0, 4H-Ar), 7.42 (s, H-C(2)), 7.37-7.32 (m, H-C(6′)), 7.17-7.11 (m, H-C(6)), 6.42 (d, *J* = 5.3, H-C(3')), 5.24-5.19 (m, H-N exchangeable with D_2O), 3.86 (s, 2H, ArC*H*2-), 3.78-3.69 (m, 1H, ArNHC*H*(CH_3)-), 2.78-2.70 (m, 2H, $ArCH_2NHCH_2$-), 1.85-1.50 (m, 5H, ArNHCH(CH_3)C*H*2- and ArNHCH(CH_3)CH_2C*H*2- and H-N exchangeable with D_2O), 1.33 (d, *J* = 6.4, 3H, ArNHCH(C*H*3)-). ^{13}C NMR (125 MHz, $CDCl_3$, δ): 161.05 (d, *J* = 240.1), 151.00, 148.84, 148.62, 139.91, 139.06 (d, *J* = 9.0), 137.58 (d, *J* = 4.5), 135.94, 134.19, 129.97, 128.85, 128.53, 128.50, 125.56, 124.32, 123.95 (d, *J* = 9.0), 119.31, 118.80, 113.37 (d, *J* = 25.3), 108.48 (d, *J* = 24.4), 98.86, 53.72, 49.05, 48.16, 34.18, 26.52, 20.30. HRMS: *m/z* 470.20471 corresponds to molecular formula $C_{29}H_{28}N_3SFH^+$ (error in ppm -2.90). HPLC purity (λ = 330 nm): method A: RT 10.651, area 97.28%; method B: RT 8.430, area 97.16%.

N^1-[4-(5-fluoro-1-benzothien-3-yl)benzyl]-N^1-methyl-N^4-quinolin-4-ylpentane-1,4-diamine (27).

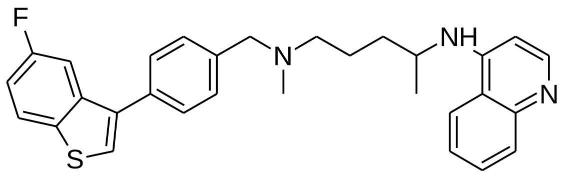

Compound **27** was prepared by method E, using compound **26** (20.0 mg, 0.0426 mmol), 37% aqueous formaldehyde (6.4 μL, 0.085 mmol), $ZnCl_2$ (11.6 mg, 0.0852 mmol), $NaBH_3CN$ (10.7 mg, 0.170 mmol) and MeOH (1 mL + 1 mL). The product was purified using column chromatography (dry-flash, SiO_2, eluent hexane/EtOAc gradient 1/1 → EtOAc, EtOAc/MeOH gradient 95/5 → 1/1 and flash, Biotage SP1, NH column, eluent EtOAc/hexane gradient 8/2 → EtOAc, EtOAc/MeOH gradient 95/5 → MeOH). Final product **27** was obtained as a colorless oil (12.3 mg, 60%). IR (ATR): 3730w, 3625w, 3276m, 3068m, 2931m, 2851m, 2792m, 1720w, 1579s, 1536m, 1496w, 1439m, 1395w, 1342m, 1253w, 1195w, 1136w, 1115w, 1058w, 1019w, 883w, 864w, 807w, 765w, 736w, 654w, 571w, 542w, 423w cm^{-1}. ^1H NMR (500MHz, $CDCl_3$, δ): 8.51 (d, *J* = 5.4, H-C(2')), 7.98-7.95 (m, H-C(8′)), 7.82 (dd, J_1 = 4.8, J_2 = 8.8, H-C(7)), 7.73-7.70 (m, H-C(5′)), 7.62-

7.55 (m, H-C(7′) and H-C(4)), 7.50, 7.43 (ABq, J_{AB} = 8.2, 4H-Ar), 7.46 (s, H-C(2)), 7.38-7.33 (m, H-C(6′)), 7.17-7.11 (m, H-C(6)), 6.41 (d, *J* = 5.4, H-C(3')), 5.22-5.16 (m, H-N exchangeable with D_2O), 3.77-3.69 (m, 1H, ArNHC*H*(CH_3)-), 3.56 (s, 2H, ArC*H*₂-), 2.50-2.41 (m, 2H, ArCH₂NHC*H*₂-), 2.23 (s, 3H, C*H*₃-N), 2.19 (bs, H-N exchangeable with D_2O), 1.82-1.65 (m, 4H, ArNHCH(CH_3)C*H*₂- and ArNHCH(CH_3)CH₂C*H*₂-), 1.33 (d, *J* = 6.4, 3H, ArNHCH(C*H*₃)-). ^{13}C NMR (125 MHz, $CDCl_3$, δ): 161.07 (d, *J* = 240.1), 150.91, 148.96, 148.52, 139.08 (d, *J* = 9.0), 138.64, 137.63 (d, *J* = 4.5), 135.98, 134.25, 129.88, 129.49, 128.91, 128.34, 125.57, 124.34, 123.97 (d, *J* = 9.0), 119.32, 118.79, 113.28 (d, *J* = 25.3), 108.52 (d, *J* = 23.5), 98.91, 62.06, 57.02, 48.11, 42.38, 34.28, 23.91, 20.33. HRMS: *m/z* 484.22192 corresponds to molecular formula $C_{30}H_{30}N_3SFH^+$ (error in ppm 0.41). HPLC purity (λ = 330 nm): method A: RT 9.127, area 97.38%; method B: RT 7.846, area 97.18%.

3-[4-({[4-(quinolin-4-ylamino)butyl]amino}methyl)phenyl]-1-benzothiophene-5-carbonitrile (28).

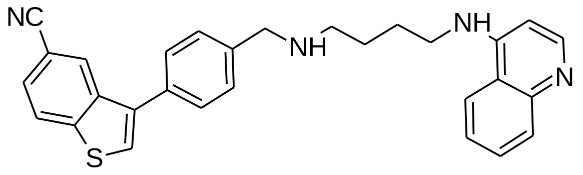

Compound **28** was prepared by method A, using aldehyde **21** (97.4 mg, 0.370 mmol), **AQ8** [1] (119.5 mg, 0.5548 mmol), glac. AcOH (32 μL, 0.55 mmol) and MeOH/CH_2Cl_2 (18 mL, 2:1, v/v). The product was purified using column chromatography (dry-flash, SiO_2, eluent hexane/EtOAc gradient 1/1 → EtOAc, EtOAc/MeOH gradient 95/5 → MeOH, flash, Biotage SP1, NH column, eluent EtOac/hexane gradient 8/2 → EtOAc, EtOAc/MeOH gradient 95/5 → 7/3 and flash, Biotage SP1, SiO_2 column, eluent EtOAc/MeOH+NH_3 (9/1) gradient 95/5 → 8/2). Final product **28** was obtained as a white foam (35.9 mg, 20%). M.p. = 54 – 60 °C. IR (ATR): 3280m, 3078m, 2932m, 2856m, 2225m, 1617w, 1580s, 1541m, 1495w, 1437w, 1398w, 1374w, 1341m, 1256w, 1222w, 1129w, 1058w, 808m, 765m, 655w, 560w cm^{-1}. ^1H NMR (500 MHz, $CDCl_3$, δ): 8.54 (d, *J* = 5.2, H-C(2′)), 8.20-8.19 (m, H-C(4)), 8.01-7.95 (m, H-C(7) and H-C(8′)), 7.75 (d, *J* = 8.2, H-C(5′)), 7.62-7.57 (m, H-C(7′) and H-C(6)), 7.52 (s, H-C(2)), 7.51-7.46 (m, 4H-Ar), 7.37-7.32 (m, H-C(6′)), 6.41 (d, *J* = 5.2, H-C(3′)), 5.69 (bs, H-N exchangeable with D_2O), 3.92 (s, 2H, ArC*H*₂-), 3.38-3.33 (m, 2H, ArNHC*H*₂-), 2.80 (t, *J* = 6.9, 2H, ArCH₂NHC*H*₂-), 1.94-1.81 (m, 3H, ArNHCH₂C*H*₂- and H-N exchangeable with D_2O), 1.80-1.71 (m, 2H, ArCH₂NHCH₂C*H*₂-). ^{13}C NMR(125 MHz, $CDCl_3$, δ): 150.91, 149.88, 148.26, 144.75, 140.35, 137.90, 133.31, 129.75, 128.94, 128.73, 128.71, 127.71, 126.27, 125.53, 124.43, 123.91, 119.51, 119.42, 118.76, 108.13, 98.62, 53.63, 48.73, 43.18, 27.76, 26.46. HRMS: *m/z* 232.10162 corresponds to molecular formula $C_{29}H_{26}N_4SH_2^{2+}$ (error in ppm 1.88); *m/z* 463.19503 corresponds to molecular formula $C_{29}H_{26}N_4SH^+$ (error in ppm -0.14). HPLC purity: method A (λ = 330 nm): RT 8.902, area 96.09%; method B (λ = 254 nm): RT 7.653, area 96.05%.

3-[4-({[4-(quinolin-4-ylamino)pentyl]amino}methyl)phenyl]-1-benzothiophene-5-carbonitrile (29).

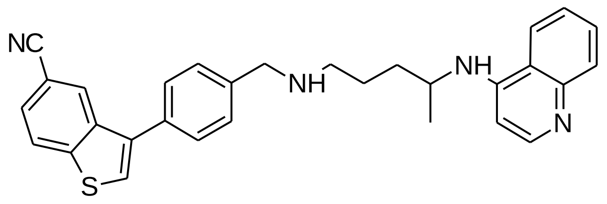

Compound **29** was prepared by method A, using aldehyde **21** (70.8 mg, 0.269 mmol), amine **S3** (89.2 mg, 0.389 mmol), glac. AcOH (23 μL, 0.39 mmol) and MeOH/CH_2Cl_2 (18 mL, 2:1, v/v). The product was purified using column chromatography (dry-flash, SiO_2, eluent hexane/EtOAc gradient 1/1 → EtOAc, EtOAc/MeOH gradient 95/5 → MeOH, flash, Biotage SP1, NH column, eluent EtOac/hexane gradient 8/2 → EtOAc, EtOAc/MeOH gradient 95/5 → 1/1 and flash, Biotage SP1, SiO_2 column, eluent EtOAc/MeOH+NH_3 (9/1) gradient 95/5 → 65/35). Final product **29** was obtained as a white foam (28.1 mg, 21%). M.p. = 71 – 74 °C. IR (ATR): 3395w, 3267w, 3071w, 2964w, 2929m, 2858w, 2225m, 1617w, 1577s, 1537m, 1495w, 1439m, 1395w, 1340m, 1282w, 1258w, 1221w, 1186w, 1136w, 1058w, 1020w, 892w, 809m, 765m, 656w, 632w, 560w cm^{-1}. ^1H NMR (500 MHz, $CDCl_3$, δ): 8.52 (d, *J* = 5.2, H-C(2′)), 8.19 (s, H-C(4)), 8.00-7.95 (m, H-C(7) and H-C(8′)), 7.74 (d, *J* = 8.2, H-C(5′)), 7.63-7.56 (m, H-C(6) and H-C(7′)), 7.52 (s, H-C(2)), 7.50-7.44 (m, 4H-Ar), 7.38-7.34 (m, H-C(6′)), 6.43 (d, *J* = 5.5, H-C(3′)), 5.25-5.20 (m, H-N exchangeable with D_2O), 3.88 (s, 2H, ArC*H*$_2$-), 3.79-3.72 (m, 1H, ArNHC*H*(CH_3)-), 2.77-2.71 (m, 2H, ArNHCH(CH_3)CH_2CH_2C*H*$_2$-), 1.86-1.67 (m, 5H, ArNHCH(CH_3)C*H*$_2$- and ArNHCH(CH_3)CH_2C*H*$_2$- and H-N exchangeable with D_2O), 1.34 (d, 3H, *J* = 6.2, ArNHCH(C*H*$_3$)-). ^{13}C NMR (125 MHz, $CDCl_3$, δ): 150.87, 148.93, 148.47, 144.74, 140.47, 137.91, 133.24, 129.86, 128.93, 128.73, 128.66, 127.71, 126.25, 125.49, 124.39, 123.89, 119.42, 119.32, 118.77, 108.11, 98.86, 53.66, 49.04, 48.19, 34.19, 26.52, 20.33. HRMS: *m/z* 239.10924 corresponds to molecular formula $C_{30}H_{28}N_4SH_2^{2+}$ (error in ppm 0.95); *m/z* 477.21086 corresponds to molecular formula $C_{30}H_{28}N_4SH^+$ (error in ppm 0.25). HPLC purity: method A (λ = 330 nm): RT 8.915, area 98.20%; method B (λ = 254 nm): RT 7.647, area 95.15%.

3-[4-({[6-(quinolin-4-ylamino)hexyl]amino}methyl)phenyl]-1-benzothiophene-5-carbonitrile (30).

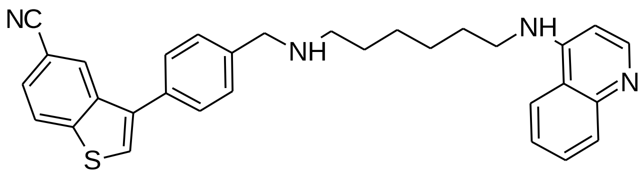

Compound **30** was prepared by method A, using aldehyde **21** (113.8 mg, 0.4322 mmol), **AQ9** (157.8 mg, 0.6486 mmol), glac. AcOH (37 μL, 0.65 mmol) and MeOH/CH_2Cl_2 (22.5 mL, 2:1, v.v). The product was purified using column chromatography (dry-flash, SiO_2, eluent hexane/EtOAc gradient 1/1 → EtOAc, EtOAc/MeOH gradient 95/5 → MeOH, flash, Biotage SP1, NH column, eluent EtOac/hexane gradient 8/2 → EtOAc, EtOAc/MeOH gradient 95/5 → 7/3 and flash, Biotage SP1, SiO_2 column, eluent EtOAc/MeOH+NH_3 (9/1) gradient 95/5 → 7/3). Final product **30** was obtained as a white foam (42.0 mg, 19%). M.p. = 45 – 49 °C. IR (ATR): 3301m, 3074m, 2928s, 2853m, 2225m, 1618w, 1579s, 1540s, 1495m, 1457w, 1436m, 1397m, 1374w, 1340m, 1254w, 1222w, 1128w, 1057w, 765m cm^{-1}. ^1H NMR (500 MHz,

$CDCl_3$, δ): 8.54 (d, *J* = 5.2, H-C(2′)), 8.20-8.19 (m, H-C(4)), 8.00-7.96 (m, H-C(7) and H-C(8′)), 7.73 (d, *J* = 8.0, H-C(5′)), 7.64-7.60 (m, H-C(7′)), 7.60-7.56 (m, H-C(6)), 7.52 (s, H-C(2)), 7.50-7.46 (m, 4H-Ar), 7.44-7.39 (m, H-C(6′)), 6.42 (d, *J* = 5.2, H-C(3′)), 5.06 (bs, H-N exchangeable with D_2O), 3.88 (s, 2H, ArC*H*2-), 3.36-3.30 (m, 2H, ArNHC*H*2-), 2.71 (t, *J* = 7.1, 2H, ArCH2NHC*H*2-), 1.88 (bs, H-N exchangeable with D_2O), 1.79 (quin, *J* = 7.1, 2H, ArNHCH2C*H*2-), 1.60 (quin, *J* = 7.1, 2H, ArCH2NHCH2C*H*2-), 1.53-1.43 (m, 4H, ArNHCH2CH2C*H*2- and ArCH2NHCH2CH2C*H*2-). ^{13}C NMR (125 MHz, $CDCl_3$, δ): 151.01, 149.62, 148.37, 144.74, 140.76, 137.98, 137.93, 133.14, 129.94, 128.92, 128.74, 128.63, 127.74, 126.24, 125.43, 124.52, 123.88, 119.44, 119.13, 118.65, 108.10, 98.74, 53.72, 49.33, 43.16, 30.05, 28.88, 27.08. HRMS: *m/z* 246.11747 corresponds to molecular formula $C_{31}H_{30}N_4SH_2^{2+}$ (error in ppm 2.58); *m/z* 491.22632 corresponds to molecular formula $C_{31}H_{30}N_4SH^+$ (error in ppm -0.15). HPLC purity: method A (*λ* = 330 nm): RT 9.042, area 99.09%; method B (*λ* = 254 nm): RT 7.712, area 96.73%.

3-[4-({[4-(quinolin-4-ylamino)butyl]amino}methyl)phenyl]-1-benzothiophene-6-carbonitrile (31).

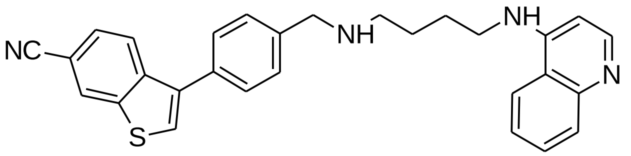

Compound **31** was prepared by method A, using aldehyde **22** (100.0 mg, 0.3798 mmol), **AQ8** [1] (122.6 mg, 0.5697 mmol), glac. AcOH (33 μL, 0.57 mmol) and MeOH/CH_2Cl_2 (18 mL, 2:1, v/v). The product was purified using column chromatography (dry-flash, SiO_2, eluent hexane/EtOAc gradient 1/1 → EtOAc, EtOAc/MeOH gradient 9/1 → MeOH, EtOAc/MeOH(NH_3 sat.)=95/5 flash, Biotage SP1, NH column, eluent EtOAc/hexane gradient 8/2 → EtOAc, EtOAc/MeOH gradient 95/5 → 7/3 and flash, Biotage SP1, SiO_2 column, eluent EtOAc/MeOH+NH_3 (9/1) gradient 95/5 → 8/2). Final product **31** was obtained as a white foam (92.2 mg, 52%). M.p. = 75 – 80 °C. IR (ATR): 3620w, 3269m, 3067m, 2935m, 2858m, 2224s, 1616w, 1580s, 1539s, 1449m, 1396m, 1374m, 1341s, 1258w, 1194w, 1129m, 946w, 825m, 765m, 622w cm^{-1}. ^{1}H NMR (500 MHz, $CDCl_3$, δ): 8.54 (d, *J* = 5.3 Hz, H-C(2′)), 8.24-8.22 (m, H-C(7)), 7.98-7.96 (m, H-C(4)), 7.95-7.93 (m, H-C(8′)), 7.74-7.73 (m, H-C(5′)), 7.63 (s, H-C(2)), 7.61-7.57 (m, H-C(5) and H-C(7′)), 7.51-7.46 (m, 4H-Ar), 7.34-7.31 (m, H-C(6′)), 6.41 (d, *J* = 5.2 Hz, H-C(3′)), 5.69 (bs, H-N, exchangeable with D_2O), 3.91 (s, 2H, ArC*H*2NH-), 3.38-3.33 (m, 2H, ArCH2NHC*H*2-), 2.80 (t, *J* = 6.9 Hz, 2H, ArNHC*H*2-), 1.94-1.88 (m, 2H, ArNHCH2C*H*2-), 1.82-1.72 (m, 3H, ArNHCH2CH2C*H*2- and H-N exchangeable with D_2O). ^{13}C NMR (125 MHz, $CDCl_3$, δ): 150.95; 149.84; 148.31; 140.71; 140.53; 140.26; 138.08; 133.49; 129.82; 128.92; 128.74; 128.66; 127.85; 127.63; 126.94; 124.41; 123.62; 119.50; 119.18; 118.77; 107.77; 98.62; 53.66; 48.80; 43.17; 27.76; 26.45. HRMS: *m/z* 463.19424 corresponds to molecular formula $C_{29}H_{26}N_4SH^+$ (error in ppm -1.85). HPLC purity (*λ* = 330 nm): method A: RT 8.824, area 96.60%; method B: RT 7.642, area 97.10%.

3-[4-({[6-(Quinolin-4-ylamino)hexyl]amino}methyl)phenyl]-1-benzothiophene-6-carbonitrile (32).

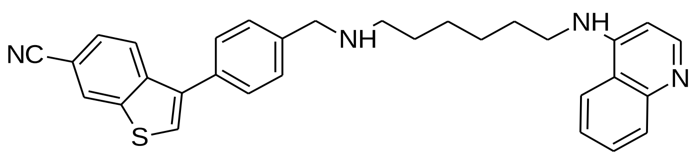

Compound **32** was prepared by method A, using aldehyde **22** (98.0 mg, 0.372 mmol), **AQ9** (135.9 mg, 0.5585 mmol), glac. AcOH (32 μL, 0.56 mmol) and MeOH/CH_2Cl_2 (18 mL, 2:1, v/v). The product was purified using column chromatography (dry-flash, SiO_2, eluent hexane/EtOAc gradient 1/1 → EtOAc, EtOAc/MeOH gradient 9/1 → MeOH, EtOAc/MeOH(NH_3 sat.) = 95/5 flash, Biotage SP1, NH column, eluent EtOAc/hexane gradient 8/2 → EtOAc, EtOAc/MeOH gradient 95/5 → 7/3 and flash, Biotage SP1, RP column, eluent MeOH/H_2O gradient 7/3 → MeOH). Final product **32** was obtained as a white foam (60.1 mg, 33%). M.p. = 68 – 75 °C. IR (ATR): 3402m, 3266m, 3069m, 2927s, 2853s, 2225m, 1669w, 1616w, 1580s, 1539s, 1452m, 1396m, 1374m, 1341s, 1258w, 1193w, 1127w, 1019w, 946w, 862w, 825m, 765m, 622w cm^{-1}. ^{1}H NMR (500 MHz, $CDCl_3$, δ): 8.55 (d, J = 5.5 Hz, H-C(2′)), 8.24-8.22 (m, H-C(7)), 7.99-7.97 (m, H-C(4)), 7.97-7.94 (m, H-C(8′)), 7.73-7.70 (m, H-C(5′)), 7.64-7.61 (m, 2H, H-C(7′) and H-C(2)), 7.61-7.58 (m, H-C(5)), 7.50-7.45 (m, 4H-Ar), 7.43-7.40 (m, H-C(6′)), 6.42 (d, J = 5.2 Hz, H-C(3′)), 5.00 (bs, H-N, exchangeable with D_2O), 3.87 (s, 2H, ArC*H*$_2$NH-), 3.35-3.31 (m, 2H, ArCH_2NHC*H*$_2$-), 2.71 (t, J = 7.1 Hz, 2H, ArNHC*H*$_2$-), 1.82-1.76 (m, 2H, ArNHCH_2C*H*$_2$-), 1.65-1.57 (m, 3H, ArCH_2NHCH_2C*H*$_2$- and H-N exchangeable with D_2O), 1.53-1.46 (m, 4H, -NHCH_2CH_2C*H*$_2$C*H*$_2$$CH_2CH_2$NH-). ^{13}C NMR (125 MHz, $CDCl_3$, δ): 151.03; 149.61; 148.40; 140.76; 140.66; 140.53; 138.19; 133.34; 130.00; 128.95; 128.66; 127.77; 127.63; 126.93; 124.56; 123.65; 119.19; 119.09; 118.66; 107.75; 98.76; 53.77; 49.45; 43.16; 30.06; 28.89; 27.09. HRMS: *m/z* 491.22593 corresponds to molecular formula $C_{31}H_{30}N_4SH^+$ (error in ppm -0.94). HPLC purity (λ = 330 nm): method A: RT 8.949, area 98.93%; method B: RT 7.699, area 98.28%.

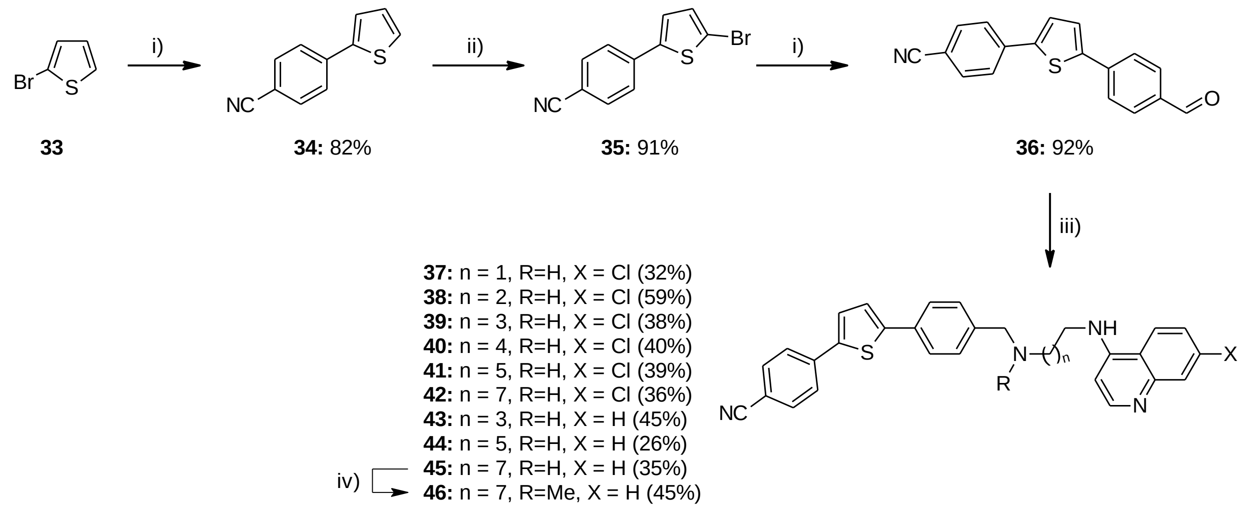

i) $ArB(OH)_2$, PdO x $1.4H_2O$, K_2CO_3, $EtOH/H_2O$, 60 °C; ii) Br_2, $C_2H_4Cl_2$, 0 °C to r.t.; iii) 1) aminoquinoline, AcOHglac, $MeOH/CH_2Cl_2$, r.t., 2 h 2) $NaBH_4$, r.t; iv) HCHO, $ZnCl_2$, $NaBH_3CN$, MeOH, r.t.

4-(thiophen-2-yl)benzonitrile(34).

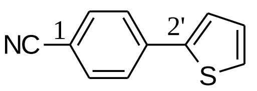

The general procedure F was followed using 2-bromothiophene (412 μL, 4.17 mmol), 4-cyanophenylboronic acid (734 mg, 5.00 mmol), PdO × 1.4 H_2O (6.2 mg, 0.042 mmol), K_2CO_3 (691 mg, 5.00 mmol), and ethanol/water (14 mL, 3:1, v/v). The purification of final product was performed using hexane/toluene as eluent to obtain **34** as a white powder (635 mg, 82%). M.p. = 78 – 80 °C. [3] IR (film): 3094w, 2925w, 2225w, 1912w, 1666w, 1603w, 1491w, 1421m, 1352w, 1311w, 1264w, 1211w, 1110w, 1052w, 959w, 849m, 823s, 770w, 713s cm^{-1}. ^1H NMR (500 MHz, $CDCl_3$, δ): 7.71 – 7.64 (m, 4H, H-C(2), H-C(3), H-C(5), H-C(6)), 7.43 – 7.40 (m, 2H, H-C(3′) and H-C(5′)), 7.13 (dd, J_1 = 5.0, J_2 = 3.7, H-C(4′)). ^{13}C NMR (125 MHz, $CDCl_3$, δ): 142.10, 138.69, 132.76, 128.54, 127.08, 126.12, 125.12, 118.84, 110.60. GC/MS (m/z (%)): 185.0 ([M^+], 100); 140.0 (16).

4-(5-bromothiophen-2-yl)benzonitrile (35).

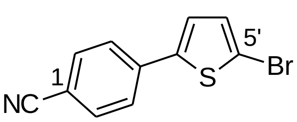

The general procedure B was followed using the solution of **34** (303 mg, 1.64 mmol) in 1,2-dichloroethane (5 mL) and the solution of bromine (92 μL, 1.8 mmol) in 1,2-dichloroethane (5 mL). The crude product was subjected to reversed-phase flash chromatography, Biotage SP1, using $MeOH/H_2O$ as eluent to afford final product **35** as a white powder (393 mg, 91%). M.p. = 96 °C. [4] (Ref: 98 – 99 °C) IR (film): 3092w, 2919w, 2850w, 2229w, 1912w, 1774w, 1654w, 1602w, 1558w, 1536w, 1495w, 1426m, 1329w, 1309w, 1247w, 1209w, 1179w, 1110w, 1067w, 977w, 942w, 834m, 804s, 737w, 661w cm^{-1}. ^1H NMR (500 MHz, $CDCl_3$, δ): 7.67 – 7.58 (m, 4H, 4H, H-C(2), H-C(3), H-C(5), H-C(6)), 7.16 (d, J = 3.8, H-C(3′)), 7.08 (d, J = 4.0, H-C(4′)). ^{13}C NMR (125

MHz, $CDCl_3$, δ): 143.41, 137.76, 132.88, 131.39, 125.78, 125.31, 118.64, 114.16, 111.05. GC/MS (m/z (%)): 264.8 ([M^+], 100); 140.1 (68).

4-[5-(4-formylphenyl)thiophen-2-yl]benzonitrile (36).

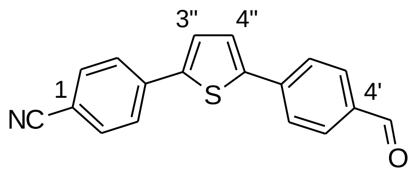

The general procedure F was followed using **35** (886 mg, 3.35 mmol), 4-formylphenylboronic acid (603 mg, 4.02 mmol), PdO × 1.4 H_2O (24.7 mg, 0.0419 mmol), K_2CO_3 (556 mg, 4.02 mmol) and ethanol/water (40 mL, 3:1, v/v). The purification of final product was performed using toluene/ethyl-acetate as eluent to obtain **36** as a yellow solid (1.07 g, 92%). M.p. = 212 – 214 °C. IR (film): 2916w, 2849w, 2754w, 2222m, 1698s, 1598s, 1535w, 1492w, 1449w, 1396w, 1342w, 1306w, 1277w, 1214m, 1167m, 1110w, 833s, 792s, 696w cm^{-1}. ^{1}H NMR (500 MHz, $(CD_3)_2SO$, δ): 10.01 (s, H-CO), 7.98 – 7.89 (m, 8H, H-C(2), H-C(3), H-C(5), H-C(6), H-C(2′), H-C(3′), H-C(5′) and H-C(6′)), 7.84 (bs, 2H, H-C(3″) and H-C(4″)). ^{13}C NMR (125 MHz, $(CD_3)_2SO$, δ): 192.31, 143.19; 142.35; 138.51, 137.47, 135.30, 133.16, 130.47, 127.90, 127.65, 125.89, 125.78, 118.74, 110.04. HRMS: *m*/*z* 290.06339 corresponds to molecular formula $C_{18}H_{11}NOSH^+$ (error in ppm -0.08).

4-(5-{4-[({2-[(7-chloroquinolin-4-yl)amino]ethyl}amino)methyl]phenyl}-2-thienyl)benzonitrile (37).

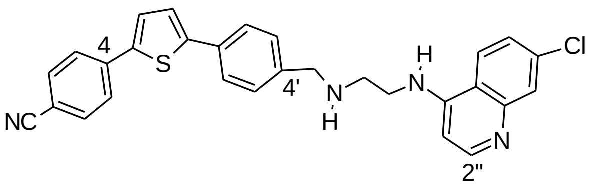

The general procedure A was followed using **36** (59.3 mg, 0.205 mmol), **AQ2** (66.5mg, 0.299 mmol), MeOH/ CH_2Cl_2 (5.3 mL, 2:1, v/v), glac. AcOH (18 μL, 0.30 mmol) and $NaBH_4$ (45.4 mg, 1.20 mmol). After working up the reaction in a manner provided in a general procedure, the crude product was subjected to silica-gel column chromatography using EtOAc/MeOH as eluent and NH flash chromatography, Biotage SP1, using hexane/EtOAc and EtOAc/MeOH as eluents. The final product **37** was obtained as a yellow solid (33 mg, 32%). M.p. = 147 – 149 °C. IR (film): 3245w, 3067w, 2954w, 2841w, 2224w, 1603w, 1579s, 1541w, 1493w, 1452w, 1432w, 1367w, 1333w, 1279w, 1250w, 1206w, 1139w, 878w, 837w, 793m, 767w, 737w, 705w, 684w, 659w, 650w, 620w, 604w, 601w cm^{-1}. ^{1}H NMR (500 MHz, $CDCl_3$ + CD_3OD, δ): 8.41 (d, *J* = 5.5, H-C(2'')), 7.90 (d, *J* = 2.1, H-C(8'')), 7.83 (d, *J* = 8.9, H- C(5'')), 7.71, 7.67 (ABq, 4H, J_{AB} = 8.4, H-C(2), H-C(3), H-C(5) and H-C(6)), 7.59, 7.36 (ABq, 4H, J_{AB} = 8.1, H-C(2'), H-C(3'), H-C(5') and H-C(6')), 7.42, 7.32 (Abq, J_{AB} = 3.7, 2H, H-ArS), 7.39 (dd, J_1 = 2.1, J_2 = 8.9, H-C(6'')), 6.38 (d, *J* = 5.5, H-C(3'')), 3.87 (s, 2H, $ArCH_2$-), 3.43 – 3.39 (m, 2H, ArNH*CH₂*-, ovlp with solvent signal), 3.06 – 3.04 (m, 2H, $ArNHCH_2$*CH₂*-). ^{13}C NMR (125 MHz, $CDCl_3$ + CD_3OD, δ): 151.32, 150.29, 148.38, 145.42, 140.87, 139.17, 138.45, 135.15, 132.79, 132.67, 128.76, 127.58, 126.08, 125.85, 125.60, 125.33, 124.32, 121.66, 118.74, 117.21, 98.74, 52.88, 46.67, 42.01. HRMS:

m/z 495.13994 corresponds to molecular formula $C_{29}H_{23}ClN_4SH^+$ (error in ppm -1.06). HPLC purity (λ = 330 nm): method E: RT 6.741, area 96.66%, method F: RT 7.940, area 96.79%.

4-(5-{4-[({3-[(7-chloroquinolin-4-yl)amino]propyl}amino)methyl]phenyl}-2-thienyl)benzonitrile (38).

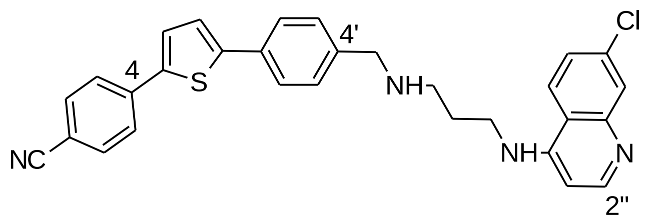

The general procedure A was followed using **36** (137.2 mg, 0.4742 mmol), **AQ3** (168 mg, 0.711 mmol), MeOH/ CH_2Cl_2 (12 mL, 2:1, v/v), glac. AcOH (43 μL, 0.71 mmol) and $NaBH_4$ (107.6 mg, 2.844 mmol). After working up the reaction in a manner provided in a general procedure, the crude product was subjected to silica-gel column chromatography using EtOAc/MeOH as eluent and NH flash chromatography, Biotage SP1, using hexane/EtOAc and EtOAc/MeOH as eluents. The final product **38** was obtained as a yellow solid (141.8 mg, 59%). M.p. = 138 – 140 °C. IR (film): 3248 m, 3066w, 2946w, 2907w, 2846m, 2560w, 2357w, 2223m, 2184w, 2166w, 2097w, 1987w, 1962w, 1918w, 1602m, 1579s, 1537m, 1494m, 1450m, 1426m, 1364w, 1332m, 1281m, 1240w, 1183w, 1136m, 1106w, 1070w, 953w, 897w, 851m, 798m, 763w, 615w, 541w cm^{-1}. ^1H NMR (500 MHz, $CDCl_3$, δ): 8.50 (d, *J* = 5.3, H-C(2'')), 7.91 (d, *J* = 2.2, H-C(8'')), 7.69, 7.67 (ABq, 4H, *J* = 8.7, H-C(2), H-C(3), H-C(5) and H-C(6)), 7.61, 7.37 (ABq, 4 H, *J* = 8.3, H-C(2′), H-C(3′), H-C(5′), H-C(6′)), 7.50 (d, *J* = 8.9, H-C(5'')), 7.48 – 7.44 (m, H-N, exchangeable with D_2O), 7.42, 7.34 (ABq, 2H, *J* = 3.9, H-ArS), 7.12 (dd, J_1 = 2.2, J_2 = 8.9, H-C(6'')), 6.32 (d, *J* = 5.4, H-C(3'')), 3.87 (s, 2H, ArCH_2-), 3.42 – 3.39 (m, 2H, ArNHCH_2-), 3.00 – 2.98 (m, 2H, -CH_2NH$CH_2$$CH_2$-), 1.98 – 1.94 (m, 2H, -$CH_2$$CH_2$$CH_2$-), 1.68 (bs, H-N, exchangeable with D_2O). ^{13}C NMR (125 MHz, $CDCl_3$, δ): 152.18, 150.36, 149.19, 145.53, 141.09, 139.64, 138.49, 134.55, 132.91, 132.76, 129.06, 128.58, 126.17, 126.03, 125.71, 124.82, 124.46, 122.03, 118.85, 117.51, 110.48, 98.37, 53.94, 49.26, 43.92, 27.49. HRMS: *m/z* 509.15630 corresponds to molecular formula $C_{30}H_{25}ClN_4SH^+$ (error in ppm 0.35). HPLC purity (λ = 330 nm): method C: RT 8.427, area 97.74%, method D: RT 7.073, area 98.21%.

4-(5-{4-[({4-[(7-chloroquinolin-4-yl)amino]butyl}amino)methyl]phenyl}-2-thienyl)benzonitrile (39).

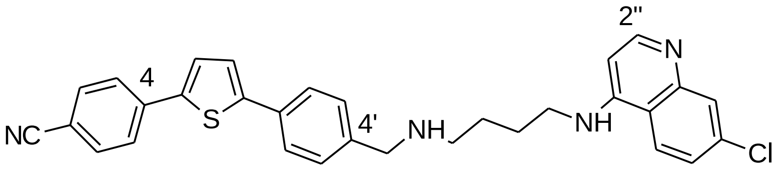

The general procedure A was followed using **36** (60 mg, 0.2 mmol), **AQ4** (77.7 mg, 0.311 mmol), MeOH/CH_2Cl_2 (5.3 mL, 2:1, v/v), glac. AcOH (19 μL, 0.31 mmol) and $NaBH_4$ (47 mg, 1.2 mmol). After working up the reaction in a manner provided in a general procedure, the crude product was subjected to silica-gel column chromatography using EtOAc/MeOH as eluent and NH flash chromatography, Biotage SP1, using hexane/EtOAc and EtOAc/MeOH as eluents. The final product **39** was obtained as a yellow solid (41.2 mg, 38%). M.p. = 163 –

164 °C. IR (film): 3278w, 3068w, 2935m, 222w, 1583s, 1544w, 1494w, 1451m, 1366w, 1338w, 1281w, 1209w, 1175w, 1139w, 1034w, 882w, 825m, 797m, 771w cm^{-1}. ^{1}H NMR (500 MHz, $CDCl_3$ + CD_3OD, δ): 8.42 (d, *J* = 5.5, H-C(2'')), 7.89 (d, *J* = 2.1, H- C(8'')), 7.77 (d, *J* = 8.9, H-C(5'')), 7.71, 7.66 (ABq, 4H, J_{AB} = 8.6, H-C(2), H-C(3), H-C(5) and H-C(6)), 7.60, 7.35 (ABq, 4H, J_{AB} = 8.1, H-C(2'), H-C(3'), H-C(5') and H-C(6')), 7.41, 7.32 (ABq, 2H, J_{AB} = 3.9, H-ArS), 7.30 – 7.27 (m, H-C(6'')), 6.37 (d, *J* = 5.6, H-C(3'')), 3.83 (s, 2H, ArCH_2-), 3.32 (t, 2H, *J* = 6.6, ArNHCH_2-), 2.74 (t, 2H, *J* = 6.9, $ArCH_2NH$$CH_2$-), 1.86 – 1.81 (m, 2H, $ArNHCH_2$$CH_2$-), 1.74 – 1.69 (m, 2H, $ArNHCH_2CH_2$$CH_2$-). ^{13}C NMR (125 MHz, $CDCl_3$ + CD_3OD, δ): 151.41, 148.48, 145.54, 140.87, 139.27, 138.47, 135.02, 132.73, 132.69, 128.79, 127.72, 126.08, 125.85, 125.62, 125.12, 124.31, 121.59, 118.78, 117.13, 110.23, 98.61, 53.31, 48.35, 42.84, 27.27, 26.03. HRMS: *m*/*z* 523.17161 corresponds to molecular formula $C_{31}H_{27}ClN_4SH^+$ (error in ppm -0.32). HPLC purity (λ = 330 nm): method C: RT 8.278, area 95.92%, method D: RT 7.072, area 96.32%.

4-(5-{4-[({5-[(7-chloroquinolin-4-yl)amino]pentyl}amino)methyl]phenyl}-2-thienyl)benzonitrile (40).

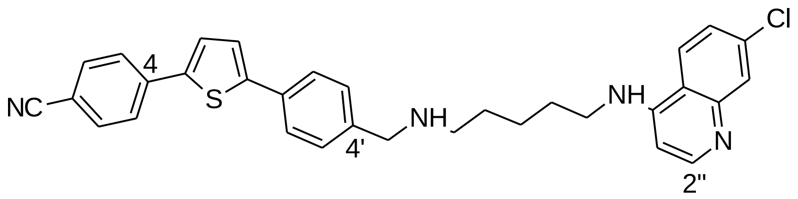

The general procedure A was followed using **36** (70.0 mg, 0.242 mmol), **AQ5** [2] (95.4 mg, 0.362 mmol), MeOH/CH_2Cl_2 (6 mL, 2:1, v/v), glac. AcOH (22 μL, 0.36 mmol) and $NaBH_4$ (54.8 mg, 1.45 mmol). After working up the reaction in a manner provided in a general procedure A, the crude product was subjected to silica-gel column chromatography using EtOAc/MeOH as eluent and NH flash chromatography, Biotage SP1, using hexane/EtOAc and EtOAc/MeOH as eluent. The final product **40** was obtained as a yellow solid (31.4 mg, 40%). M.p. = 150 – 152 °C. IR (film): 3405m, 3022w, 2988w, 2932m, 2858m, 2823w, 2219m, 2182w, 1604m, 1582s, 1531m, 1498w, 1478m, 1450m, 1371w, 1335w, 1278w, 1180w, 1134w, 1107w, 1076w, 902w, 879w, 850w, 831w, 801m, 739w cm^{-1}. ^{1}H NMR (500 MHz, $CDCl_3$ + CD_3OD, δ): 8.25 (d, *J* = 5.5, H-C(2'')), 7.73 (d, *J* = 2.1, H-C(8'')), 7.63 (d, *J* = 9.0, H-C(5'')), 7.54, 7.50 (ABq, 4H, J_{AB} = 8.6, H-C(2), H-C(3), H-C(5) and H-C(6)), 7.45 – 7.43 (m, 2H, H- C(2') and H-C(6')), 7.24 (d, *J* = 3.9, H-ArS), 7.19 – 7.15 (m, 4H, H-C(3'), H-C(5'), H-ArS and H-C(6'')), 6.22 (d, *J* = 5.6, H-C(3'')), 3.65 (s, 2H, ArCH_2-), 3.16 (t, 2H, *J* = 7.0, ArNHCH_2-), 2.79 (s, H-N, exchangeable with D_2O), 2.52 (t, 2H, *J* = 7.1, $ArCH_2NH$$CH_2$-), 1.63 – 1.57 (m, 2H, $ArNHCH_2$$CH_2$-), 1.49 – 1.43 (m, 2H, -$CH_2NHCH_2$$CH_2$-), 1.38 – 1.31 (m, 2H, $ArNHCH_2CH_2$$CH_2$-). ^{13}C NMR (125 MHz, $CDCl_3$ + CD_3OD, δ): 151.29, 150.29, 148.39, 145.55, 140.76, 139.23, 138.45, 135.02, 132.64, 128.82, 127.58, 126.06, 125.76, 125.56, 125.13, 124.25, 121.49, 118.75, 117.03, 110.12, 98.60, 53.21, 48.54, 42.73, 29.08, 28.09, 24.53. HRMS: *m*/*z* 537.18574 corresponds to molecular

formula $C_{32}H_{29}ClN_4SH^+$ (error in ppm -3.13). HPLC purity (λ = 330 nm): method C: RT 8.209, area 98.02%, method D: RT 6.985, area 97.40%.

4-(5-{4-[({6-[(7-chloroquinolin-4-yl)amino]hexyl}amino)methyl]phenyl}-2-thienyl)benzonitrile (41).

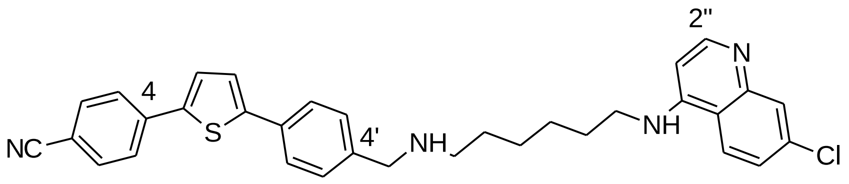

The general procedure A was followed using **36** (279 mg, 0.964 mmol), **AQ6** (401.7 mg, 1.466 mmol), MeOH/CH_2Cl_2 (23.5 mL, 2:1, v/v), glac. AcOH (87 μL, 1.4 mmol) and $NaBH_4$ (218.8 mg, 5.784 mmol). After working up the reaction in a manner provided in a general procedure, the crude product was subjected to silica-gel column chromatography using EtOAc/MeOH as eluent and NH flash chromatography, Biotage SP1, using hexane/EtOAc and EtOAc/MeOH as eluents. The final product **41** was obtained as a yellow solid (205.7 mg, 39%). M.p. = 130 – 131 °C. IR (film): 3411w, 3059w, 2927w, 2854w, 2221w, 1578s, 1451m, 1365w, 1323w, 1132w, 833w, 797w cm^{-1}. ^{1}H NMR (500 MHz, $CDCl_3$ + D_2O, δ): 8.51 (d, *J* = 8.3, H-C(2'')), 7.95 (d, *J* = 2.1, H-C(5'')), 7.70 – 7.58 (m, 7H, H-C(2), H-C(3), H-C(5), H-C(6), H-C(8''), H-C(2'), H-C(6')), 7.39 – 7.33 (m, 4H, H-ArS, H-C(3'), H-C(5'), H-C(6'')), 7.30 (d, *J* = 3.7, H-ArS), 6.40 (d, *J* = 5.5, H-C(3'')), 3.82 (s, 2H, Ar*CH*$_2$-), 3.30 (t, *J* = 7.2, 2H, ArNH*CH*$_2$-), 2.65 (t, *J* = 7.1, 2H, ArCH$_2$NH*CH*$_2$-), 1.79 – 1.73 (m, 2H, ArNHCH$_2$*CH*$_2$-), 1.59 – 1.43 (m, 6H, ArNHCH$_2$CH$_2$*CH*$_2$*CH*$_2$*CH*$_2$-). ^{13}C NMR (125 MHz, $CDCl_3$, δ): 152.05, 149.68, 149.13, 145.94, 140.78, 140.64, 138.55, 134.86, 132.76, 132.44, 128.89, 128.79, 126.10, 125.81, 125.68, 125.29, 124.22, 120.76, 118.88, 117.10, 110.42, 99.10, 53.68, 49.24, 43.21, 30.00, 28.85, 27.05. HRMS: *m*/*z* 551.20336 corresponds to molecular formula $C_{33}H_{31}ClN_4SH^+$ (error in ppm 0.53). HPLC purity (λ = 330 nm): method A: RT 8.681, area 95.22%, method B: RT 7.415, area 95.74%.

***N*-(7-chloroquinolin-4-yl)octane-1,8-diamine AQ88.**

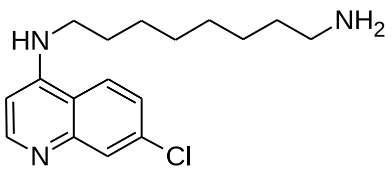

The general procedure D was followed using 4,7-dichloroquinoline (500 mg, 2 mmol) and 1,8-diaminooctane (1.8 g, 13 mmol). The final product **AQ88** was obtained as a pale yellow solid (535.6 mg, 69%). M.p. = 97 – 98 °C. IR (ATR): 3325m, 3262m, 2928m, 2851m, 1612m, 1577m, 1537m, 1480w, 1449w, 1431w, 1369m, 1330w, 1281w, 1251w, 1198w, 1134w, 1080w, 1027w, 960w, 922w, 903w, 876w, 852w, 809w, 771w, 729w, 649 cm^{-1}. ^{1}H NMR (500 MHz, $CDCl_3$ + D_2O, δ): 8.51 (d, *J* = 5.4, H-C(2')), 7.95 (d, *J* = 2.1, H-C(8')), 7.66 (d, *J* = 8.9, H-C(5')), 7.34 (dd, J_1 = 8.9, J_2 = 2.1, H-C(6')), 6.40 (d, *J* = 5.4, H-C(3')), 3.29 (t, 2H, *J* = 7.2, H-C(1)), 2.66 (t, 2H, *J* = 7.0, H-C(8)), 1.78 – 1.72 (m, 2H, H-C(2)), 1.48 – 1.32 (m, 10H, H-C(3), H-C(4), H-C(5), H-C(6) and H-C(7)). ^{13}C NMR (125 MHz, $CDCl_3$, δ): 152.02, 149.68, 149.12,

134.74, 128.79, 125.16, 120.83, 117.09, 99.02, 43.22, 42.16, 33.70, 29.34, 29.28, 28.84, 27.05, 26.76. HRMS: *m/z* 306.17168 corresponds to molecular formula $C_{17}H_{24}N_3ClH^+$ (error in ppm -4.82).

4-(5-{4-[({8-[(7-chloroquinolin-4-yl)amino]octyl}amino)methyl]phenyl}-2-thienyl)benzonitrile (42).

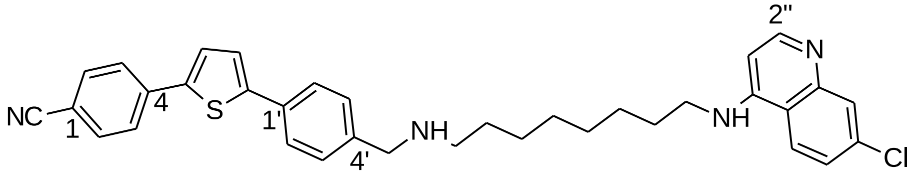

The general procedure A was followed using **36** (242 mg, 0.835 mmol), **AQ88** (383.4 mg, 1.253 mmol), $MeOH/CH_2Cl_2$ (23.5 mL, 2:1, v/v), glac. AcOH (75 μL, 1.2 mmol) and $NaBH_4$ (189.5 mg, 5.009 mmol). After working up the reaction in a manner provided in a general procedure A, the crude product was subjected to silica-gel column chromatography using EtOAc/MeOH as eluent and NH flash chromatography, Biotage SP1, using hexane/EtOAc and EtOAc/MeOH as eluents. The final product **42** was obtained as a pale yellow solid (173.8 mg, 36%). M.p. = 123 – 125 °C. IR (film): 3641w, 3612w, 3298m, 3072m, 2930s, 2855m, 2222m, 2173w, 1602m, 1581s, 1543m, 1495m, 1471m, 1450m, 1373m, 1332m, 1280w, 1207w, 1177w, 1137w, 1076w, 1022w, 907w, 877w, 840m, 798m, 770w, 733w, 648w, 538w cm^{-1}. ^{1}H NMR (500 MHz, $CDCl_3$ + D_2O, δ): 8.51 (d, J = 5.4, H-C(2'')), 7.95 (d, *J* = 2.0, H-C(8'')), 7.69 – 7.57 (m, 7H, H-C(2), H-C(3), H-C(5), H-C(6), H-C(2′), H-C(6′), H-C(5'')), 7.38 – 7.29 (m, 5H, H-(3′), H-C(5′), 2H-ArS, H-C(6'')), 6.39 (d, *J* = 5.3, H-C(3'')), 3.80 (s, Ar*CH*$_2$NH-), 3.28 (t, 2H, *J* = 7.1, ArNH*CH*$_2$-), 2.63 (t, 2H, *J* = 7.2, ArCH$_2$NH*CH*$_2$-), 1.77 – 1.71 (m, 2H, ArNHCH$_2$*CH*$_2$-), 1.54 – 1.42 (m, 4H, ArNHCH$_2$CH$_2$*CH*$_2$CH$_2$CH$_2$CH$_2$*CH*$_2$-), 1.42 – 1.29 (m, 6H, CH$_2$NHCH$_2$CH$_2$*CH*$_2$*CH*$_2$*CH*$_2$-). ^{13}C NMR (125 MHz, $CDCl_3$, δ): 152.01, 149.67, 149.08, 145.93, 140.69, 138.50, 134.78, 132.70, 132.33, 128.80, 128.74, 126.05, 125.74, 125.61, 125.20, 124.14, 120.76, 118.84, 117.07, 110.32, 99.03, 53.64, 49.38, 43.23, 30.02, 29.38, 29.24, 28.84, 27.21, 27.04. HRMS: *m/z* 579.23307 corresponds to molecular formula $C_{35}H_{35}N_4SClH^+$ (error in ppm -2.25). HPLC purity (λ = 330 nm): method D: RT 11.456, area 96.24%, method G: RT 6.658, area 99.44%.

4-{5-[4-({[4-(quinolin-4-ylamino)butyl]amino}methyl)phenyl]-2-thienyl}benzonitrile (43).

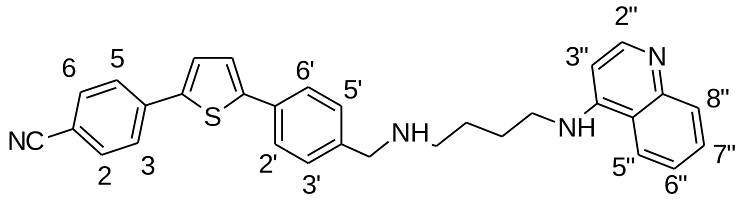

The general procedure A was followed using **36** (70.0 mg, 0.241 mmol), **AQ8** [1] (77.5 mg, 0.360 mmol), $MeOH/CH_2Cl_2$ (6 mL, 2:1, v/v), glac. AcOH (22 μL, 0.36 mmol) and $NaBH_4$ (54.5 mg, 1.44 mmol). After working up the reaction in a manner provided in a general procedure, the crude product was subjected to silica-gel column chromatography using

EtOAc/MeOH as eluent and NH flash chromatography, Biotage SP1, using hexane/EtOAc and EtOAc/MeOH as eluent. The final product **43** was obtained as a yellow solid (53 mg, 45%). M.p. = 117 – 118 °C. IR (film): 3401m, 3273m, 3067m, 2930m, 2856m, 2225m, 1584s, 1543m, 1497w, 1454m, 1376w, 1344m, 1279w, 1129w, 839w, 804m, 767w, 737w cm^{-1}. ^{1}H NMR (500 MHz, $CDCl_3$ + D_2O, δ): 8.53 (d, *J* = 5.3, H-C(2'')), 7.98 – 7.96 (m, H-C(8'')), 7.73 – 7.58 (m, 8H, H-C(5''), H-C(2), H-C(3), H-C(5), H-C(6), H-C(7''), H-C(2′) and H-C(6′)), 7.40 – 7.31 (m, 5H, 2H-ArS, H-C(3′), H-C(5′), H-C(6'')), 6.40 (d, *J* = 5.3, H-C(3'')), 3.84 (s, 2H, Ar-CH_2), 3.34 (t, *J* = 6.7, 2H, ArNHCH_2-), 2.75 (t, *J* = 6.8, 2H, -CH_2NH$CH_2$$CH_2$-), 1.91 – 1.85 (m, 2H, ArNH$CH_2$$CH_2$-), 1.75 – 1.70 (m, 2H, -CH_2NH$CH_2$$CH_2$-). ^{13}C NMR (125 MHz, $CDCl_3$, δ): 150.94, 149.85, 148.27, 145.80, 140.80, 140.26, 138.49, 132.71, 129.75, 128.93, 128.73, 126.08, 125.83, 125.64, 124.42, 124.24, 119.49, 118.84, 118.76, 110.37, 98.60, 53.55, 48.62, 43.14, 27.70, 26.40. HRMS: *m*/*z* 489.21074 corresponds to molecular formula $C_{31}H_{28}N_4SH^+$ (error in ppm -3.81). HPLC purity (*λ* = 330 nm): method C: RT 8.238, area 96.23%, method D: RT 6.871, area 96.87%.

4-{5-[4-({[6-(quinolin-4-ylamino)hexyl]amino}methyl)phenyl]-2-thienyl}benzonitrile (44).

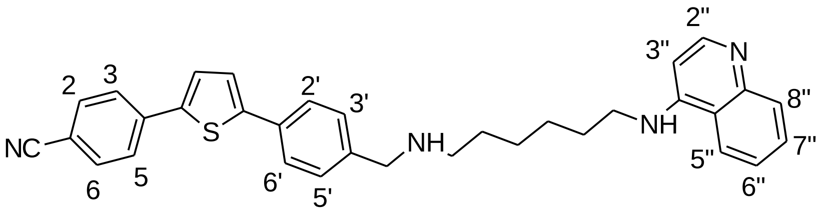

The general procedure A was followed using **36** (70.0 mg, 0.241 mmol), **AQ9** (88 mg, 0.36 mmol), MeOH/CH_2Cl_2 (6 mL, 2:1, v/v), glac. AcOH (22 μL, 0.36 mmol) and $NaBH_4$ (54.9 mg, 1.45 mmol). After working up the reaction in a manner provided in a general procedure A, the crude product was subjected to silica-gel column chromatography using EtOAc/MeOH as eluent and NH flash chromatography, Biotage SP1, using hexane/EtOAc and EtOAc/MeOH eluent. The final product **44** was obtained as a yellow solid (33 mg, 26%). M.p. = 117 – 119 °C. IR (film): 3298w, 3068w, 2926m, 2853m, 2224m, 1729w, 1581s, 1541m, 1496m, 1452m, 1375w, 1342m, 1279w, 1224w, 1177w, 1126w, 1017w, 972w, 837w, 801m, 765m, 733w, 671w cm^{-1}. ^{1}H NMR (500 MHz, $CDCl_3$, δ): 8.55 (d, *J* = 5.3, H-C(2'')), 7.98 (d, *J* = 7.8, H-C(8'')), 7.72 – 7.58 (m, 8H, H-C(5''), H-C(2), H-C(3), H-C(5), H-C(6), H-C(7''), H-C(2′) and H-C(6′)), 7.73 – 7.35 (m, 4H, H-C(6''), H-ArS, H-C(2′) and H-C(6′)), 7.30 (d, *J* = 3.9, H-ArS), 6.42 (d, *J* = 5.5, H-C(3'')), 4.97 (bs, H-N), 3.82 (s, 2H, Ar-CH_2), 3.33 – 3.29 (m, 2H, ArNHCH_2-), 2.66 (t, *J* = 7.1, 2H, -CH_2NH$CH_2$$CH_2$-), 1.80 – 1.74 (m, 2H, ArNH$CH_2$$CH_2$-), 1.60 – 1.54 (m, 2H, -CH_2NH$CH_2$$CH_2$-), 1.52 – 1.42 (m, 4H, ArNH$CH_2$$CH_2$$CH_2$$CH_2$-). ^{13}C NMR (125 MHz, $CDCl_3$, δ): 151.06, 149.59, 148.43, 145.94, 140.72, 138.52, 132.72, 132.36, 130.02, 128.92, 128.73, 126.06, 125.77, 125.63, 124.54, 124.17, 119.09, 118.85, 118.66, 110.36, 98.77, 53.67, 49.27, 43.15, 30.03, 28.88, 27.05. HRMS: *m*/*z* 517.24197 corresponds to molecular

formula $C_{33}H_{32}N_4SH^+$ (error in ppm -0.14). HPLC purity (λ = 330 nm): method A: RT 7.995, area 95.20%, method B: RT 9.836, area 95.69%.

4-{5-[4-({[8-(quinolin-4-ylamino)octyl]amino}methyl)phenyl]-2-thienyl}benzonitrile (45).

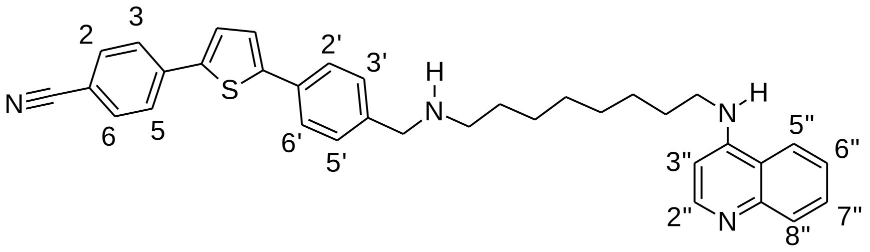

The general procedure A was followed using **36** (96.0 mg, 0.332 mmol), **AQ10** (135 mg, 0.497 mmol), MeOH/CH_2Cl_2 (7.5 mL, 2:1, v/v), glac. AcOH (31 μL, 0.50 mmol) and $NaBH_4$ (75.3 mg, 1.99 mmol). After working up the reaction in a manner provided in a general procedure A, the crude product was subjected to silica-gel column chromatography using EtOAc/MeOH as eluent and NH flash chromatography, Biotage SP1, using hexane/EtOAc and EtOAc/MeOH as eluents. The final product **45** was obtained as a yellow solid (62.7 mg, 35 %). M.p. = 110 – 112 °C. IR (film): 3255m, 3072m, 2928s, 2854m, 2226m, 1582s, 1541m, 1496w, 1454m, 1396w, 1375w, 1343m, 1280w, 1179w, 1128w, 838w, 803m, 766w, 737w cm^{-1}. ^{1}H NMR (500 MHz, $CDCl_3$ + D_2O, δ): 8.54 (d, *J* =5.4, H-C(2")), 7.98 (d, *J* = 8.3, H-C(8")), 7.72 – 7.58 (m, 8H, H-C(5"), H-C(7"), H-C(2), H-C(3), H-C(5), H-C(6), H-C(2') and H-C(6')), 7.43 – 7.35 (m, 4H, H-C(6"), H-ArS and H-C(3') and H-C(5')), 7.30 (d, *J* = 3.8, H-ArS), 6.4 (d, *J* = 5.3, H-C(3")), 3.80 (s, 2H, Ar*CH*$_2$-), 3.30 (t, *J* = 7.1, 2H, ArNH*CH*$_2$-), 2.63 (t, *J* = 7.2, 2H, -CH_2NH*CH*$_2$$CH_2$-), 1.78 – 1.72 (m, 2H, ArNHCH_2*CH*$_2$-), 1.54 – 1.35 (m, 10H, -CH_2NHCH_2*CH*$_2$*CH*$_2$*CH*$_2$*CH*$_2$*CH*$_2$-). ^{13}C NMR (125 MHz, $CDCl_3$, δ): 151.01, 149.64, 148.35, 145.96, 140.73, 140.70, 138.52, 132.72, 132.34, 129.94, 128.94, 128.75, 126.06, 125.76, 125.63, 124.54, 124.16, 119.09, 118.84, 118.64, 110.35, 98.74, 53.66, 49.42, 43.22, 30.05, 29.41, 29.27, 28.91, 27.22, 27.07. HRMS: *m/z* 545.27237 corresponds to molecular formula $C_{35}H_{36}N_4SH^+$ (error in ppm -1.78).

4-{5-[4-({methyl[8-(quinolin-4-ylamino)octyl]amino}methyl)phenyl]-2-thienyl}benzonitrile (46).

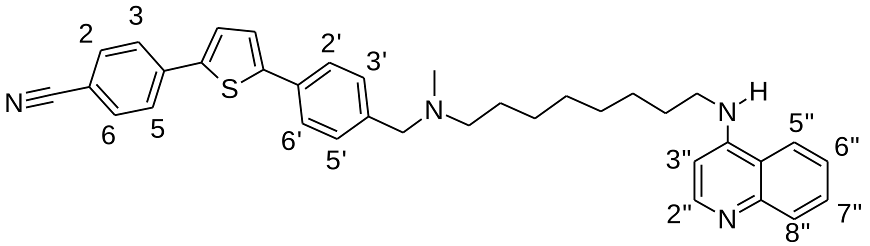

The general procedure E was followed using the solution of **45** (47.9 mg, 0.0879 mmol) in MeOH (2 mL), 37 % aqueous formaldehyde (13.2 μL, 0.176 mmol) and a mixture of $ZnCl_2$ (24.0 mg, 0.176 mmol), $NaBH_3CN$ (22.1 mg, 0.352 mmol) and MeOH (2 mL). After working up the reaction in a manner provided in a general procedure E, the crude product was subjected to NH flash chromatography, Biotage SP1, using hexane/EtOAc and EtOAc/MeOH as eluents. The final product **46** was obtained as a yellow solid (23.4 mg, 45 %). Mp = 99 – 100 °C. IR (film):

3285m, 3056m, 2926s, 2853s, 2225m, 1602s, 1582s, 1541s, 1497m, 1456s, 1372m, 1343m, 1273m, 1176m, 1124m, 1068m, 1016w, 838m, 802m, 765m, 736m cm^{-1}. ^{1}H NMR (500 MHz, $CDCl_3$ + D_2O, δ): 8.53 (d, *J* = 5.2, H-C(2'')), 7.98 (d, *J* = 8.4, H-C(8'')), 7.71 – 7.57 (m, 8H, H-C(5''), H-C(7''), H-C(2), H-C(3), H-C(5), H-C(6), H-C(2′) and H-C(6′)), 7.43 – 7.40 (m, 4H, H-C(6''), H-ArS, H-C(3′) and H-C(5′)), 7.30 (d, *J* = 3.9, H-ArS), 6.41 (d, *J* = 5.4, H-C(3'')), 3.50 (s, 2H, Ar-CH_2), 3.29 (t, *J* = 7.2, 2H, ArNHCH_2-), 2.39 – 2.36 (m, 2H, -$CH_2N(CH_3)$$CH_2$-), 2.21 (s, 3H, CH_3N), 1.78 – 1.72 (m, 2H, ArNH$CH_2$$CH_2$-), 1.54 – 1.33 (m, 10H, ArCH_2 N(CH3)$CH_2$$CH_2CH_2CH_2CH_2CH_2$-). ^{13}C NMR (125 MHz, $CDCl_3$, δ): 151.00, 149.69, 148.35, 146.08, 140.68, 139.67, 138.56, 132.75, 132.35, 129.96, 129.68, 129.00, 126.09, 125.65, 125.60, 124.58, 124.14, 119.11, 118.89, 118.66, 110.37, 98.78, 62.02, 57.45, 43.29, 42.36, 29.44, 29.34, 28.97, 27.38, 27.30, 27.14. HRMS: *m*/*z* 559.28899 corresponds to molecular formula $C_{36}H_{38}N_4SH^+$ (error in ppm -2.13). HPLC purity (*λ* = 330 nm): method I: RT 5.420, area 95.23 %, method H: RT 4.826, area 96.54 %.

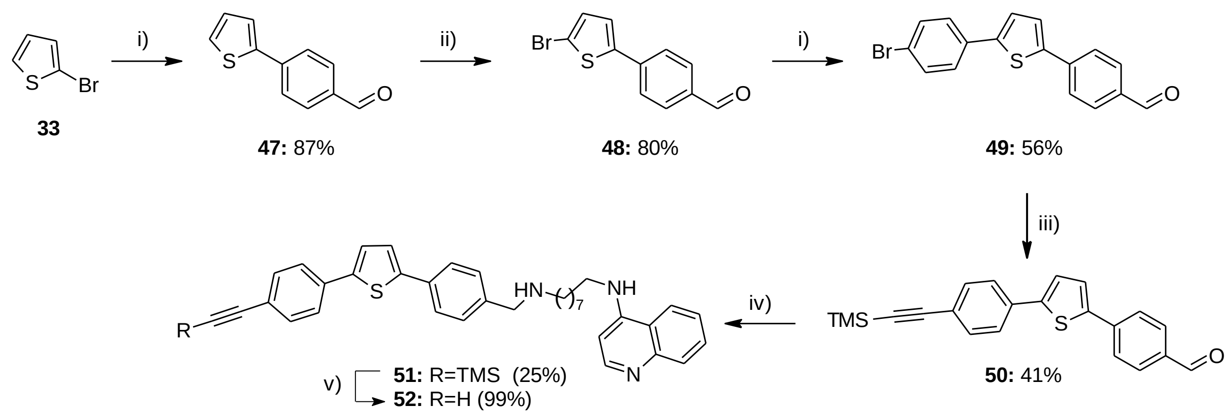

i) $ArB(OH)_2$, $Pd(OAc)_2$, PPh_3, Na_2CO_3, MeOH/PhMe, 110 °C; ii) NBS, THF, r.t.; iii) ethynyltrimethylsilane, $PdCl_2(PPh_3)_2$, PPh_3, CuI, Et_2NH, DMF, MW; iv) 1) AQ10, AcOH glac, MeOH/CH_2Cl_2, r.t., 2 h 2) $NaBH_4$, r.t.; v) K_2CO_3, MeOH, r.t.

4-(2-thienyl)benzaldehyde (47).

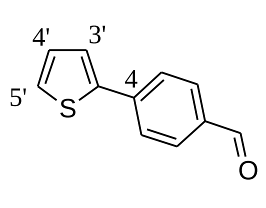

The general procedure G was followed using $Pd(OAc)_2$ (68.8 mg, 0.306 mmol) and PPh_3 (321.6 mg, 1.226 mmol) in toluene (35 mL), 2-bromothiophene (594 µL, 6.13 mmol), 4-formylphenylboronic acid (1.01 g, 6.74 mmol) and 2M aq. Na_2CO_3 (6 mL) in MeOH (6 mL). After colling to room temperature, the reaction mixture was extracted with CH_2Cl_2, organic layers were washed with brine and dried over anh. Na_2SO_4. After filtration, the solvent was removed under reduced pressure. The crude product was subjected to silica-gel flash chromatography, Biotage SP1, using hexane / EtOAc as eluent. The final product **47** was obtained as a pale yellow solid (1.00 g, 87%). M.p. = 60 – 61 °C. [5] IR (ATR): 3107w, 2926w, 2851w, 2738w, 2005w,

1703s, 1604m, 1565w, 1426w, 1216w, 1174w, 822m, 708w cm^{-1}. ^1H NMR (500 MHz, $CDCl_3$, δ): 10.00 (s, H-CO), 7.90 – 7.76 (m, 4H, H-C(2), H-C(3), H-C(5) and H-C(6)), 7.47 – 7.40 (m, 2H, H-C(3′) and H-C(5′)), 7.15 – 7.13 (m, H-C(4′)). ^{13}C NMR (125 MHz, $CDCl_3$, δ): 191.47, 142.74, 140.12, 135.10, 130.47, 128.47, 126.92, 126.05, 125.03. GC/MS (m/z (%)): 188.0 ([M^+], 100); 115.1 (40).

4-(5-bromo-2-thienyl)benzaldehyde (48).

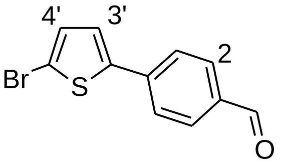

N-bromosuccinimide (228.9 mg, 1.286 mmol) was added to the stirring solution of **47** (226.4 mg, 1.203 mmol) in dry THF (11 mL) in the dark, at room temperature. Reaction progress was monitored by TLC (RP, MeOH). After 4h of stirring, an aq. $Na_2S_2O_3$ solution was added, and the desired product was extracted with CH_2Cl_2. Combined organic layers were washed with brine, and dried over anh. Na_2SO_4. After filtration, the solvent was removed under reduced pressure. The crude product was subjected to reversed-phase flash chromatography, Biotage SP1, using MeOH/H_2O as eluent. The final product **48** was obtained as an orange solid (256.1 mg, 80%). M.p. = 117 - 118 °C. IR (ATR): 3477s, 2922m, 2851m, 2759w, 1692s, 1666m, 1604m, 1566w, 1425m, 1397w, 1220w, 1204w, 1175m, 1110w, 832w, 796m, 686w, 652w cm^{-1}. ^1H NMR (500 MHz, $CDCl_3$, δ): 10.00 (s, H-CO), 7.88, 7.66 (ABq, J_{AB} = 8.3, 4H, H-C(2), H-C(3), H-C(5) and H-C(6)), 7.20, 7.09 (ABq, J_{AB} = 3.9, 2H, H-C(3′) and H-C(4′)). ^{13}C NMR (125 MHz, $CDCl_3$, δ): 191.29, 144.08, 139.14, 135.36, 131.32, 130.53, 125.70, 125.18, 113.92. GC/MS (m/z (%)): 267.9 ([M^+], 100); 158.0 (66).

4-[5-(4-bromophenyl)-2-thienyl]benzaldehyde (49).

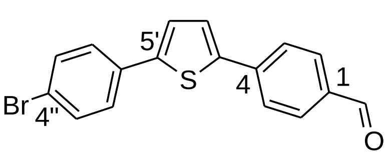

The general procedure G was followed using $Pd(OAc)_2$ (14.6 mg, 0.0650 mmol) and PPh_3 (68.7 mg, 0.262 mmol) in toluene (15 mL), **48** (350 mg, 1.3 mmol), 4-bromophenylboronic acid (289.2 mg, 1.440 mmol) and 2M aq. Na_2CO_3 (1.3 mL) in MeOH (6 mL) and toluene (15 mL). After colling to room temperature, the reaction mixture was diluted with CH_2Cl_2, filtered through a small pad of celite and dried over anh. Na_2SO_4. After filtration, the solvent was removed under reduced pressure. The crude product was subjected to silica-gel flash chromatography, Biotage SP1, using hexane / EtOAc as eluent. The final product **49** was obtained as a pale yellow solid (249.6 mg, 56%). IR (ATR): 3435s, 2922w, 2852w, 2728w, 1698s, 1662w, 1560s, 1564w, 1541w, 1508w, 1438w, 1450w, 1418w, 1394w, 1338w, 1307w, 1277w, 1213m, 1169m, 1112w, 1068w, 1007w, 939w, 831m, 803s, 695w, 671w, 633w cm^{-1}. ^1H NMR (500 MHz, $CDCl_3$, δ): 10.01 (s, H-CO), 7.90, 7.76 (ABq, 4H, J_{AB} = 8.4, H-C(2), H-C(3), H-C(5) and H-C(6)), 7.53, 7.50 (ABq, 4H, J_{AB} = 8.8, H-C(2''), H-C(3''), H-C(5'') and H-C(6'')), 7.02, 6.83 (ABq, 2H, J_{AB} = 4.0, H-C(3′) and H-C(4′)). ^{13}C NMR (125 MHz, $CDCl_3$, δ): 191.32, 144.42, 142.15, 139.72, 135.16, 132.76, 132.11, 130.48, 127.16, 126.08, 125.70, 124.73, 121.90. GC/MS (m/z (%)): 343.9 ([M^+], 100); 234.0 (54).

4-(5-{4-[(trimethylsilyl)ethynyl]phenyl}-2-thienyl)benzaldehyde (50).

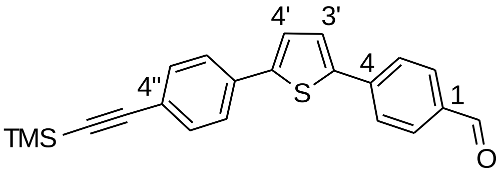

To a solution of **49** (71 mg, 0.21 mmol) in DMF (1 mL), $PdCl_2(PPh_3)_2$ (8.7 mg, 0.012 mmol), PPh_3 (10.8 mg, 0.0412 mmol), ethinyltrimethylsilane (32 μL, 0.027 mmol), CuI (2.3 mg, 0.012 mmol), and Et_2NH (323 μL, 3.12 mmol) were added under an argon atmosphere. The reaction mixture was subjected to microwave irradiation using Biotage Initiator 2.5 apparatus for 35 min at 120 °C. After cooling to room temperature, the reaction mixture was diluted with ether and CH_2Cl_2 and filtrate through a small pad of celite. The filtrate was washed with 0.1M HCl and extracted with CH_2Cl_2. The organic layer was washed with satd. aq. solution of $NaHCO_3$ and water and dried over anh Na_2SO_4. After filtration, the solvent was removed under reduced pressure. The crude product was subjected to silica-gel flash chromatography, Biotage SP1, using hexane / EtOAc as eluent. The final product **50** was obtained as a pale yellow solid (30.4 mg, 41%). IR (ATR): 3392w, 2962w, 2835w, 2742w, 2156w, 1702m, 1598m, 1566w, 1491w, 1449w, 1415w, 1341w, 1308w, 1250m, 1216w, 1169w, 1112w, 838m, 799m, 762w, 699w, 643w cm^{-1}. ^1H NMR (500 MHz, $CDCl_3$, δ): 10.00 (s, H-CO), 7.89, 7.76 (ABq, J_{AB} = 8.2, 4H, H-C(2), H-C(3), H-C(5) and H-C(6)), 7.57, 7.49 (ABq, J_{AB} = 8.4, 4H, H-C(2''), H-C(3''), H-C(5'') and H-C(6'')), 7.44, 7.34 (ABq, J_{AB} = 3.8, 2H, H-C(3′) and H-C(4′)), 0.27 (s, 9H, 3 × Si-CH_3). ^{13}C NMR (125 MHz, $CDCl_3$, δ): 191.40, 145.03, 142.33, 139.84, 135.21, 133.77, 132.63, 130.56, 126.18, 125.75, 125.38, 124.94, 122.69, 104.74, 95.79. GC/MS (m/z (%)): 360.1 ([M^+], 91); 345.1 (100).

N

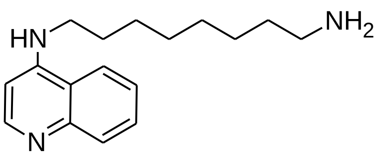

The general procedure D was followed using 4-chloroquinoline (650 mg, 4.0 mmol) and 1,8-diaminooctane (2.86 g, 19.8 mmol). The final product was obtained as a brown oil (701.9 mg, 65%). IR (ATR): 3290m, 2928s, 2854m, 1653w, 1583s, 1544m, 1462m, 1440m, 1396w, 1375w, 1342m, 1256w, 1127w, 882w, 809w, 767m cm^{-1}. ^1H NMR (500 MHz, $CDCl_3$, δ): 8.55 (d, *J* = 5.3, H-C(2′)), 7.99 – 7.97 (m, H-C(8′)), 7.74 – 7.72 (m, H-C(5′)), 7.64 – 7.60 (m, H-C(7′)), 7.43 – 7.40 (m, H-C(6′)), 6.42 (d, *J* = 5.4, H-C(3′)), 5.04 (bs, H-N), 3.33 – 3.29 (m, 2H, H-C(1)), 2.68 (t, 2H, *J* = 7.0, H-C(8)), 1.79 – 1.73 (m, 2H, H-C(2)), 1.50 – 1.30 (m, 12H, 2H-N, 2H-C(3), 2H-C(4), 2H-C(5), 2H-C(6) and H-C(7)). ^{13}C NMR (125 MHz, $CDCl_3$, δ): 151.03, 149.64, 148.40, 129.93, 128.89, 124.48, 119.14, 118.66, 98.72, 43.19, 42.16, 33.71, 29.30, 28.89, 27.06, 26.76. HRMS *m/z* 272.21141 corresponds to molecular formula $C_{17}H_{25}N_3H^+$ (error in ppm -2.62).

***N*-quinolin-4-yl-*N*'-[4-(5-{4-[(trimethylsilyl)ethynyl]phenyl}-2-thienyl)benzyl]octane-1,8-diamine (51).**

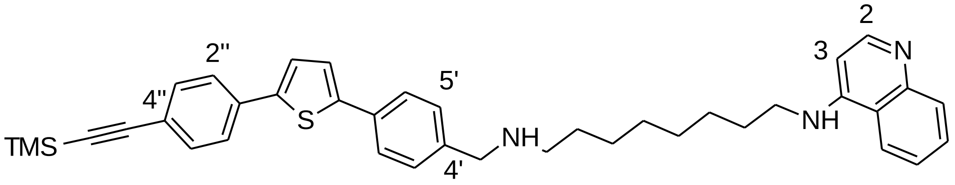

The general procedure A was followed using **50** (44.6 mg, 0.124 mmol), **AQ10** (40.2 mg, 0.148 mmol), MeOH/CH_2Cl_2 (5.4 mL, 2:1, v/v), glac. AcOH (12 μL, 0.21 mmol) and $NaBH_4$ (28 mg, 0.74 mmol). After working up the reaction in a manner provided in a general procedure A, the crude product was subjected to silica-gel column chromatography using EtOAc/MeOH as eluent and NH flash chromatography, Biotage SP1, using hexane/EtOAc and EtOAc/MeOH as eluent. The final product **51** was obtained as a pale yellow solid (19 mg, 25%). IR (ATR): 3244m, 3073m, 3025m, 2924s, 2852m, 2156m, 1582s, 1544m, 1496m, 1454m, 1398m, 1376m, 1280w, 1248m, 1130w, 1109w, 1017w, 943w, 863m, 841m, 802m, 760m, 664w cm^{-1}. ^{1}H NMR (500 MHz, $CDCl_3$ + D_2O, δ): 8.53 (d, *J* = 5.3, H-C(2)), 7.98 (d, *J* = 8.5, H-C(8)), 7.71 (d, *J* = 8.4, H-C(5)), 7.64 – 7.60 (m, H-C(7)), 7.58 – 7.54 (m, 4H, H-C(2′), H-C(6′), H-C(2'') and H-C(6'')), 7.47 – 7.46 (m, 2H, H-C(3'') and H-C(5'')), 7.43 – 7.40 (m, H-C(6)), 7.34 – 7.33 (m, 2H, H-C(5′) and H-C(3′)), 7.29 – 7.26 (m, 2H, ovlp with solvent signal, H-ArS), 6.41 (d, J = 5.3, H-C(3)), 3.79 (s, 2H, $ArCH_2$-), 3.30 (t, J = 7.2, 2H, $ArNHCH_2$-), 2.63 (t, J = 7.2, 2H, -CH_2NH*CH_2*CH_2-), 1.78 – 1.72 (m, 2H, $ArNHCH_2$*CH_2*-), 1.54 – 1.43 (m, 4H, -CH_2NHCH_2*CH_2*$(CH_2)_3$*CH_2*-), 1.39 – 1.35 (m, 6H, -$CH_2NHCH_2CH_2$*$CH_2CH_2CH_2$*-), 0.26 (s, 9H, 3 × Si-CH_3). ^{13}C NMR (125 MHz, $CDCl_3$, δ): 151.03, 149.70, 148.37, 144.19, 142.51, 140.20, 134.28, 132.84, 132.52, 129.96, 128.99, 128.73, 125.67, 125.14, 124.59, 124.57, 123.96, 121.99, 119.14, 118.68, 104.93, 98.79, 95.31, 53.72, 49.42, 43.27, 30.09, 29.46, 29.33, 28.97, 27.28, 27.12. HRMS: *m/z* 616.31641 corresponds to molecular formula $C_{39}H_{45}N_3SSiH^+$ (error in ppm -1.96).

***N*-{4-[5-(4-ethynylphenyl)-2-thienyl]benzyl}-*N*'-quinolin-4-yloctane-1,8-diamine (52).**

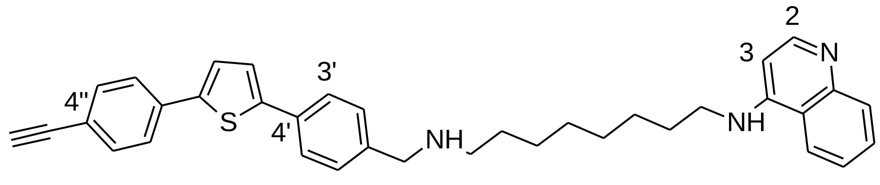

To a solution of **51** (16 mg, 0.026 mmol) in a dry MeOH (0.5 mL), anh. K_2CO_3 was added. The reaction mixture was stirred at room temperature for 24h. The organic product was extracted using ethyl-acetate, combined organic layers were washed with satd. aqueous solution of $NaHCO_3$ and dried over anh. $NaSO_4$. After filtration, the crude product was subjected to NH flash chromatography, Biotage SP1, using CH_2Cl_2/MeOH as eluent. The final product **52** was obtained as a pale yellow solid (14 mg, 99%). M.p. = 110 – 112 °C. IR (film): 3302s, 2928m, 2854m, 1583s, 1541m, 1496m, 1456m, 1397w, 1375w, 1342m, 1281w, 1127w, 940w, 837m, 801s, 765m, 737m, 647m cm^{-1}. ^{1}H NMR (500 MHz, $CDCl_3$, δ): 8,54 (d, *J* = 5.3, H-C(2)),

7.98 (dd, J_1 = 8.4, J_2 = 0.9, H-C(8), 7.72 – 7.71 (m, H-C(5)), 7.64 – 7.60 (m, H-C(7)), 7.59 – 7.56 (m, 4H, H-C(2''), H-C(3''), H-C(5'') and H-C(6'')), 7.50 – 7.49 (m, 2H, H-C(3′) and H-C(5′)), 7.43 – 7.40 (m, H-C(6)), 7.34 (d, *J* = 8.3, 2H, H-C(2′) and H-C(6′)), 7.31, 7.25 (ABq, J_{AB} = 3.8, 2H, H-ArS), 6.41 (d, *J* = 5.4, H-C(3)), 5.00 (bs, H-N, exchangeable with D_2O), 3.80 (s, 2H, ArCH_2NH-), 3.32 – 3.28 (m, 2H, ArNHCH_2-), 3.14 (s, HCC-Ar), 2.65 – 2.62 (m, 2H, ArCH$_2$NHCH_2-), 1.92 (bs, H-N, exchangeable with D_2O), 1.78 – 1.72 (m, 2H, ArNHCH$_2$$CH_2$-), 1.56 – 1.43 (m, 4H, ArNHCH$_2$CH$_2$$CH_2CH_2CH_2CH_2$$CH_2$-), 1.40 – 1.34 (m, 6H, CH$_2$NHCH$_2$CH$_2$$CH_2$$CH_2$$CH_2$-). ^{13}C NMR (125 MHz, $CDCl_3$, δ): 150.93, 149.75, 148.26, 144.31, 142.34, 140.18, 134.64, 132.80, 132.68, 129.86, 129.02, 128.74, 125.68, 125.26, 124.68, 124.60, 123.97, 120.93, 119.16, 118.66, 98.77, 83.51, 78.09, 53.69, 49.39, 43.27, 30.05, 29.44, 29.31, 28.95, 27.26, 27.11. HRMS: *m/z* 544.27759 corresponds to molecular formula $C_{36}H_{37}N_3SH^+$ (error in ppm -0.94). HPLC purity (λ = 330 nm): method A: RT 10.262, area 95.36%, method B: RT 11.233, area 95.16%.

In vitro antiplasmodial activity

The Malaria SYBR Green I - Based Fluorescence (MSF) Assay is a microtiter plate drug sensitivity assay that uses the presence of malarial DNA as a measure of parasitic proliferation in the presence of antimalarial drugs or experimental compounds. As the intercalation of SYBR Green I dye and its resulting fluorescence is relative to parasite growth, a test compound that inhibits the growth of the parasite will result in a lower fluorescence. D6 (CDC/Sierra Leone), TM91C235 (WRAIR, Thailand), and W2 (CDC/Indochina III) laboratory strains of *P. falciparum* were used for each drug sensitivity assessment. The parasite strains were maintained continuously in long-term cultures as previously described in Johnson et al [6]. Pre-dosed microtiter drug plates for use in the MSF assay were produced using sterile 384-well black optical bottom tissue culture plates containing quadruplicate 12 two-fold serial dilutions of each test compound suspended in dimethyl sulfoxide. The final concentration range tested was 0.5 – 10000 ng/ml for all assays. Predosed plates were stored at 4°C until used, not to exceed five days. No difference was seen in drug sensitivity determinations between stored or fresh drug assay plates (data not shown). A batch control plate using Chloroquine (Sigma-Aldrich Co., Catalog #C6628) at a final concentration of 2000 ng/ml was used to validate each assay run. The Tecan Freedom Evo liquid handling system (Tecan US, Inc., Durham, NC) was used to produce all drug assay plates. Based on modifications of previously described methods by Plouffe et al [7] and Johnson et al [6], P. falciparum strains in late-ring or early-trophozoite stages were cultured in the predosed 384-well microtiter drug assay plates in 38 µl culture volume per well at a starting parasitemia of 0.3% and a hematocrit of 2%. The cultures were then incubated at 37°C within a humidified atmosphere of 5% CO_2, 5% O_2 and 90% N_2, for 72 hours. Lysis buffer (38 µl per well), consisting of 20mM Tris HCl, 5mM EDTA, 1.6% Triton X, 0.016% saponin, and SYBR green I dye at a 20x concentration (Invitrogen, Catalog #S-7567) was then added to the assay plates for a final SYBR Green concentration of 10x. The Tecan Freedom Evo liquid handling system was used to dispense malaria cell culture and lysis buffer. The plates were then incubated in the dark at room temperature for 24 hours and examined for the relative fluorescence units (RFU) per well using the Tecan Genios Plus (Tecan US, Inc., Durham, NC). Each drug concentration was transformed into Log[X] and plotted against the RFU values. The 50% inhibitory concentrations (IC50s) were then generated with GraphPad Prism (GraphPad Software Inc., SanDiego, CA) using the nonlinear regression (sigmoidal dose-response/variable slope) equation.

---

1. Terzić, N.; Konstantinović, J.; Tot, M.; Burojević, J.; Đurković-Đaković, O.; Srbljanović, J.; Štajner, T.; Verbić, T.; Zlatović, M.; Machado, M.; Albuquerque, I. S.; Prudêncio, M.; Sciotti, R. J.; Pecic, S.; D'Alessandro, S.; Taramelli, D.; Solaja, B. A. Reinvestigating Old Pharmacophores: Are 4-Aminoquinolines and Tetraoxanes Potential Two-Stage Antimalarials? *J. Med. Chem.* **2016**, 59, 264 – 281, doi: 10.1021/acs.jmedchem.5b01374. Available online: http://pubs.acs.org/doi/abs/10.1021/acs.jmedchem.5b01374 (accessed on 19 December 2016).
2. Solaja, B. A.; Opsenica, D.; Smith, K. S.; Milhous, W. K.; Terzic, N.; Opsenica, I.; Burnett, J. C.; Nuss, J.; Gussio, R.; Bavari, S. Novel 4-Aminoquinolines Active against Chloroquine-Resistant and Sensitive P. falciparum Strains that also Inhibit Botulinum Serotype A. *J. Med. Chem.* **2008**, *51*, 4388 – 4391, doi: 10.1021/jm800737y. Available online: http://pubs.acs.org/doi/abs/10.1021/jm800737y (accessed on 19 December 2016)
3. Seggio, A.; Priem, G.; Chevallier, F.; Mongin, F. Palladium-Catalyzed Cross-Couplings of Lithium Arylzincates with Aromatic Halides: Synthesis of Analogues of Isomeridianin G and Evaluation as GSK-

3β Inhibitors *Synthesis* **2009**, *21*, 3617 – 3632, doi: 10.1055/s-0029-1217003. Available online: https://www.thieme-connect.com/products/ejournals/abstract/10.1055/s-0029-1217003.
4. Chaires, J. B.; Ren, J.; Hamelberg, D.; Kumar, A.; Pandya, V.; Boykin, D. W.; Wilson, W. D. Structural Selectivity of Aromatic Diamidines. *J. Med. Chem*. **2004**, *47*, 5729 – 5742, doi: 10.1021/jm049491e. Available online: http://pubs.acs.org/doi/abs/10.1021/jm049491e (accessed on 19 December 2016).
5. Baghbanzadeh, M.; Pilger, C.; Kappe, C. O. Palladium-Catalyzed Direct Arylation of Heteroaromatic Compounds: Improved Conditions Utilizing Controlled Microwave Heating. *J. Org. Chem*. **2011**, *76*, 8138 – 8142, doi: 10.1021/jo201516v. Available online: http://pubs.acs.org/doi/abs/10.1021/jo201516v (accessed on 19 December 2016).
6. Johnson, J. D.; Dennull, R. A.; Gerena, L.; Lopez-Sanchez, M.; Roncal, N. E.; Waters, N. C. Assessment and Continued Validation of the Malaria SYBR Green I-Based Fluorescence Assay for Use in Malaria Drug Screening. *Antimicrob. Agents Chemother.* **2007**, *51*, 1926-1933, doi: 10.1128/AAC.01607-06. Available online: https://www.ncbi.nlm.nih.gov/pmc/articles/PMC1891422/ (accessed on 27 January 2017).
7. Plouffe, D.; Brinker, A.; McNamara, C.; Henson, K.; Kato, N.; Kuhen, K.; Nagle, A.; Adrián, F.; Matzen, J. T.; Anderson, P.; Nam, T.G.; Gray, N. S.; Chatterjee, A.; Janes, J.; Yan, S. F.; Trager, R.; Caldwell, J.S.; Schultz, P.G.; Zhou, Y.; Winzeler, E. A. In silico activity profiling reveals the mechanism of action of antimalarials discovered in a high-throughput screen. *Proc. Natl. Acad. Sci. U.S.A.* **2008**, 105, 9059-9064, doi: 10.1073/pnas.0802982105. Available online: https://www.ncbi.nlm.nih.gov/pmc/articles/PMC2440361/ (accessed on 27 January 2017).
