# Supplementary material for: Antimalarials with Benzothiophene Moieties as Aminoquinoline Partners
Source: Molecules. 2017 Feb 24;22(3):343. doi: 10.3390/molecules22030343 (PMC6155332; doi:10.3390/molecules22030343)
Supplement: Supplementary file 1 [file molecules-22-00343-s001.zip › Supplementary_material_II_1Feb2017.pdf]

Supplementary Material – II

Antimalarials with benzothiophene moieties as aminoquinoline partners

Jelena Konstantinović[1], Milica Videnović[2], Jelena Srbljanović[3], Olgica Djurković-Djaković[3], Katarina Bogojević[1], Richard Sciotti[4] and Bogdan Šolaja[1,*]

[1] Faculty of Chemistry, University of Belgrade, Studentski trg 16, P.O. Box 51, 11158, Belgrade, Serbia; jelena_konstantinovic@chem.bg.ac.rs (J.K.), bogojevickatarina@gmail.com (K.B.), bsolaja@chem.bg.ac.rs (B.Š.)

[2] Innovation center of the Faculty of Chemistry, Studentski trg 12-16, 11158 Belgrade, Serbia; milica_videnovic @chem.bg.ac.rs (M.V.)

[3] Institute for Medical Research, University of Belgrade, Dr. Subotića 4, 11129 Belgrade, Serbia; jelena.srbljanovic@imi.bg.ac.rs (J.S.); olgicadj@imi.bg.ac.rs (O.DjDj.)

[4] Experimental Therapeutics Branch, Walter Reed Army Institute of Research, Silver Spring, Maryland 20910, United States; richard.j.sciotti.civ@mail.mil (R.S.)

[*] Correspondence: bsolaja@chem.bg.ac.rs; Tel.: +381-11-263-86-06

Table of contents

NMR spectra of synthesized compounds **II-S3**

HPLC analyses for purity **II-S31**

N-(1-benzothien-2-ylmethyl)-N'-quinolin-4-ylpropane-1,3-diamine (8)

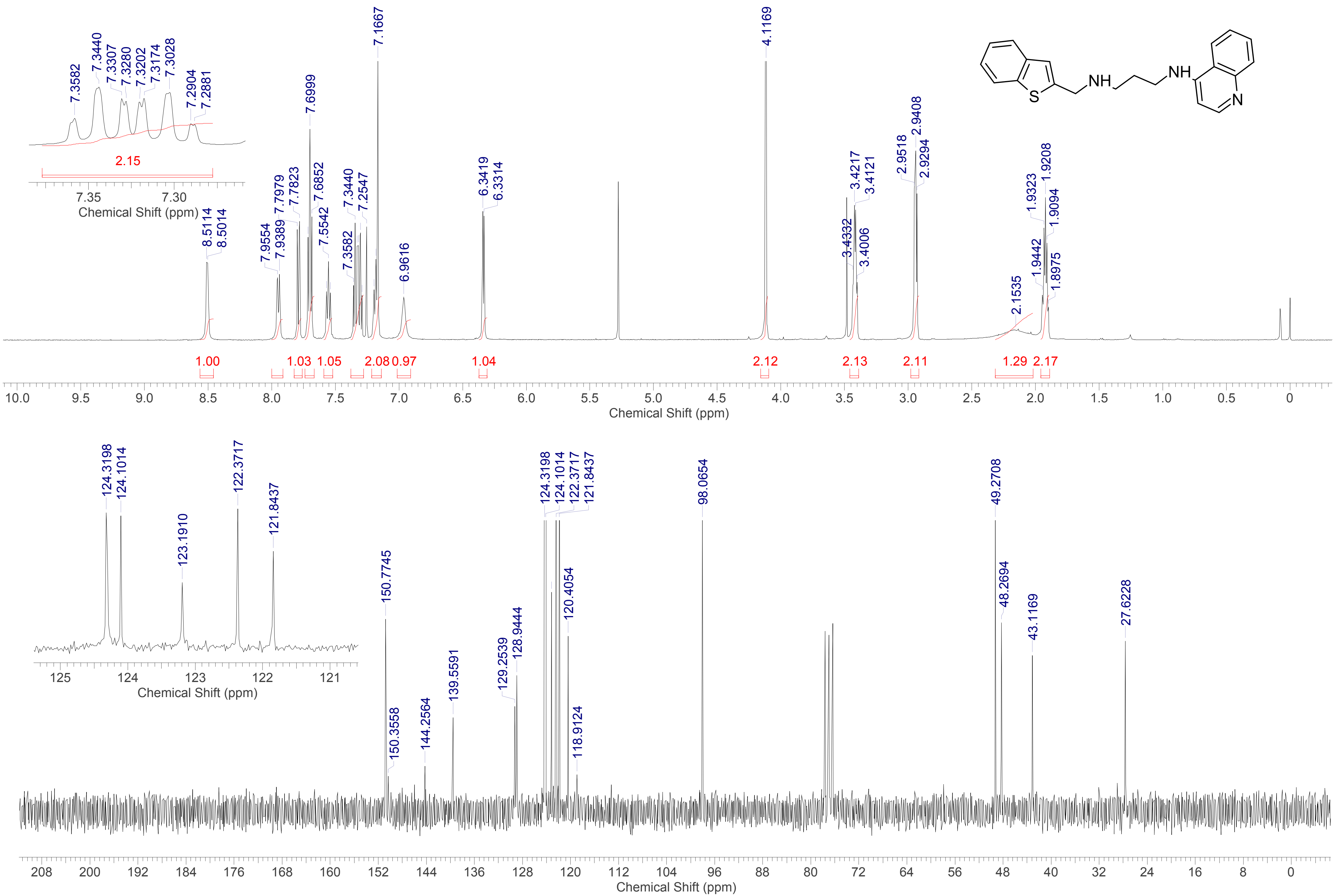

N-(1-benzothien-2-ylmethyl)-N'-quinolin-4-ylbutane-1,4-diamine (9)

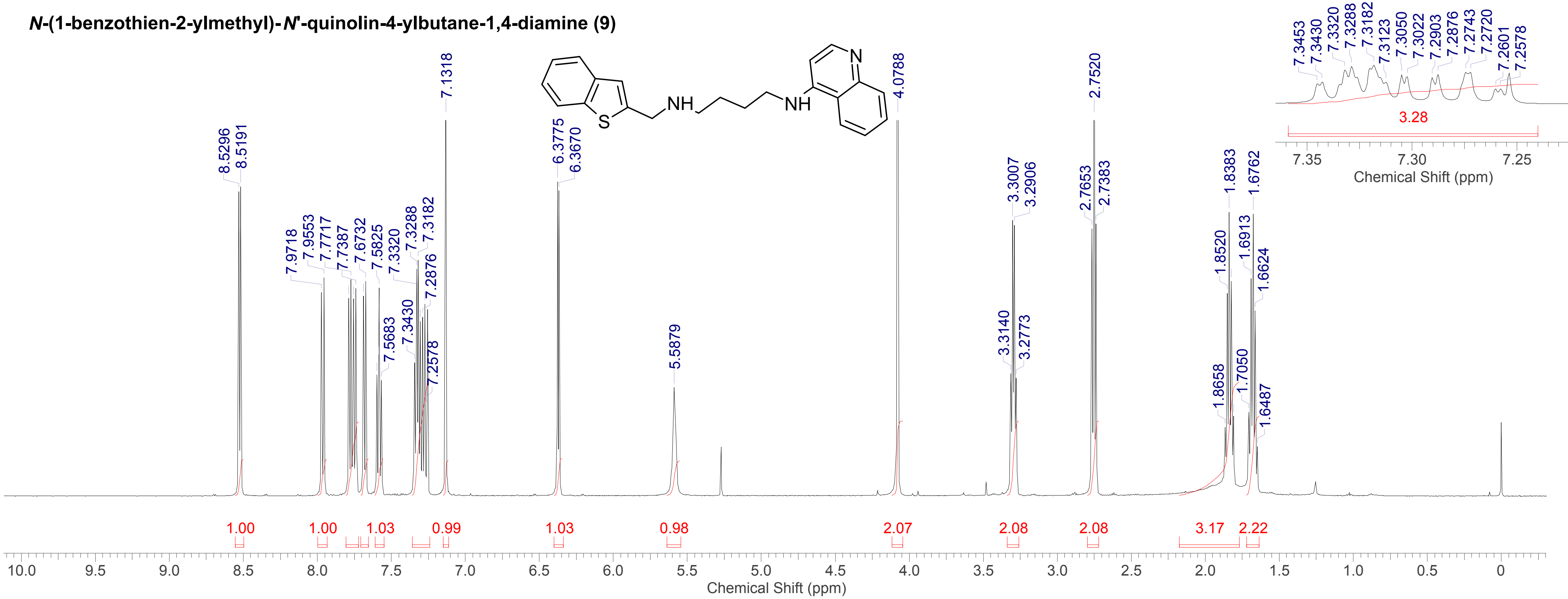

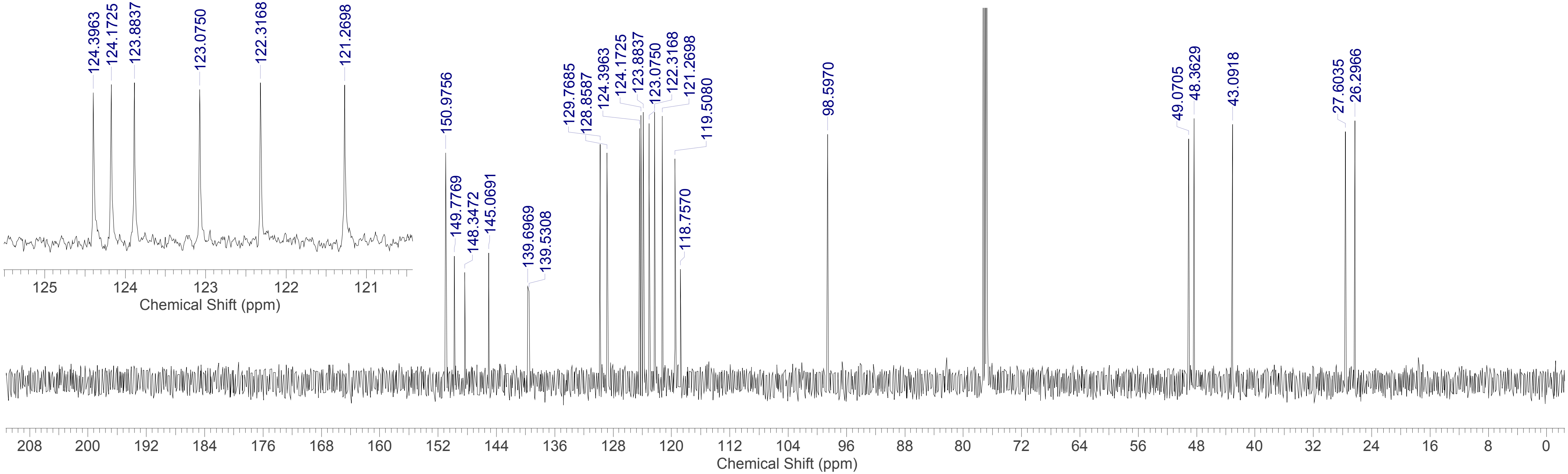

***N*-[(6-fluoro-1-benzothien-3-yl)methyl]-*N'*-quinolin-4-ylpropane-1,3-diamine (12)**

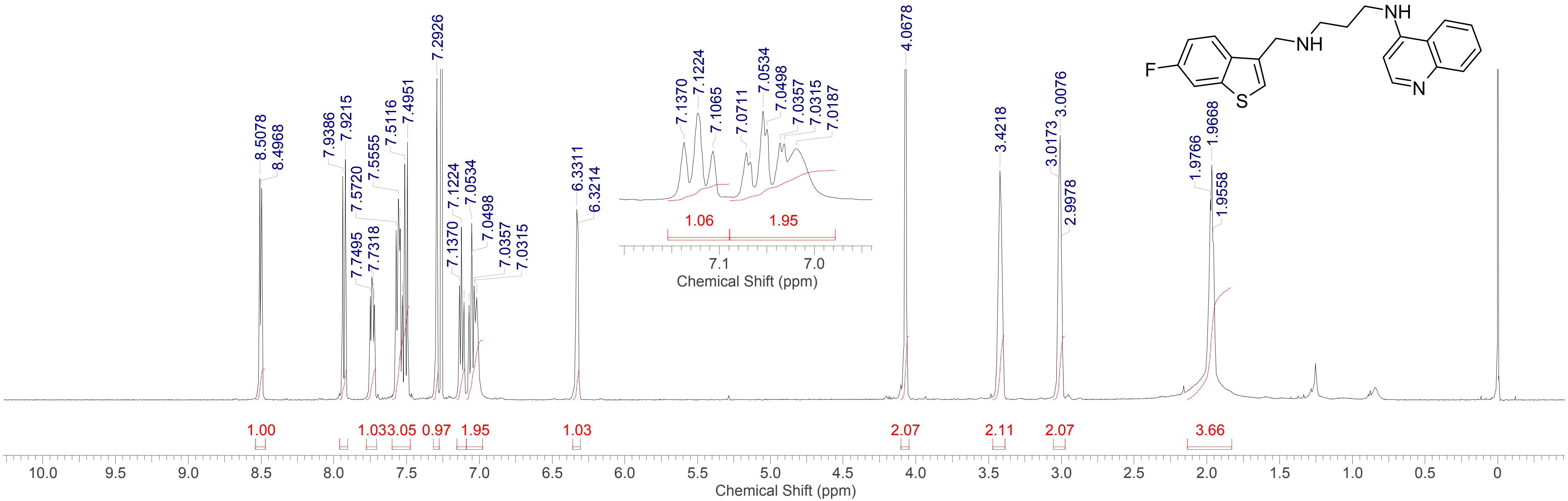

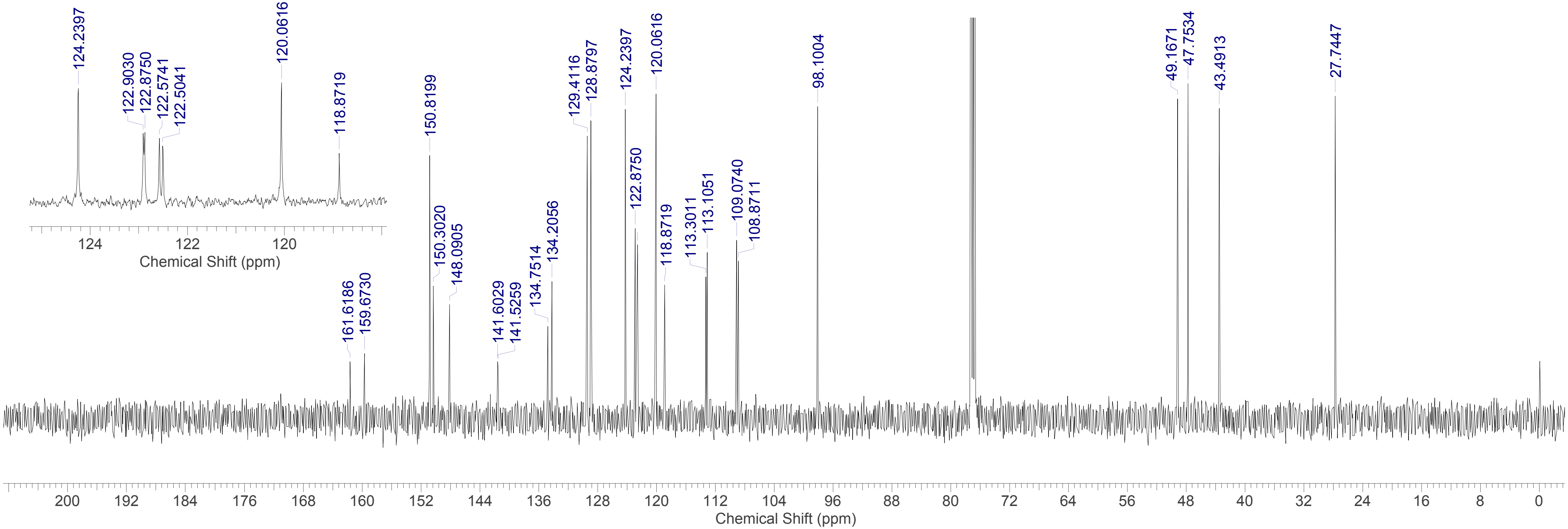

***N*-[(5-fluoro-1-benzothien-3-yl)methyl]-*N'*-quinolin-4-ylbutane-1,4-diamine (13)**

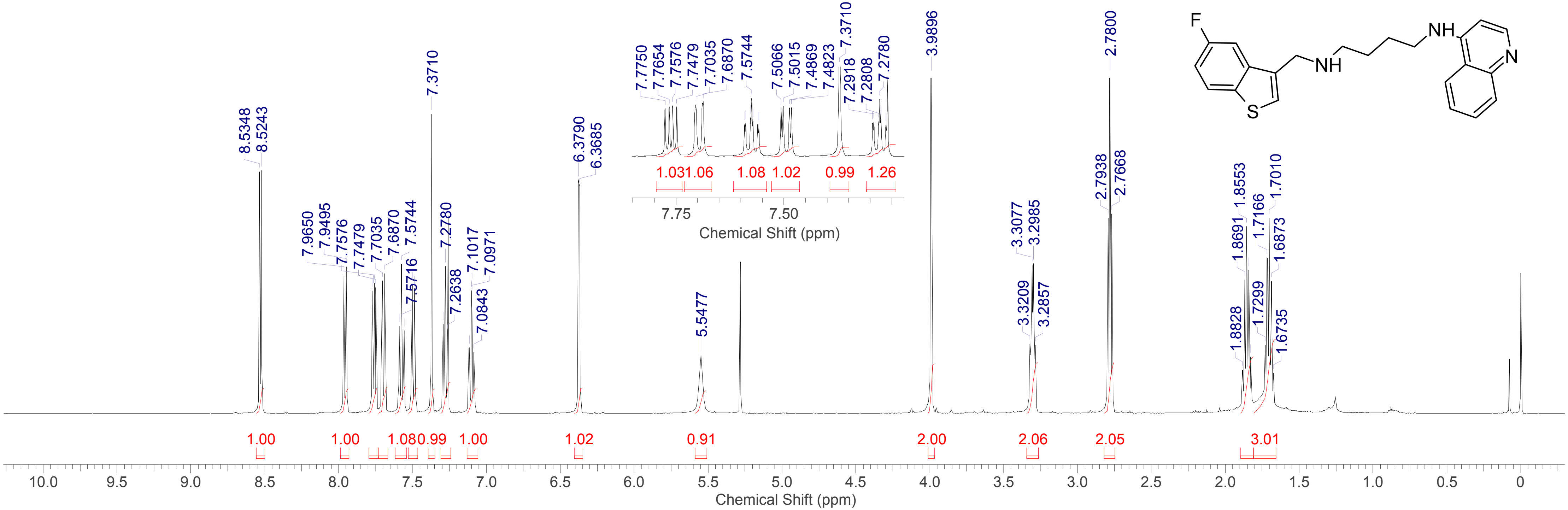

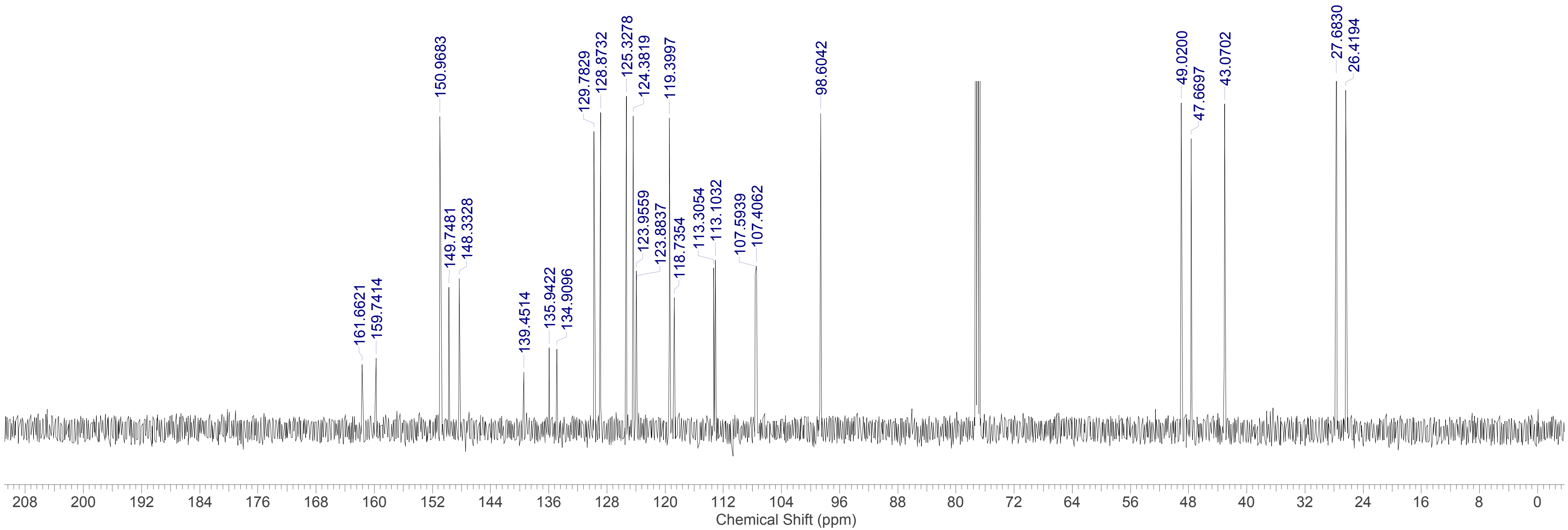

***N*-[4-(5-fluoro-1-benzothien-3-yl)benzyl]- *N'*-quinolin-4-ylpropane-1,3-diamine (23)**

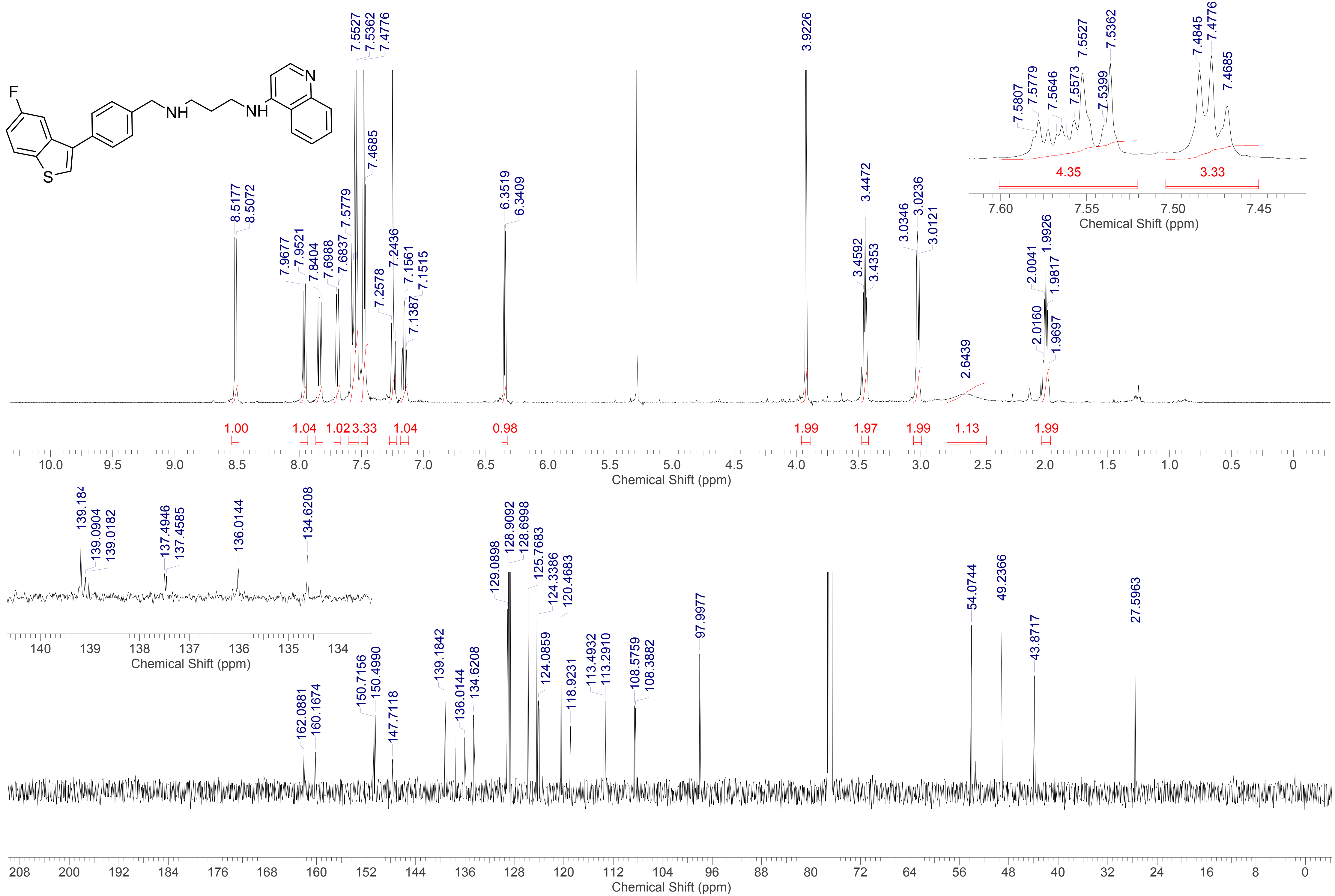

***N*-[4-(5-fluoro-1-benzothien-3-yl)benzyl]- *N'*-quinolin-4-ylhexane-1,6-diamine (24)**

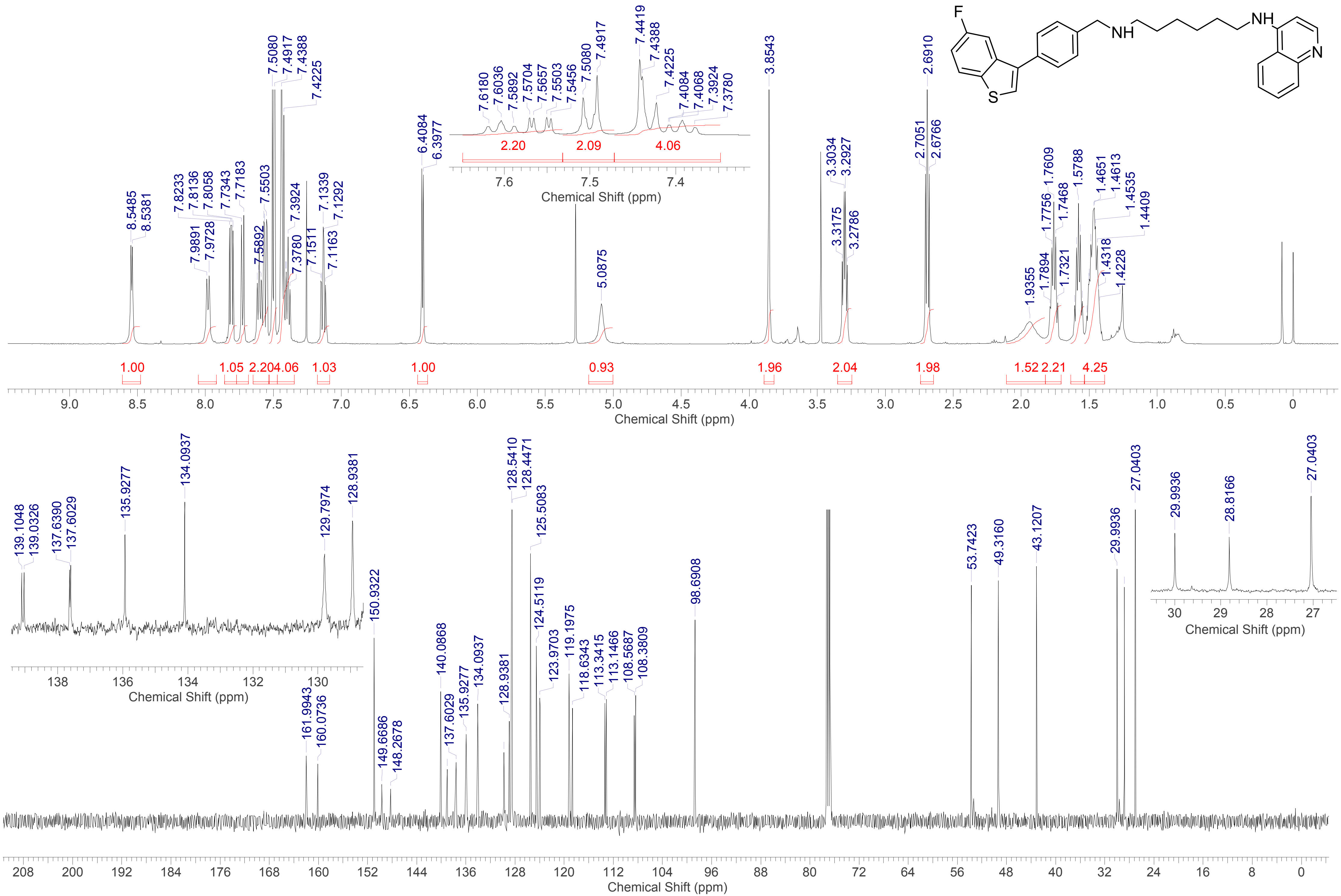

N^4-(7-chloroquinolin-4-yl)-N^1-[4-(5-fluoro-1-benzothien-3-yl)benzyl]pentane-1,4-diamine (25)

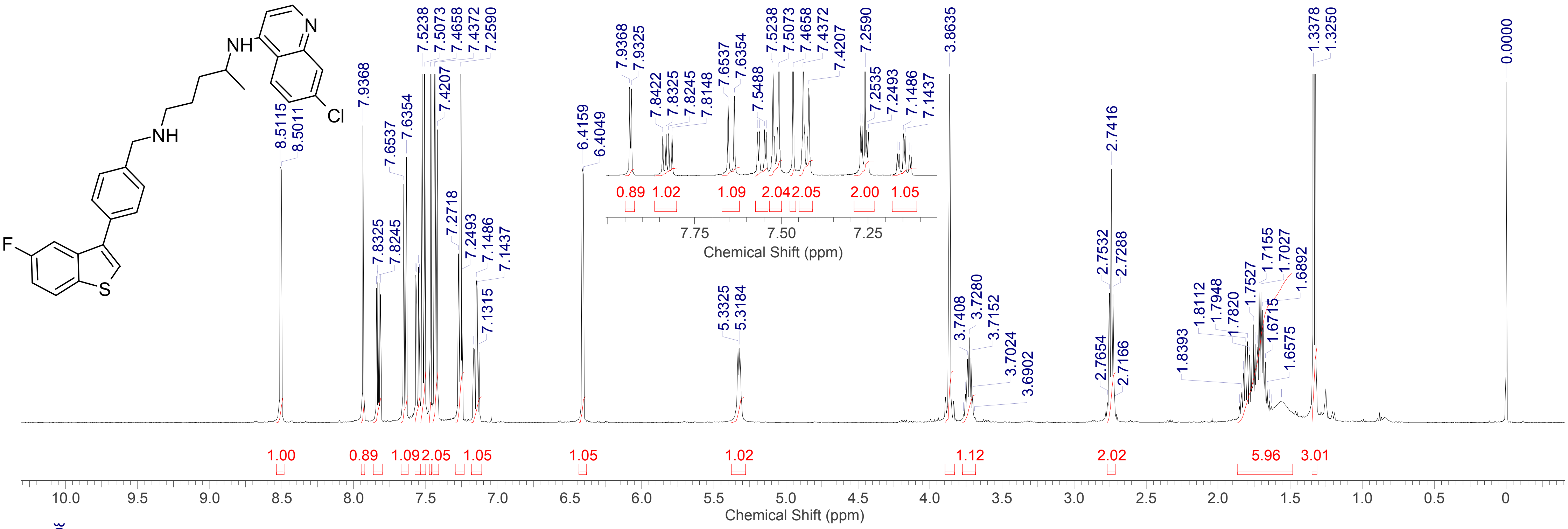

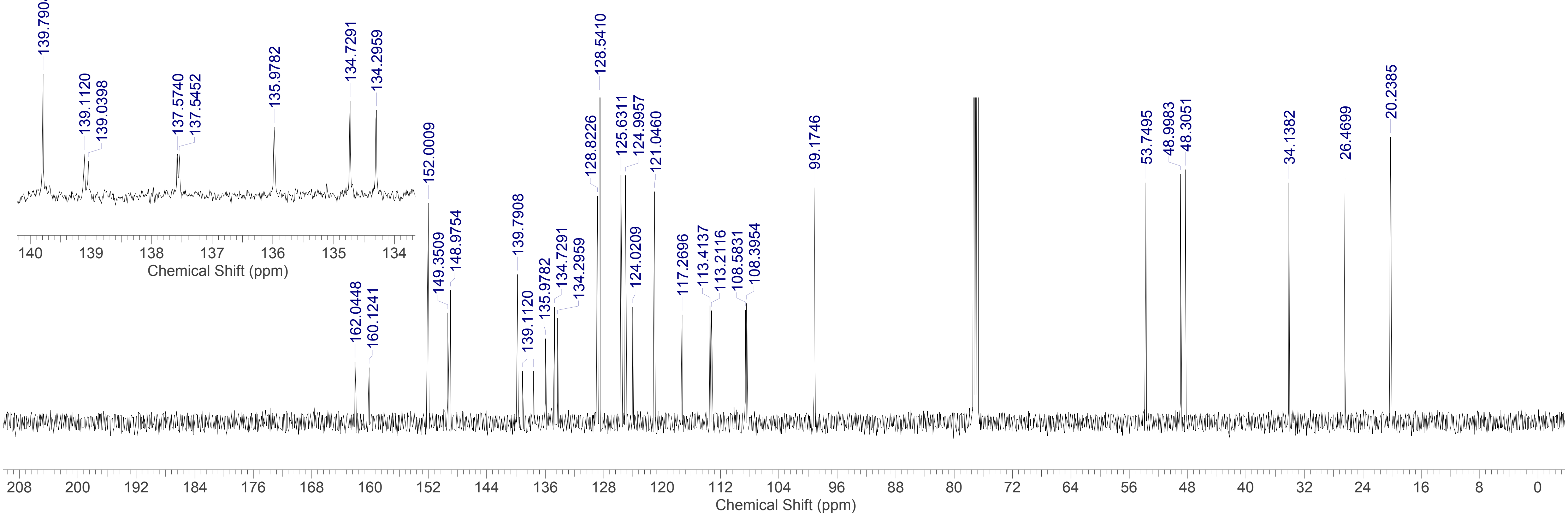

N^1-[4-(5-fluoro-1-benzothien-3-yl)benzyl]- N^4-quinolin-4-ylpentane-1,4-diamine (26)

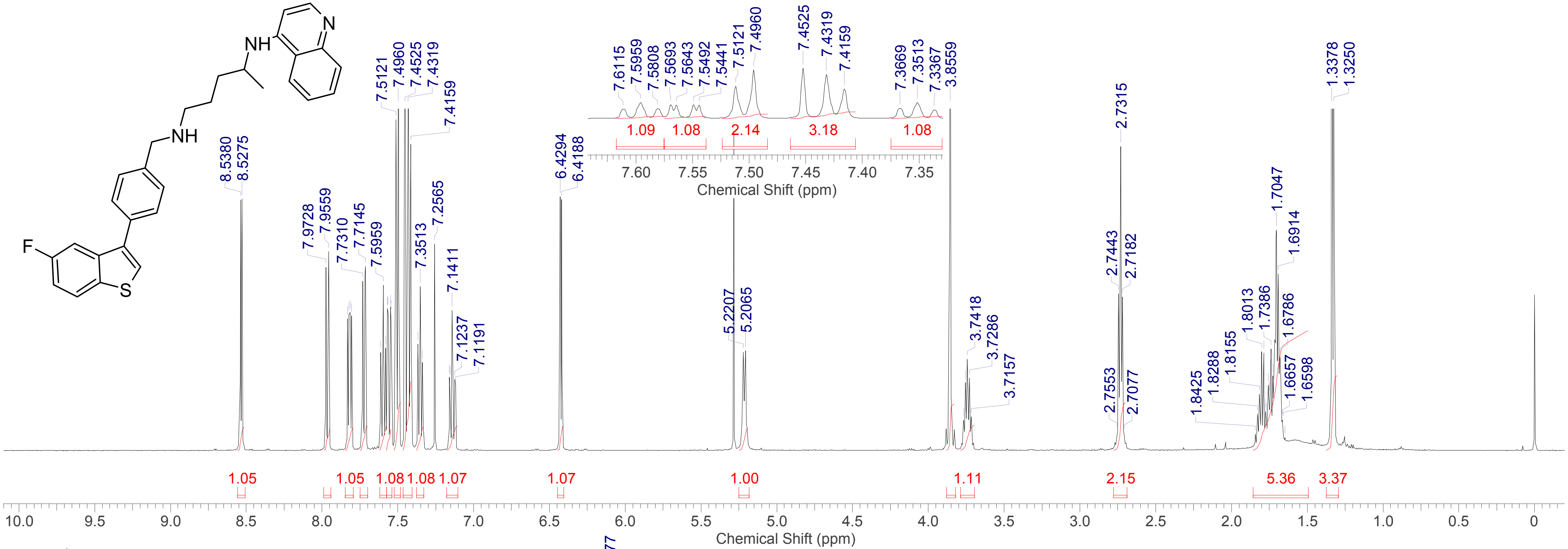

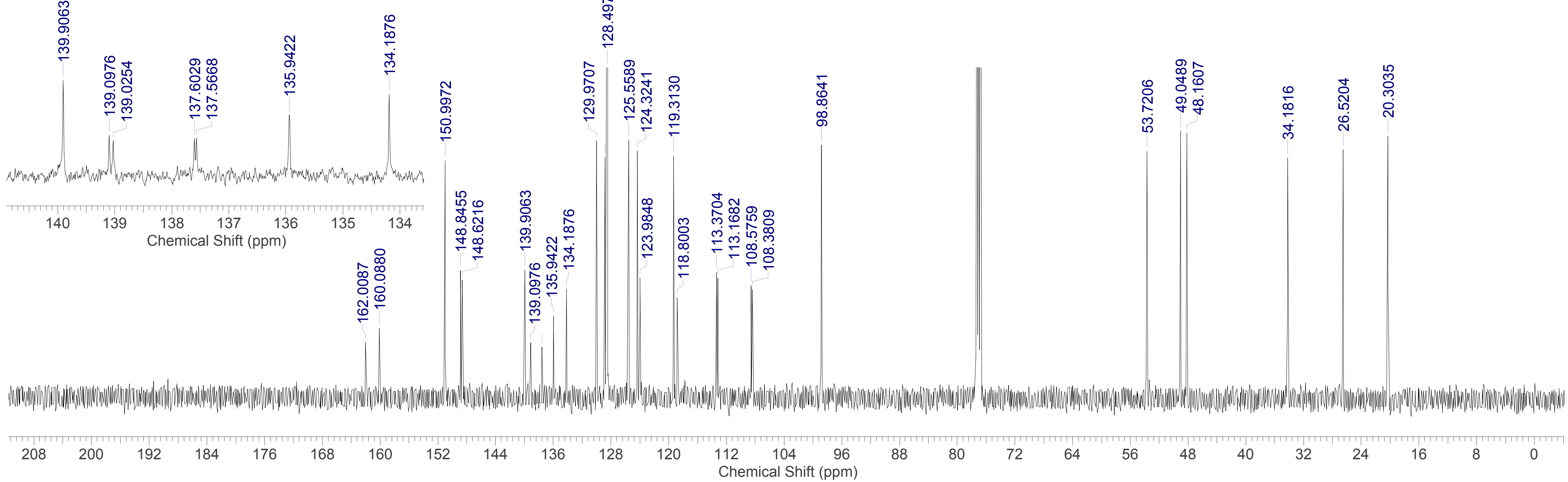

N[1]-[4-(5-fluoro-1-benzothien-3-yl)benzyl]- *N*[1]-methyl-*N*[4]-quinolin-4-ylpentane-1,4-diamine (27)

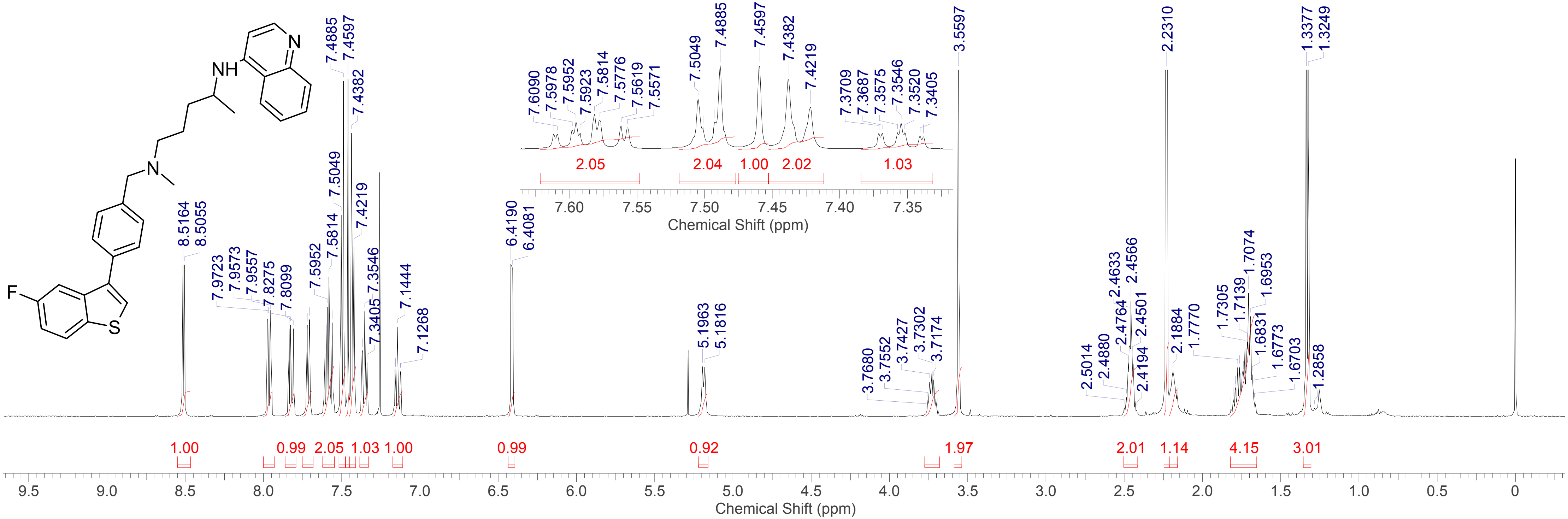

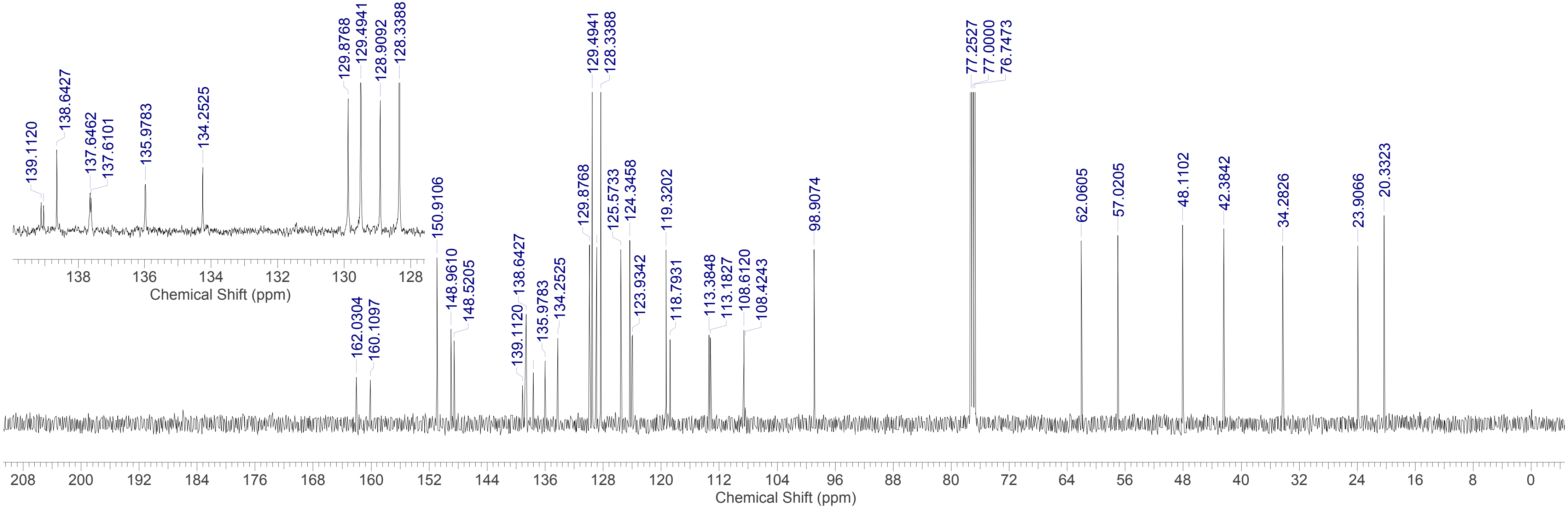

3-[4-({[4-(quinolin-4-ylamino)butyl]amino}methyl)phenyl]-1-benzothiophene-5-carbonitrile (28)

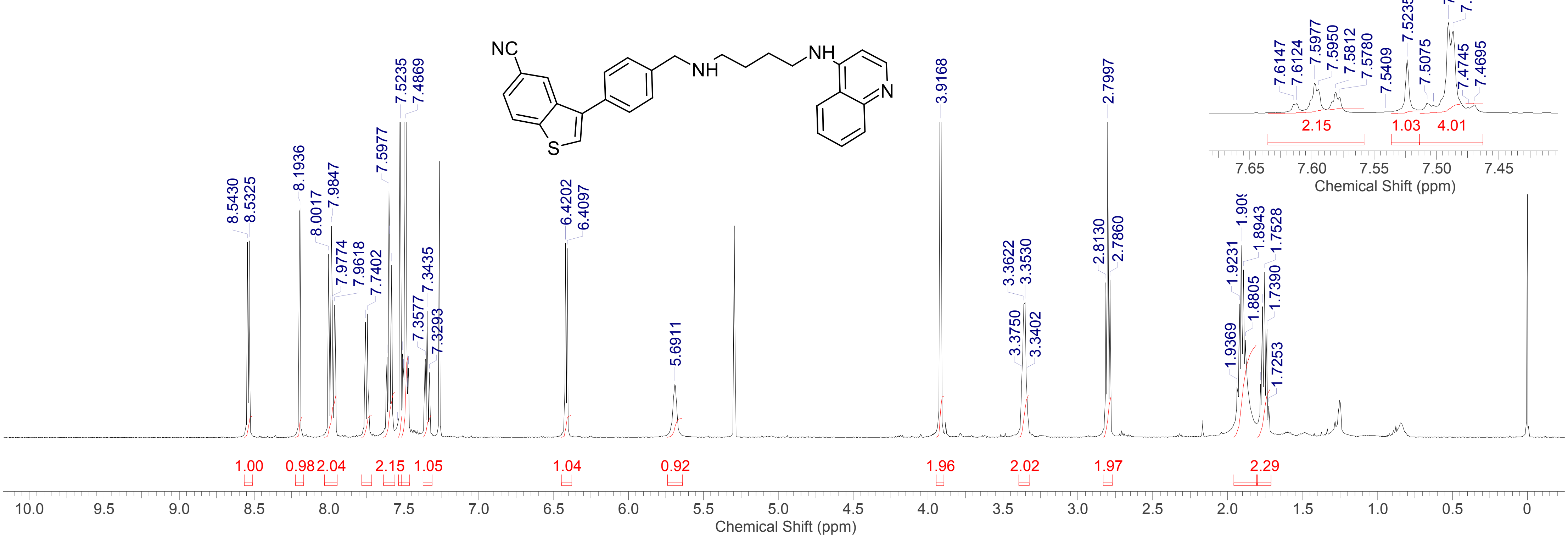

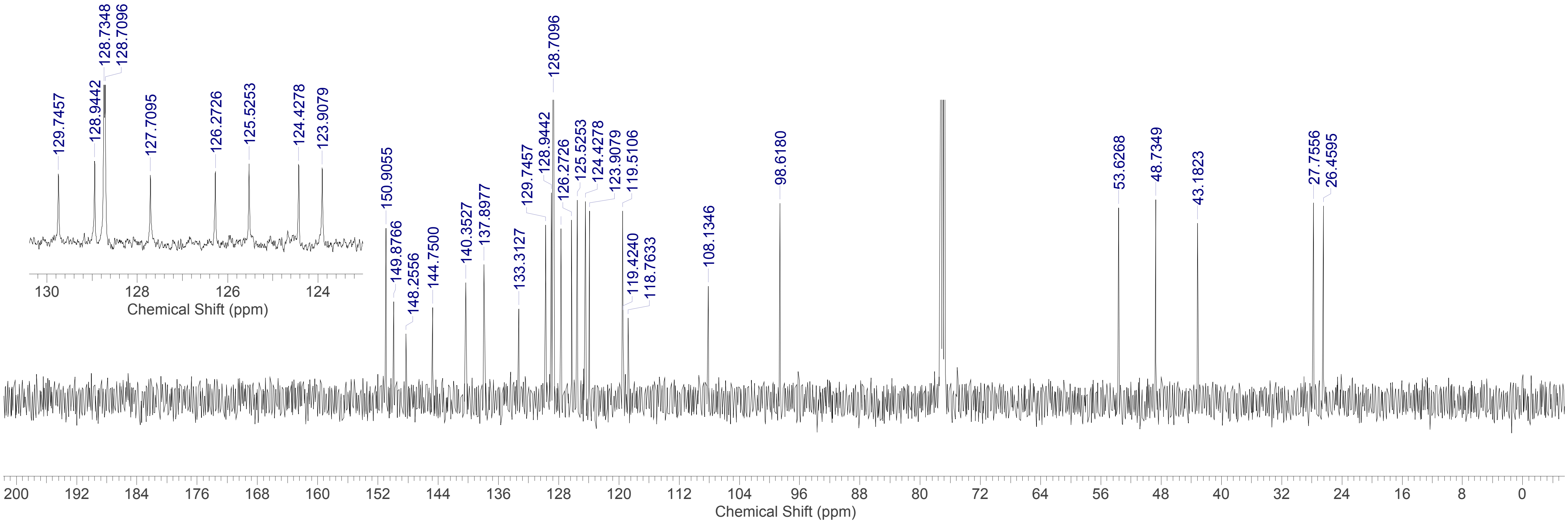

3-[4-({[4-(quinolin-4-ylamino)pentyl]amino}methyl)phenyl]-1-benzothiophene-5-carbonitrile (29)

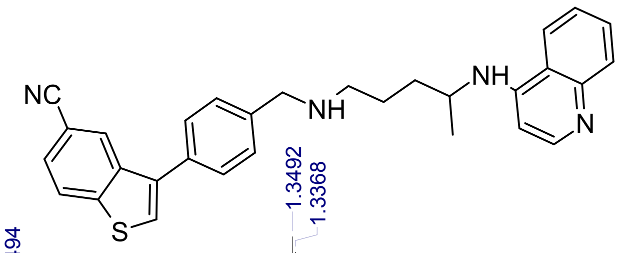

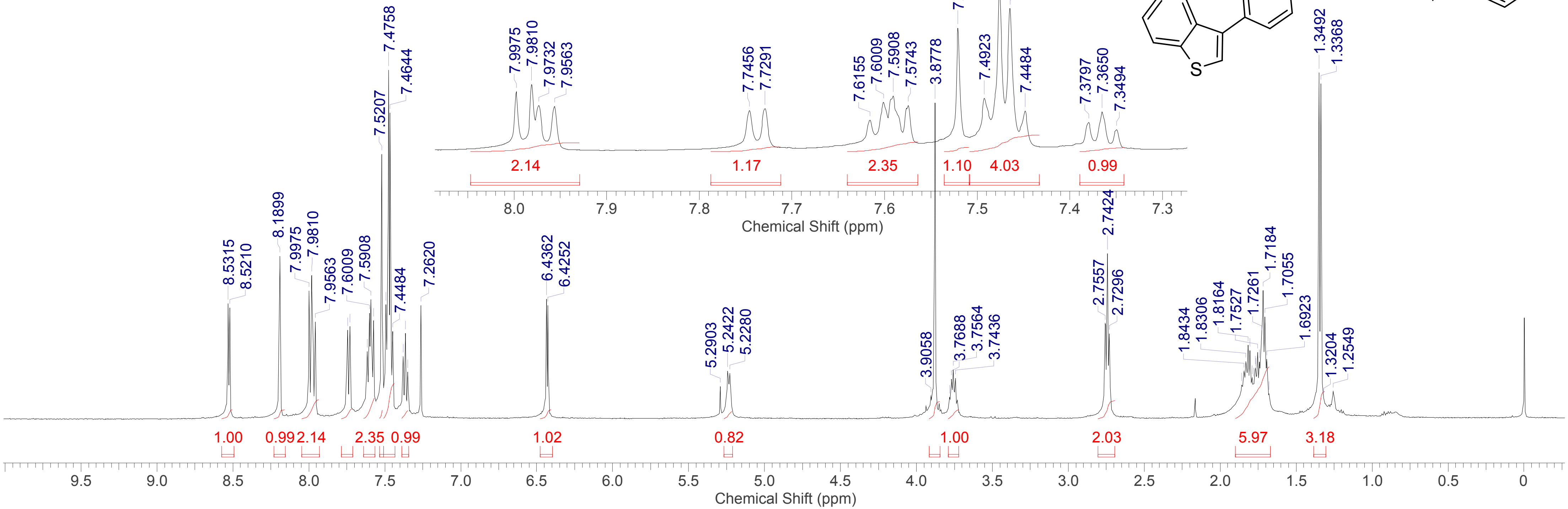

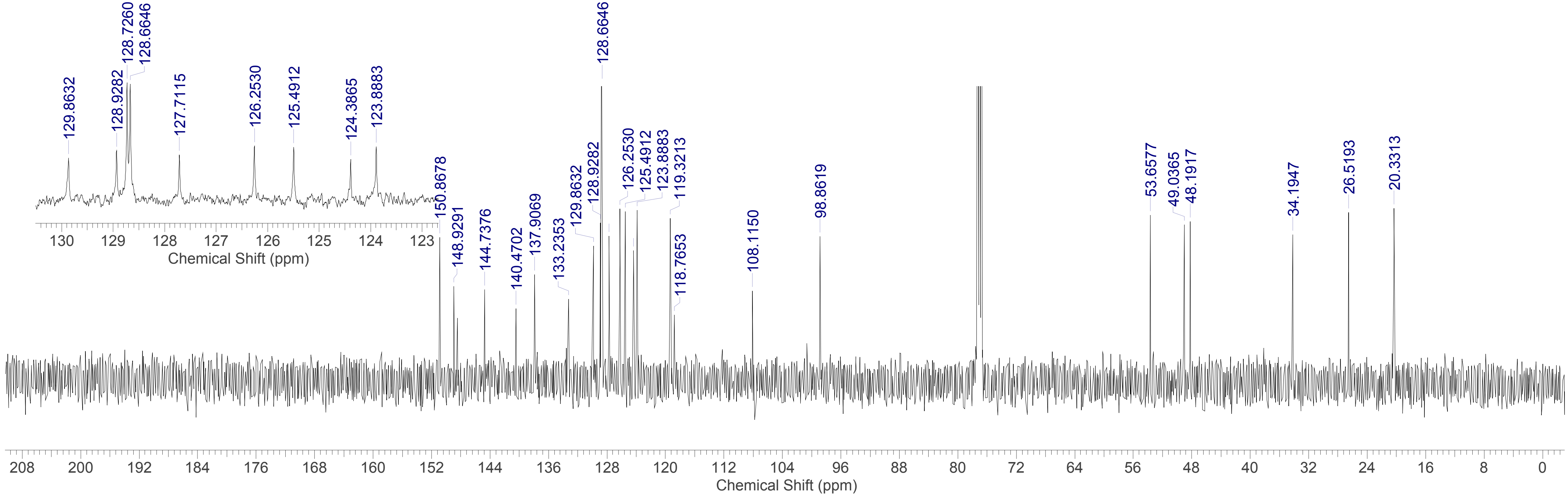

3-[4-({[6-(quinolin-4-ylamino)hexyl]amino}methyl)phenyl]-1-benzothiophene-5-carbonitrile (30)

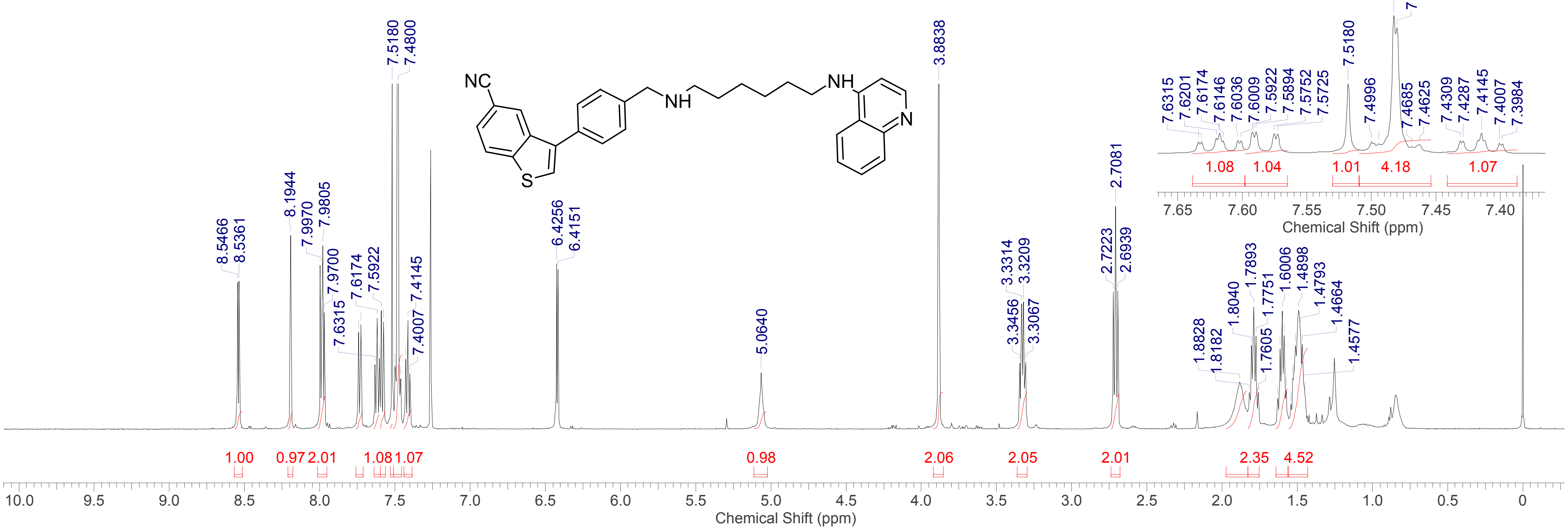

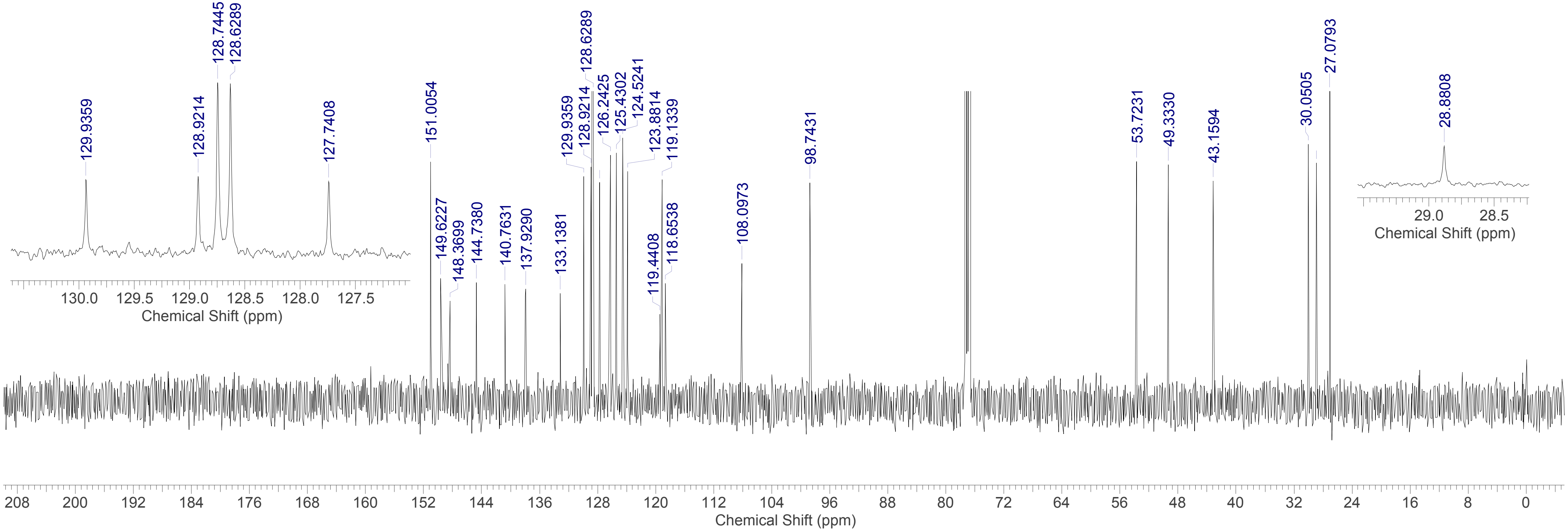

3-[4-({[4-(quinolin-4-ylamino)butyl]amino}methyl)phenyl]-1-benzothiophene-6-carbonitrile (31)

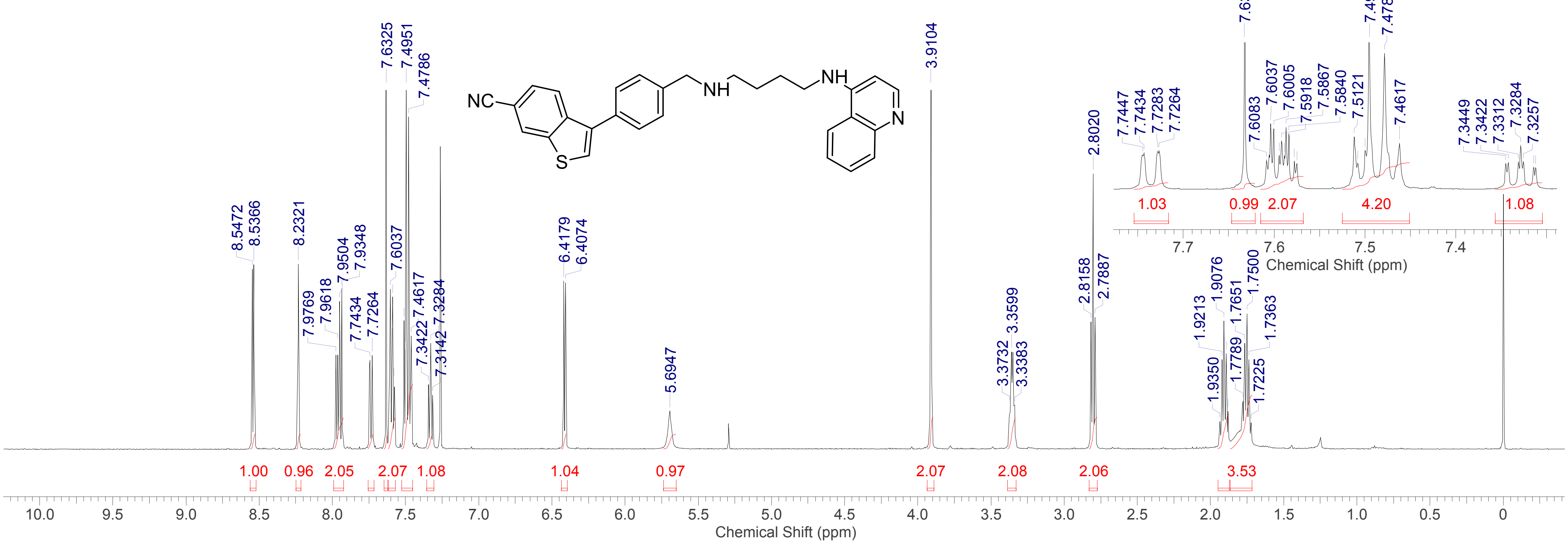

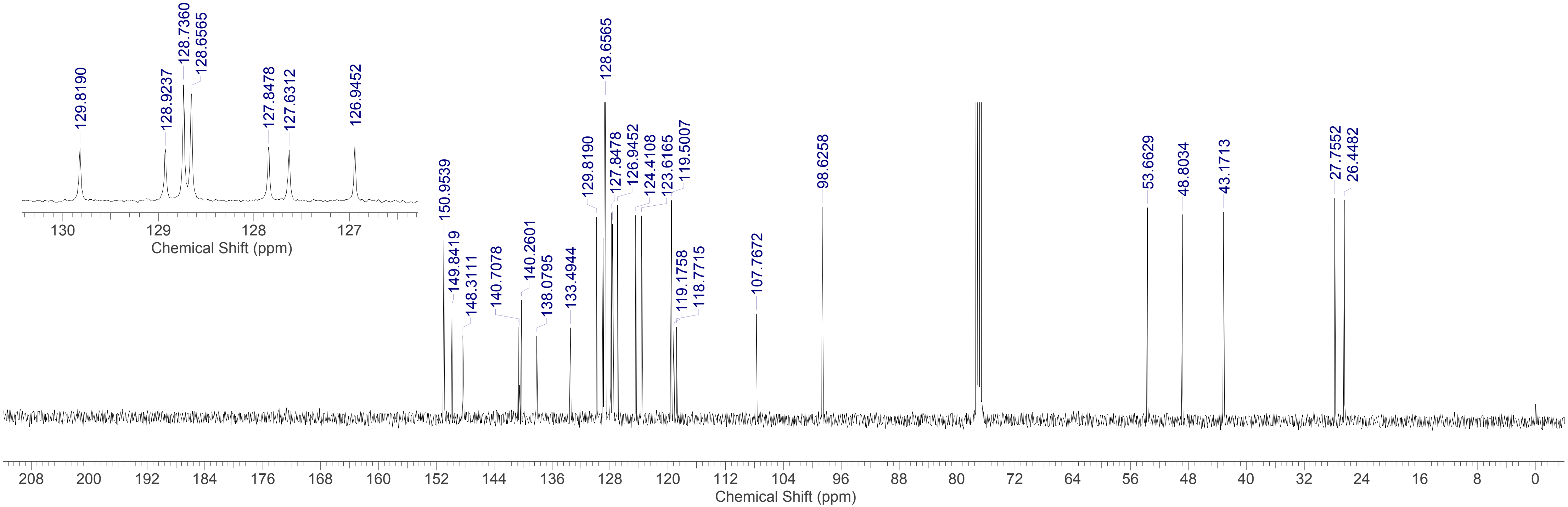

3-[4-({[6-(quinolin-4-ylamino)hexyl]amino}methyl)phenyl]-1-benzothiophene-6-carbonitrile (32)

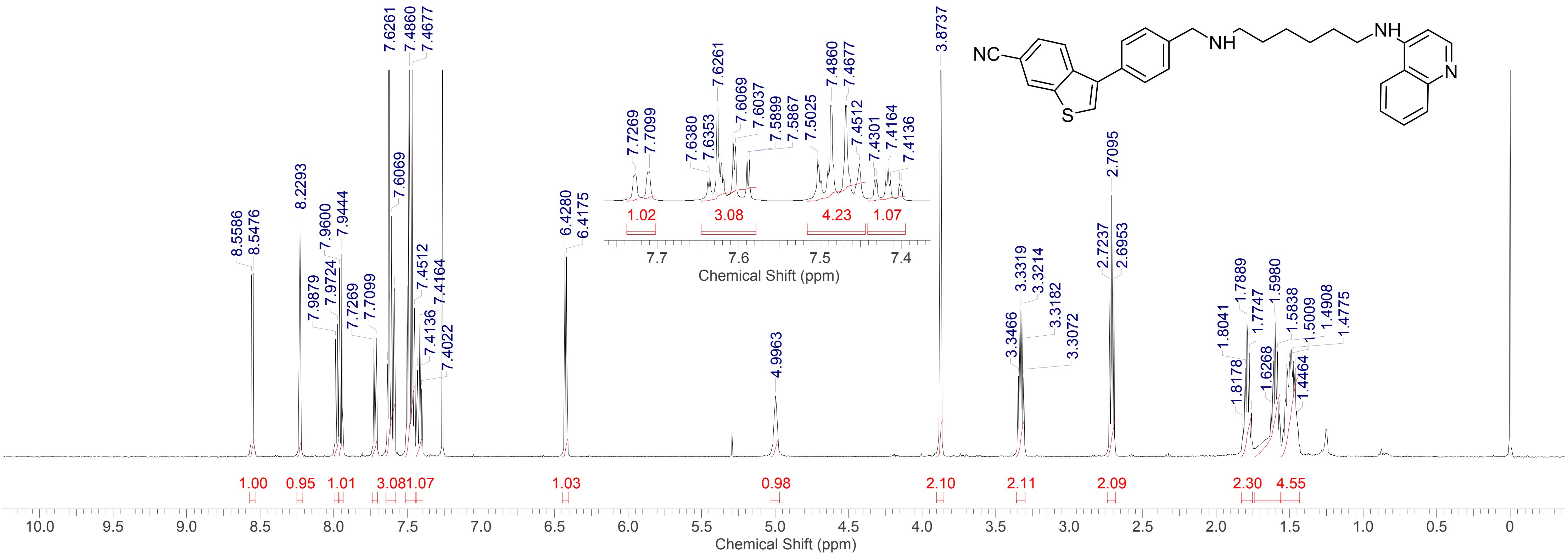

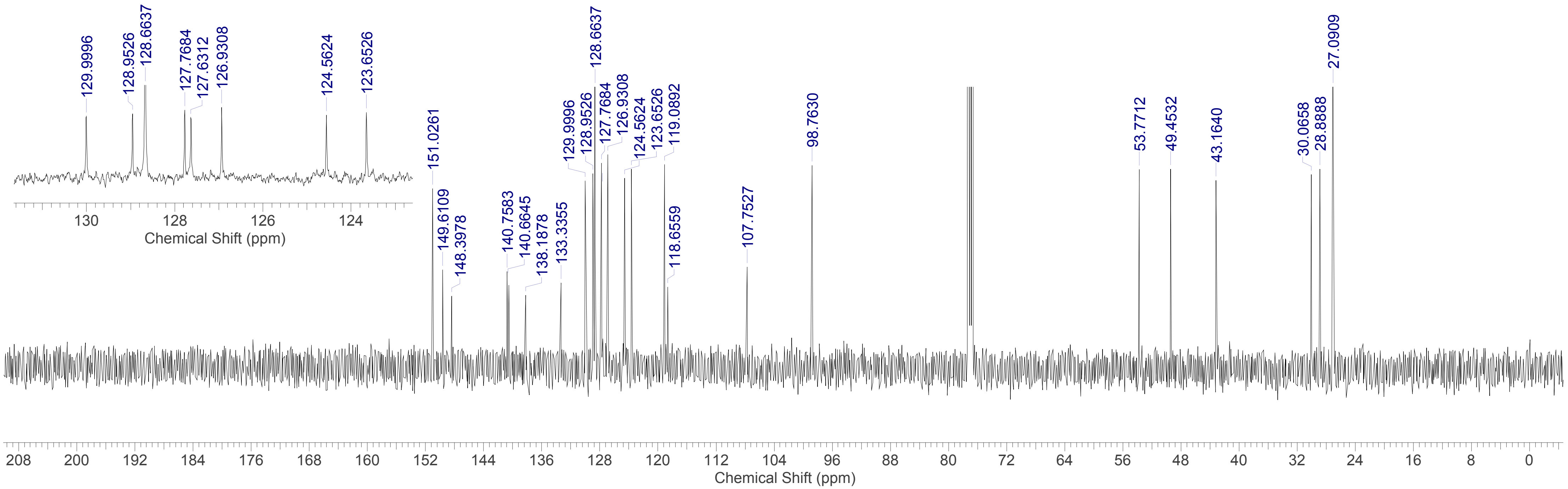

4-(5-{4-[({2-[(7-chloroquinolin-4-yl)amino]ethyl}amino)methyl]phenyl}-2-thienyl)benzonitrile (37)

MV146 St AQ2

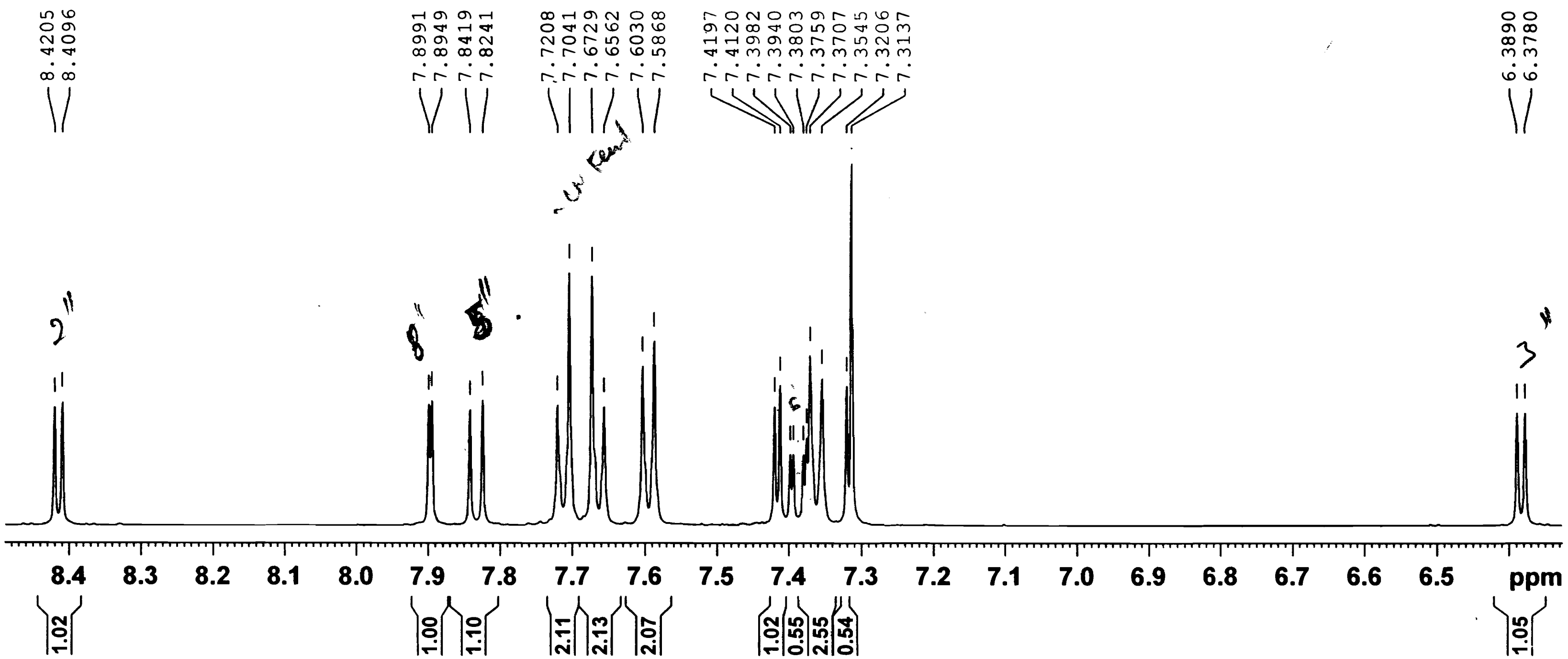

NAME	MVAA181
EXPNO	1
PROCNO	1
Date_	20111216
Time	9.43
INSTRUM	spect
PROBHD	5 mm BBO BB-1H
PULPROG	zg30
TD	32768
SOLVENT	CDCl3
NS	30
DS	0
SWH	5813.954 Hz
FIDRES	0.177428 Hz
AQ	2.8180981 sec
RG	575
DW	86.000 usec
DE	6.50 usec
TE	298.0 K
D1	2.00000000 sec
TD0	1
======== CHANNEL f1 ========	
NUC1	1H
P1	9.35 usec
PL1	0.00 dB
PL1W	27.37956238 W
SFO1	500.2625923 MHz
SI	16384
SF	500.2599881 MHz
WDW	EM
SSB	0
LB	0.30 Hz
GB	0
PC	1.00

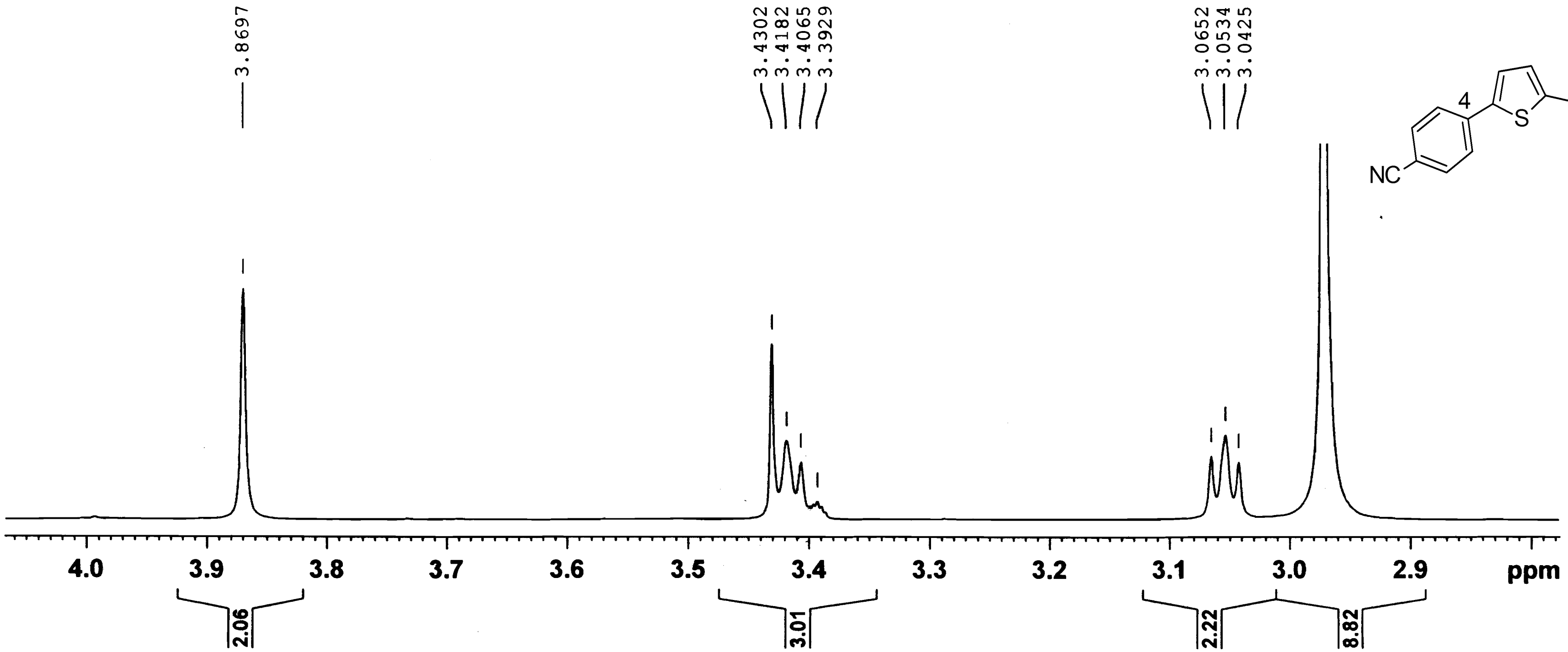

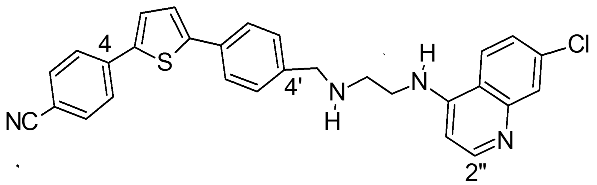

4-(5-{4-[({2-[(7-chloroquinolin-4-yl)amino]ethyl}amino)methyl]phenyl}-2-thienyl)benzonitrile (37)

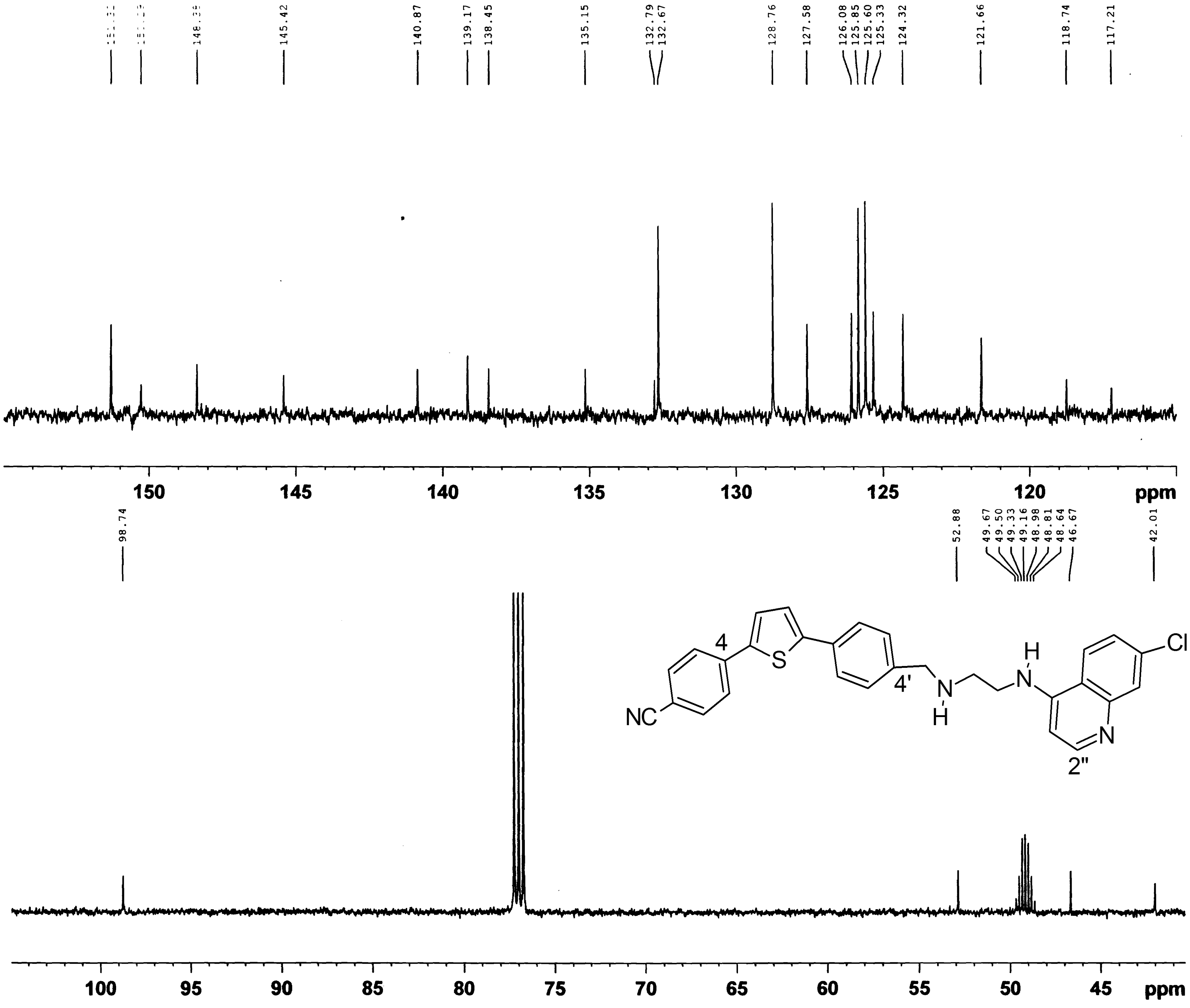

NAME	MVAA181
EXPNO	2
PROCNO	1
Date_	20111216
Time	9.48
INSTRUM	spect
PROBHD	5 mm BBO BB-1H
PULPROG	zgpg30
TD	32768
SOLVENT	CDCl3
NS	625
DS	4
SWH	29761.904 Hz
FIDRES	0.908261 Hz
AQ	0.5505524 sec
RG	1290
DW	16.800 usec
DE	6.50 usec
TE	298.1 K
D1	2.00000000 sec
D11	0.03000000 sec
TD0	1
======== CHANNEL f1 ========	
NUC1	13C
P1	11.50 usec
PL1	3.00 dB
PL1W	32.22848892 W
SFO1	125.8043140 MHz
======== CHANNEL f2 ========	
CPDPRG2	waltz16
NUC2	1H
PCPD2	80.00 usec
PL2	1.20 dB
PL12	18.40 dB
PL13	18.40 dB
PL2W	20.76952171 W
PL12W	0.39575511 W
PL13W	0.39575511 W
SFO2	500.2620010 MHz
SI	32768
SF	125.7904878 MHz
WDW	EM
SSB	0
LB	1.50 Hz
GB	0
PC	1.40

4-(5-{4-[({3-[(7-chloroquinolin-4-yl)amino]propyl}amino)methyl]phenyl}-2-thienyl)benzonitrile (38)

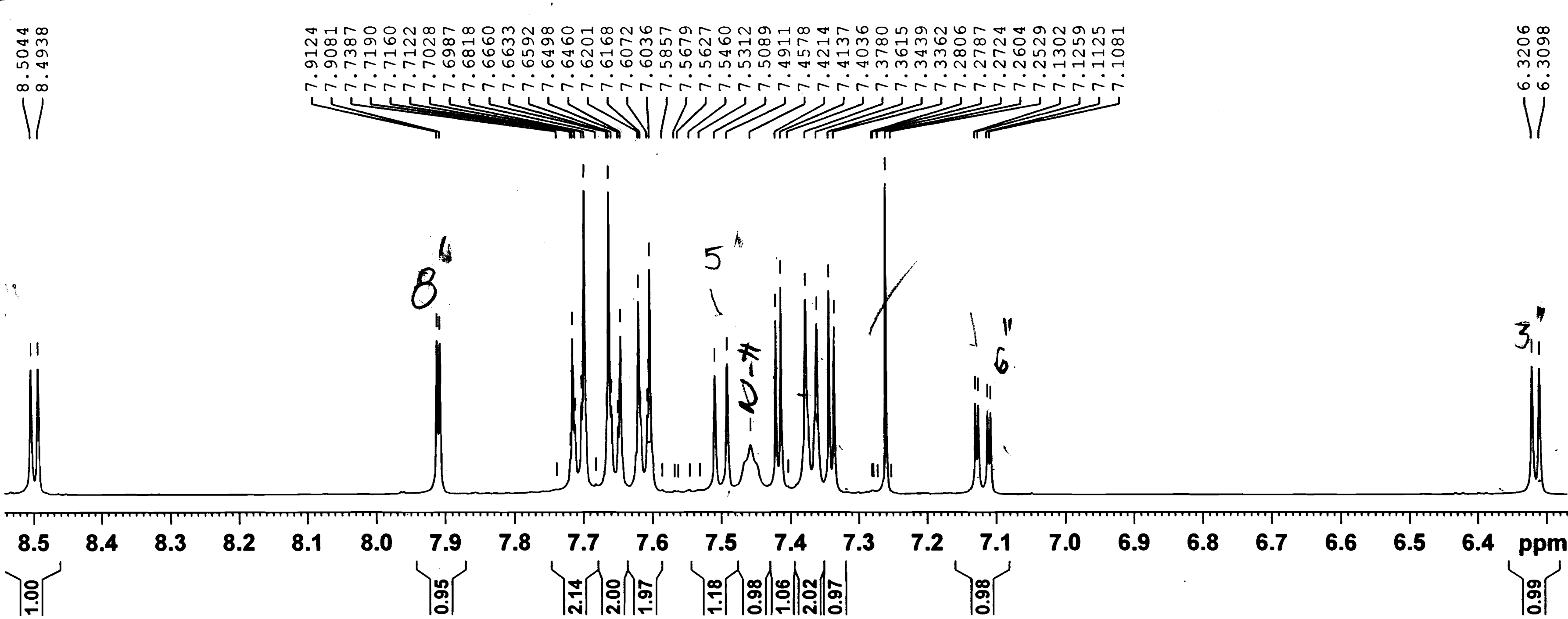

NAME	MVAA210a
EXPNO	1
PROCNO	1
Date_	20120315
Time	8.16
INSTRUM	spect
PROBHD	5 mm BBO BB-1H
PULPROG	zg30
TD	32768
SOLVENT	CDCl3
NS	16
DS	0
SWH	6487.889 Hz
FIDRES	0.197995 Hz
AQ	2.5253706 sec
RG	322
DW	77.067 use
DE	6.50 use
TE	298.0 K
D1	2.00000000 sec
TD0	1

======== CHANNEL f1 ========

NUC1	1H
P1	9.35 use
PL1	0.00 dB
PL1W	27.37956238 W
SFO1	500.2627938 MHz
SI	32768
SF	500.2600143 MHz
WDW	EM
SSB	0
LB	0.20 Hz
GB	0
PC	1.00

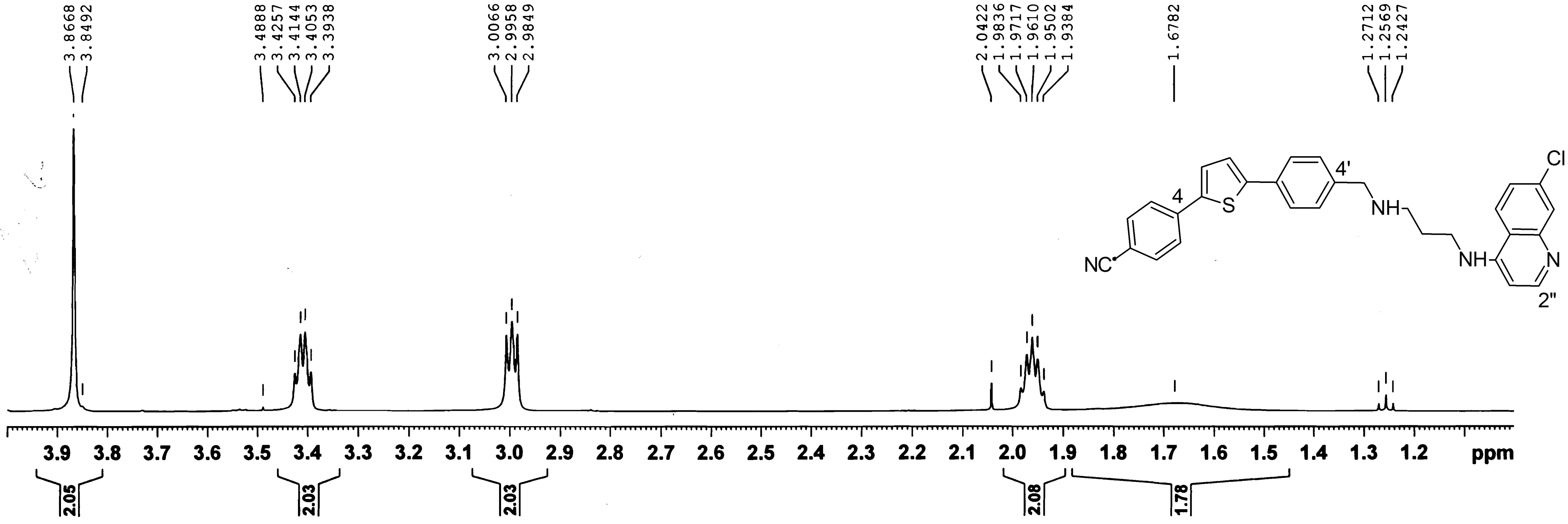

4-(5-{4-[({3-[(7-chloroquinolin-4-yl)amino]propyl}amino)methyl]phenyl}-2-thienyl)benzonitrile (38)

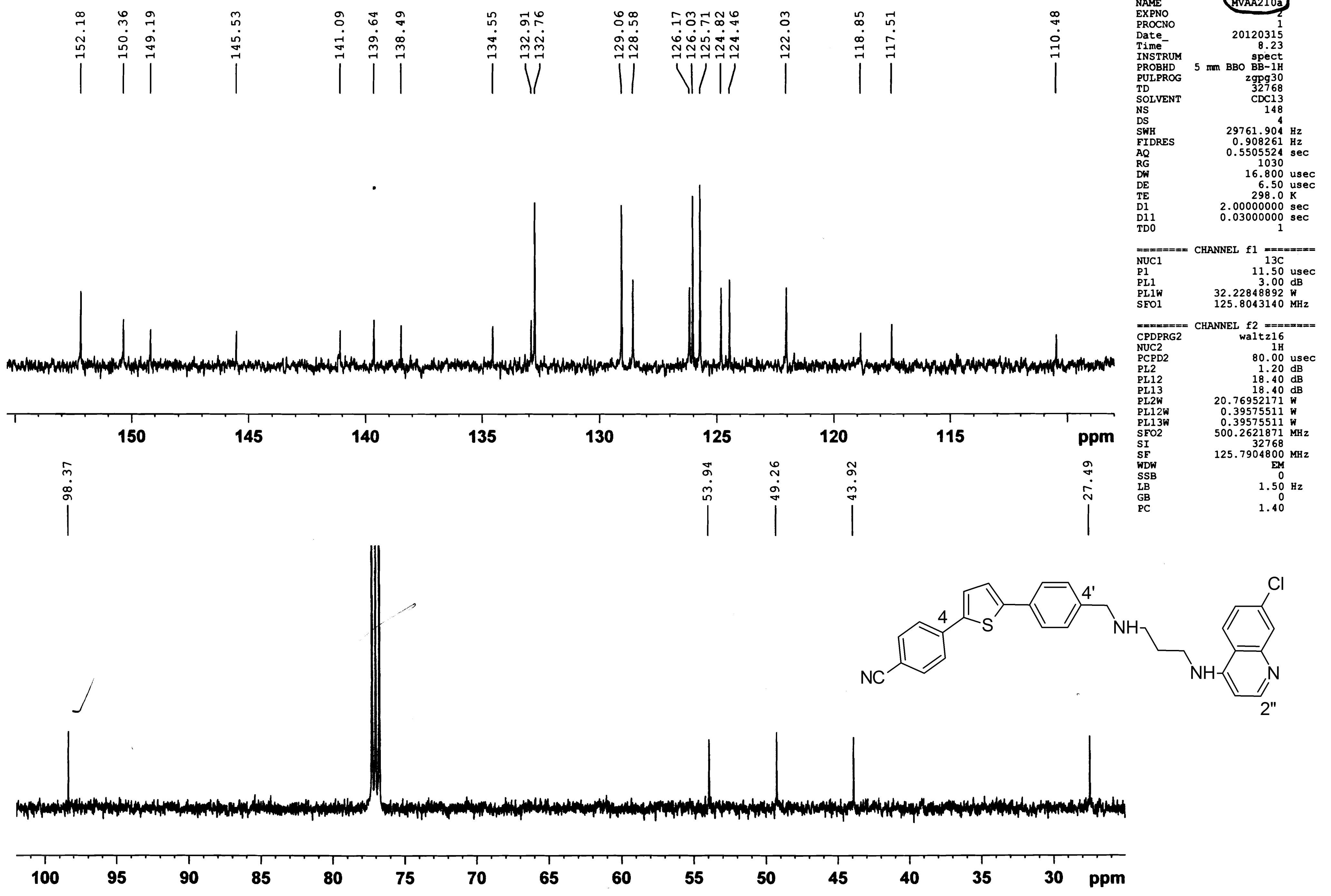

4-(5-{4-[({4-[(7-chloroquinolin-4-yl)amino]butyl}amino)methyl]phenyl}-2-thienyl)benzonitrile (39)

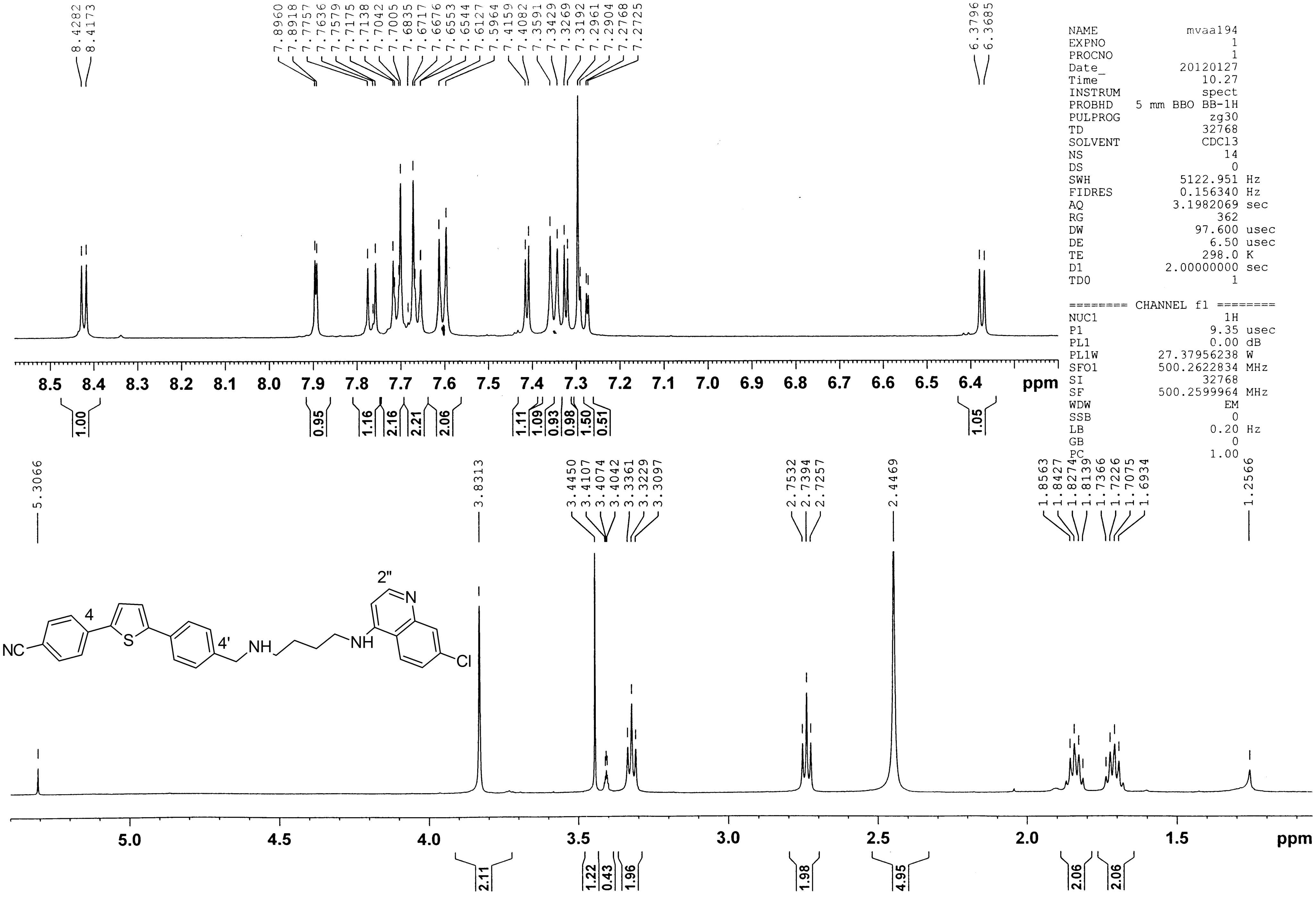

4-(5-{4-[({4-[(7-chloroquinolin-4-yl)amino]butyl}amino)methyl]phenyl}-2-thienyl)benzonitrile (39)

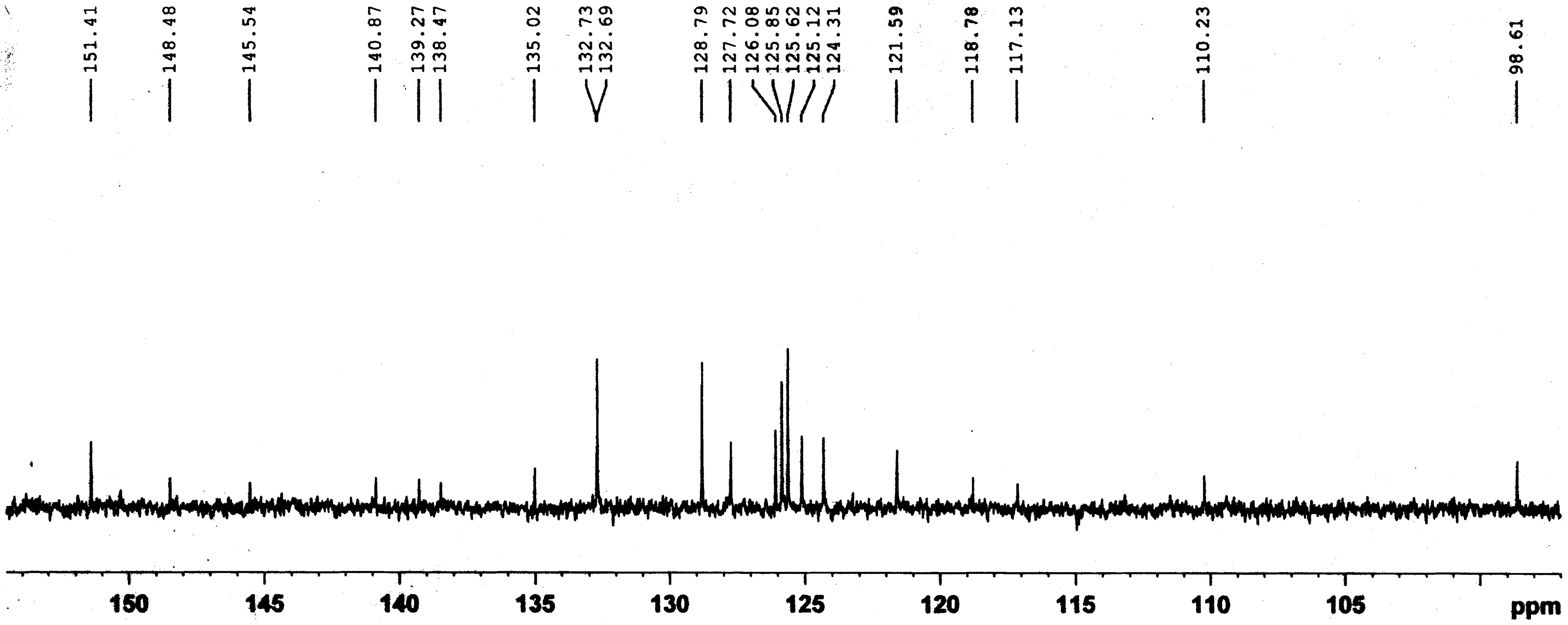

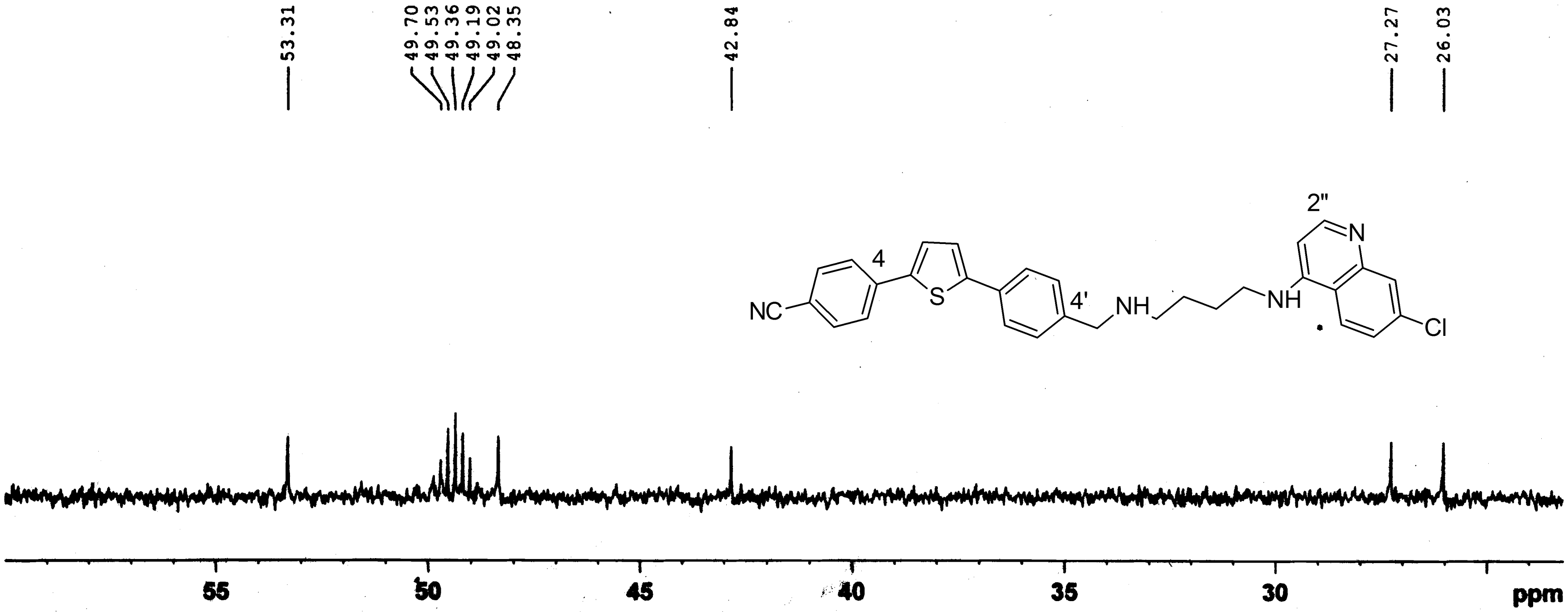

NAME	mvaa194
EXPNO	6
PROCNO	1
Date_	20120127
Time	10.35
INSTRUM	spect
PROBHD	5 mm BBO BB-1H
PULPROG	zgpg30
TD	32768
SOLVENT	CDCl3
NS	221
DS	4
SWH	29761.904 Hz
FIDRES	0.908261 Hz
AQ	0.5505524 sec
RG	1030
DW	16.800 usec
DE	6.50 usec
TE	298.0 K
D1	2.00000000 sec
D11	0.03000000 sec
TD0	1
======== CHANNEL f1 ========	
NUC1	13C
P1	11.50 usec
PL1	3.00 dB
PL1W	32.22848892 W
SFO1	125.8043140 MHz
======== CHANNEL f2 ========	
CPDPRG2	waltz16
NUC2	1H
PCPD2	80.00 usec
PL2	1.20 dB
PL12	18.40 dB
PL13	18.40 dB
PL2W	20.76952171 W
PL12W	0.39575511 W
PL13W	0.39575511 W
SFO2	500.2619175 MHz
SI	32768
SF	125.7904862 MHz
WDW	EM
SSB	0
LB	1.50 Hz
GB	0
PC	1.40

4-(5-{4-[({5-[(7-chloroquinolin-4-yl)amino]pentyl}amino)methyl]phenyl}-2-thienyl)benzonitrile (40)

MV151 sa AQ5

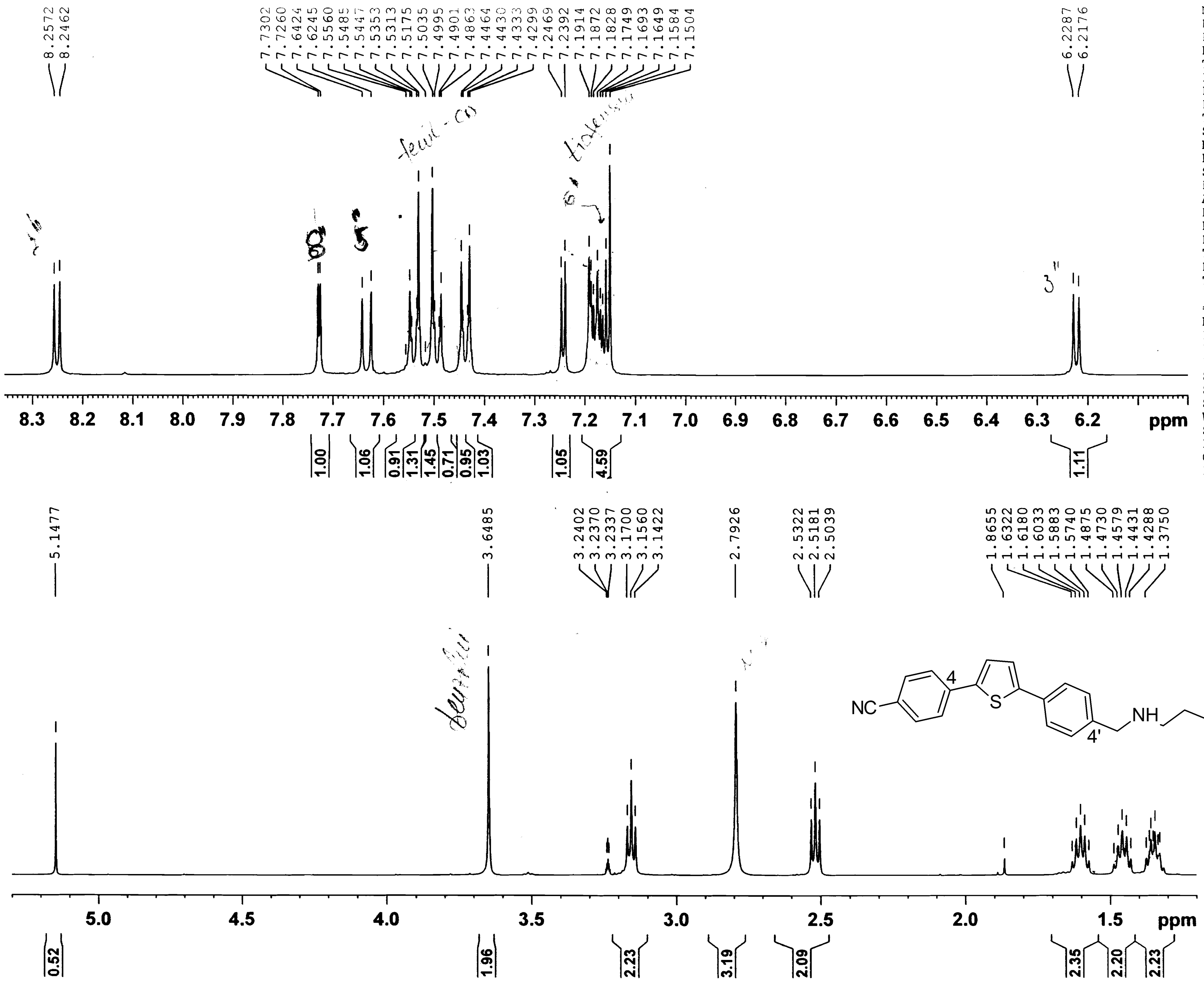

NAME	MVAA208
EXPNO	1
PROCNO	1
Date_	20120312
Time	10.00
INSTRUM	spect
PROBHD	5 mm BBO BB-1H
PULPROG	zg30
TD	32768
SOLVENT	CDCl3
NS	16
DS	0
SWH	5555.556 Hz
FIDRES	0.169542 Hz
AQ	2.9491701 sec
RG	322
DW	90.000 usec
DE	6.50 usec
TE	298.0 K
D1	2.00000000 sec
TD0	1
======== CHANNEL f1 ========	
NUC1	1H
P1	9.35 usec
PL1	0.00 dB
PL1W	27.37956238 W
SFO1	500.2623221 MHz
SI	32768
SF	500.2600689 MHz
WDW	EM
SSB	0
LB	0.20 Hz
GB	0
PC	1.00

4-(5-{4-[({5-[(7-chloroquinolin-4-yl)amino]pentyl}amino)methyl]phenyl}-2-thienyl)benzonitrile (40)

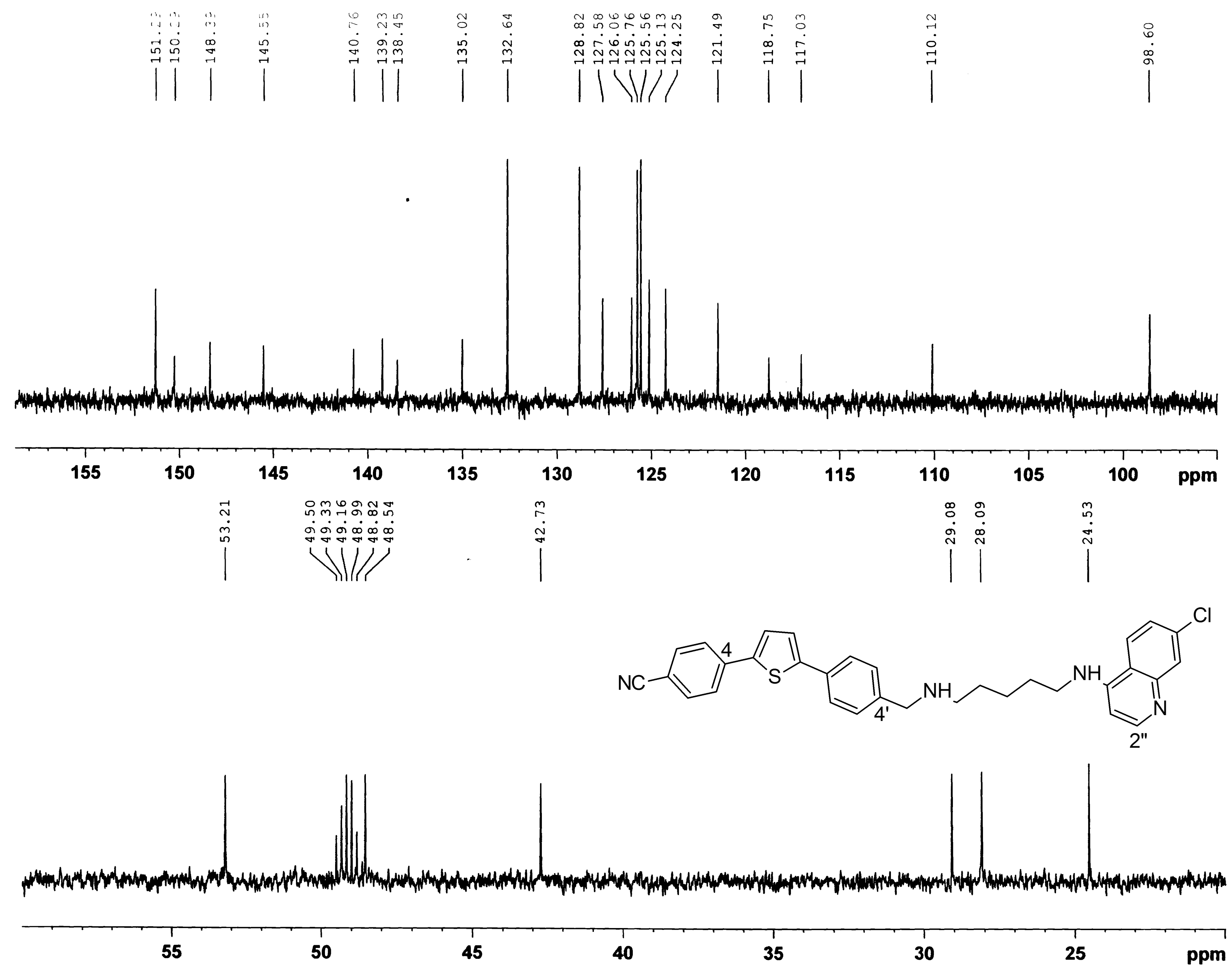

NAME	MVAA208
EXPNO	2
PROCNO	1
Date_	20120312
Time	10.04
INSTRUM	spect
PROBHD	5 mm BBO BB-1H
PULPROG	zgpg30
TD	32768
SOLVENT	CDCl3
NS	100
DS	4
SWH	29761.904 Hz
FIDRES	0.908261 Hz
AQ	0.5505524 sec
RG	1030
DW	16.800 usec
DE	6.50 usec
TE	298.0 K
D1	2.00000000 sec
D11	0.03000000 sec
TD0	1
======== CHANNEL f1 ========	
NUC1	13C
P1	11.50 usec
PL1	3.00 dB
PL1W	32.22848892 W
SFO1	125.8030560 MHz
======== CHANNEL f2 ========	
CPDPRG2	waltz16
NUC2	1H
PCPD2	80.00 usec
PL2	1.20 dB
PL12	18.40 dB
PL13	18.40 dB
PL2W	20.76952171 W
PL12W	0.39575511 W
PL13W	0.39575511 W
SFO2	500.2621271 MHz
SI	32768
SF	125.7904891 MHz
WDW	EM
SSB	0
LB	1.50 Hz
GB	0
PC	1.40

4-(5-{4-[({6-[(7-chloroquinolin-4-yl)amino]hexyl}amino)methyl]phenyl}-2-thienyl)benzonitrile (41)

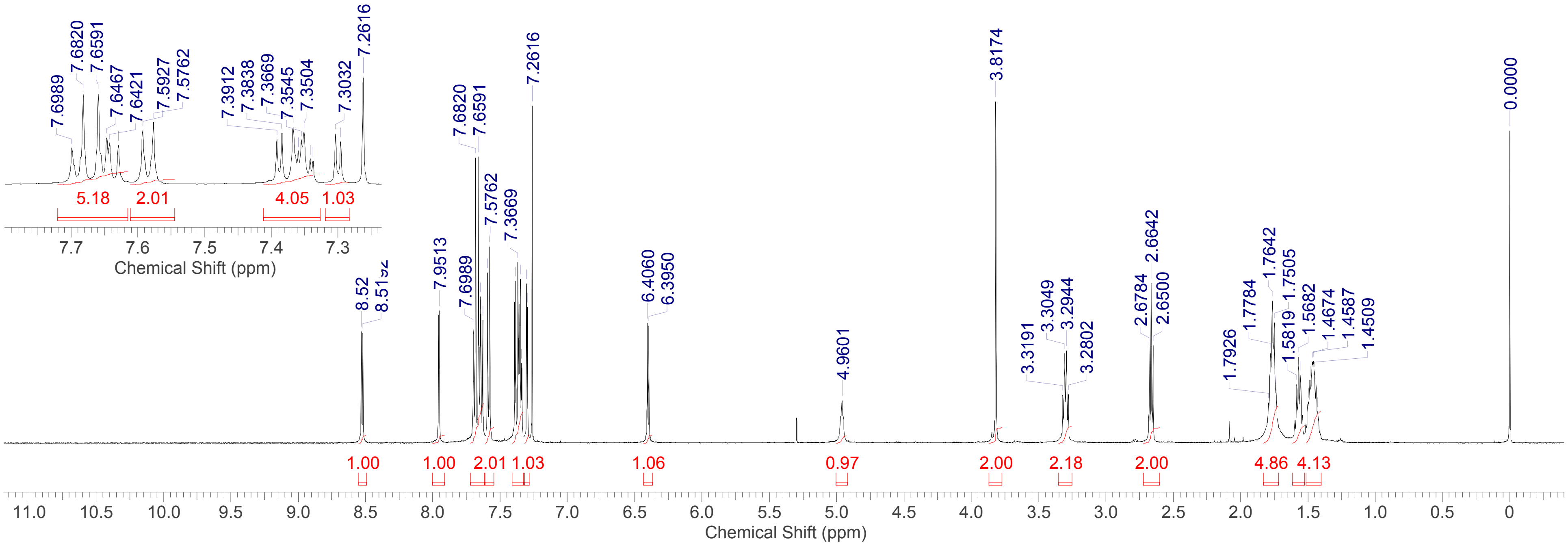

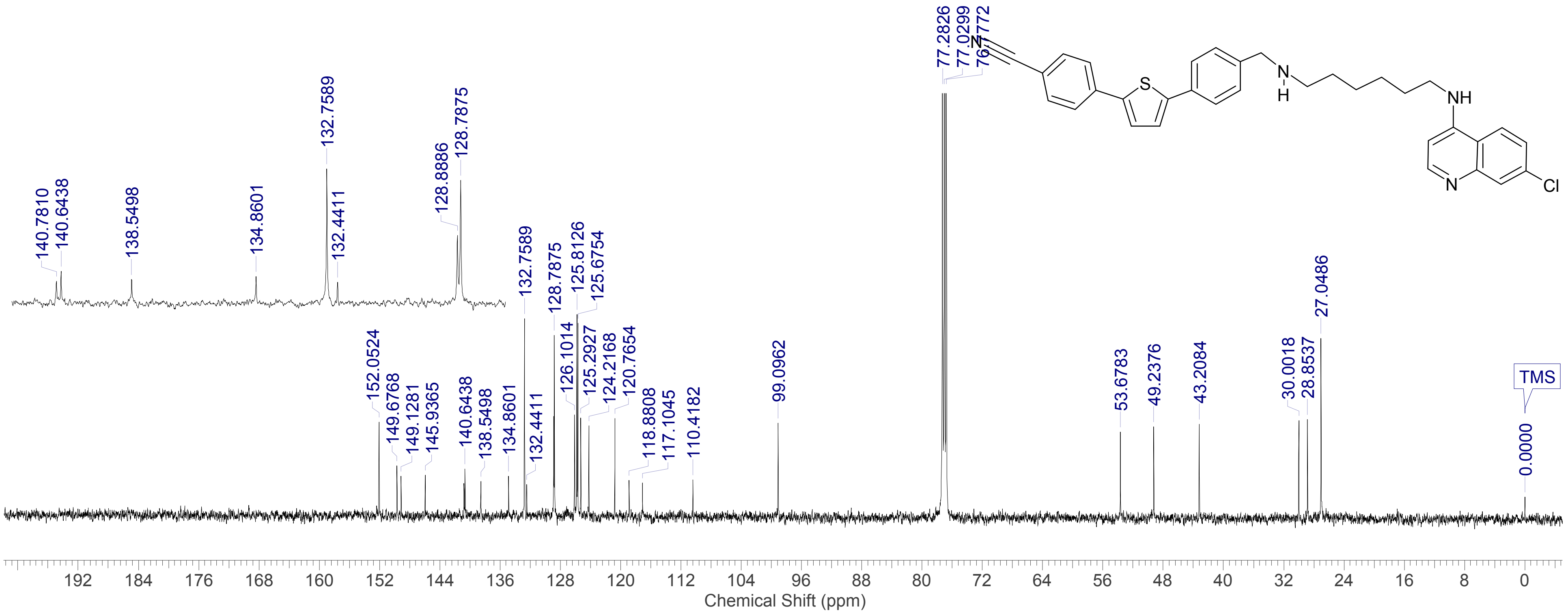

4-(5-{4-[({8-[(7-chloroquinolin-4-yl)amino]octyl}amino)methyl]phenyl}-2-thienyl)benzonitrile (42)

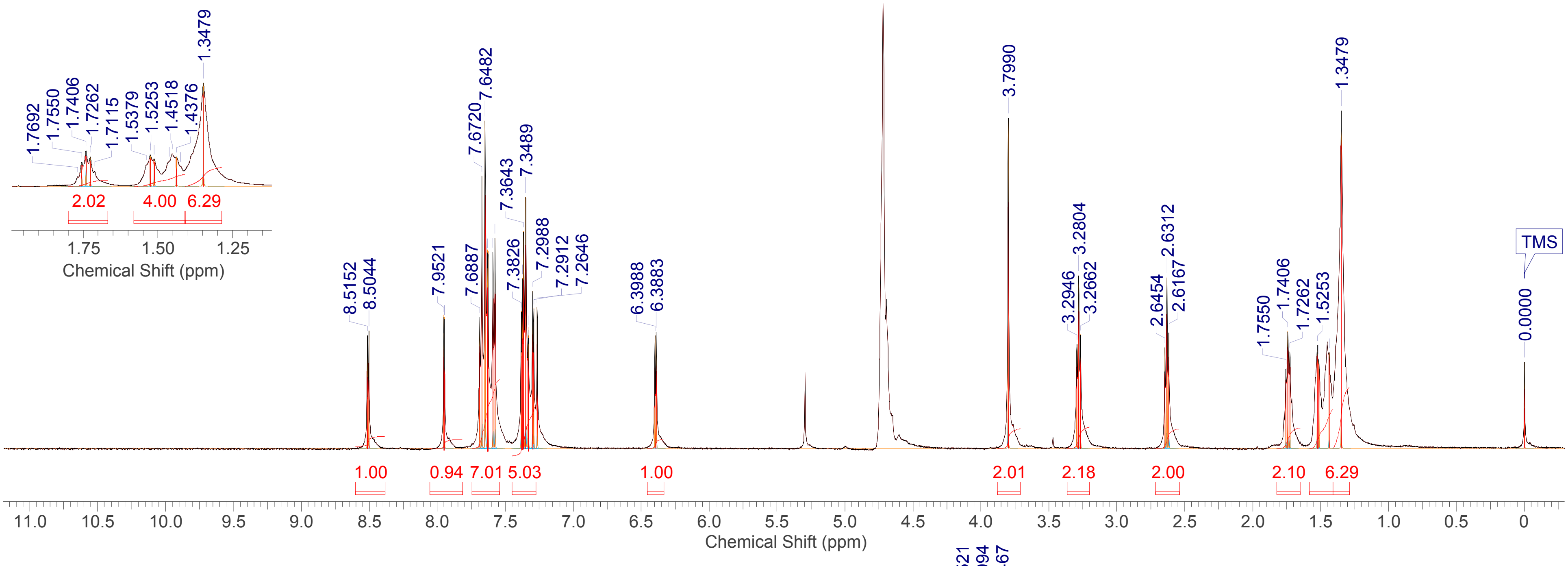

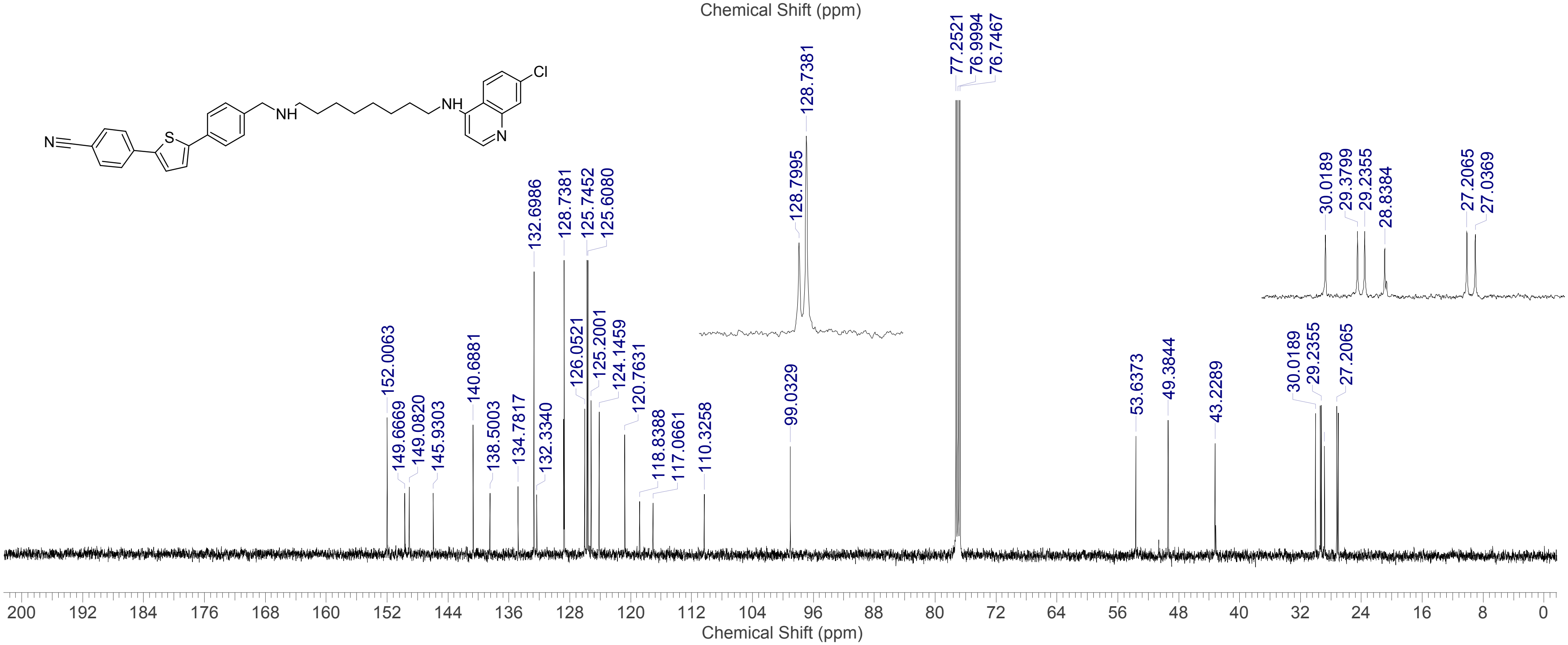

4-{5-[4-({[4-(quinolin-4-ylamino)butyl]amino}methyl)phenyl]-2-thienyl}benzonitrile (43)

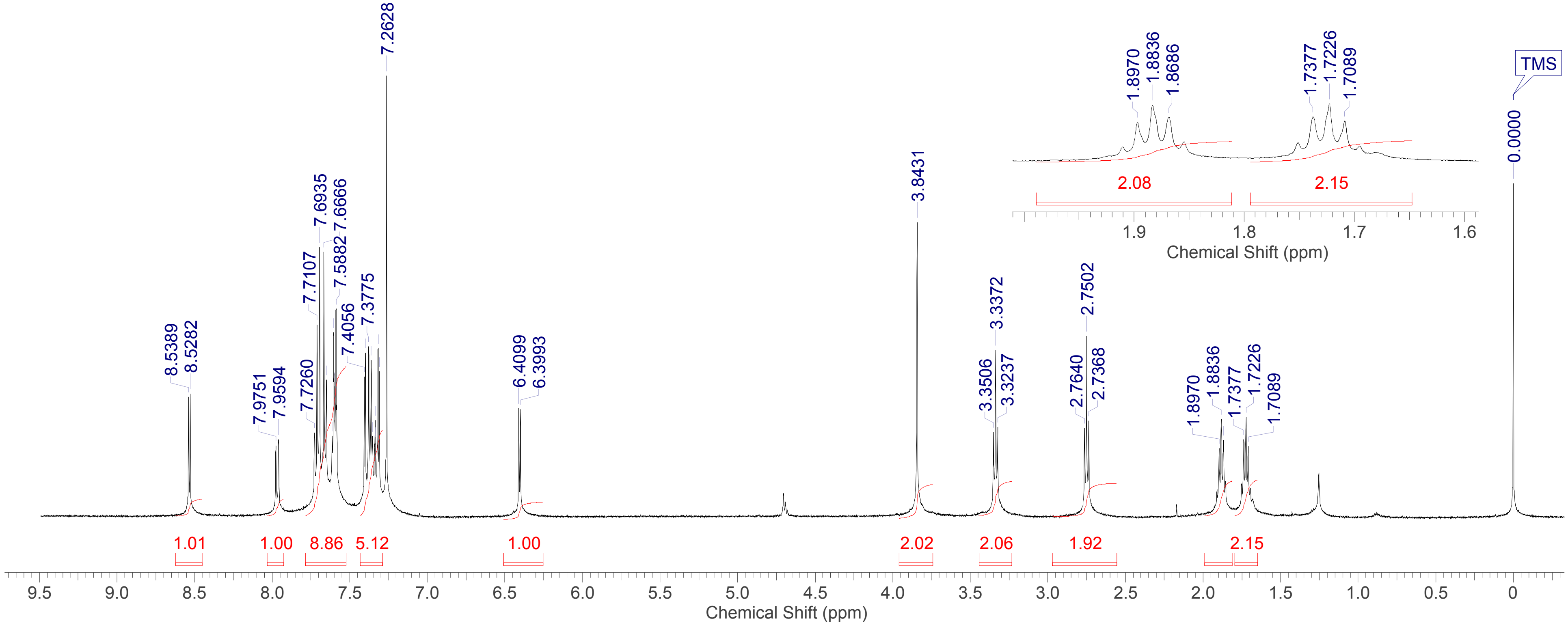

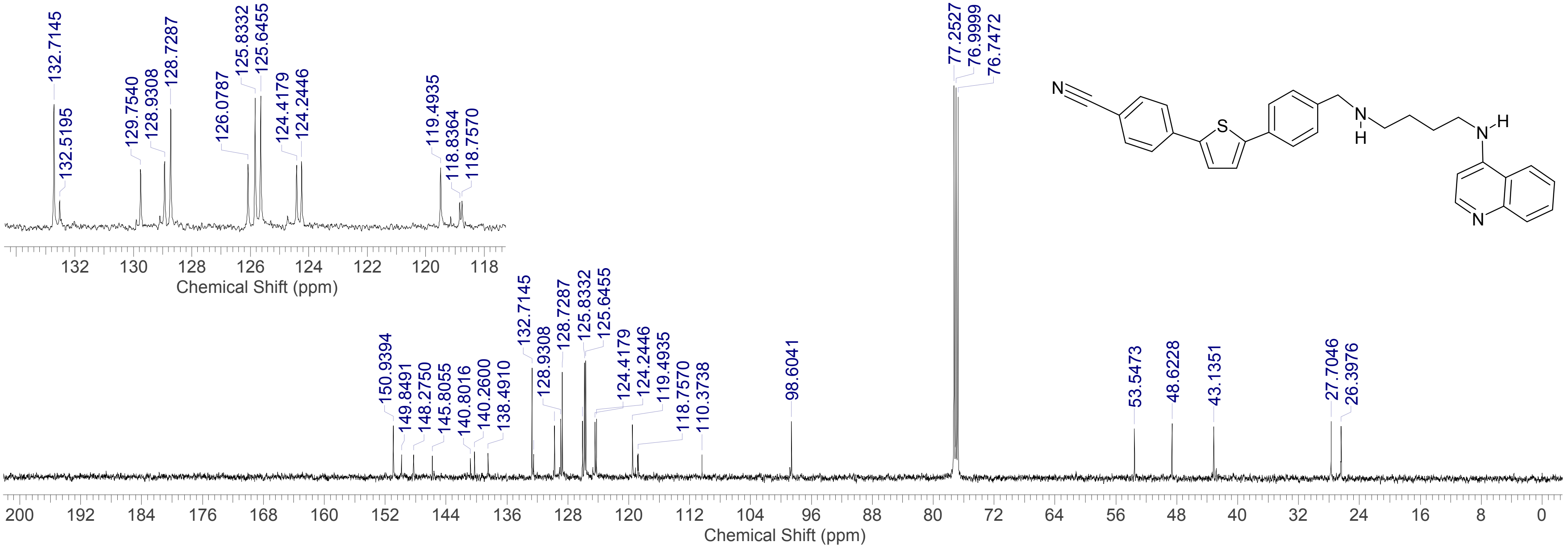

4-{5-[4-({[6-(quinolin-4-ylamino)hexyl]amino}methyl)phenyl]-2-thienyl}benzonitrile (44)

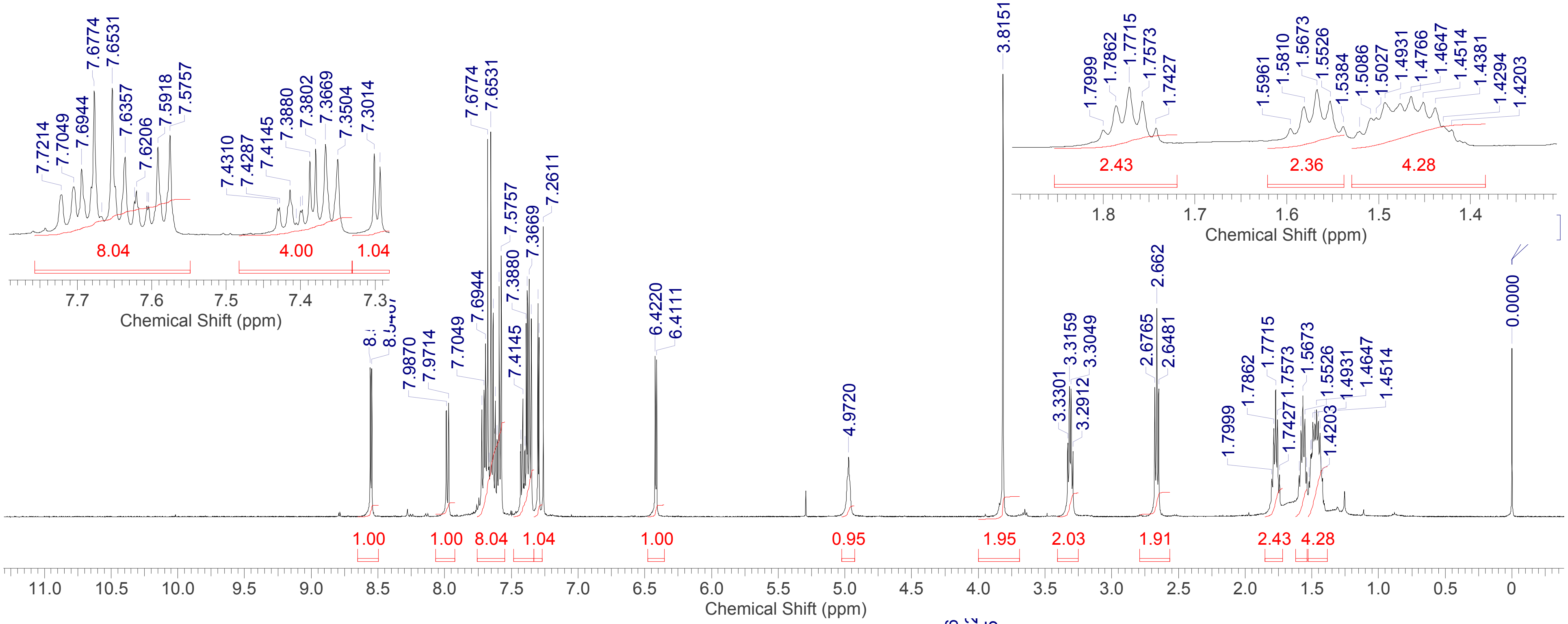

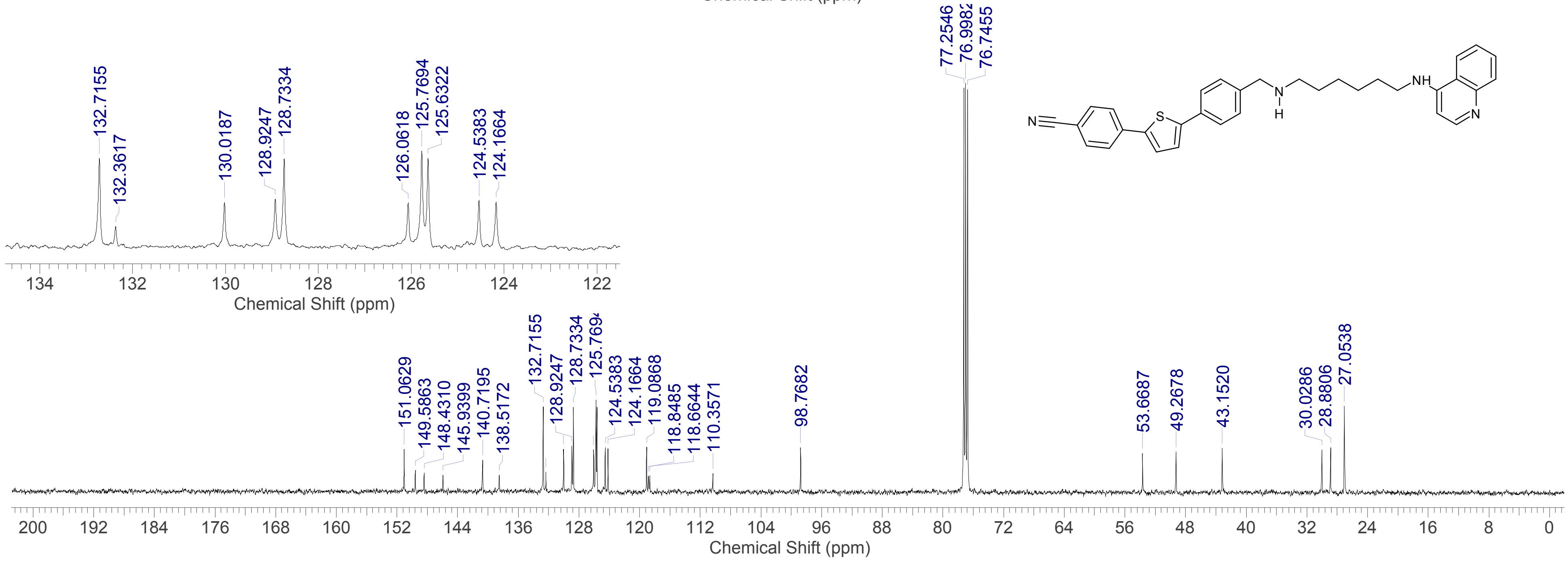

4-{5-[4-({methyl[8-(quinolin-4-ylamino)octyl]amino}methyl)phenyl]-2-thienyl}benzonitrile (46)

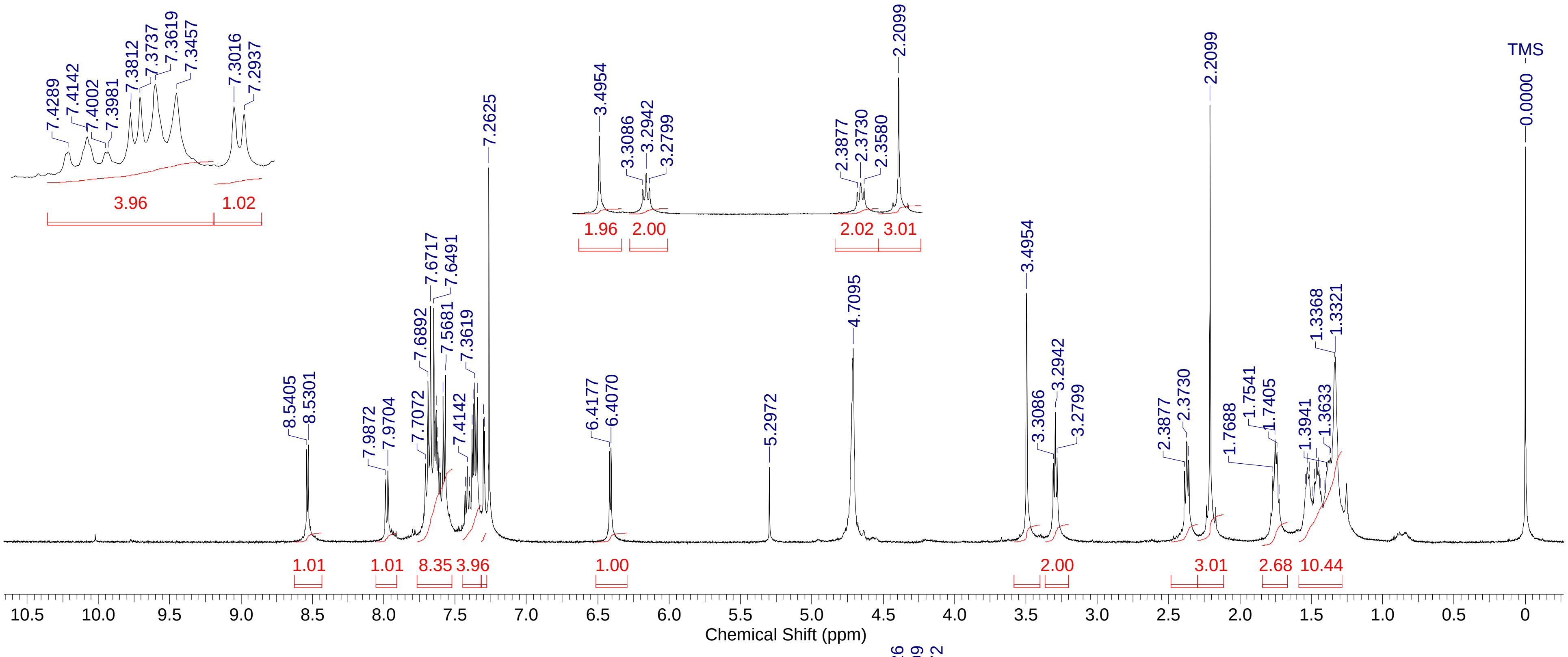

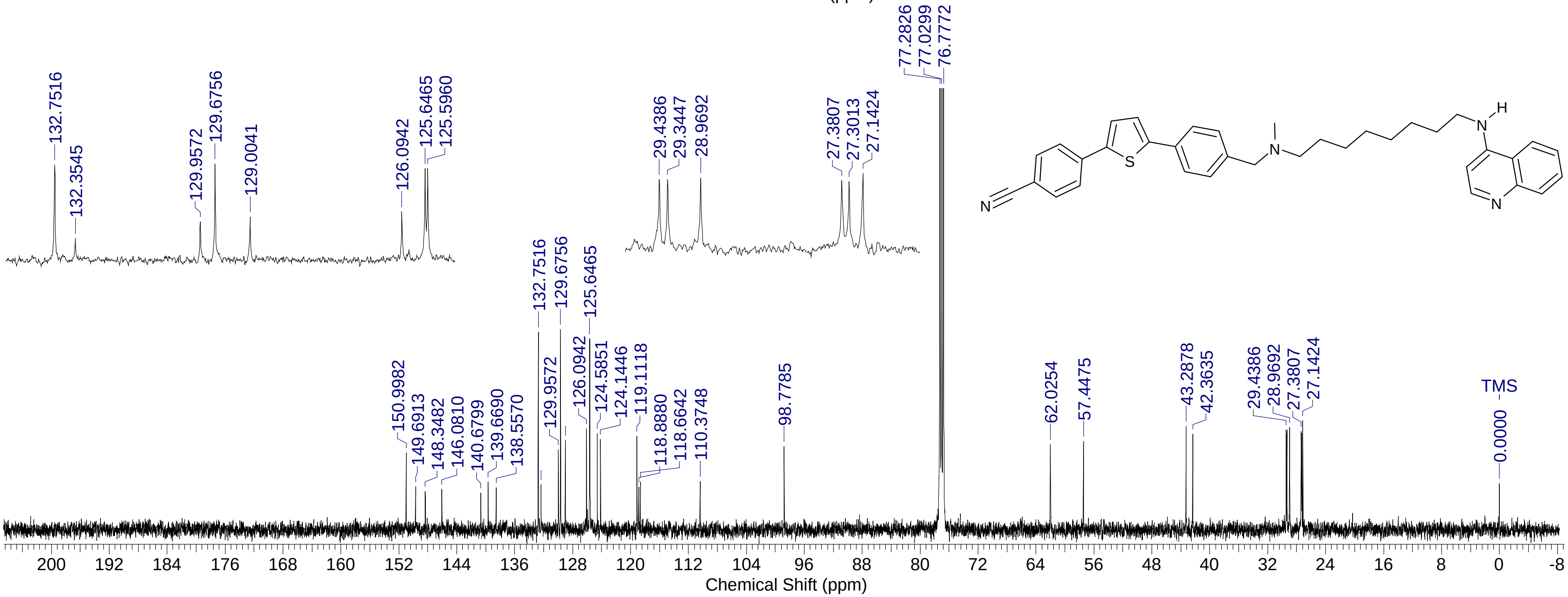

N-{4-[5-(4-ethynylphenyl)-2-thienyl]benzyl}-N'-quinolin-4-yloctane-1,8-diamine (52)

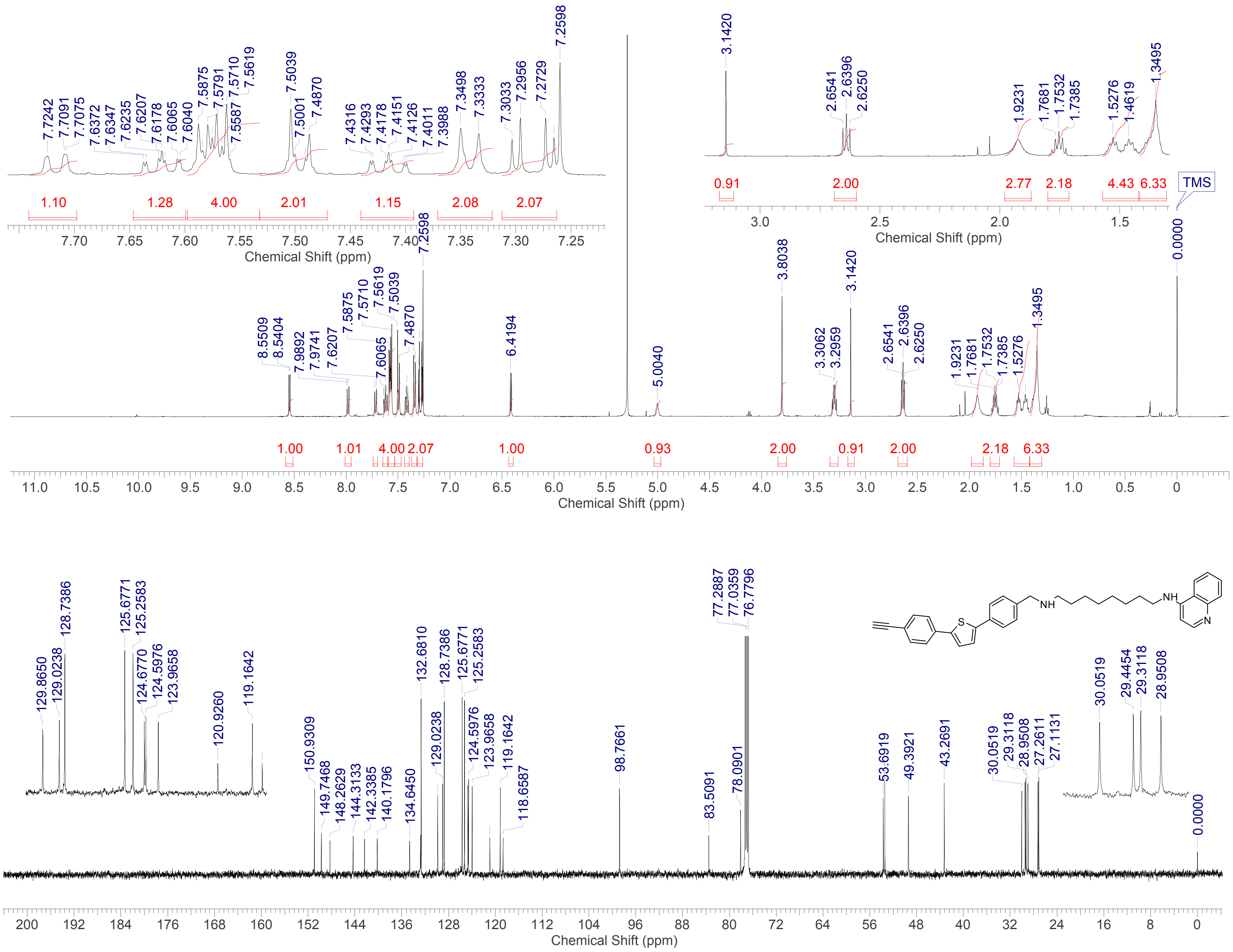

Compound: 8

Method A

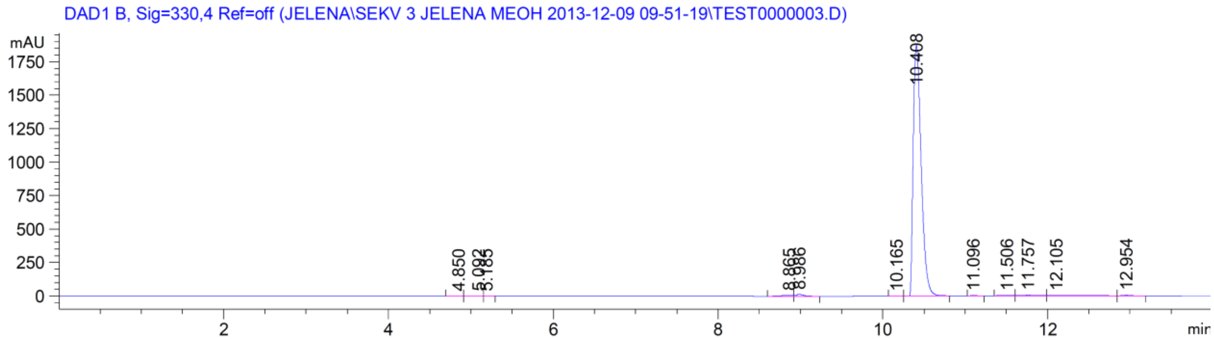

Signal 2: DAD1 B, Sig=330,4 Ref=off

Peak #	RetTime [min]	Type	Width [min]	Area [mAU*s]	Height [mAU]	Area %
1	4.850	BV	0.1185	12.05899	1.20222	0.0931
2	5.092	VV	0.1232	22.98553	2.21302	0.1774
3	5.185	VB	0.0602	6.02563	1.29629	0.0465
4	8.865	BV	0.1306	68.79704	6.28988	0.5310
5	8.986	VB	0.0933	89.84259	14.59206	0.6934
6	10.165	BV	0.0758	12.86007	2.44710	0.0992
7	10.408	VV	0.1058	1.25434e4	1869.86841	96.8059
8	11.096	BB	0.0682	6.48302	1.14317	0.0500
9	11.506	BV	0.0907	28.83651	4.11794	0.2226
10	11.757	VV	0.1681	64.71497	4.54664	0.4994
11	12.105	VV	0.3068	72.90954	2.79494	0.5627
12	12.954	VB	0.0869	28.35678	4.83394	0.2188
Totals :				1.29573e4	1915.34562	

Method B

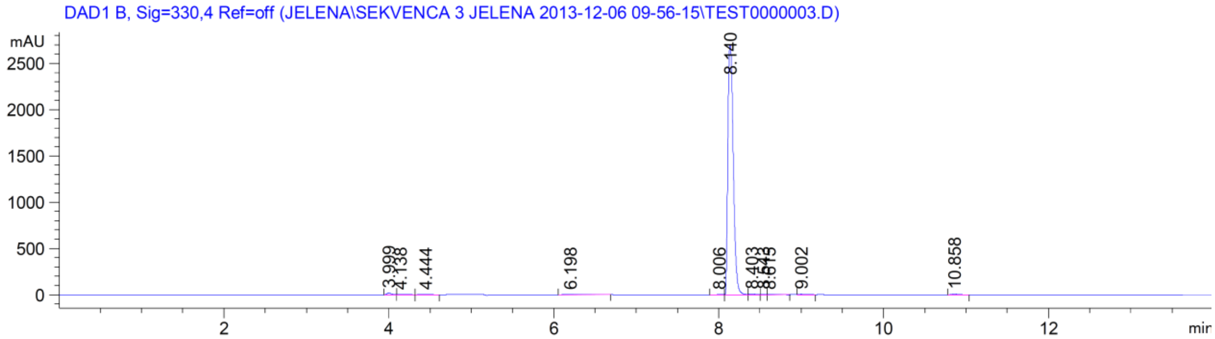

Signal 2: DAD1 B, Sig=330,4 Ref=off

Peak #	RetTime [min]	Type	Width [min]	Area [mAU*s]	Height [mAU]	Area %
1	3.999	BV	0.0671	94.15331	21.30407	0.7341
2	4.138	VV	0.1166	43.08921	4.46536	0.3359
3	4.444	VB	0.1275	31.27032	2.96527	0.2438
4	6.198	BB	0.1530	58.01780	4.58770	0.4523
5	8.006	BV	0.0632	27.83480	6.21669	0.2170
6	8.140	VV	0.0724	1.23704e4	2704.55054	96.4456
7	8.403	VV	0.0893	71.28415	11.26316	0.5558
8	8.543	VV	0.0624	30.18333	6.91841	0.2353
9	8.615	VB	0.0775	33.58511	5.59406	0.2618
10	9.002	VV	0.0662	28.54764	6.14638	0.2226
11	10.858	BB	0.0665	37.93799	8.85370	0.2958
Totals :				1.28263e4	2782.86534	

Compound: 9

Method A

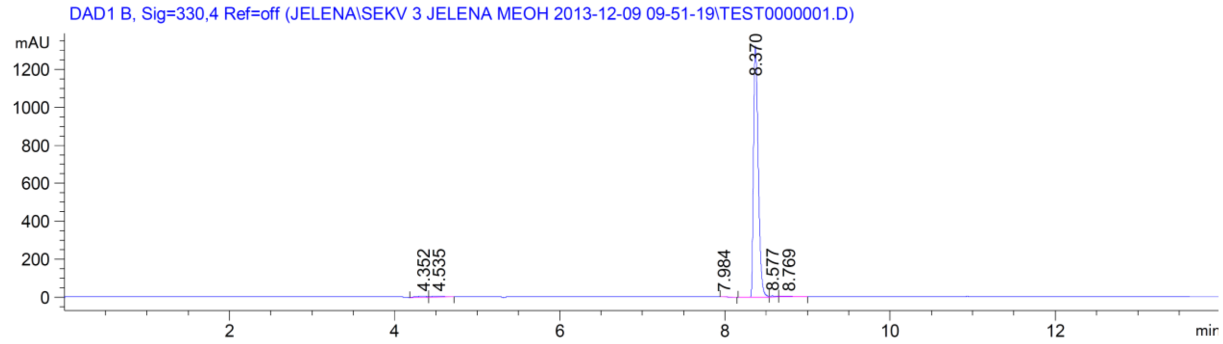

Signal 2: DAD1 B, Sig=330,4 Ref=off

Peak #	RetTime [min]	Type	Width [min]	Area [mAU*s]	Height [mAU]	Area %
1	4.352	BV	0.1243	57.48040	5.46058	1.0512
2	4.535	VB	0.1316	52.48553	4.72408	0.9598
3	7.984	BB	0.0690	8.03986	1.61602	0.1470
4	8.370	BV	0.0616	5289.68652	1325.03345	96.7339
5	8.577	VV	0.0726	30.47131	5.58997	0.5572
6	8.769	VB	0.1543	30.12488	2.33077	0.5509
Totals :				5468.28849	1344.75486	

Method B

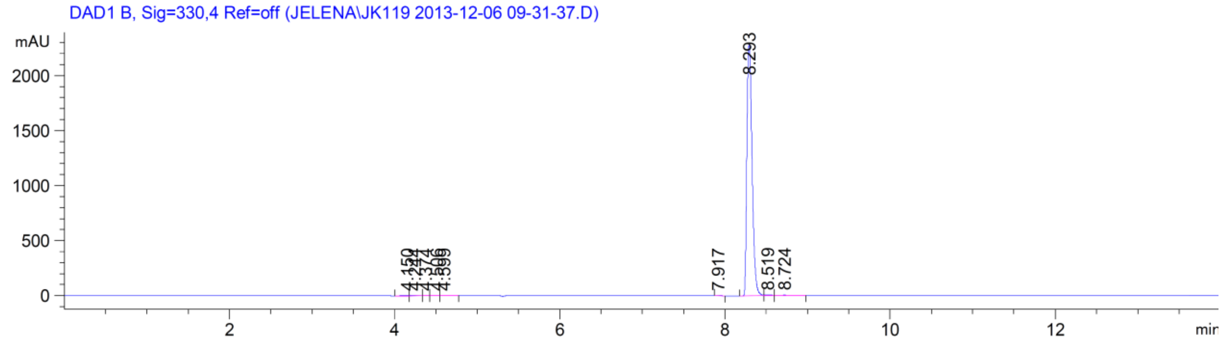

Signal 2: DAD1 B, Sig=330,4 Ref=off

Peak #	RetTime [min]	Type	Width [min]	Area [mAU*s]	Height [mAU]	Area %
1	4.150	BV	0.0873	45.32769	6.16115	0.4460
2	4.244	VV	0.0958	43.91695	5.61102	0.4321
3	4.374	VB	0.0496	7.37839	2.04480	0.0726
4	4.506	BV	0.0527	6.91900	1.57619	0.0681
5	4.599	VB	0.0947	14.64486	1.99405	0.1441
6	7.917	BB	0.0474	10.53126	3.41979	0.1036
7	8.293	BV	0.0683	9942.72656	2281.36108	97.8319
8	8.519	VV	0.0791	49.58509	9.02305	0.4879
9	8.724	VB	0.1409	42.04624	3.62201	0.4137
Totals :				1.01631e4	2314.81313	

Compound: 12

Method A

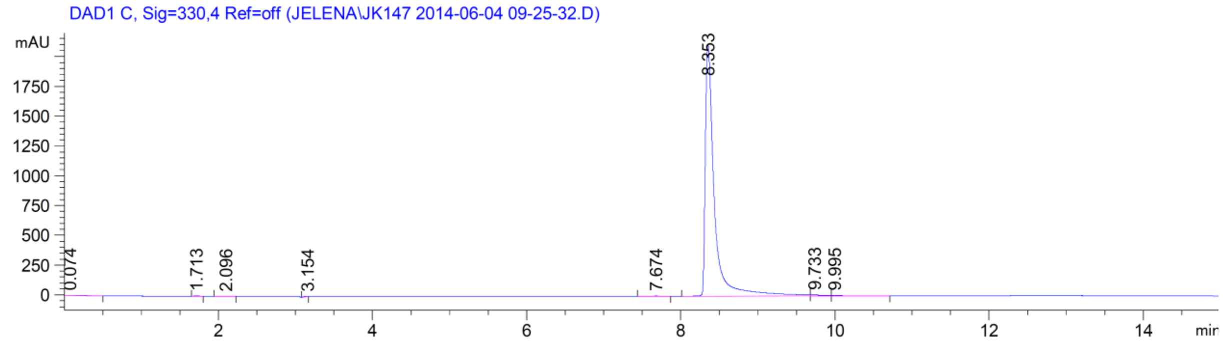

Signal 2: DAD1 C, Sig=330,4 Ref=off

Peak #	RetTime [min]	Type	Width [min]	Area [mAU*s]	Height [mAU]	Area %
1	0.074	BB	0.1604	19.67772	1.44898	0.1114
2	1.713	BB	0.0510	6.68019	1.95069	0.0378
3	2.096	BB	0.1023	8.61326	1.01226	0.0488
4	3.154	BB	0.0859	9.82954	1.59532	0.0557
5	7.674	BV	0.1022	23.39944	2.78123	0.1325
6	8.353	BV	0.1202	1.73580e4	2104.66650	98.3049
7	9.733	VV	0.1478	129.20125	10.63046	0.7317
8	9.995	VB	0.2171	101.90282	5.56680	0.5771

Totals : 1.76573e4 2129.65225

Method B

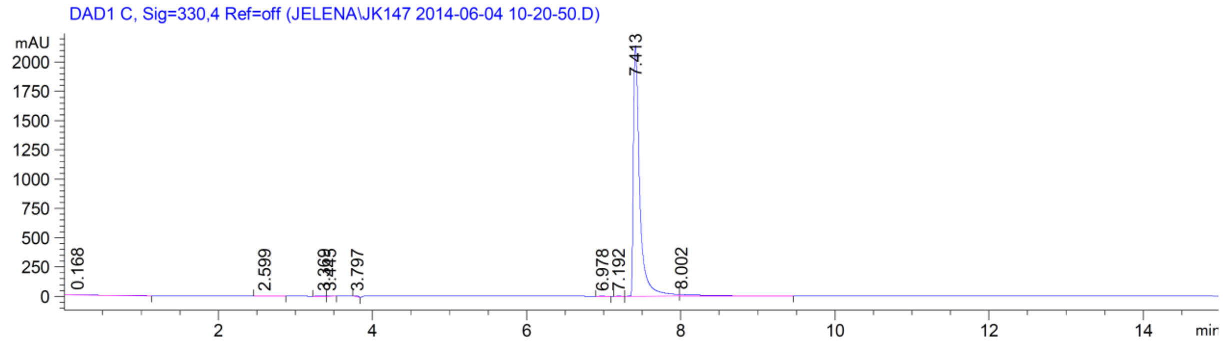

Signal 2: DAD1 C, Sig=330,4 Ref=off

Peak #	RetTime [min]	Type	Width [min]	Area [mAU*s]	Height [mAU]	Area %
1	0.168	BB	0.3394	70.85670	2.45041	0.5491
2	2.599	BB	0.1420	25.60596	2.12583	0.1984
3	3.369	BV	0.1117	38.72963	4.17586	0.3001
4	3.445	VB	0.0655	16.13107	3.11295	0.1250
5	3.797	BB	0.0503	20.28446	5.87740	0.1572
6	6.978	BB	0.0607	15.59155	3.77341	0.1208
7	7.192	BB	0.0540	7.76888	1.81285	0.0602
8	7.413	BV	0.0850	1.22846e4	2137.53125	95.1924
9	8.002	VB	0.3297	425.45694	15.38377	3.2968

Totals : 1.29050e4 2176.24373

Compound: 13

Method A

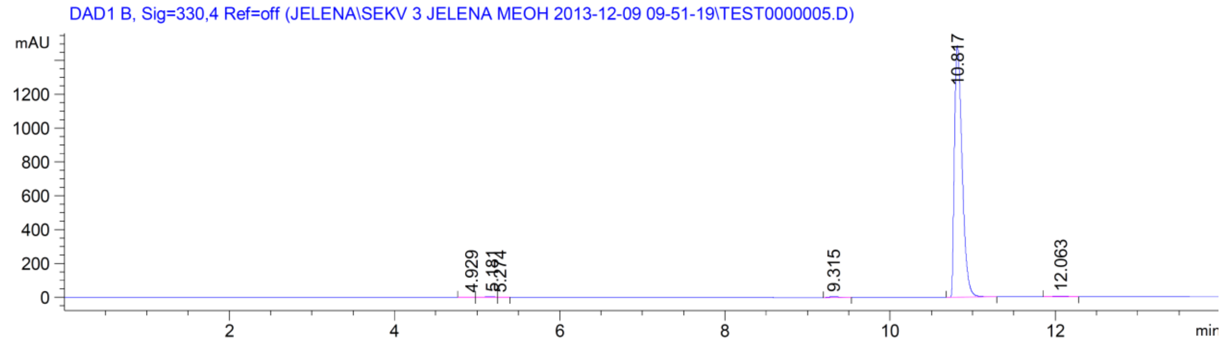

Signal 2: DAD1 B, Sig=330,4 Ref=off

Peak #	RetTime [min]	Type	Width [min]	Area [mAU*s]	Height [mAU]	Area %
1	4.929	BV	0.1265	12.74808	1.19916	0.1302
2	5.181	VV	0.1340	24.21175	2.19891	0.2473
3	5.274	VB	0.0565	5.68291	1.24002	0.0581
4	9.315	BB	0.0959	41.74291	6.17310	0.4264
5	10.817	BV	0.1028	9664.74316	1487.79065	98.7323
6	12.063	BB	0.1158	39.70742	4.05378	0.4056

Totals : 9788.83624 1502.65562

Method B

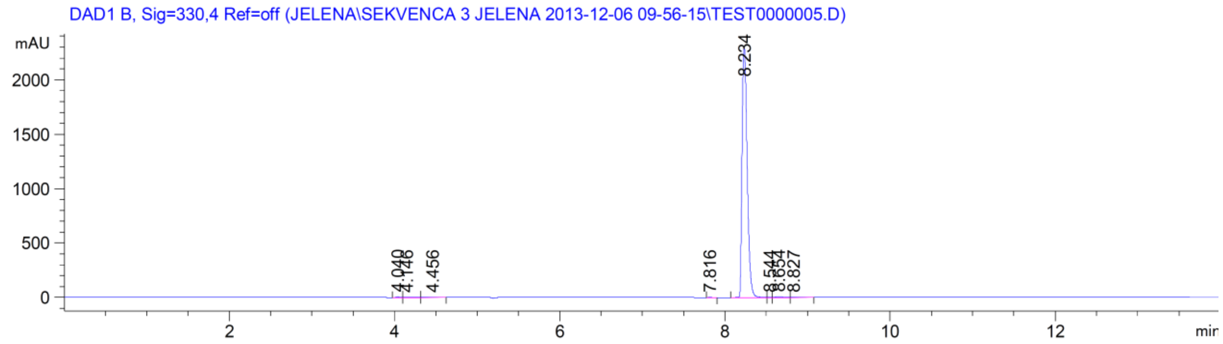

Signal 2: DAD1 B, Sig=330,4 Ref=off

Peak #	RetTime [min]	Type	Width [min]	Area [mAU*s]	Height [mAU]	Area %
1	4.040	BV	0.0758	45.86581	9.09407	0.4506
2	4.146	VV	0.1158	56.62686	5.93704	0.5563
3	4.456	VB	0.1422	40.98989	3.43617	0.4027
4	7.816	BB	0.0455	10.28627	3.42736	0.1011
5	8.234	BV	0.0677	9946.89648	2310.45972	97.7200
6	8.544	VV	0.0498	9.33347	2.51353	0.0917
7	8.654	VV	0.0921	58.95234	8.86755	0.5792
8	8.827	VB	0.1039	10.02561	1.19515	0.0985

Totals : 1.01790e4 2344.93058

Compound: 23

Method A

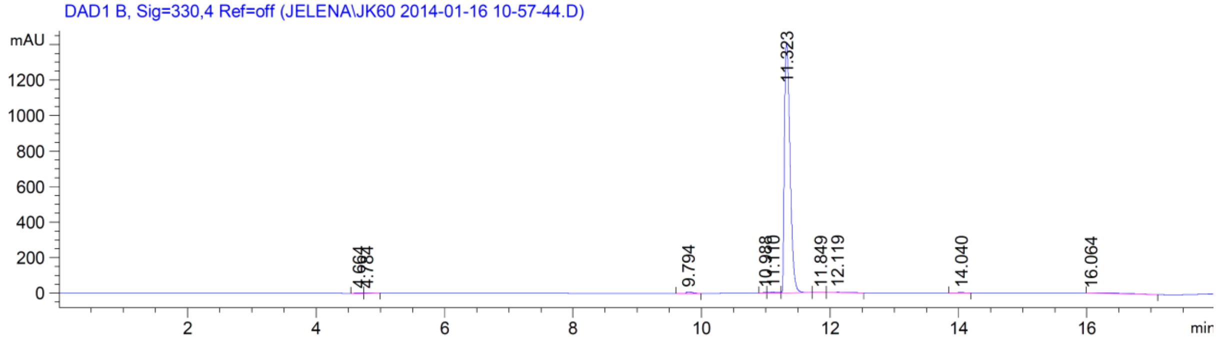

Signal 2: DAD1 B, Sig=330,4 Ref=off

Peak #	RetTime [min]	Type	Width [min]	Area [mAU*s]	Height [mAU]	Area %
1	4.664	BV	0.0943	15.66329	2.03467	0.1766
2	4.784	VB	0.0910	10.42214	1.35854	0.1175
3	9.794	BB	0.0914	56.56019	9.24286	0.6376
4	10.988	BV	0.0477	8.31650	2.12431	0.0937
5	11.110	VV	0.1061	39.14568	4.85521	0.4413
6	11.323	VB	0.0971	8559.00391	1404.85425	96.4787
7	11.849	BV	0.1092	9.35392	1.01358	0.1054
8	12.119	VB	0.1611	37.96208	2.81885	0.4279
9	14.040	BB	0.0871	18.44501	2.94247	0.2079
10	16.064	BB	1.3295	116.52189	1.02403	1.3135
Totals :				8871.39462	1432.26877	

Method B

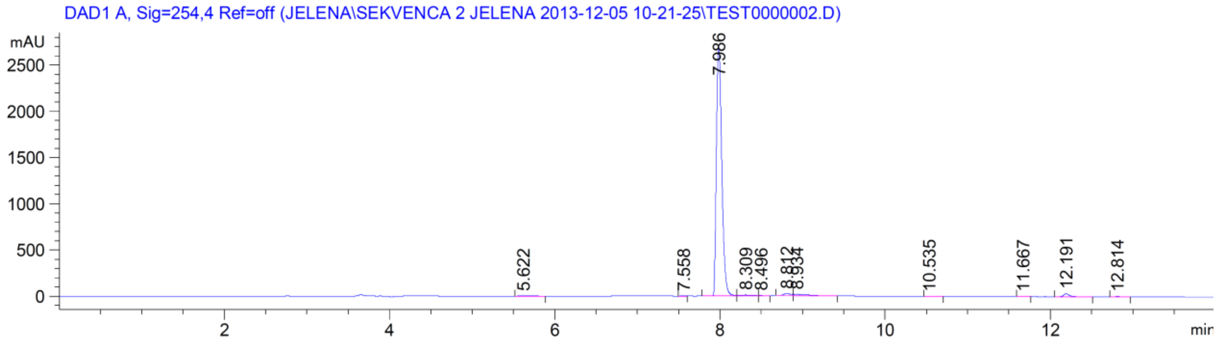

Signal 1: DAD1 A, Sig=254,4 Ref=off

Peak #	RetTime [min]	Type	Width [min]	Area [mAU*s]	Height [mAU]	Area %
1	5.622	BB	0.0967	15.96181	2.17336	0.1245
2	7.558	BV	0.0514	11.08910	3.24339	0.0865
3	7.986	BV	0.0700	1.22225e4	2713.69238	95.3222
4	8.309	VV	0.1264	85.21599	8.96584	0.6646
5	8.496	VB	0.0673	11.59744	2.46787	0.0904
6	8.812	BV	0.0940	133.48244	22.41257	1.0410
7	8.934	VB	0.1666	176.08990	14.01727	1.3733
8	10.535	BB	0.0686	7.66952	1.65335	0.0598
9	11.667	BV	0.0669	9.78625	2.26665	0.0763
10	12.191	BB	0.0672	143.71243	32.43956	1.1208
11	12.814	BB	0.0705	5.20053	1.03634	0.0406
Totals :				1.28223e4	2804.36858	

Compound: 24

Method A

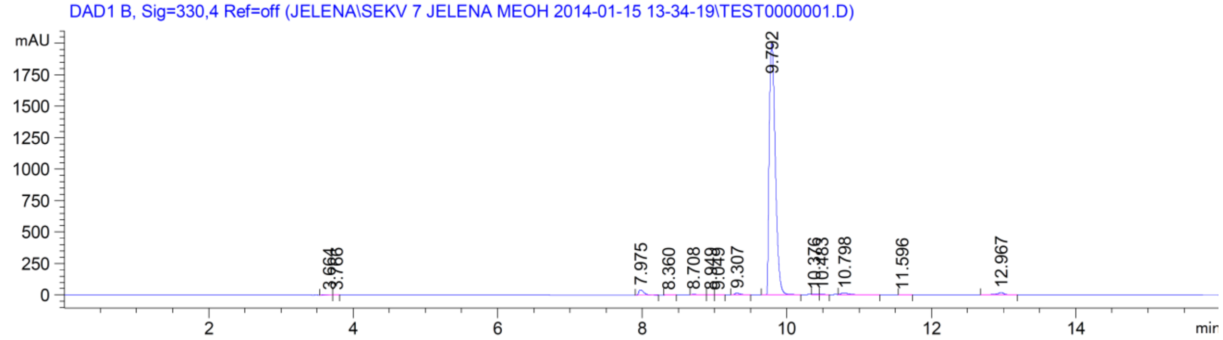

Signal 2: DAD1 B, Sig=330,4 Ref=off

Peak #	RetTime [min]	Type	Width [min]	Area [mAU*s]	Height [mAU]	Area %
1	3.664	VV	0.0817	11.33854	1.89843	0.0928
2	3.766	VV	0.0561	6.78175	1.62783	0.0555
3	7.975	BB	0.0761	201.10028	41.83434	1.6464
4	8.360	BB	0.0634	16.86065	3.97890	0.1380
5	8.708	BB	0.0658	23.51722	4.74509	0.1925
6	8.949	BV	0.0594	5.58993	1.13657	0.0458
7	9.049	VB	0.0600	7.36560	1.56366	0.0603
8	9.307	BB	0.0766	68.93701	13.49542	0.5644
9	9.792	BB	0.0929	1.16359e4	1998.52209	95.2615
10	10.376	VV	0.0614	6.96108	1.47843	0.0570
11	10.483	VB	0.0618	5.27069	1.08283	0.0432
12	10.798	VB	0.1018	91.19942	11.40279	0.7466
13	11.596	BB	0.0584	5.00760	1.05414	0.0410
14	12.967	VB	0.0952	128.86189	18.98979	1.0550
Totals :				1.22147e4	2102.81032	

Method B

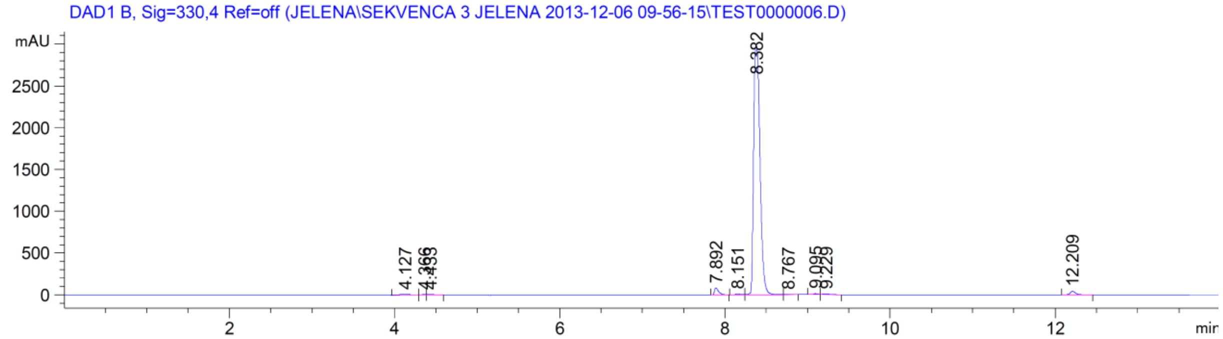

Signal 2: DAD1 B, Sig=330,4 Ref=off

Peak #	RetTime [min]	Type	Width [min]	Area [mAU*s]	Height [mAU]	Area %
1	4.127	BV	0.1630	87.12283	6.39188	0.5202
2	4.366	VV	0.0617	19.44756	3.77032	0.1161
3	4.433	VB	0.0866	23.66706	3.51180	0.1413
4	7.892	BB	0.0540	301.16214	84.70996	1.7982
5	8.151	BV	0.0736	58.17103	10.58412	0.3473
6	8.382	VV	0.0735	1.59474e4	2998.71411	95.2217
7	8.767	VB	0.0793	9.72310	1.49650	0.0581
8	9.095	BV	0.0587	48.58176	12.40609	0.2901
9	9.229	VB	0.0835	63.92706	10.58218	0.3817
10	12.209	BB	0.0675	188.44490	42.24783	1.1252
Totals :				1.67476e4	3174.41478	

Compound: 25

Method A

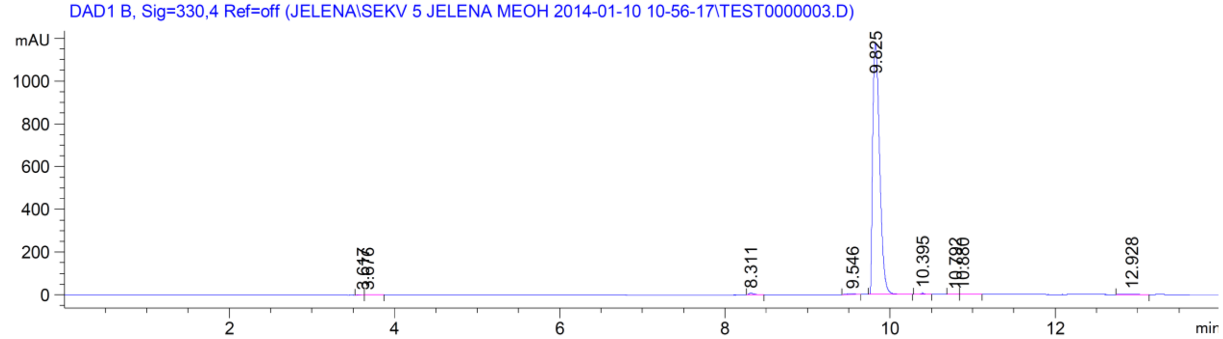

Signal 2: DAD1 B, Sig=330,4 Ref=off

Peak #	RetTime [min]	Type	Width [min]	Area [mAU*s]	Height [mAU]	Area %
1	3.617	BV	0.0505	9.37448	2.25364	0.1340
2	3.676	VB	0.0847	14.90127	2.23736	0.2131
3	8.311	VB	0.0742	39.13313	8.04708	0.5596
4	9.546	BB	0.0928	29.24839	4.00034	0.4182
5	9.825	BB	0.0931	6851.14600	1174.38684	97.9665
6	10.395	BB	0.0227	13.68492	7.53647	0.1957
7	10.792	VV	0.0836	9.87055	1.40295	0.1411
8	10.880	VB	0.0773	9.74870	1.57217	0.1394
9	12.928	BB	0.0831	16.24894	2.61204	0.2323
Totals :				6993.35639	1204.04889	

Method B

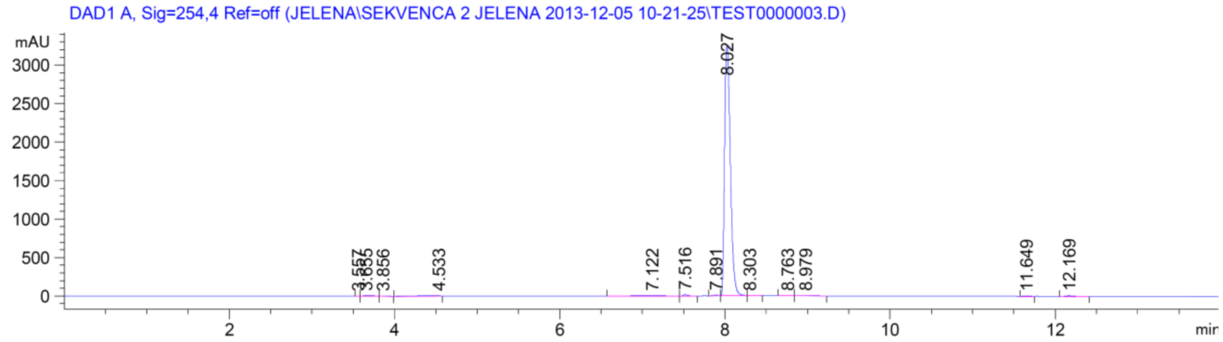

Signal 1: DAD1 A, Sig=254,4 Ref=off

Peak #	RetTime [min]	Type	Width [min]	Area [mAU*s]	Height [mAU]	Area %
1	3.557	BV	0.0368	7.45170	3.07264	0.0453
2	3.655	VV	0.1398	77.40693	7.00958	0.4704
3	3.856	VB	0.0833	31.95885	5.22805	0.1942
4	4.533	BB	0.3180	214.98785	8.12900	1.3066
5	7.122	BB	0.2686	92.61446	4.06795	0.5629
6	7.516	BB	0.0513	54.86071	16.51079	0.3334
7	7.891	BV	0.0584	36.18497	9.19101	0.2199
8	8.027	VV	0.0676	1.58162e4	3257.52295	96.1242
9	8.303	VB	0.0713	26.08952	5.17310	0.1586
10	8.763	BV	0.0728	9.52312	1.67680	0.0579
11	8.979	VB	0.1424	40.52629	3.51855	0.2463
12	11.649	BV	0.0633	9.24310	2.14548	0.0562
13	12.169	BB	0.0674	36.87713	8.28553	0.2241
Totals :				1.64539e4	3331.53142	

Compound: 26

Method A

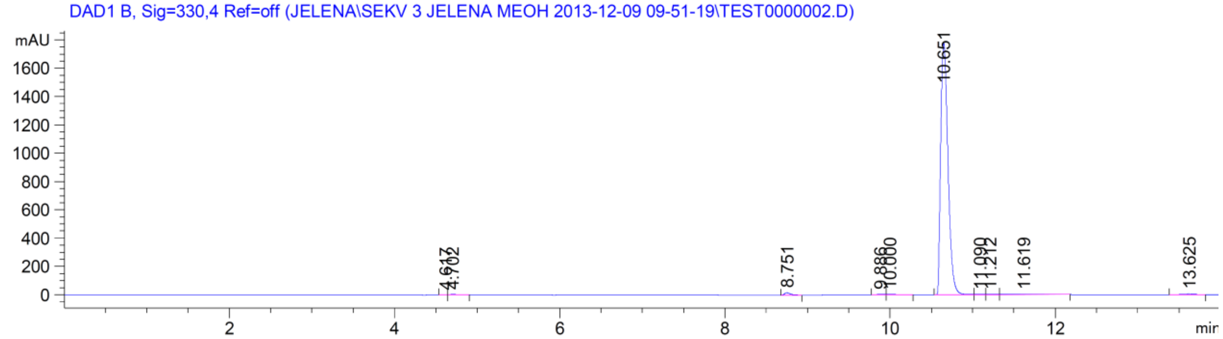

Signal 2: DAD1 B, Sig=330,4 Ref=off

Peak #	RetTime [min]	Type	Width [min]	Area [mAU*s]	Height [mAU]	Area %
1	4.617	BV	0.0554	7.51402	1.87047	0.0675
2	4.702	VB	0.0986	21.65252	2.60144	0.1946
3	8.751	BB	0.0759	79.05142	15.65897	0.7105
4	9.886	BV	0.0829	29.91393	5.21826	0.2689
5	10.000	VB	0.0964	30.30073	4.18722	0.2723
6	10.651	BV	0.0972	1.08238e4	1774.77820	97.2785
7	11.090	VV	0.0813	17.35509	2.58638	0.1560
8	11.212	VV	0.0998	16.42139	1.95930	0.1476
9	11.619	VB	0.2318	64.19098	3.25205	0.5769
10	13.625	BB	0.0865	36.40666	5.98266	0.3272
Totals :				1.11266e4	1818.09495	

Method B

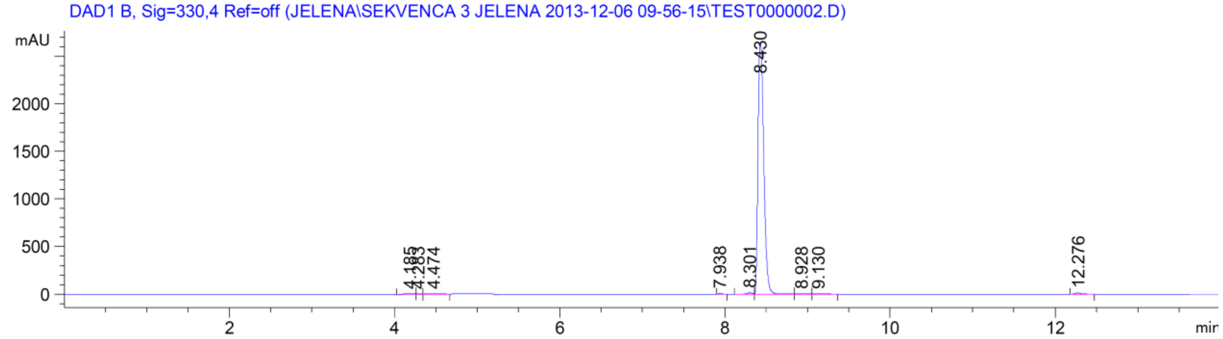

Signal 2: DAD1 B, Sig=330,4 Ref=off

Peak #	RetTime [min]	Type	Width [min]	Area [mAU*s]	Height [mAU]	Area %
1	4.185	BV	0.1262	72.88445	6.92958	0.5492
2	4.283	VV	0.0562	23.05473	5.05257	0.1737
3	4.474	VB	0.1468	55.43713	4.48294	0.4177
4	7.938	BV	0.0474	39.08035	12.89380	0.2945
5	8.301	BV	0.0724	81.92766	16.95385	0.6173
6	8.430	VV	0.0770	1.28938e4	2638.54663	97.1565
7	8.928	VV	0.1118	24.42923	2.61844	0.1841
8	9.130	VB	0.0905	25.61717	3.66795	0.1930
9	12.276	BB	0.0679	54.92933	12.23009	0.4139
Totals :				1.32711e4	2703.37585	

Compound: 27

Method A

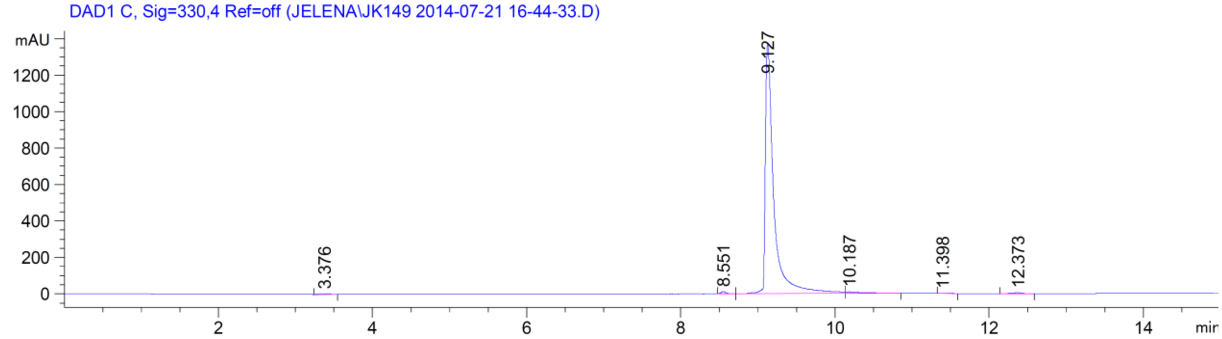

Signal 2: DAD1 C, Sig=330,4 Ref=off

Peak #	RetTime [min]	Type	Width [min]	Area [mAU*s]	Height [mAU]	Area %
1	3.376	BB	0.1286	37.46206	3.46634	0.3481
2	8.551	VB	0.0662	58.72265	12.74624	0.5457
3	9.127	BV	0.1110	1.04794e4	1373.59509	97.3772
4	10.187	VB	0.2017	143.71709	8.41833	1.3355
5	11.398	BB	0.0582	5.11887	1.07167	0.0476
6	12.373	BB	0.0892	37.23857	5.66381	0.3460

Totals : 1.07616e4 1404.96148

Method B

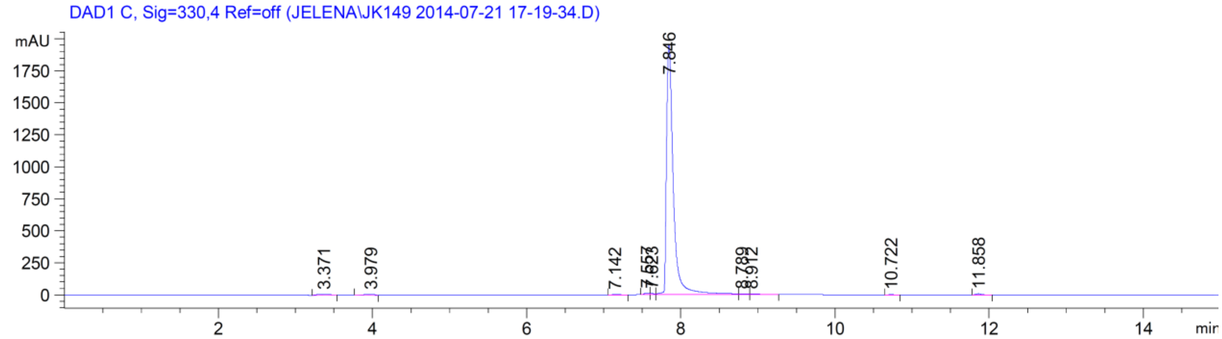

Signal 2: DAD1 C, Sig=330,4 Ref=off

Peak #	RetTime [min]	Type	Width [min]	Area [mAU*s]	Height [mAU]	Area %
1	3.371	BB	0.1493	54.54555	4.33396	0.4380
2	3.979	BB	0.1362	43.53336	3.76921	0.3496
3	7.142	BB	0.0835	33.94343	5.61969	0.2726
4	7.557	BV	0.0688	53.47674	12.15959	0.4294
5	7.623	VV	0.0534	34.94339	8.98462	0.2806
6	7.846	VV	0.0937	1.21029e4	1956.65942	97.1804
7	8.789	VV	0.0937	44.77769	6.28099	0.3595
8	8.912	VB	0.1204	41.32400	4.14327	0.3318
9	10.722	BB	0.0551	5.33902	1.28228	0.0429
10	11.858	VB	0.0663	39.26548	8.93423	0.3153

Totals : 1.24540e4 2012.16725

Compound: 28

Method A

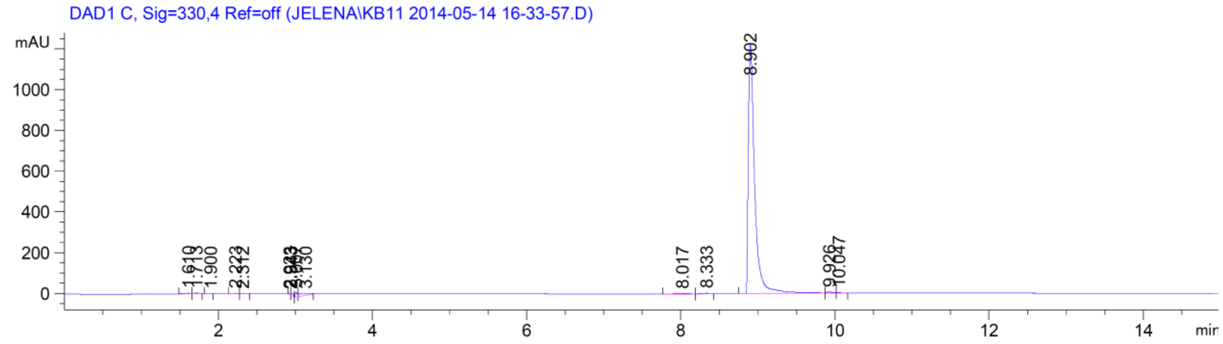

Signal 2: DAD1 C, Sig=330,4 Ref=off

Peak #	RetTime [min]	Type	Width [min]	Area [mAU*s]	Height [mAU]	Area %
1	1.610	BB	0.0779	15.70328	2.58483	0.2156
2	1.713	BV	0.0513	12.59033	3.04276	0.1729
3	1.900	VV	0.0643	9.27365	1.76377	0.1274
4	2.223	VV	0.0677	10.39338	1.99806	0.1427
5	2.312	VV	0.0659	6.52515	1.27125	0.0896
6	2.923	BV	0.0229	5.54642	3.41748	0.0762
7	2.943	VB	0.0422	26.24262	7.53159	0.3604
8	3.007	BV	0.0277	38.72638	21.87623	0.5318
9	3.130	VV	0.1229	91.16618	8.79532	1.2520
10	8.017	VB	0.1427	16.69414	1.37970	0.2293
11	8.333	BB	0.0752	16.95468	2.87798	0.2328
12	8.902	BV	0.0855	6996.87158	1219.09692	96.0861
13	9.926	VV	0.0699	27.38257	5.37854	0.3760
14	10.047	VV	0.0694	7.80772	1.44832	0.1072

Totals : 7281.87809 1282.46276

Method B

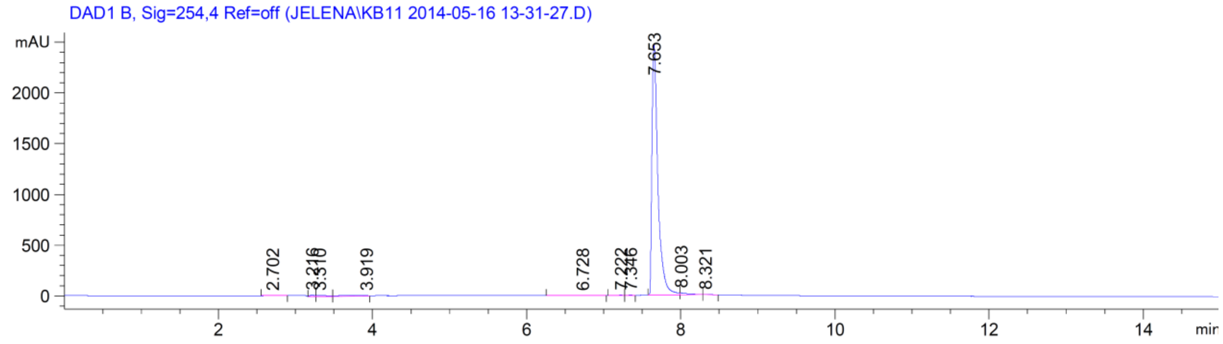

Signal 1: DAD1 B, Sig=254,4 Ref=off

Peak #	RetTime [min]	Type	Width [min]	Area [mAU*s]	Height [mAU]	Area %
1	2.702	BB	0.1061	9.49930	1.05988	0.0688
2	3.216	BV	0.0634	49.92383	12.14818	0.3614
3	3.310	VV	0.1107	77.92776	8.47815	0.5642
4	3.919	VB	0.2681	172.10172	7.76571	1.2460
5	6.728	BB	0.2594	59.97980	2.72341	0.4342
6	7.222	BV	0.0990	23.79228	3.05056	0.1722
7	7.346	VB	0.0686	10.72035	1.96540	0.0776
8	7.653	BV	0.0813	1.32674e4	2464.38770	96.0514
9	8.003	VB	0.1029	128.24153	16.54515	0.9284
10	8.321	BB	0.0638	13.22463	2.89726	0.0957

Totals : 1.38128e4 2521.02139

Compound: 29

Method A

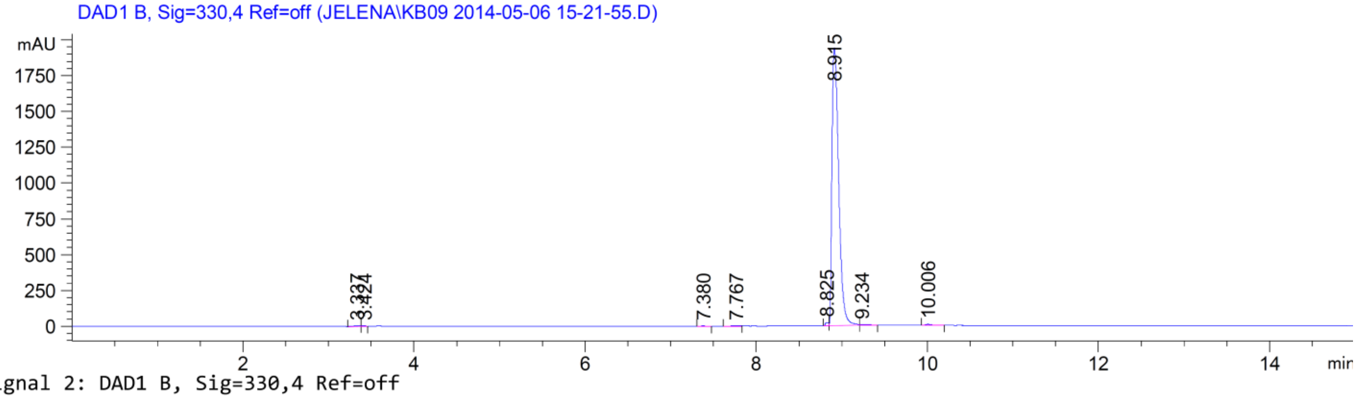

Signal 2: DAD1 B, Sig=330,4 Ref=off

Peak #	RetTime [min]	Type	Width [min]	Area [mAU*s]	Height [mAU]	Area %
1	3.337	BV	0.0872	25.04193	3.62127	0.2336
2	3.424	VB	0.0548	5.00681	1.12849	0.0467
3	7.380	BB	0.0482	5.18607	1.32440	0.0484
4	7.767	BV	0.0729	10.99308	1.82142	0.1026
5	8.825	BV	0.0401	59.87083	23.63365	0.5586
6	8.915	VV	0.0853	1.05243e4	1941.10217	98.1950
7	9.234	VB	0.0827	45.85837	6.83856	0.4279
8	10.006	VB	0.0749	41.49577	8.01592	0.3872

Totals : 1.07178e4 1987.48589

Method B

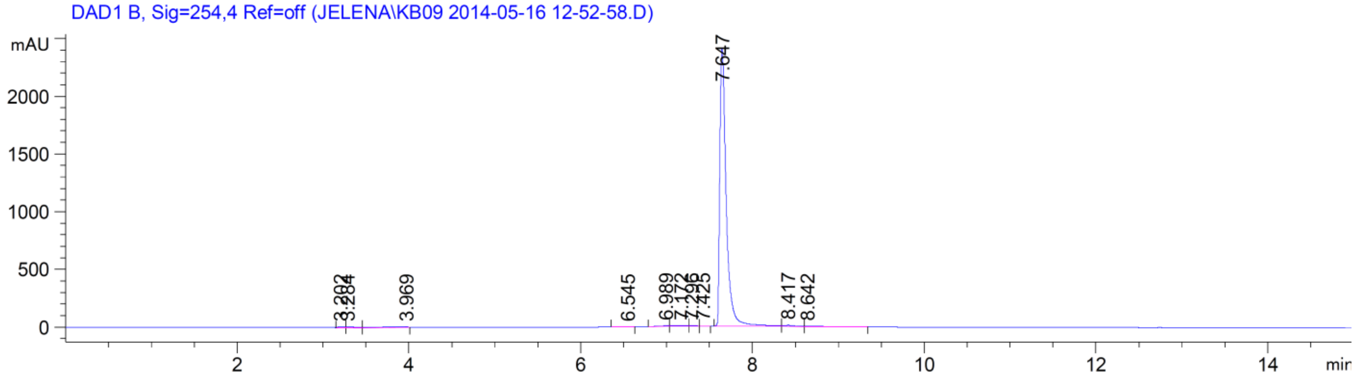

Signal 1: DAD1 B, Sig=254,4 Ref=off

Peak #	RetTime [min]	Type	Width [min]	Area [mAU*s]	Height [mAU]	Area %
1	3.202	BV	0.0764	52.98580	10.40431	0.4002
2	3.284	VV	0.0877	55.00214	7.53007	0.4154
3	3.969	VB	0.2957	170.33707	6.94471	1.2864
4	6.545	BB	0.0887	11.36586	1.54739	0.0858
5	6.989	BV	0.0933	48.44630	6.94798	0.3659
6	7.172	VV	0.1366	101.32356	9.08473	0.7652
7	7.296	VV	0.0696	33.77491	6.14939	0.2551
8	7.425	VB	0.0690	13.18127	2.36600	0.0995
9	7.647	BV	0.0795	1.25998e4	2410.41748	95.1541
10	8.417	VV	0.1227	99.51318	10.87686	0.7515
11	8.642	VB	0.1922	55.73877	3.40944	0.4209

Totals : 1.32414e4 2475.67835

Compound: 30

Method A

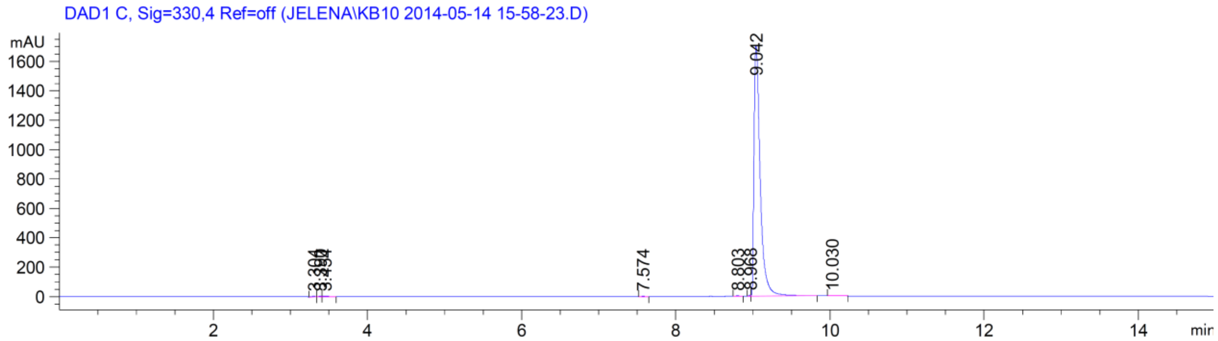

Signal 2: DAD1 C, Sig=330,4 Ref=off

Peak #	RetTime [min]	Type	Width [min]	Area [mAU*s]	Height [mAU]	Area %
1	3.304	BV	0.0493	16.06239	4.23136	0.1669
2	3.390	VV	0.0467	11.89283	3.13706	0.1236
3	3.454	VB	0.0773	18.19798	2.84010	0.1891
4	7.574	BV	0.0485	7.66518	2.35465	0.0796
5	8.803	BV	0.0545	13.79927	3.88356	0.1434
6	8.968	BV	0.0280	11.70945	6.65575	0.1217
7	9.042	VV	0.0842	9537.06250	1706.78528	99.0906
8	10.030	BB	0.0879	8.19428	1.11260	0.0851
Totals :				9624.58387	1731.00036	

Method B

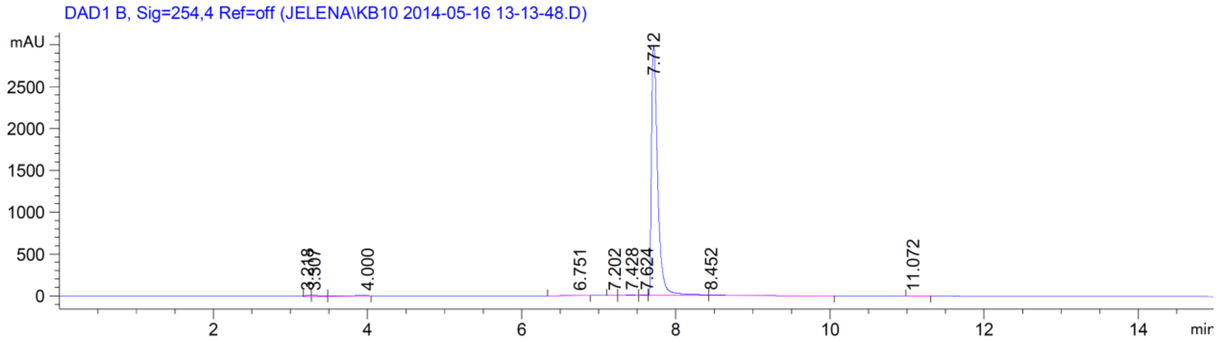

Signal 1: DAD1 B, Sig=254,4 Ref=off

Peak #	RetTime [min]	Type	Width [min]	Area [mAU*s]	Height [mAU]	Area %
1	3.218	BV	0.0672	52.70157	11.89874	0.3043
2	3.307	VV	0.0993	67.16257	8.21857	0.3879
3	4.000	VB	0.3056	162.46524	6.37006	0.9382
4	6.751	BV	0.2007	23.72937	1.38993	0.1370
5	7.202	BV	0.0586	6.79644	1.42566	0.0392
6	7.428	VB	0.1261	28.87228	2.75825	0.1667
7	7.624	BV	0.0659	21.37490	4.42103	0.1234
8	7.712	VV	0.0848	1.67501e4	2992.34570	96.7285
9	8.452	VB	0.2838	192.19623	8.02754	1.1099
10	11.072	BV	0.0648	11.21800	2.41173	0.0648
Totals :				1.73166e4	3039.26721	

Compound: 31

Method A

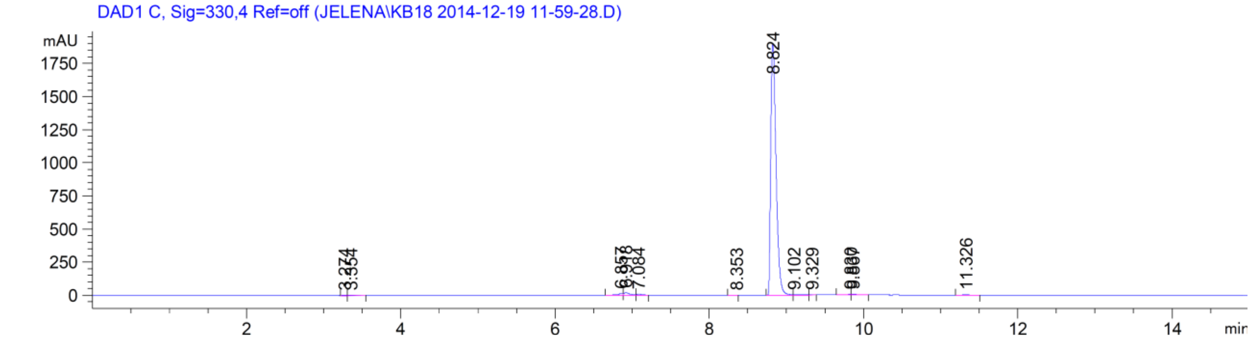


```
Signal 2: DAD1 C, Sig=330,4 Ref=off

Peak RetTime Type  Width     Area      Height     Area
  #   [min]        [min]   [mAU*s]     [mAU]       %
----|-------|----|-------|----------|----------|--------|
   1   3.274 BV    0.0493   10.80486    2.91279   0.1149
   2   3.354 VB    0.0990   24.59103    2.94322   0.2615
   3   6.857 BV    0.0663   60.44729   12.41429   0.6428
   4   6.918 VV    0.0740   97.95042   18.87545   1.0416
   5   7.084 VB    0.0541   30.88347    8.35320   0.3284
   6   8.353 BV    0.0611    5.07837    1.01095   0.0540
   7   8.824 BV    0.0744 9083.40430 1897.02832  96.5961
   8   9.102 VV    0.0853   18.90624    2.69666   0.2011
   9   9.329 VV    0.0601    5.40396    1.09434   0.0575
  10   9.830 BV    0.0487   21.79220    6.03165   0.2317
  11   9.867 VB    0.0637   30.03695    6.71436   0.3194
  12  11.326 BB    0.0685   14.19316    2.73837   0.1509

Totals :                  9403.49227 1962.81359
```

Method B

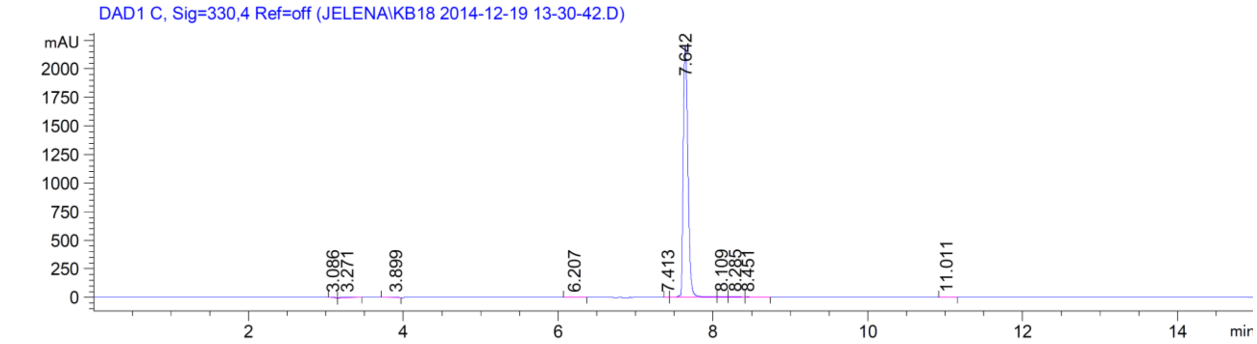


```
Signal 2: DAD1 C, Sig=330,4 Ref=off

Peak RetTime Type  Width     Area      Height     Area
  #   [min]        [min]   [mAU*s]     [mAU]       %
----|-------|----|-------|----------|----------|--------|
   1   3.086 BB    0.0644   15.37207    2.87526   0.1604
   2   3.271 BB    0.1461   71.78635    5.91384   0.7489
   3   3.899 BB    0.1071   35.31371    3.90318   0.3684
   4   6.207 BB    0.0936   11.34405    1.45344   0.1183
   5   7.413 BV    0.0405    5.69019    2.01068   0.0594
   6   7.642 VV    0.0663 9308.26074 2204.40454  97.1037
   7   8.109 VV    0.0847   43.05571    7.00236   0.4492
   8   8.285 VV    0.1146   58.04589    6.35468   0.6055
   9   8.451 VB    0.0865   22.64468    3.36756   0.2362
  10  11.011 VV    0.0601   14.38130    3.56447   0.1500

Totals :                  9585.89468 2240.85000
```

Compound: 32

Method A

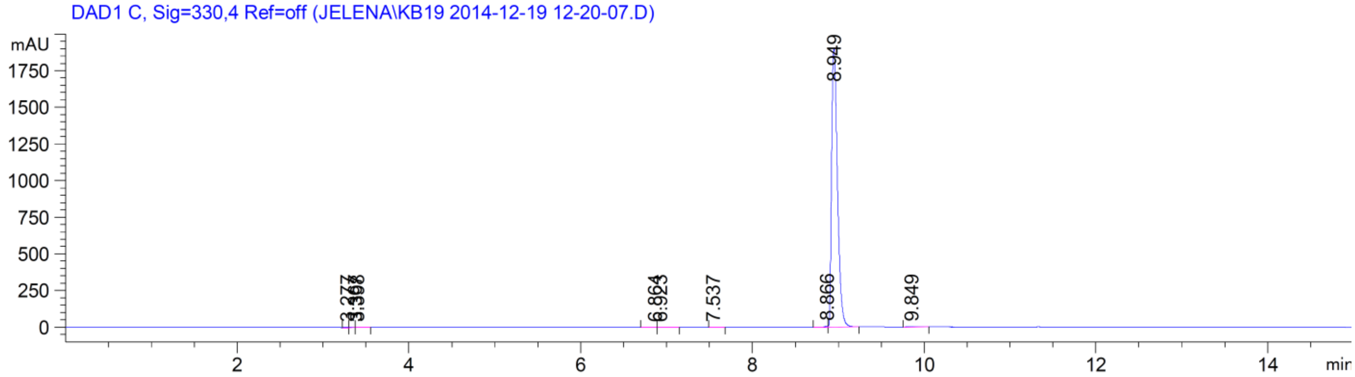

Signal 2: DAD1 C, Sig=330,4 Ref=off

Peak #	RetTime [min]	Type	Width [min]	Area [mAU*s]	Height [mAU]	Area %
1	3.277	BV	0.0440	11.43471	3.17432	0.1267
2	3.367	VV	0.0518	11.68167	2.71204	0.1294
3	3.398	VB	0.0707	14.61853	2.46387	0.1619
4	6.864	BV	0.0727	9.55289	1.58673	0.1058
5	6.923	VV	0.0863	12.87940	1.95542	0.1427
6	7.537	BB	0.0501	8.28078	2.40734	0.0917
7	8.866	BV	0.0401	17.60524	7.04945	0.1950
8	8.949	VV	0.0727	8931.37207	1904.48181	98.9348
9	9.849	BB	0.0682	10.10827	1.92651	0.1120

Totals : 9027.53357 1927.75751

Method B

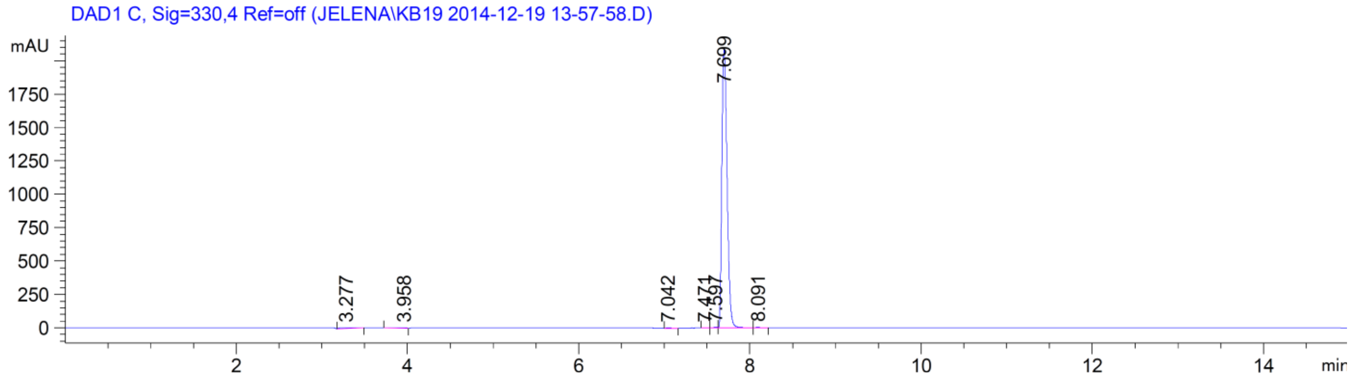

Signal 2: DAD1 C, Sig=330,4 Ref=off

Peak #	RetTime [min]	Type	Width [min]	Area [mAU*s]	Height [mAU]	Area %
1	3.277	BB	0.1383	77.06673	6.57384	0.8579
2	3.958	VB	0.1217	37.16552	3.65288	0.4137
3	7.042	BB	0.0436	7.98812	2.69098	0.0889
4	7.471	VB	0.0455	5.80909	1.90782	0.0647
5	7.597	BV	0.0583	14.32890	3.57426	0.1595
6	7.699	VV	0.0669	8829.40625	2084.87109	98.2832
7	8.091	VB	0.0631	11.87412	2.53636	0.1322

Totals : 8983.63873 2105.80724

Compound: 37

Method E

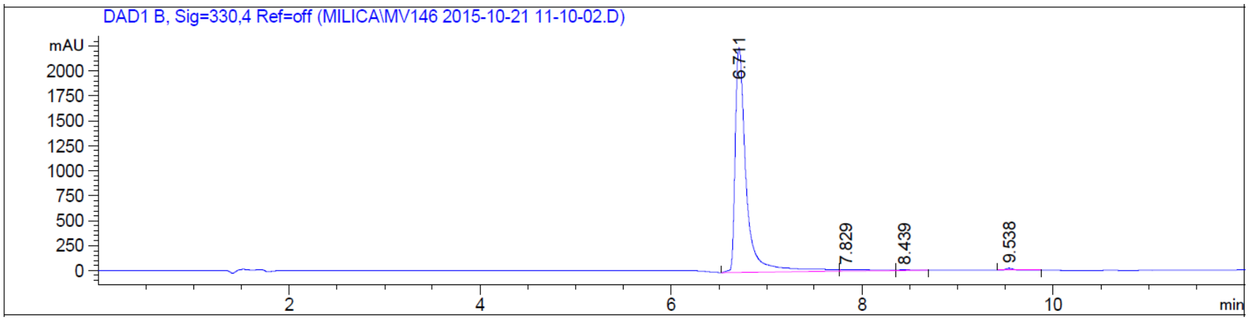

Peak #	RetTime [min]	Type	Width [min]	Area [mAU*s]	Height [mAU]	Area %
1	6.711	BV	0.1195	1.82389e4	2252.57471	96.6559
2	7.829	VV	0.2625	397.60565	17.98081	2.1071
3	8.439	VB	0.1208	99.11401	11.24984	0.5252
4	9.538	BB	0.0940	134.30275	20.89571	0.7117

Totals : 1.88699e4 2302.70107

Method F

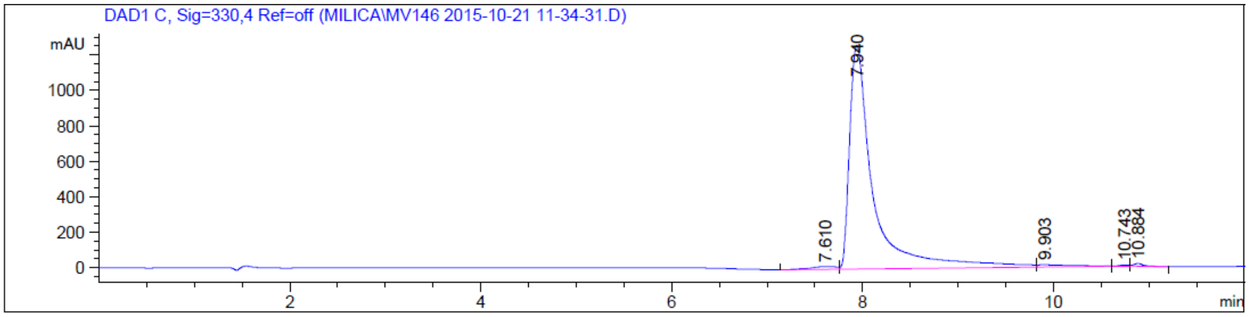

Peak #	RetTime [min]	Type	Width [min]	Area [mAU*s]	Height [mAU]	Area %
1	7.610	BV	0.2310	294.36676	14.99576	1.3172
2	7.940	VV	0.2484	2.16302e4	1259.08069	96.7885
3	9.903	VB	0.2489	268.31442	12.70510	1.2006
4	10.743	BV	0.0781	43.19379	6.75414	0.1933
5	10.884	VB	0.1038	111.83592	15.94952	0.5004

Totals : 2.23479e4 1309.48521

Compound: 38

Method C

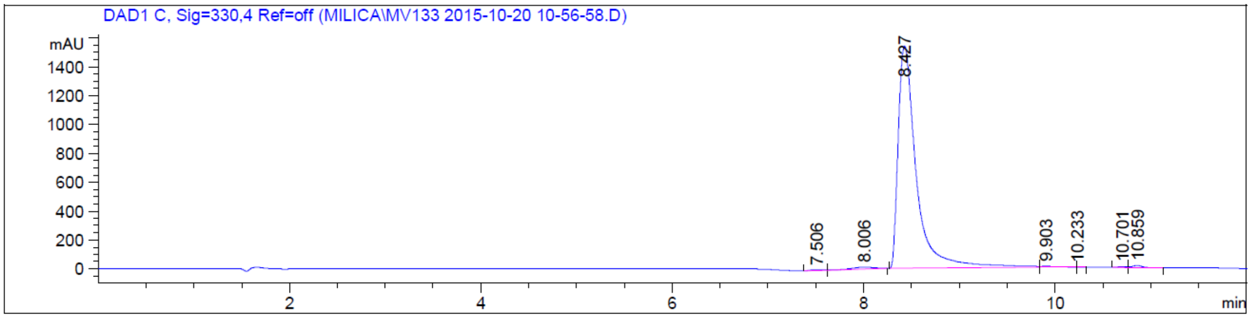

Peak #	RetTime [min]	Type	Width [min]	Area [mAU*s]	Height [mAU]	Area %
1	7.506	BV	0.1035	50.68055	5.88504	0.2439
2	8.006	VB	0.1723	195.34010	13.86675	0.9399
3	8.427	BV	0.1985	2.03127e4	1538.38354	97.7393
4	9.903	VB	0.1387	80.54507	6.87300	0.3876
5	10.233	BB	0.0317	4.16970e-1	1.72298e-1	2.006e-3
6	10.701	BV	0.0792	39.33719	6.58619	0.1893
7	10.859	VB	0.1012	103.50700	14.16939	0.4980
Totals :				2.07825e4	1585.93621	

Method D

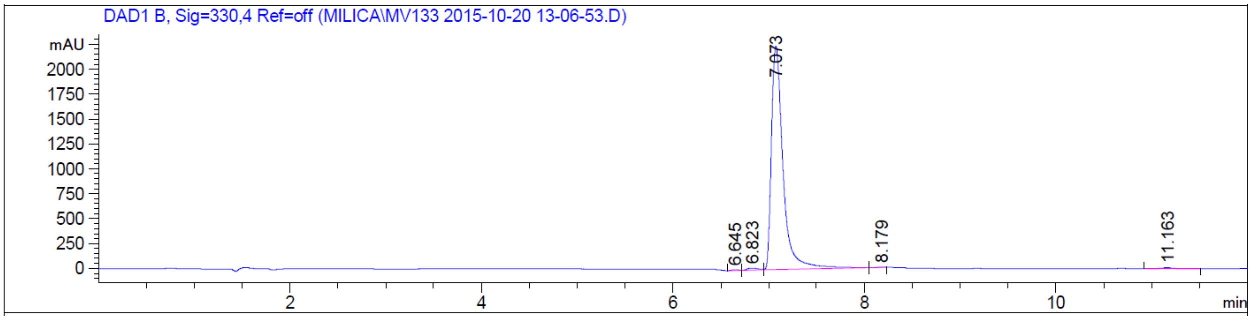

Peak #	RetTime [min]	Type	Width [min]	Area [mAU*s]	Height [mAU]	Area %
1	6.645	BV	0.0760	45.04110	8.68723	0.2325
2	6.823	VV	0.1168	178.07773	22.88065	0.9191
3	7.073	VV	0.1138	1.90274e4	2247.26343	98.2077
4	8.179	VB	0.1417	14.16832	1.17885	0.0731
5	11.163	BV	0.1225	109.96989	10.87646	0.5676
Totals :				1.93747e4	2290.88661	

Compound: 39

Method C

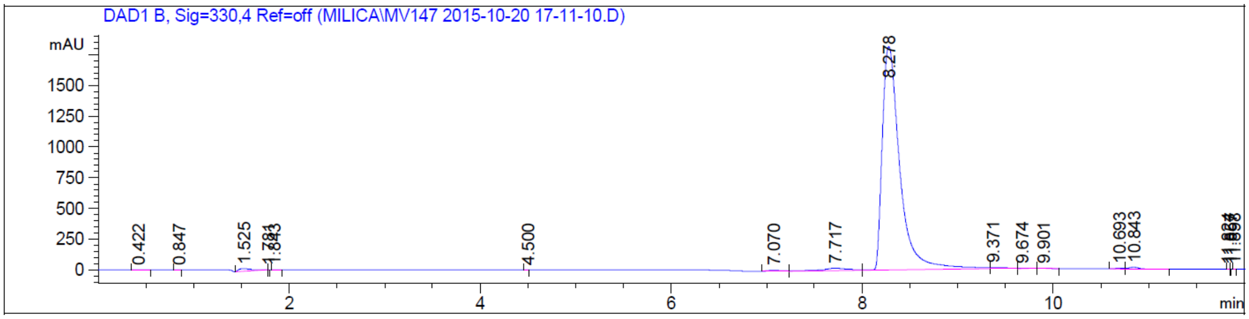

Peak #	RetTime [min]	Type	Width [min]	Area [mAU*s]	Height [mAU]	Area %
1	0.422	BB	0.0611	2.82612	5.53636e-1	0.0117
2	0.847	BV	0.0332	2.96316e-1	1.09217e-1	1.224e-3
3	1.525	BV	0.1431	210.84047	21.09748	0.8713
4	1.781	VB	9.10e-3	2.87732e-1	4.41475e-1	1.189e-3
5	1.843	BB	0.0523	1.02715	2.35891e-1	4.245e-3
6	4.500	BB	0.0206	1.37211e-1	8.59659e-2	5.670e-4
7	7.070	BV	0.1274	78.65191	8.23348	0.3250
8	7.717	VV	0.2327	376.93610	19.83461	1.5576
9	8.278	VV	0.1921	2.32117e4	1815.73730	95.9184
10	9.371	VV	0.1484	126.86670	10.10944	0.5243
11	9.674	VB	0.0869	24.97002	3.41009	0.1032
12	9.901	BB	0.0726	14.11047	2.34950	0.0583
13	10.693	BV	0.0754	40.38631	6.93634	0.1669
14	10.843	VB	0.1015	108.59284	15.07354	0.4487
15	11.824	BB	0.0111	1.02296	1.31299	4.227e-3
16	11.857	BB	5.50e-3	1.64866e-1	4.80579e-1	6.813e-4
17	11.898	BB	8.09e-3	6.00064e-1	1.25231	2.480e-3
Totals :				2.41994e4	1907.25384	

Method D

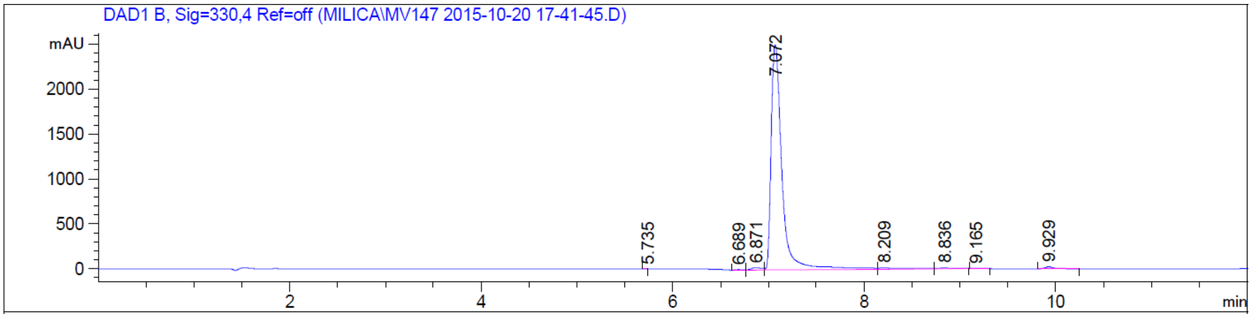

Peak #	RetTime [min]	Type	Width [min]	Area [mAU*s]	Height [mAU]	Area %
1	5.735	BB	0.0240	1.07254e-1	5.57080e-2	5.243e-4
2	6.689	BV	0.0761	35.09261	6.81063	0.1716
3	6.871	VV	0.1151	208.44798	26.55494	1.0190
4	7.072	VV	0.1089	1.97024e4	2500.60498	96.3184
5	8.209	VV	0.2473	301.50256	14.45508	1.4739
6	8.836	VB	0.1063	83.81390	10.47714	0.4097
7	9.165	BB	0.0662	1.79404	3.28806e-1	8.770e-3
8	9.929	BB	0.0911	122.33539	20.34780	0.5981
Totals :				2.04555e4	2579.63507	

Compound: 40

Method C

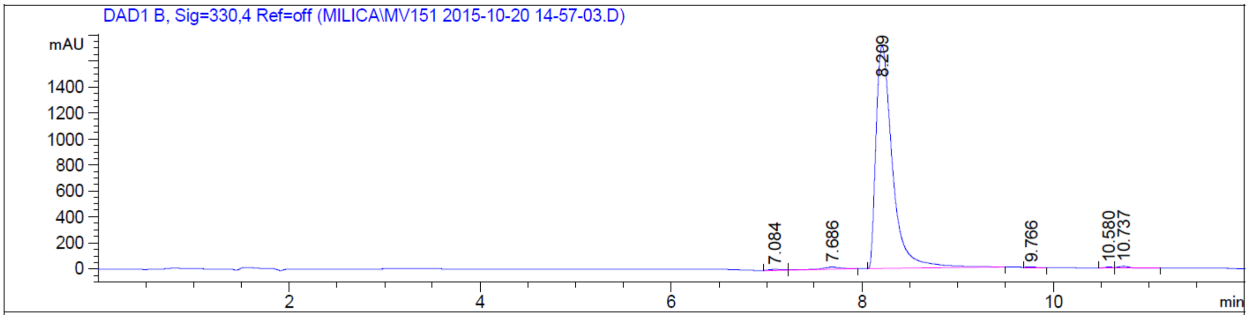

Peak #	RetTime [min]	Type	Width [min]	Area [mAU*s]	Height [mAU]	Area %
1	7.084	BV	0.1096	62.42950	7.50396	0.2966
2	7.686	VB	0.1675	196.83829	15.10379	0.9353
3	8.209	BB	0.1824	2.06286e4	1714.65063	98.0206
4	9.766	BB	0.0717	13.48948	2.27542	0.0641
5	10.580	BV	0.0724	39.16399	6.53230	0.1861
6	10.737	VB	0.1066	104.63895	14.10237	0.4972
Totals :				2.10452e4	1760.16847	

Method D

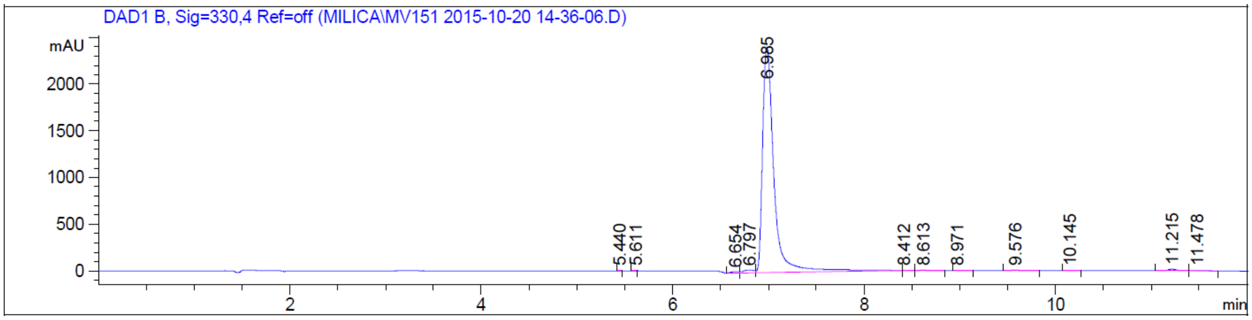

Peak #	RetTime [min]	Type	Width [min]	Area [mAU*s]	Height [mAU]	Area %
1	5.440	BB	0.0218	1.00664e-1	5.79854e-2	4.883e-4
2	5.611	BB	0.0220	1.14201e-1	6.50129e-2	5.540e-4
3	6.654	BV	0.0846	87.79340	15.84885	0.4259
4	6.797	VV	0.1108	249.82582	32.44704	1.2119
5	6.985	VV	0.1246	2.00783e4	2411.83472	97.4014
6	8.412	VB	0.0573	12.32068	2.66933	0.0598
7	8.613	BB	0.0702	16.85452	2.90565	0.0818
8	8.971	BB	0.0790	2.19960	3.31078e-1	0.0107
9	9.576	BB	0.0835	41.53010	7.01789	0.2015
10	10.145	BB	0.0679	1.91939	3.42590e-1	9.311e-3
11	11.215	BV	0.0941	113.18070	17.94900	0.5490
12	11.478	VB	0.0929	9.82690	1.30380	0.0477
Totals :				2.06140e4	2492.77293	

Compound: 41

Method A

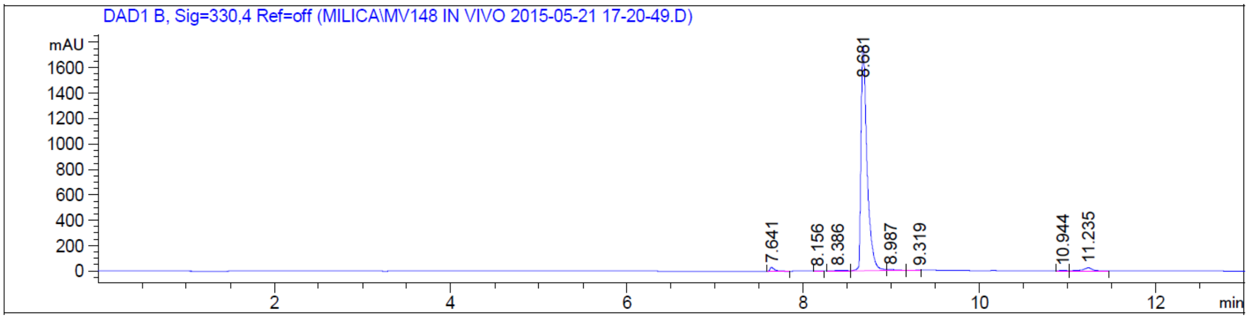

Peak #	RetTime [min]	Type	Width [min]	Area [mAU*s]	Height [mAU]	Area %
1	7.641	BB	0.0501	108.00912	31.43413	1.2242
2	8.156	BB	0.0447	7.95650	2.36894	0.0902
3	8.386	BB	0.0794	23.89058	3.93031	0.2708
4	8.681	BV	0.0716	8400.87695	1764.00928	95.2194
5	8.987	VB	0.0910	65.83261	10.30554	0.7462
6	9.319	BV	0.0675	17.72163	3.39033	0.2009
7	10.944	VV	0.0625	19.06977	3.70876	0.2161
8	11.235	VB	0.1043	179.29144	24.67681	2.0322
Totals :				8822.64861	1843.82410	

Method B

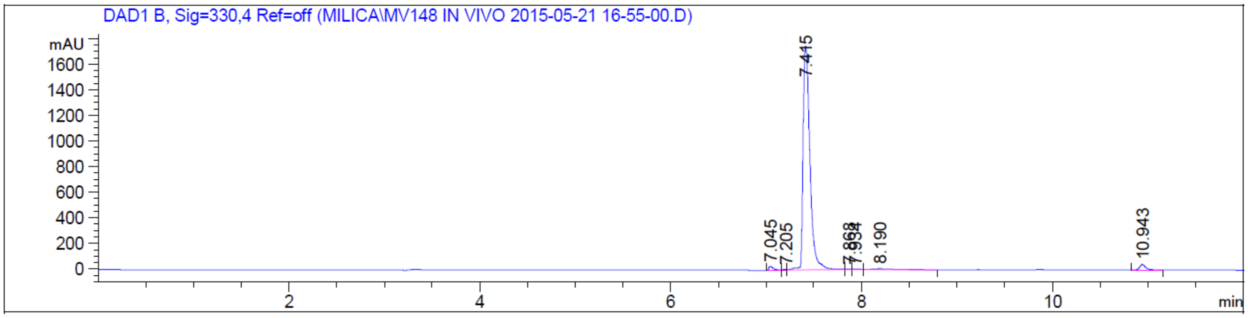

Peak #	RetTime [min]	Type	Width [min]	Area [mAU*s]	Height [mAU]	Area %
1	7.045	BB	0.0501	93.71075	29.83362	1.0323
2	7.205	BV	0.0317	6.64790	2.70537	0.0732
3	7.415	VV	0.0774	8691.20410	1751.03418	95.7429
4	7.868	VV	0.0573	15.86938	3.61076	0.1748
5	7.934	VB	0.0522	14.12953	3.57062	0.1557
6	8.190	BB	0.1407	69.97594	6.39396	0.7709
7	10.943	BV	0.0652	186.11200	42.81100	2.0502
Totals :				9077.64961	1839.95951	

Compound: 42

Method D

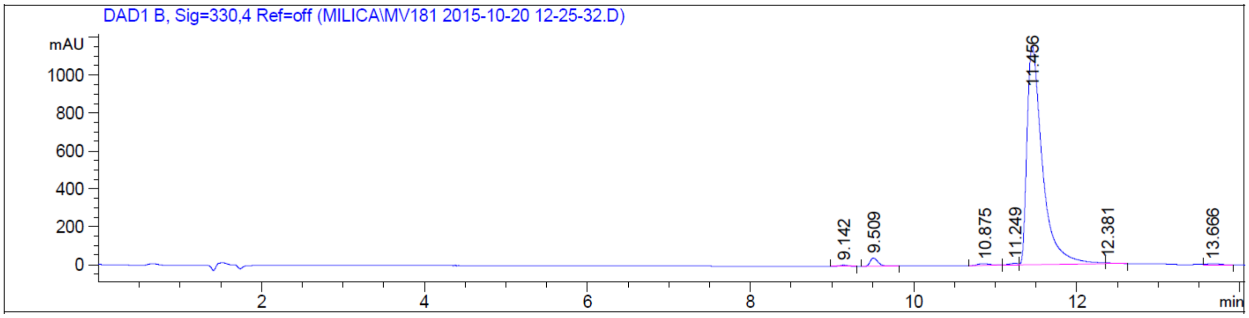

Peak #	RetTime [min]	Type	Width [min]	Area [mAU*s]	Height [mAU]	Area %
1	9.142	BB	0.0884	40.84436	6.54013	0.2585
2	9.509	BB	0.1027	281.94089	42.63459	1.7841
3	10.875	BB	0.1335	88.58769	8.10531	0.5606
4	11.249	BV	0.0864	51.77385	7.37327	0.3276
5	11.456	VV	0.1951	1.52084e4	1155.23315	96.2378
6	12.381	VV	0.1265	59.23616	5.57472	0.3748
7	13.666	VB	0.1326	72.15301	6.42168	0.4566
Totals :				1.58030e4	1231.88284	

Method G

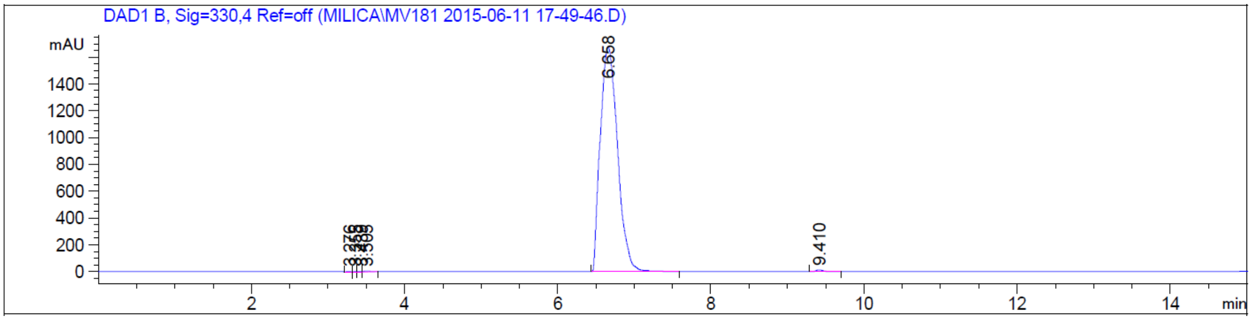

Peak #	RetTime [min]	Type	Width [min]	Area [mAU*s]	Height [mAU]	Area %
1	3.276	BB	0.0565	16.32383	4.95032	0.0619
2	3.355	BV	0.0352	7.34878	3.20923	0.0279
3	3.439	VV	0.0529	16.47764	4.18833	0.0625
4	3.505	VB	0.0779	29.29096	4.47131	0.1111
5	6.658	BB	0.2200	2.62094e4	1680.20728	99.4388
6	9.410	BB	0.0876	78.48502	13.65381	0.2978
Totals :				2.63574e4	1710.68028	

Compound: 43

Method C

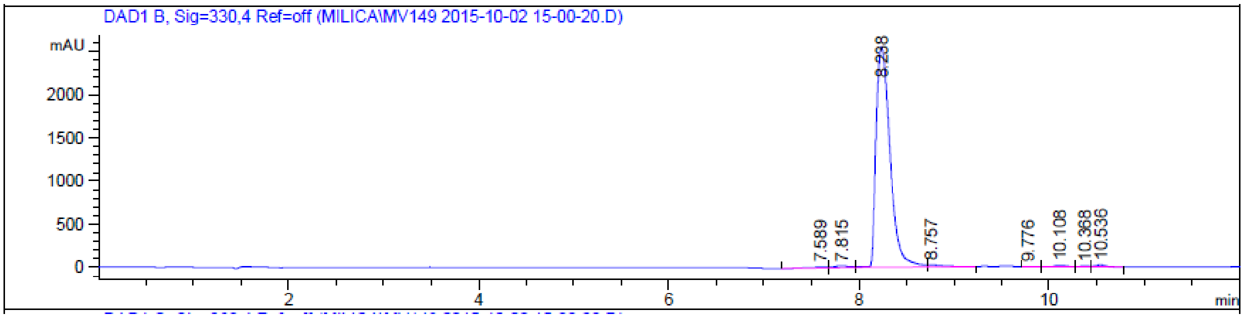

Peak #	RetTime [min]	Type	Width [min]	Area [mAU*s]	Height [mAU]	Area %
1	7.589	BV	0.1793	157.24036	10.37813	0.5796
2	7.815	VV	0.1532	260.49203	21.51618	0.9601
3	8.238	VV	0.1220	2.61074e4	2558.75122	96.2292
4	8.757	VB	0.1324	259.51468	23.12087	0.9565
5	9.776	VB	0.0797	16.24070	2.52059	0.0599
6	10.108	BB	0.1147	128.51112	16.34383	0.4737
7	10.368	BV	0.0879	49.34284	8.17360	0.1819
8	10.536	VB	0.1040	151.68288	21.44783	0.5591

Totals : 2.71304e4 2662.25225

Method D

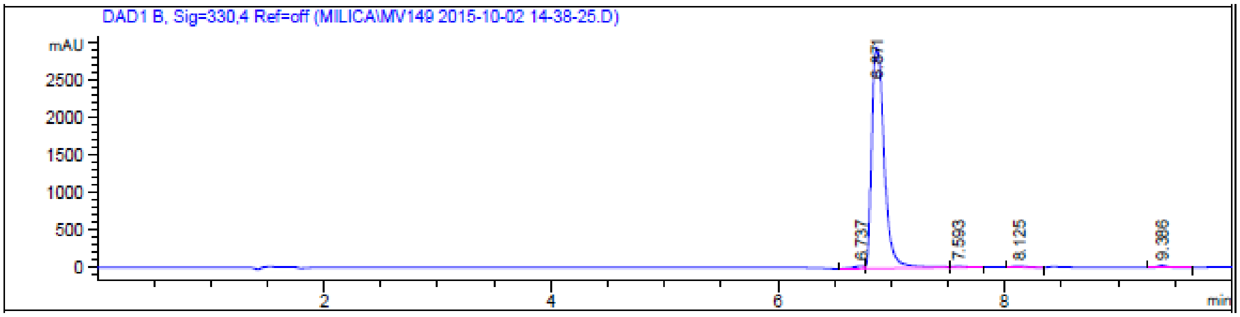

Peak #	RetTime [min]	Type	Width [min]	Area [mAU*s]	Height [mAU]	Area %
1	6.737	BV	0.0964	243.48349	37.20078	1.0436
2	6.871	VV	0.0929	2.26023e4	2934.04883	96.8746
3	7.593	VB	0.1159	150.26065	17.89824	0.6440
4	8.125	BB	0.1069	146.13326	20.19939	0.6263
5	9.386	BV	0.0844	189.32347	34.30740	0.8115

Totals : 2.33315e4 3043.65464

Compound: 44

Method A

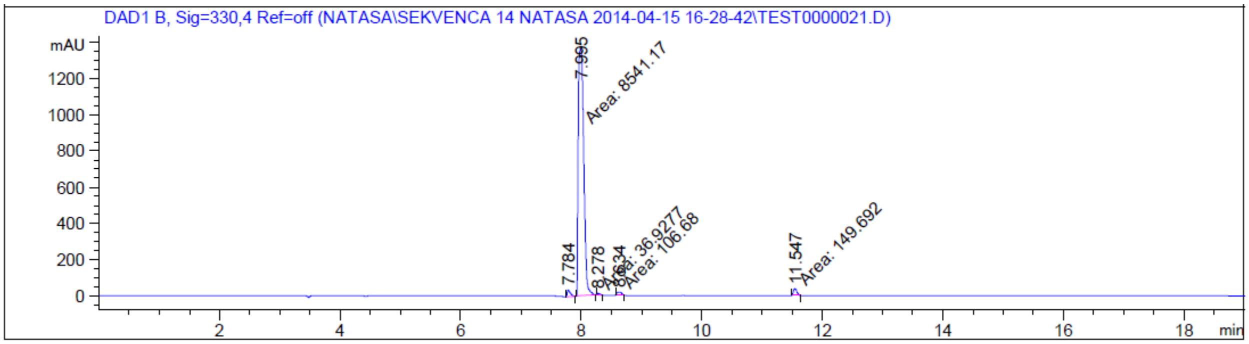

Peak #	RetTime [min]	Type	Width [min]	Area [mAU*s]	Height [mAU]	Area %
1	7.784	BB	0.0549	137.49791	36.94980	1.5325
2	7.995	MM	0.1044	8541.16699	1363.20300	95.1984
3	8.278	MM	0.0676	36.92768	9.09978	0.4116
4	8.634	MM	0.0909	106.68042	19.57051	1.1890
5	11.547	MM	0.0657	149.69228	37.99706	1.6684

Totals : 8971.96528 1466.82016

Method B

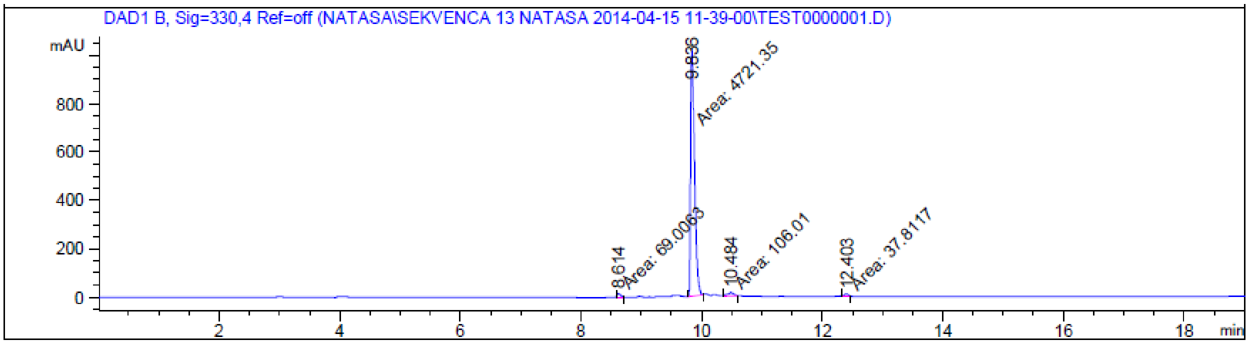

Peak #	RetTime [min]	Type	Width [min]	Area [mAU*s]	Height [mAU]	Area %
1	8.614	MM	0.0698	69.00631	16.48519	1.3985
2	9.836	MM	0.0770	4721.34766	1021.60065	95.6867
3	10.484	MM	0.1201	106.00954	14.70533	2.1485
4	12.403	MM	0.0679	37.81166	9.28764	0.7663

Totals : 4934.17516 1062.07880

Compound: 46

Method I

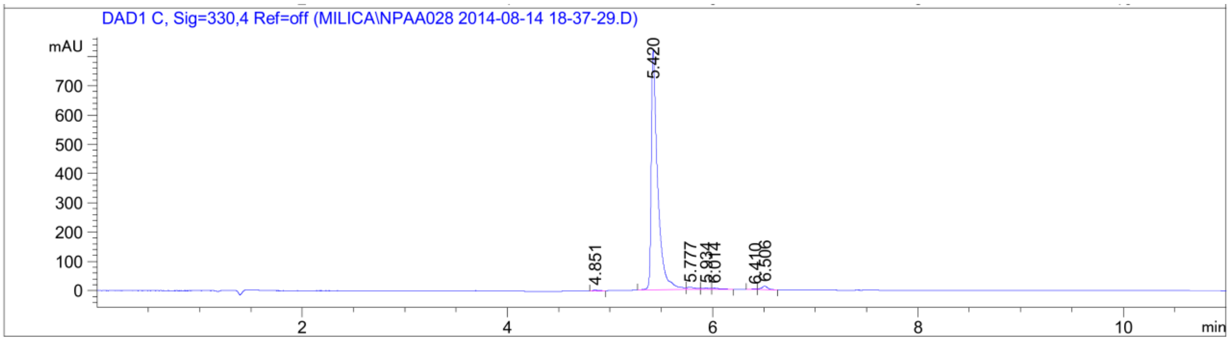

Peak #	RetTime [min]	Type	Width [min]	Area [mAU*s]	Height [mAU]	Area %
1	4.851	BB	0.0531	11.55937	3.20699	0.3092
2	5.420	BV	0.0611	3560.69458	821.58832	95.2354
3	5.777	VV	0.0796	51.85744	8.28047	1.3870
4	5.934	VV	0.0744	28.90337	4.86178	0.7731
5	6.014	VB	0.0706	25.86342	4.70832	0.6918
6	6.410	BV	0.0422	9.21101	2.64298	0.2464
7	6.506	VB	0.0626	50.74789	11.81134	1.3573
Totals :				3738.83708	857.10019	

Method H

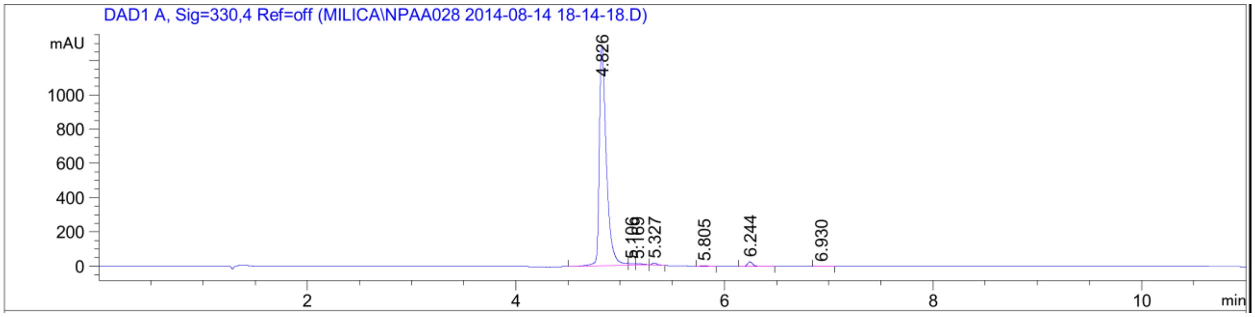

Peak #	RetTime [min]	Type	Width [min]	Area [mAU*s]	Height [mAU]	Area %
1	4.826	BV	0.0704	6058.60107	1287.40662	96.5369
2	5.106	VV	0.0529	37.04706	9.40642	0.5903
3	5.169	VB	0.0597	33.36654	7.67828	0.5317
4	5.327	BB	0.0535	34.47512	9.93727	0.5493
5	5.805	BB	0.0698	11.68613	2.42034	0.1862
6	6.244	BB	0.0532	94.46011	26.75001	1.5051
7	6.930	BB	0.0577	6.30531	1.52711	0.1005
Totals :				6275.94134	1345.12604	

Compound: 52

Method A

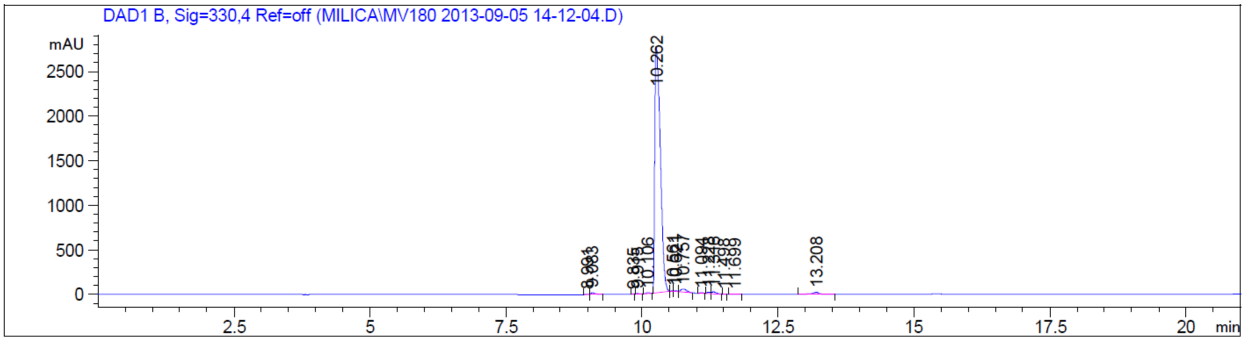

Peak #	RetTime [min]	Type	Width [min]	Area [mAU*s]	Height [mAU]	Area %
1	8.991	BV	0.0582	19.20522	4.90571	0.0934
2	9.083	VV	0.0714	116.13204	22.98373	0.5646
3	9.835	BB	0.0425	4.62732	1.79770	0.0225
4	9.915	BB	0.0548	23.58116	6.28665	0.1146
5	10.106	BB	0.0674	52.62742	11.61486	0.2559
6	10.262	BB	0.0970	1.96133e4	2758.01270	95.3572
7	10.561	BV	0.0388	20.52732	8.47703	0.0998
8	10.621	VB	0.0609	40.19218	9.69200	0.1954
9	10.757	BB	0.1171	245.83640	34.52837	1.1952
10	11.094	BB	0.0684	29.20466	7.04172	0.1420
11	11.228	BV	0.0688	64.26157	15.21992	0.3124
12	11.316	VB	0.0799	124.99713	22.99112	0.6077
13	11.498	BB	0.0396	1.01013	3.78477e-1	4.911e-3
14	11.699	BB	0.0980	6.47267	7.94974e-1	0.0315
15	13.208	BB	0.1227	206.26627	23.21161	1.0028
Totals :				2.05683e4	2927.93657	

Method B

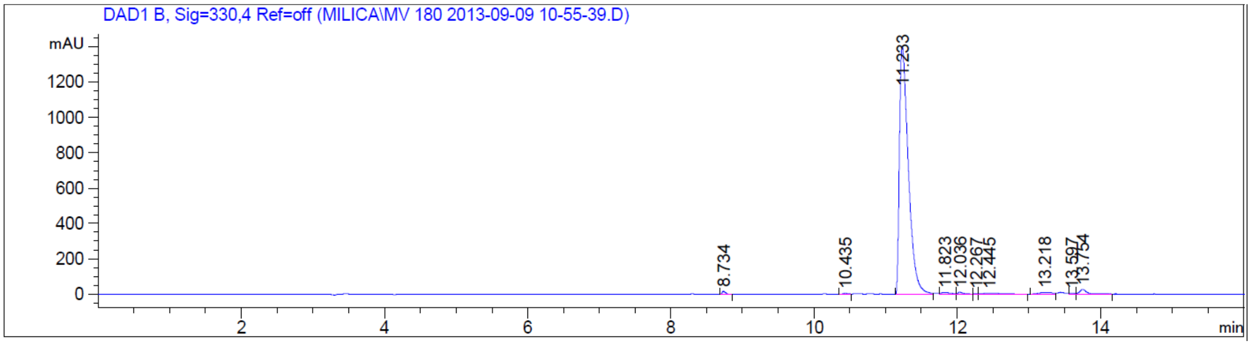

Peak #	RetTime [min]	Type	Width [min]	Area [mAU*s]	Height [mAU]	Area %
1	8.734	BB	0.0510	59.07262	17.68832	0.4644
2	10.435	BV	0.0698	26.55964	5.60821	0.2088
3	11.233	BV	0.1304	1.21063e4	1399.71387	95.1654
4	11.823	VV	0.1312	79.11050	8.72864	0.6219
5	12.036	VB	0.0730	48.03447	9.75311	0.3776
6	12.267	BV	0.0489	3.74937	1.20084	0.0295
7	12.445	VB	0.1986	94.13501	6.09839	0.7400
8	13.218	BV	0.1745	111.99912	7.59500	0.8804
9	13.597	VV	0.0710	23.11556	4.49778	0.1817
10	13.754	VB	0.0932	169.25041	26.26011	1.3304
Totals :				1.27213e4	1487.14425	
